# Polyanionic Receptors for Carboxylates in Water

**DOI:** 10.1002/anie.202413505

**Published:** 2024-10-18

**Authors:** Xudong Ren, Alister J. Flint, Daniel Austin, Anthony P. Davis

**Affiliations:** ^1^ School of Chemistry University of Bristol Cantock's Close Bristol BS8 1TS United Kingdom

**Keywords:** Molecular Recognition, Supramolecular Chemistry, Anion Receptors, Biomimetic Receptors, Aqueous Solution

## Abstract

Receptors for carboxylate anions have many possible biomedical applications, including mimicry of the vancomycin group of antibiotics. However, binding carboxylates in water, the biological solvent, is highly challenging due to the hydrophilicity of these polar anions. Here we report, for the first time, the recognition of simple carboxylates such as acetate and formate in water by synthetic receptors with charge‐neutral binding sites. The receptors are solubilised by polyanionic side‐chains which, remarkably, do not preclude anion binding. The tricyclic structures feature two identical binding sites linked by polyaromatic bridges, capable of folding into closed, twisted conformations. This folding is hypothesised to preorganise the structures for anion recognition, mimicking the process which generates many protein binding sites. The architecture is suitable for elaboration into enclosed structures with potential for selective recognition of biologically relevant carboxylates.

## Graphical abstract

Carboxylate anions are important but challenging substrates for recognition in aqueous solution. These divalent receptors employ charge‐neutral binding sites to bind simple carboxylates in water with no cosolvent, despite the presence of multiply anionic solubilising groups.

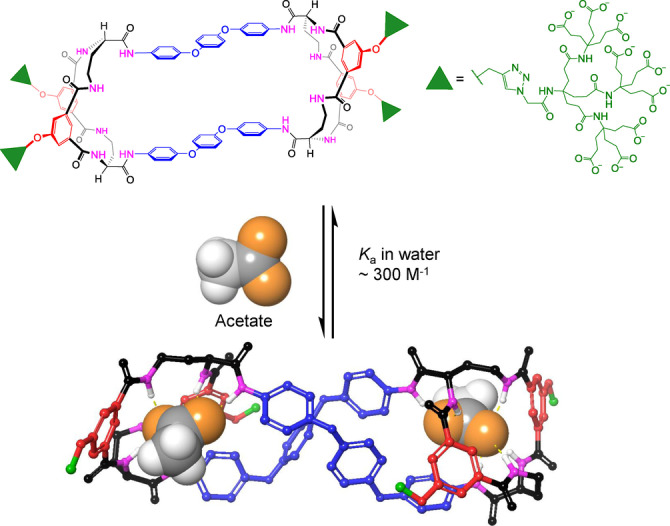

## Introduction

Anionic species play many important roles in biology. Agents which bind anions selectively could therefore have valuable effects or applications,[^1^, ^2^, ^3^] provided they can operate in the aqueous biological medium. However, most biologically relevant anions are hydrophilic and well‐solvated by water, so achieving strong binding in aqueous solution is challenging.[^4^, ^5^, ^6^] Moreover, designing host molecules which are soluble and monomeric in water is not straightforward. Consequently, most of the work carried out in this area employs organic cosolvents to solubilise host molecules.[^6^, ^7^, ^8^, ^9^, ^10^, ^11^] Positive charges can help with both affinity and solubility, but it is notable that many natural anion‐binding sites are charge‐neutral,[^12^, ^13^] and it may be that positive charges can promote non‐selective binding or otherwise hinder applications.^[14]^ Of the few examples of charge‐neutral synthetic anion receptors operating in water, most seem to favour hydrophobic anions,[^15^, ^16^, ^17^, ^18^] with the exception of two elegant systems which have been shown to bind sulfate[^14^, ^19^] and one which favours phosphate.^[20]^

**Figure 1:**
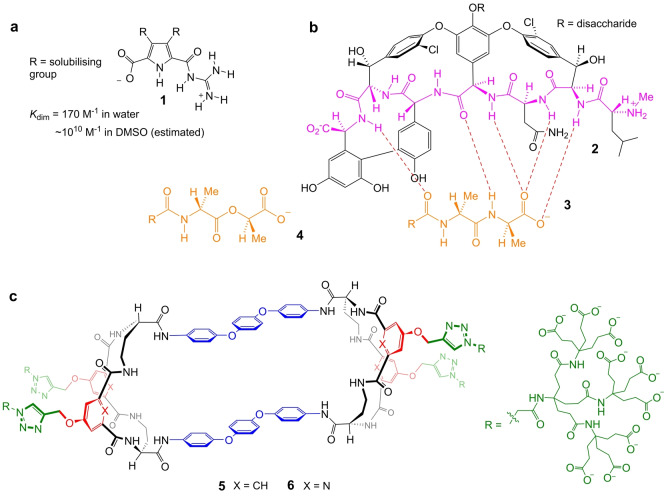
Molecular formulae: (a) Schmuck's self‐complementary guanidinium **1**, demonstrating carboxylate recognition in water. (b) Vancomycin **2** binding its target D‐Ala‐D‐Ala **3**. The *N*‐methylleucine unit is positively charged but doesn't interact directly with the carboxylate,^[13]^ hence the binding site may be considered charge‐neutral. Also shown is the D‐Ala‐D‐Lac unit **4** which replaces **3** in resistant bacteria. (c) The tricyclic divalent carboxylate receptors described in this paper. Although side chains are shown as fully ionized for simplicity, some carboxylate groups will be protonated at near‐neutral pH. For further discussion, see text.

One anionic unit of special biological importance is the carboxylate. This group appears widely in proteins, peptides, oligosaccharides and biochemical intermediates, so that selective carboxylate receptors could enable a wide variety of applications. However, it is also an especially challenging substrate to target in water, being among the most strongly hydrated of the common monovalent anions.[^21^, ^22^, ^23^] Synthetic receptors for carboxylates in aqueous media have generally exploited positively charged binding units, and are rarely employed in pure water without organic cosolvents.[^4^, ^6^, ^24^] For example, the guanidiniocarbonyl pyrrole unit as in **1** (Figure 1a), introduced by Schmuck, is positively charged and has mainly been studied in organic‐aqueous mixtures.[^25^, ^26^, ^27^] **1** itself is self‐complementary, soluble in nearly pure water and dimerises with *K*_dim_=170 m ^−1^, the result of two guanidinium‐carboxylate interactions.^[28]^ By contrast, the estimated *K*_dim_ for **1** in DMSO is 10^10^ m ^−1^, emphasising the special challenge of water as solvent. As far as we know, there are no examples where synthetic receptors with charge‐neutral binding sites have been shown to bind simple carboxylates in water.

Among the most important carboxylate targets is the D‐Ala−D‐Ala *C*‐terminal peptide **3**. This unit plays a key role in bacterial cell‐wall biosynthesis, and is bound selectively by vancomycin **2** and related glycopeptide antibiotics (Figure 1b).[^29^, ^30^] There has been long‐standing interest in designing synthetic molecules which could mimic **2** and serve as prototypes for new families of antibiotics.[^31^, ^32^, ^33^, ^34^, ^35^] Especially important would be agents which could bind both **3** and D‐Ala−D‐Lac **4**, which replaces **3** in some vancomycin‐resistant bacteria.^[30]^ Vancomycin has a charge‐neutral carboxylate binding site incorporated in an extended structure capable of hydrogen bonding and apolar interactions with other parts of its target.^[13]^ There is therefore special interest in neutral systems capable of binding carboxylates with potential for elaboration into more complex, receptor‐like structures.

Herein we report the first synthetic receptors with charge‐neutral binding sites that complex simple carboxylates with appreciable affinities in water. Tricyclic receptors **5** and **6** (Figure 1c) feature twin binding sites and achieve binding constants *K*_a_ ~300 m ^−1^ in water for substrates such as acetate and formate. Remarkably, these results are achieved despite the presence of solubilising groups bearing multiple negative charges. The mechanism of binding appears to involve folding of the tricyclic structure, reminiscent of the processes which generate protein binding sites. Moreover, the molecular architecture has potential for elaboration towards selective receptors for more complex substrates of biological importance.

## Results and Discussion

### Receptor Design and Preliminary Experiments

**Figure 2:**
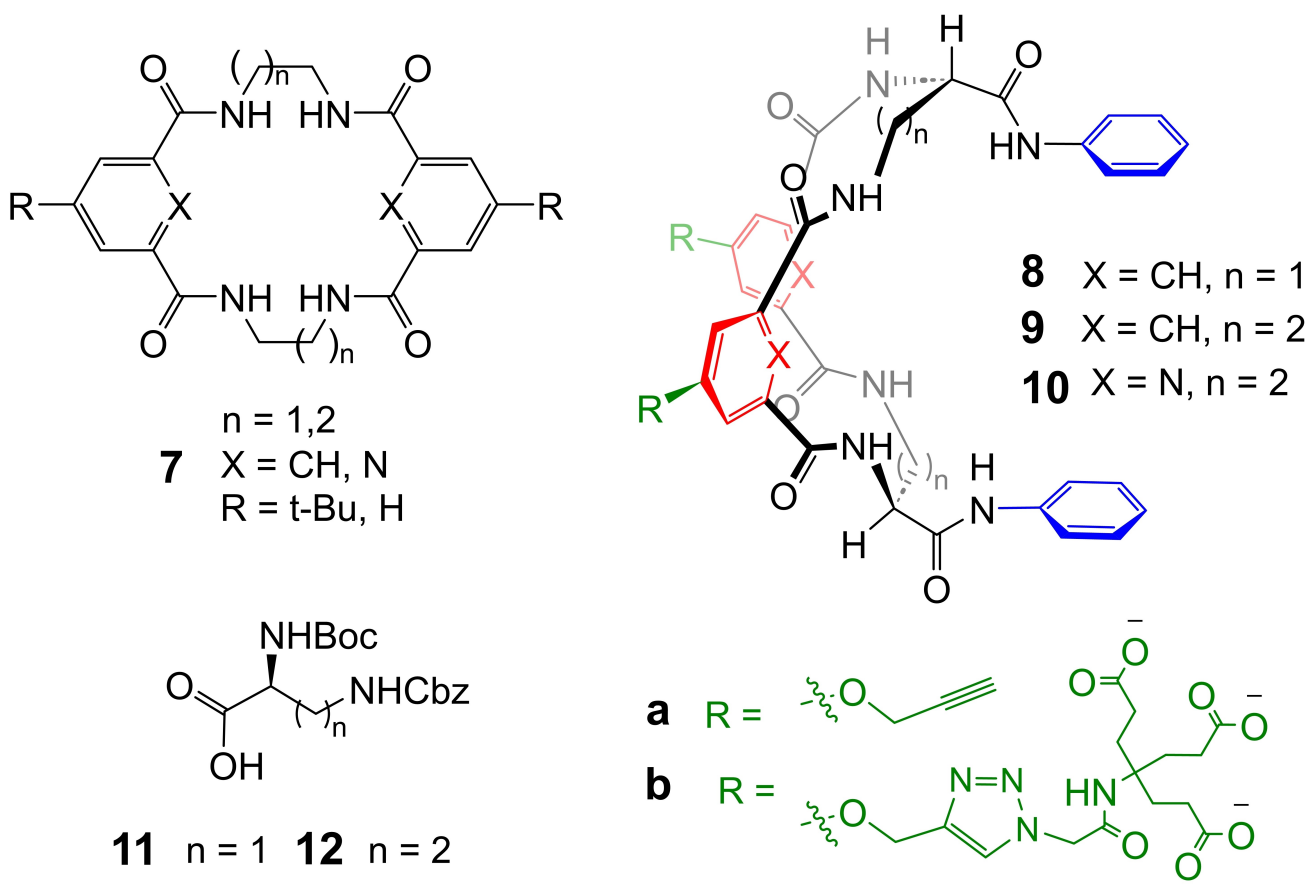
Structures of tetralactam anion receptors **7** from Jurczak, Chmielewski and co‐workers, monocyclic receptors **8**–**10** studied here in preliminary experiments, and precursor amino acid derivatives **11** and **12**.

This work represents the first steps in a long‐term program aimed at binding **3** and **4**, and thus generating new families of antibiotics. Our initial aim was to establish a carboxylate‐binding core that can be developed into three‐dimensional structures with clefts or cavities providing interactions with groups R’ in R'CO_2_^−^ targets. Charge‐neutral designs were preferred partly for practical reasons, but also encouraged by the neutral binding site of vancomycin (see Figure 1b). An attractive starting point was the tetralactam family of anion receptors **7** (Figure 2) studied by Jurczak, Chmielewski and co‐workers.[^36^, ^37^, ^38^] These macrocycles showed good affinities to carboxylates in DMSO, and possessed sp^3^ CH groups which could be substituted to generate cleft‐type architectures, e.g. by using diamino acid starting materials. Groups R could be modified to engender solubility in water. Because of uncertainty regarding the best solubilising groups to use, we decided to prepare alkynyl functionalised aromatic spacers and use Cu‐catalysed azide cycloadditions[^39^, ^40^] for late‐stage modifications.

Initial designs are exemplified by macrocycles **8**–**10** (Figure 2), prepared from amino acid derivatives **11** and **12** as described in the Supporting Information. As expected, the organic‐soluble **8 a**–**10 a** were found to be effective anion receptors in DMSO. For example ^1^H NMR titration of **9 a** with Bu_4_N^+^AcO^−^ caused signal movements up to 0.3 ppm, analysed using the programme Bindfit^[41]^ to yield *K*_a_ (1 : 1)=6200 m ^−1^ (see Figure S46). To achieve water‐solubility we attached PEG‐ and glucamine‐based charge‐neutral solubilising groups, as in **S13** and **S14** (see Supporting Information Section 1.9). However, neither could prevent self‐association at concentrations realistic for NMR studies, and we therefore resorted to the tricarboxylate unit shown. ^1^H NMR titrations of macrocycles **8 b**–**10 b** against acetate and chloride in water resulted in small signal movements (typically ~0.02 ppm), suggestive of binding. The data for **8 b** did not give satisfactory analyses using any binding model, but the titrations with **9 b** and **10 b** were consistent with 1 : 1 binding with very low affinities (e.g. 8 and 13 m ^−1^ for **9 b** with acetate and chloride respectively; see Figures S48 and S49). These preliminary experiments suggested that (a) we should focus on the larger 20‐membered macrocycles, which seemed to be more promising; (b) there was little difference between macrocycles containing benzene and pyridine units, so both should be pursued; and (c) negative charges did not preclude measurable anion binding. On the other hand the low affinities, especially to acetate, further emphasised the difficulty of binding hydrophilic anions in water.

### Tricyclic Octalactams 5 and 6

**Scheme 1:**
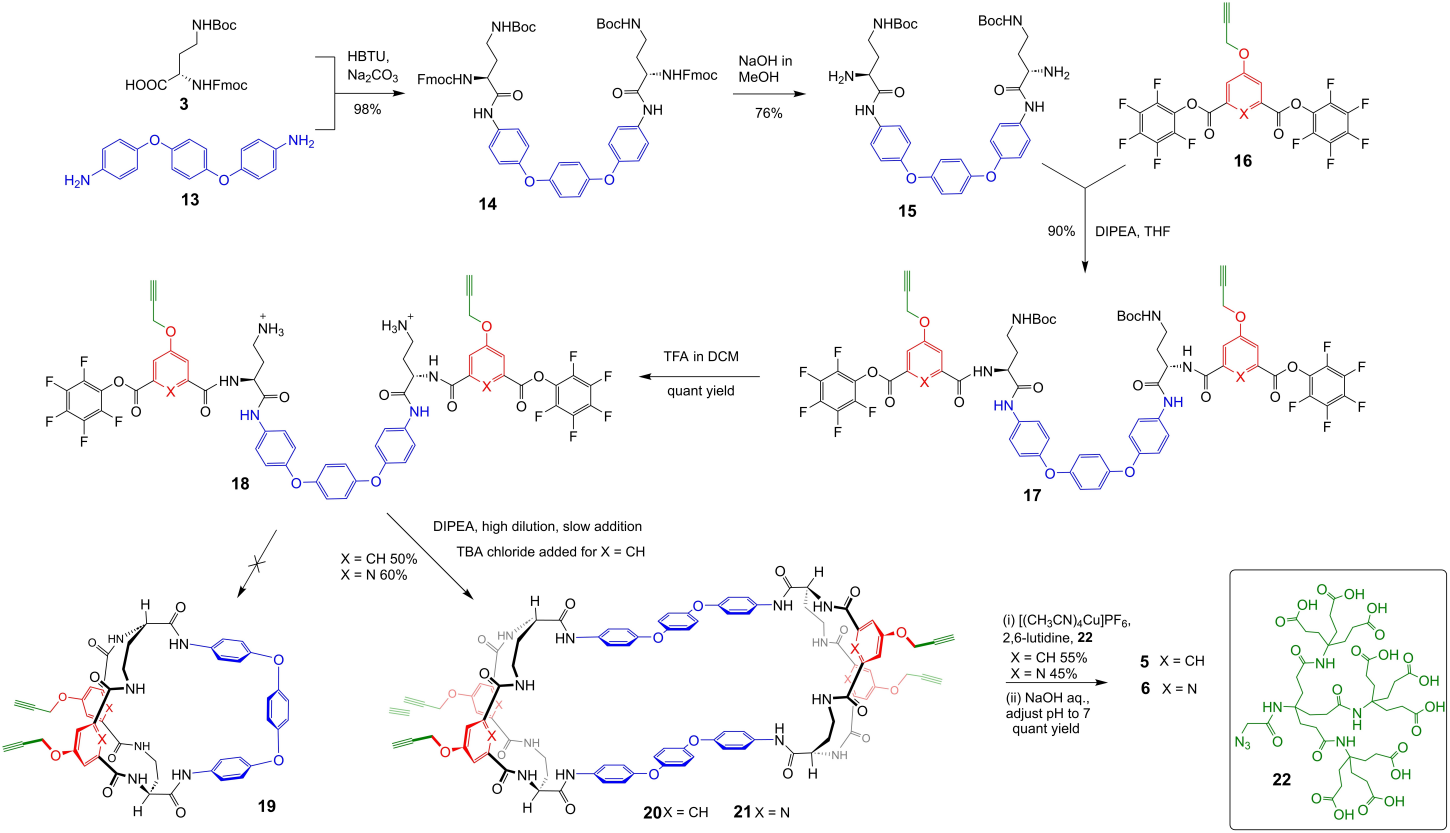
Synthetic route to receptors **5**, **6**, **20** and **21**. HBTU: 2‐(1*H*‐benzotriazol‐1‐yl)‐1,1,3,3‐tetramethyluronium hexafluorophosphate, DIPEA: N,N‐diisopropylethylamine, TFA: trifluoroacetic acid, TBA: tetrabutylammonium. For details of procedures see Supporting Information.

Our next aim was to raise affinities and control substrate‐selectivity by introducing further interactions. An obvious approach was to link the anilino groups in **9 b** and **10 b**, creating a bridge with a hydrophobic inner surface and generating an amphiphilic three‐dimensional binding site. The diamine **13** is commercially available, inexpensive, and seemed a good candidate for a bridging unit, as in **19**. We therefore embarked on the sequences outlined in Scheme 1. On reaching intermediates **18**, the expectation was that treatment with base to expose the NH_2_ groups would be followed by cyclisation to **19**. In fact, the major products isolated were the tricycles **20** and **21** in 50 % and 60 % yields respectively, derived from initial reaction between two molecules of **18** followed by cyclisations at either end. This unintended outcome presumably relates to the conformational properties of the bis(phenoxy)phenyl bridge in **18**, which may resist bending into the curved shape required for cyclisation. For **18** (X=CH), the cyclisation was inconveniently slow but could be accelerated by adding chloride ions as templates.

To achieve solubility in water, **20** and **21** were treated with azide **22** under copper catalysed “click/CuAAC” conditions to give **5** and **6**. After purification by chromatography, water (or D_2_O) was then added followed by NaOH to give solutions at pH≈7.4 for NMR studies. Dilution studies showed small signal movements down to 22 μm for **5** and 74 μm for **6**, but no further changes so the tricycles were taken to be monomeric below these concentrations (Figures S36 and S37). Similar cycloadditions were used to place smaller tricarboxylate groups, as used for **8 b**–**10 b**, at the four positions, but NMR dilution studies implied aggregation even at the lowest concentrations (Figure S40).

Tricycles **20**, **21**, **5** and **6** are essentially dimeric versions of **9** and **10** (ignoring the change in water‐solubilising groups), with identical binding sites at either end. The binding sites are well‐spaced, but as they are connected via a macropolycyclic architecture they may not behave independently, so that analysis according to the simple statistical 1 : 2 model (the divalent equivalent of 1 : 1 stoichiometry)^[42]^ may not be appropriate. Indeed, ^1^H NMR titrations of **20** and **21** against anions in DMSO‐*d* ^6^ gave very poor fits to the statistical model, with signals often changing their direction of movement part way through the titration (see Figures S54–S59). Alternative models, available in Bindfit, are 1 : 2 non‐cooperative, which assumes that affinities of the two binding sites are the same but that chemical shifts of protons in the 1 : 1 and 1 : 2 complexes are independent of each other, and 1 : 2 cooperative which allows for different affinities.[^41^, ^42^] Of these two, the 1 : 2 non‐cooperative model gave better fits with lower errors, so was adopted for this work. The results implied that incorporation in the “dimeric” tricyclic architecture made little difference to the tetralactams’ binding ability in DMSO. For example, titration of **20** with Bu_4_N^+^AcO^−^ in DMSO‐*d* ^6^ yielded *K*_a_=7560 m ^−1^, very similar to the value of 6600 m ^−1^ measured for **9 a**.

For studies of **5** and **6** in water, a first question was whether the side‐chain carboxylates might bind in an intramolecular fashion, potentially preventing the molecules acting as receptors. Examination of models suggested that this should be possible, though not necessarily strongly favoured. A ^1^H NMR pH titration on **5** was performed, revealing downfield movements of ~0.14 ppm between pH 7.7 and 9.0 for the isophthalamide CH*d* and amide NH*a* and NH*b* signals (see Figure S41). These protons should make contact with bound anions, so the movements are consistent with side‐chain carboxylate binding. If this explanation is correct, the data also imply that empty binding sites were available at pH 7.7. We have previously established that the dendrimeric nonacarboxylate solubilising groups are not fully deprotonated at neutral pH,^[43]^ so more internal binding might be expected at increasing pH. Another factor might be the interaction between −CO_2_H and −CO_2_^−^ groups within the partially ionised dendrimers. If both groups are present they might bind to each other, making the carboxylate less available for the tetralactam binding site. In any case, the pH titration indicated that carboxylate recognition should be possible at near‐neutral pH, even if affinities might be lowered by intramolecular competition.

**Figure 3:**
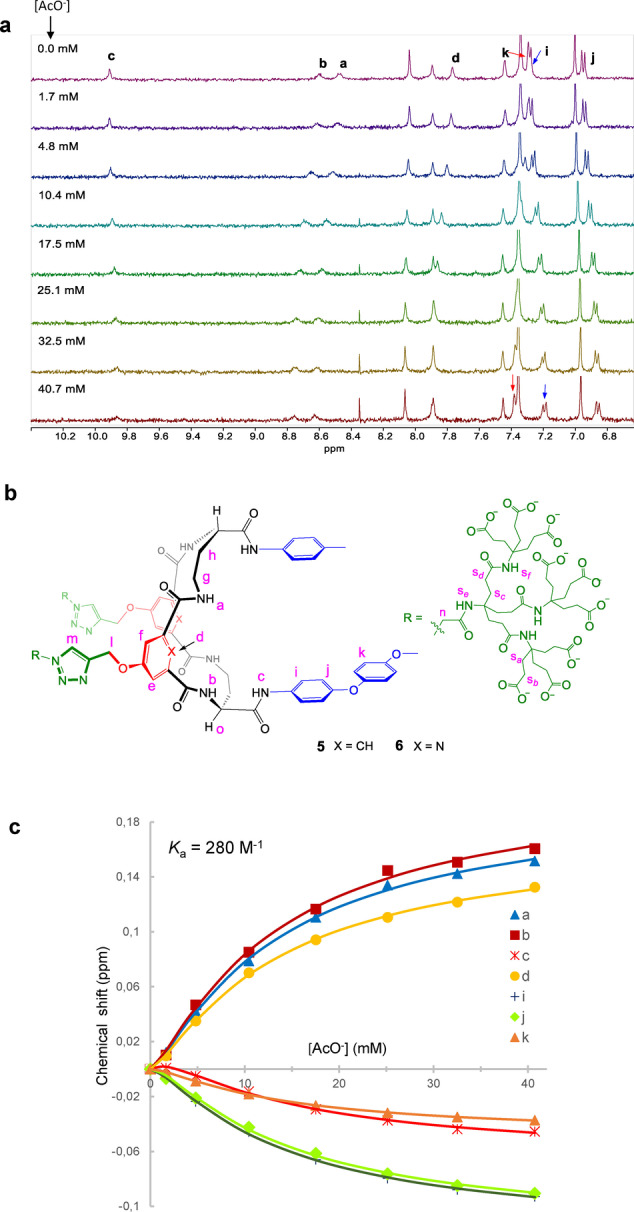
(a) Partial ^1^H NMR spectra from titration of receptor **5** (10 μM) with sodium acetate in 9 : 1 H_2_O/D_2_O at 298 K, pH=7.4; (b) Labelling system for **5** and **6** used in discussion; (c) Chemical shift movements from the spectra in (a) fitted to a 1 : 2 non‐cooperative binding model using Bindfit.

Tricycle **5** was then titrated against a number of carboxylate anions as sodium salts. Given the sensitivity of the spectra to changes in pH, all titrations were performed at a steady pH in the range 7.4–7.6. Where necessary, both D_2_O and 9 : 1 H_2_O/D_2_O were employed as solvents, allowing all protons to be followed. Spectra from a titration of **5** (10 μm) with sodium acetate are shown in Figure 3a (for signal labels, see Figure 3b). Significant movements are observed for several receptor signals. Consistent with the pH titration (see above) isophthalamide CH*d* and amide NH*a* and NH*b* moved downfield by ~0.16 ppm. The signals for NH*c*, CH*i*, CH*j* and CH*k* showed smaller up‐field movements ranging from 0.03 ppm to 0.08 ppm. Notably the signal movements were generally much larger than those for **9 b**+acetate, suggesting a qualitative difference between monomeric and dimeric water‐soluble receptors. As observed previously for **20**, the 1 : 2 statistical model was clearly inappropriate due to bidirectional signal movements, and the 1 : 2 non‐cooperative model gave lower errors than 1 : 2 cooperative. The 1 : 2 non‐cooperative model, as implemented in Bindfit, was therefore employed for all analyses. In the case of acetate (Figure 3c), the resulting affinity was ~280 m ^−1^, roughly thirty times that of **9 b** for acetate, and remarkably high considering the challenge of binding carboxylates in water and the net negative charge on the receptor. The affinity is all the more impressive given that some interference from side‐chain carboxylates is likely at the near‐neutral pH.

**Figure 4:**
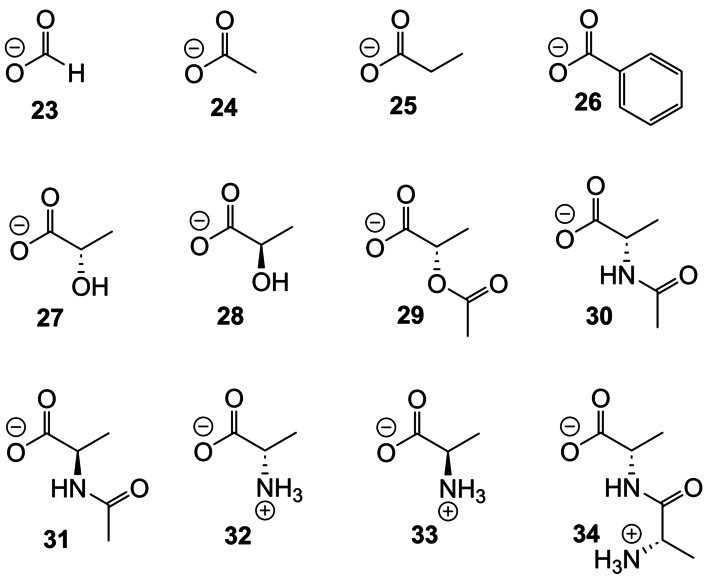
Carboxylate anions and zwitterions employed as substrates in this work.

Figure 4 shows the carboxylate anions tested as substrates for receptor **5** and Table 1 lists the measured binding constants. Some data are also given for tricycle **6**, but as this receptor lacks the relatively sharp and mobile^1^H NMR signal for CH*d*, it was more difficult to study and received less attention. In general, results for **5** and **6** were fairly similar. An interesting aspect of the results in Table 1 is the lack of selectivity between different carboxylates. By extending to polycyclic structures, we had expected to reinforce binding with hydrophobic interactions. However, the fact that formate, acetate, propionate and benzoate gave such similar results suggests that polar interactions are dominant. It seems that incorporation of the tetralactam unit in the tricyclic structure generates a binding site for carboxylates in general, sufficiently powerful to be effective in water. Some enantioselectivity might be expected given the chiral structure of **5**, and indeed D‐lactate **27** was bound ~1.5 times more strongly than L‐lactate **28**. The three amino acids tested caused almost no movements of receptor signals suggesting that the −NH_3_^+^ groups somehow interfere with binding, possibly by strengthening substrate hydration.

**Table 1:** Affinities of receptors **5** and **6** to anionic and zwitterion substrates, in water at pH ~7.5.

Substrate	Affinities (*K*_a_, m ^−1^) ^[a]^	
	Receptor **5** ^[a]^	Receptor **6**
Formate **23**	270 (±1.8 %)	
Acetate **24**	280 (±1.4 %)	320 (±5.4 %)
Propionate **25**	420 (±1.8 %)	310 (±5.0 %)
Benzoate **26**	230 (±1.0 %)	320 (±5.4 %)
*L–*Lactate **27**	210 (±1.6 %)	
*D*‐Lactate **28**	310 (±3.5 %)	
*O*‐Ac‐*L–*Lactate **29**	330 (±1.8 %)	
*N*‐Ac‐*L*‐Alanine **30**	180 (±1.3 %)	
*N*‐Ac‐*D*‐Alanine **31**	210 (±1.3 %)	
*L*‐Alanine **32**	NB ^[b]^	
*D*‐Alanine **33**	NB	
*L‐*Ala*‐L‐*Ala **34**	NB	
Chloride	120 (±0.6 %)	
Bromide	90 (±1.2 %)	
Iodide	30 (±0.6 %)	
Nitrate	110 (±1.7 %)	
Sulfate	410 (±3.3 %)	

[a] Measured by ^1^H NMR titration, receptor chemical shift movements analysed according to a 1 : 2 non‐cooperative binding model. For further details, see SI. [b] NB=no evidence of binding.

Inorganic anions were also investigated and gave signal movements generally similar to carboxylates. The affinity towards sulfate was ~410 m ^−1^, roughly as strong as propionate. Notably, the order of affinities SO_4_^2−^>AcO^−^>Cl^−^>Br^−^>I^−^ parallels the Hofmeister series, favouring anions which are good H‐bond acceptors. This is also the order commonly found for anion receptors in organic media,[^1^, ^2^, ^3^, ^22^] and reinforces the notion that binding occurs through direct polar interactions. Here, of course, the differences are muted by solvation, as the best H‐bond acceptors are also the most strongly hydrated. The behaviour of **5** provides an interesting contrast to other charge‐neutral water‐soluble binding sites which favour hydrophobic anions and presumably operate via different principles.[^15^, ^16^, ^18^, ^19^]

Seeking a method to confirm the binding constants, we attempted to use Isothermal Titration Calorimetry (ITC). Unfortunately the heats generated during titrations at [**5**]=20 μm were insufficient for analysis. However, data obtained at [**5**]=250 μm could be analysed in some cases and were broadly consistent with the NMR results, allowing for the fact that receptor aggregation at this concentration should suppress apparent affinities (see Figures S85–S90 and Table S1). For example, *K*_a_ values for **5**+propionate and sulfate were measured at ~125 m ^−1^ and ~230 m ^−1^ respectively. For carboxylates and sulfate, binding was entropy‐driven with small positive enthalpies.

### Structural Insights

**Figure 5:**
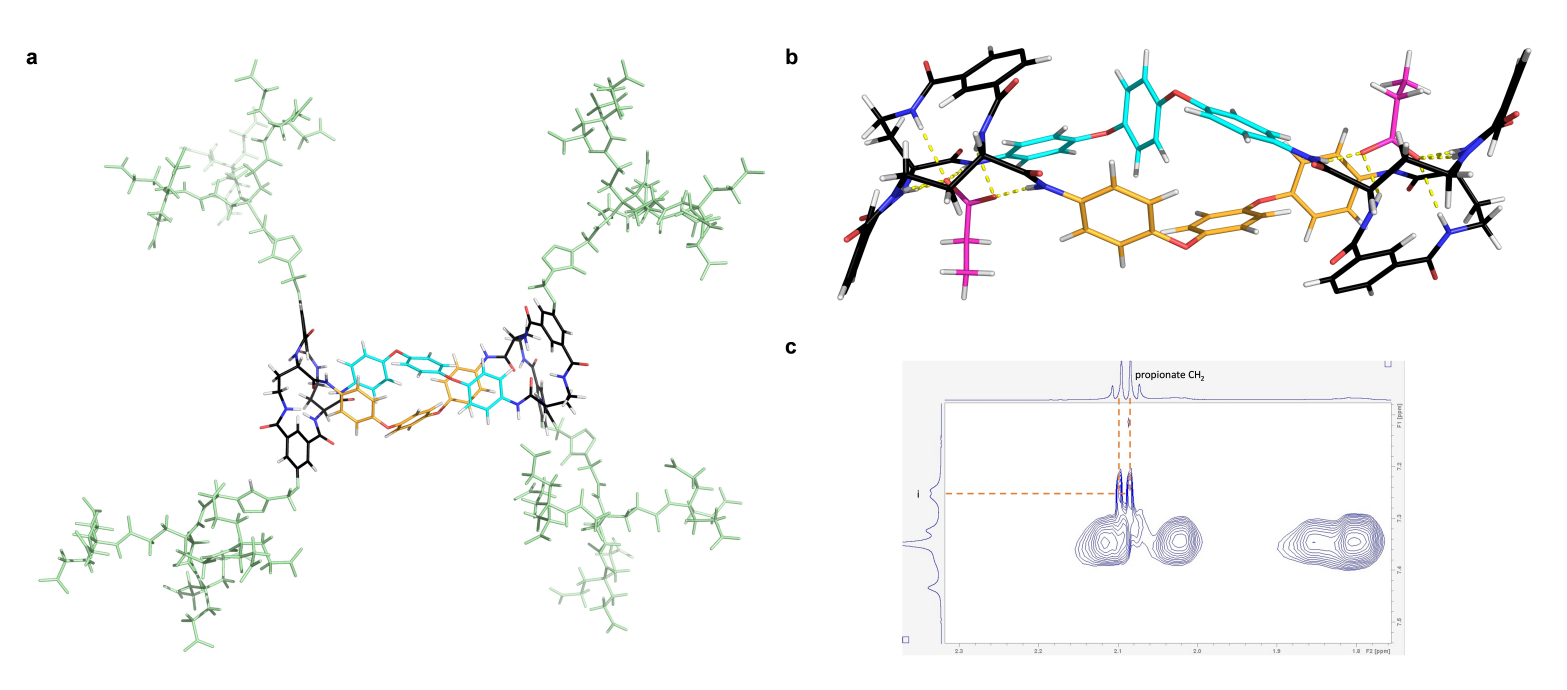
Structural hypotheses for **5** and its complexes with carboxylates. (a) Energy‐minimised structure of receptor **5**, with bis(phenoxy)phenyl bridges (cyan and gold carbons) brought into contact via twisting of one tetralactam ring relative to the other. Solubilising side‐chains are coloured light green. (b) Energy‐minimised structure of receptor **5** binding two propionate anions (magenta carbons). Receptor side‐chains are omitted for clarity. Hydrogen bonds are shown as yellow broken lines. (c) Portion of the 2D NOESY spectrum (9 : 1 H_2_O/D_2_O) of receptor **5** (1.33 mM)+sodium propionate (250 mM), showing cross‐peaks between receptor CH*i* and propionate CH_2_.

To throw light on these results, the structures of the receptors and complexes were investigated by molecular modelling and NMR. The core tetralactam macrocycle **9** (R=H) was subjected to Monte Carlo Molecular Mechanics (MCMM), which yielded a variety of conformations. However MCMM in the presence of formate confirmed that favourable binding geometries are feasible, with all NH groups involved in *H*‐bond donation to the anion (e.g. Figure S91). Dimeric structure **5** is too large for comprehensive studies, but models were elaborated from **9** and minimised with water GB/SA solvation to give an indication of the system's behaviour. Notably, all the resulting conformations featured folded arrangements whereby the tetralactams are twisted relative to each other, bringing the two bis(phenoxy)phenyl bridges together. Figure 5a shows one such example (see also Figure S92). Contact between the bridges should be driven by hydrophobic interactions, so these conformations are not unexpected in water. Support for inter‐bridge contacts could not be obtained through NOE spectroscopy because of the symmetry of the system, but major differences between the spectra of **20** in DMSO and **5** in water suggested a conformational change on moving between solvents. In particular, the signal due to CH*i* shifts upfield by ~0.4 ppm on transferring from DMSO to water, while CH*k* is almost stationary (see Figure S82). This could suggest a folded structure in water promoting inter‐bridge shielding. The solvophobic interactions pulling the bridges together should be far weaker in DMSO than in water, so one might expect a shift from flexible to folded conformation on moving between these solvents.

Rigorous modelling of the interaction of **5** with carboxylates was again unrealistic, but we noted that conformations with multiple NH−O^−^ hydrogen bonds and good interbridge interactions could be generated if the bridges were allowed to slide relative to each other. The conformation in Figure 5b, with propionate guests, is illustrative (see also Figure S93). After energy minimisation, the binding sites at each end of the complex form six hydrogen bonds to carboxylate oxygens, ranging from 1.9 to 2.4 Å. Support for structures of this type was obtained through 2D NOESY investigations of the **5**+propionate complex. Unfortunately the spectra required concentrations of **5** around 1 mM, at which self‐association was expected to be considerable. Accordingly, the spectra were dominated by through‐space contacts between core and side‐chain protons. However, as shown in Figure 5c, a clear and distinctive cross‐peak was observed between the sharp quartet of the propionate and H*i*, the bridge proton nearest to the tetralactam core. Modelling based on the conformation in Figure 5b suggests that these protons can approach within 3 Å of each other in the complex. On the other hand, the model accounts for the promiscuity of receptor **5**. Space is available for a variety of groups R in RCO_2_^−^, while contact between R and receptor is marginal and unlikely to lead to much affinity enhancement.

These structural studies suggest an explanation for the affinity variations between monomer and dimer structures, and between DMSO and aqueous media. Monocyclic structures **9** and **10** are quite flexible and are fairly effective in DMSO (*K*_a_ ~10^3^–10^4^ m ^−1^). Tricyclic organic‐soluble receptors **20** and **21** are still quite flexible and show similar affinities to **8 a** and **9 a** in DMSO, suggesting that the binding sites in monocyclic and tricyclic structures possess similar characteristics in this solvent. In contrast, tricyclic water‐soluble receptors **5** and **6** are firmly held in a closed twisted state by hydrophobic interactions, and this folded structure is well preorganised for binding. Receptors **5** and **6** are thus far more effective in water than flexible monocyclic analogues **8 b** and **9 b**. If this is correct, comparisons can be drawn with natural (protein) receptors which denature in organic solvents but fold into active conformations through hydrophobic interactions in water.

## Conclusions

In conclusion, we have shown for the first time that synthetic receptors with charge‐neutral binding sites can bind carboxylates in water in the absence of organic cosolvent. Selectivity data imply that it is the carboxylate group itself which is complexed, as opposed to some other part of the substrate. Our receptors achieve respectable affinities of ~300 m ^−1^, despite the use of negatively charged solubilising groups with potential for competitive inhibition. The success of these dendrimeric polycarboxylate solubilising groups is perhaps surprising but also encouraging, in that the study of anion recognition in water becomes much easier if solubility issues can be resolved.

The system is not especially selective for carboxylates as against inorganic anions such as sulfate, but this should not detract from the long‐term objective of binding C‐terminal peptides. The architecture of **5** and **6**, featuring macrocyclic binding sites with sp^3^‐hybridised carbon linkage points, is well adapted for elaboration into structures with enclosed, functionalised cavities which can contribute to affinities and control selectivities. The design can therefore serve as a platform for the development of selective receptors for C‐terminal peptides, and perhaps ultimately for alternatives to the glycopeptide antibiotics.

## Conflict of Interests

The authors declare no conflict of interest.

1

## Supporting information

As a service to our authors and readers, this journal provides supporting information supplied by the authors. Such materials are peer reviewed and may be re‐organized for online delivery, but are not copy‐edited or typeset. Technical support issues arising from supporting information (other than missing files) should be addressed to the authors.

Supporting Information

## Data Availability

The data that support the findings of this study are available in the Supporting Information of this article.

## References

[1] L. J. Chen , S. N. Berry , X. Wu , E. N. W. Howe , P. A. Gale , Chem 2020, 6, 61–141.

[2] X. Wu , A. M. Gilchrist , P. A. Gale , Chem 2020, 6, 1296–1309.

[3] L. K. Macreadie , A. M. Gilchrist , D. A. McNaughton , W. G. Ryder , M. Fares , P. A. Gale , Chem 2022, 8, 46–118.

[4] S. Kubik , Chem. Soc. Rev. 2010, 39, 3648–3663. 20617241 10.1039/b926166b

[5] S. Kubik , Acc. Chem. Res. 2017, 50, 2870–2878. 29125287 10.1021/acs.accounts.7b00458

[6] M. J. Langton , C. J. Serpell , P. D. Beer , Angew. Chem. Int. Ed. 2016, 55, 1974–1987. 10.1002/anie.201506589 PMC4755225 26612067

[7] L. Qin , J. R. Wright , J. D. E. Lane , S. N. Berry , R. B. P. Elmes , K. A. Jolliffe , Chem. Commun. 2019, 55, 12312–12315. 10.1039/c9cc06812k 31559993

[8] F. Sommer , S. Kubik , Org. Biomol. Chem. 2014, 12, 8851–8860. 25254969 10.1039/c4ob01497a

[9] J. Bartl , S. Kubik , ChemPlusChem 2020, 85, 963–969. 32406613 10.1002/cplu.202000255

[10] S. N. Berry , L. Qin , W. Lewis , K. A. Jolliffe , Chem. Sci. 2020, 11, 7015–7022. 33250974 10.1039/d0sc02533j PMC7690315

[11] L. M. Hancock , L. C. Gilday , S. Carvalho , P. J. Costa , V. Felix , C. J. Serpell , N. L. Kilah , P. D. Beer , Chem. Eur. J. 2010, 16, 13082–13094. 21031371 10.1002/chem.201002076

[12] J. W. Pflugrath , F. A. Quiocho , Nature 1985, 314, 257–260. 3885043 10.1038/314257a0

[13] Y. Nitanai , T. Kikuchi , K. Kakoi , S. Hanmaki , I. Fujisawa , K. Aoki , J. Mol. Biol. 2009, 385, 1422–1432. 18976660 10.1016/j.jmb.2008.10.026

[14] L. Jing , E. Deplazes , J. K. Clegg , X. Wu , Nat. Chem. 2024, 16, 335–342. 38351381 10.1038/s41557-024-01457-5

[15] A. Borissov , I. Marques , J. Y. C. Lim , V. Felix , M. D. Smith , P. D. Beer , J. Am. Chem. Soc. 2019, 141, 4119–4129. 30730716 10.1021/jacs.9b00148

[16] M. A. Yawer , V. Havel , V. Sindelar , Angew. Chem. Int. Ed. 2015, 54, 276–279. 10.1002/anie.201409895 25385515

[17] T. Lizal , V. Sindelar , Isr. J. Chem. 2018, 58, 326–333.

[18] M. Lisbjerg , B. E. Nielsen , B. O. Milhoj , S. P. A. Sauer , M. Pittelkow , Org. Biomol. Chem. 2014, 13, 369–373. 10.1039/c4ob02211d 25407665

[19] F. Sommer , Y. Marcus , S. Kubik , ACS Omega 2017, 2, 3669–3680. 31457681 10.1021/acsomega.7b00867 PMC6641638

[20] M. He , Y. Yao , Z. Yang , B. Li , J. Wang , Y. Wang , Y. Kong , Z. Zhou , W. Zhao , X.-J. Yang , J. Tang , B. Wu , Angew. Chem. Int. Ed. 2024, 63, e202406946. 10.1002/anie.202406946 38802316

[21] M. G. Cacace , E. M. Landau , J. J. Ramsden , Quart. Rev. Biophys. 1997, 30, 241–277. 10.1017/s0033583597003363 9394422

[22] A. L. Sisson , J. P. Clare , A. P. Davis , Chem. Commun. 2005, 5263–5265. 10.1039/b510768g 16244722

[23] Y. Marcus, in *Ions in Solution and Their Solvation*, John Wiley & Sons, **2015**, p. 107–155.

[24] M. Wenzel , J. Steup , K. Ohto , J. J. Weigand , Chem. Lett. 2022, 51, 20–29.

[25] C. Schmuck , Chem. Commun. 1999, 843–844.

[26] J. Hatai , C. Schmuck , Acc. Chem. Res. 2019, 52, 1709–1720. 31150198 10.1021/acs.accounts.9b00142

[27] M. Giese , J. Niemeyer , J. Voskuhl , ChemPlusChem 2020, 85, 985–997. 32410387 10.1002/cplu.202000142

[28] C. Schmuck , W. Wienand , J. Am. Chem. Soc. 2003, 125, 452–459. 12517158 10.1021/ja028485+

[29] D. H. Williams , B. Bardsley , Angew. Chem. Int. Ed. 1999, 38, 1172–1193. 10.1002/(SICI)1521-3773(19990503)38:9<1172::AID-ANIE1172>3.0.CO;2-C 29711719

[30] Z.-C. Wu , D. L. Boger , Acc. Chem. Res. 2020, 53, 2587–2599. 33138354 10.1021/acs.accounts.0c00569 PMC7674238

[31] B. Hinzen , P. Seiler , F. Diederich , Helv. Chim. Acta 1996, 79, 942–960.

[32] K. B. Jensen , T. M. Braxmeier , M. Demarcus , J. G. Frey , J. D. Kilburn , Chem. Eur. J. 2002, 8, 1300–1309. 11921213 10.1002/1521-3765(20020315)8:6<1300::aid-chem1300>3.0.co;2-w

[33] J. Q. Zhang , J. Kemmink , D. T. S. Rijkers , R. M. J. Liskamp , Chem. Commun. 2013, 49, 4498–4500. 10.1039/c3cc40628h 23571454

[34] A. J. Flint , A. P. Davis , Org. Biomol. Chem. 2022, 20, 7694–7712. 36165239 10.1039/d2ob01381a

[35] C. Schmuck , D. Rupprecht , W. Wienand , Chem. Eur. J. 2006, 12, 9186–9195. 16969772 10.1002/chem.200600573

[36] A. Szumna , J. Jurczak , Eur. J. Org. Chem. 2001, 4031–4039.

[37] M. J. Chmielewski , T. Zielinski , J. Jurczak , Pure Appl. Chem. 2007, 79, 1087–1096.

[38] M. J. Chmielewski , J. Jurczak , Chem. Eur. J. 2005, 11, 6080–6094. 16052653 10.1002/chem.200500232

[39] M. Meldal , C. W. Tornoe , Chem. Rev. 2008, 108, 2952–3015. 18698735 10.1021/cr0783479

[40] V. V. Rostovtsev, L. G. Green, V. V. Fokin, K. B. Sharpless, *Angew. Chem. Int. Ed*. **2002**, *41*, 2596-+. 10.1002/1521-3773(20020715)41:14<2596::AID-ANIE2596>3.0.CO;2-4 12203546

[41] http://supramolecular.org.

[42] D. B. Hibbert , P. Thordarson , Chem. Commun. 2016, 52, 12792–12805. 10.1039/c6cc03888c 27779264

[43] H. Destecroix , C. M. Renney , T. J. Mooibroek , T. S. Carter , P. F. N. Stewart , M. P. Crump , A. P. Davis , Angew. Chem. Int. Ed. 2015, 54, 2057–2061. 10.1002/anie.201409124 PMC4506558 25645064

