# Supplementary material for: Polyanionic Receptors for Carboxylates in Water
Source: Angew Chem Int Ed Engl. 2024 Oct 18;64(1):e202413505. doi: 10.1002/anie.202413505 (PMC11701366; doi:10.1002/anie.202413505)
Supplement: Supplementary file 1 — Supporting Information [file ANIE-64-e202413505-s001.pdf]

Angewandte Chemie
Eine Zeitschrift der Gesellschaft Deutscher Chemiker

Supporting Information

Polyanionic Receptors for Carboxylates in Water

*X. Ren, A. J. Flint, D. Austin, A. P. Davis**

Polyanionic Receptors for Carboxylates in Water

Xudong Ren,[1] Alister J. Flint,[1] Daniel Austin,[1] and Anthony P. Davis[1]

[1]School of Chemistry, University of Bristol, Cantock's Close, Bristol, BS8 1TS, UK.

Table of Contents

1. Synthesis and Characterisation 3

1.1 General 3

1.2 Synthesis of azido solubilising groups (S2 and 22) 4

1.3 Synthesis of activated linker reagents (16a and 16b) 6

1.4 Synthesis of receptors 8a, 8b, 9a, 9b 10a and 10b 9

1.5 Synthesis of receptors 5, 6, 20, 21, 5-G$_1$ and 6-G$_1$ 23

1.6 Characterisation of receptors in their operating environments 34

Receptor **8a** 34

Receptor **8b** 36

Receptor **9a** 38

Receptor **9b** 40

Receptor **10a** 43

Receptor **10b** 45

Receptor **20** 47

Receptor **21** 49

Receptor **5** 51

Receptor **6** 53

1.7 Dilution studies 55

1.8 pH titrations of receptors 5 and 6 61

1.9 Receptors with charge-neutral solubilising groups 63

2. Binding studies 64

2.1 Nuclear Magnetic Resonance (NMR) titrations 64

Receptor **8a** & TBA acetate (DMSO-*d*^{6}) 65

Receptor **8a** & TBA chloride (DMSO-*d*^{6}) 66

Receptor **8b** & chloride (H_2O/D_2O) 67

Receptor **9a** & TBA acetate (DMSO-d^6) 68
Receptor **9a** & TBA chloride (DMSO-d^6) 69
Receptor **9b** & acetate (H_2O/D_2O) 70
Receptor **9b** & chloride (H_2O/D_2O) 71
Receptor **10a** & TBA acetate (DMSO-d^6) 72
Receptor **10a** & TBA chloride (DMSO-d^6) 73
Receptor **10b** & acetate (H_2O/D_2O) 74
Receptor **10b** & chloride (H_2O/D_2O) 75
Receptor **20** & TBA acetate (DMSO-d^6) 76
Receptor **20** & TBA benzoate (DMSO-d^6) 77
Receptor **20** & TBA chloride (DMSO-d^6) 78
Receptor **21** & TBA acetate (DMSO-d^6) 79
Receptor **21** & TBA benzoate (DMSO-d^6) 80
Receptor **21** & TBA chloride (DMSO-d^6) 81
Receptor **5** & formate (D_2O) 82
Receptor **5** & acetate (H_2O/D_2O) 83
Receptor **5** & propionate (D_2O) 84
Receptor **5** & benzoate (H_2O/D_2O) 85
Receptor **5** & *L* and *D*-Lactate (D_2O) 86
Receptor **5** & *O*-Ac-*L*-Lactate (D_2O) 88
Receptor **5** & *N*-Ac-*L* and *D*-Alanine (D_2O) 89
Receptor **5** & chloride (H_2O/D_2O) 91
Receptor **5** & bromide (H_2O/D_2O) 92
Receptor **5** & iodide (H_2O/D_2O) 93
Receptor **5** & nitrate (H_2O/D_2O) 94
Receptor **5** & sulfate (H_2O/D_2O) 95
Receptor **5** & *L/D*-Alanine (D_2O) 96
Receptor **5** & *L*-Ala-*L*-Ala (D_2O) 97
Receptor **6** & acetate (H_2O/D_2O) 98
Receptor **6** & propionate (H_2O/D_2O) 99
Receptor **6** & benzoate (H_2O/D_2O) 100
NMR Structural Studies of Hosts and Complexes 101

2.2 Isothermal Titration Microcalorimetry (ITC) titrations ... 106

Receptor **5** (20 μM) & propionate (250 mM) ... 108

Receptor **5** (250 μM) & propionate (250 mM) ... 109

Receptor **5** (250 μM) & propionate (100 mM) ... 110

Receptor **5** (250 μM) & acetate (250 mM) ... 111

Receptor **5** (250 μM) & chloride (250 mM) ... 112

Receptor **5** (250 μM) & sulfate (250 mM) ... 113

3. Modelling studies ... 114

1. Synthesis and Characterisation

1.1 General

Commercial reagents were purchased from Sigma–Aldrich, Alfa Aesar or Acros Organics and were used without further purification unless otherwise specified. All air and moisture sensitive manipulations were carried out using standard vacuum line and Schlenk techniques. Solvents for air and moisture sensitive manipulations were obtained from an Anhydrous Engineering Solvent Purification System, distilled and dried over activated molecular sieves, or purchased from Acros Organics.

Flash column chromatography was performed on a Biotage® Selekt System using silica (Biotage® Sfär Silica D - 60 µm) or C18 (Biotage® Sfär C18 D - Duo 100 Å 30 µm) columns and a suitable eluent. TLC was performed using aluminium backed TLC plates (Merck-Keiselgel 60 F254) and visualised using UV fluorescence (254 or 365 nm) and/or developed using ninhydrin, potassium permanganate or bromocresol green.

NMR spectra were recorded on Varian VNMR 400 MHz, Bruker 400 MHz, Varian VNMRS 500 MHz, Bruker Advance III HD Cryo 500 MHz, Bruker Neo Cryo 600 MHz and Bruker Cryo 700 MHz spectrometers. All spectra were obtained at 298 K. All ^{1}H and ^{13}C NMR chemical shifts are reported relative to the ^{1}H and ^{13}C chemical shifts of the solvent as standard. LRMS (low resolution mass spectrometry was performed on a Waters 600 Controller with a Waters SQ Detector 2. HRMS (high resolution mass spectrometry) was performed on a Thermo Scientific Orbitrap Elite or a Waters Synapt G2S.

The following starting materials were obtained commercially; diamino acid building blocks Boc-Dap(Z)-OH **(11)**, Boc-Dab(Z)-OH **(12)**, Fmoc-Dab(Boc)-OH **(S12)** and 1,4-Bis(4-aminophenoxy)benzene **(13)**. First and second generation dendritic amine **G_1-NH_2** and **G_2-NH_2** (see Scheme S1) were synthesized following the previously reported procedure.[1] The terms G_1 and G_2 are hereafter applied in building blocks or macrocycles which derived from these components, as in "G_1 azido triacid" **(S2)** and "isophthaloyl G2 tricycle" **(5)**. Receptors with protonated COOH side chains are named with '-H' suffixes, as in "dipicolinoyl G2-acid tricycle" **(6-H)**.

All synthesized molecules were characterized by ^{1}H and ^{13}C NMR spectroscopy in organic solvents to confirm their structure. Macrocycles were further characterized in their operating environments, i.e. DMSO-*d*^{6} for receptors **8a**, **9a**, **10a**, **20**, **21**, and D_2O or 9:1 H_2O/D_2O for receptors **8b**, **9b**, **10b**, **5**, **6**.

1.2 Synthesis of azido solubilising groups (S2 and 22)

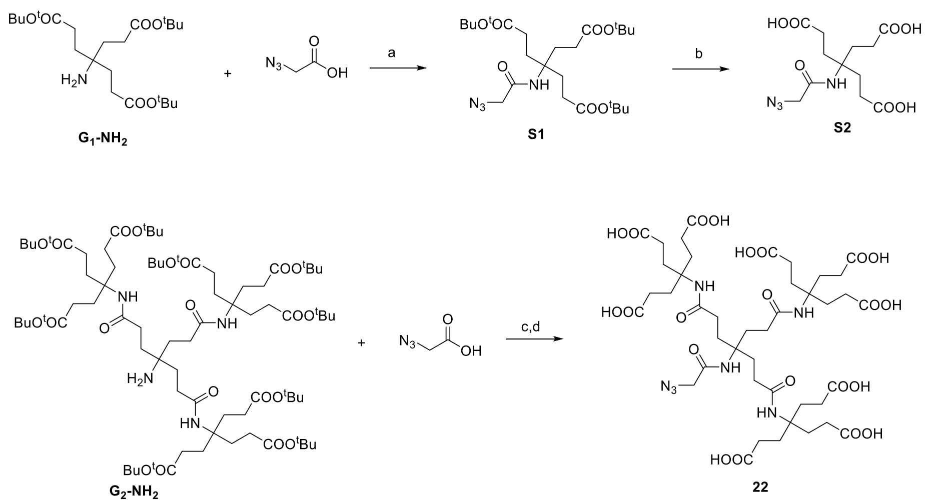

Scheme S1. The synthesis of azido solubilising groups **S2** and **22**. a) DIPEA, HBTU, DMF; b) TFA, DCM; c) DIPEA, HBTU, DMF; d) TFA, DCM.

G_1 azido tri-ester (S1)

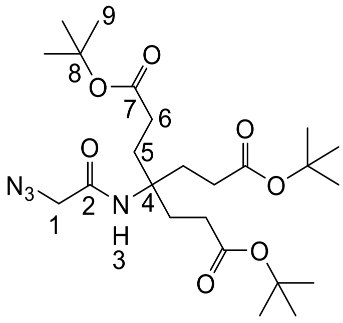

To a solution of the **G_1-NH_2** (400 mg, 0.75 mmol, 1.0 eqv) in THF (20 ml) was added 2-azidoacetic acid (146 mg, 1.44 mmol, 1.5 eqv), HBTU (548 mg, 1.44 mmol, 1.5 eqv) and DIPEA (335 µL, 1.93 mmol, 2.0 eqv). The mixture was allowed to react at room temperature for 12 hours before removing the solvent *in vacuo*. The residue was purified by column chromatography (EtOAc:hexane = 65:35). The solvent was removed *in vacuo* to give the title compound as a white solid (320 mg, 0.63 mmol, 65 %).

^{1}H NMR (500 MHz, $CDCl_3$) δ 6.46 (s, 1H, N3H), 3.86 (s, 2H, C1H), 2.20 (t, *J* = 5.0 Hz, 6H, C6H), 1.98 (t, *J* = 4.9 Hz, 6H, C5H), 1.43 (s, 27H, C9H). **^{13}C NMR** (126 MHz, $CDCl_3$) δ 172.7 (C2), 166.0 (C7), 80.9 (C8), 58.0 (C4), 53.1 (C1), 30.0 (C5), 29.8 (C6), 28.2 (C9). The NMR spectra are in accordance with the literature[2].

G_1 azido tri-acid (S2)

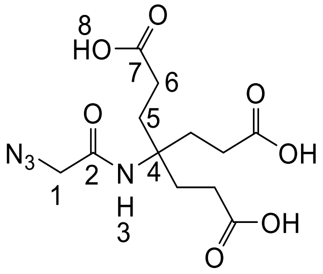

To a solution of the G_1 azido tri-ester **S1** (50 mg, 0.1 mol, 1.0 eqv) in dry DCM (2 ml) solution was added TFA (1 ml), and the mixture was stirred at 0 ℃ for 30 min. The solvent was then removed under N_2 flow to give the title compound as a white solid (33 mg, 99 %).

^{1}H NMR (500 MHz, DMSO-*d_6*) δ 7.44 (s, 1H, N3H), 3.75 (s, 2H, C1H), 2.18 – 2.09 (m, 6H, C6H), 1.89 – 1.79 (m, 6H, C5H). **^{13}C NMR** (126 MHz, DMSO-*d_6*) δ 174.3 (C2), 166.9 (C7), 56.9 (C4), 50.8 (C1), 29.0 (C6), 28.0 (C5). **HRMS** for $C_{12}H_{17}N_4O_7$ $[M-H]^-$ Calculated m/z = 329.1097 Found m/z = 329.1090

G_2 azido nona-acid (22)

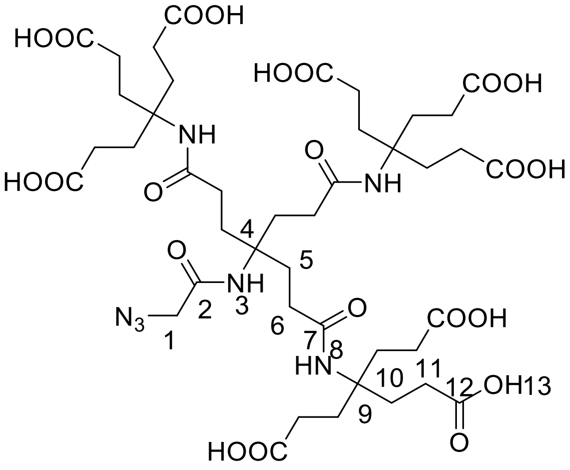

To a solution of **G_2-NH_2** (600 mg, 0.42 mmol, 1.0 eqv) in THF (20 ml) was added 2-azidoacetic acid (63 mg, 0.63 mmol, 1.5 eqv), HBTU (320 mg, 0.63 mmol, 1.5 eqv) and DIPEA (145 µL, 0.83 mmol, 2.0 eqv). The reaction mixture was stirred at room temperature for 12 hours before removing the solvent *in vacuo*. The residue was then purified by column chromatography (EtOAc = 100 %) to give the intermediate nona-ester as a white solid. The solid was stirred for overnight in 25 % TFA DCM solution. Volatiles were then removed under N_2 flow to give the title compound as a white solid (240 mg, 0.24 mmol, 56 %).

^{1}H NMR (500 MHz, DMSO) δ 12.04 (s, 9H, O13H), 7.48 (s, 1H, N3H), 7.20 (s, 3H, N8), 3.74 (s, 2H, C1H), 2.15 – 2.06 (m, 18H, C11H), 2.06 – 1.99 (m, 6H, C6H), 1.88 – 1.72 (m, 24H, C5,10H). **^{13}C NMR** (126 MHz, DMSO) δ 174.9 (C12), 172.5 (C7), 161.4 (C2), 74.7 (C4), 56.8 (C1), 50.9 (C9), 31.2 (C6), 30.5 (C5), 29.5 (C11), 28.5 (C10). **HRMS** for $C_{42}H_{62}N_7O_{22}$ $[M-H]^-$ Calculated m/z = 1016.3948 Found m/z =1016.3965.

1.3 Synthesis of activated linker reagents (16a and 16b)

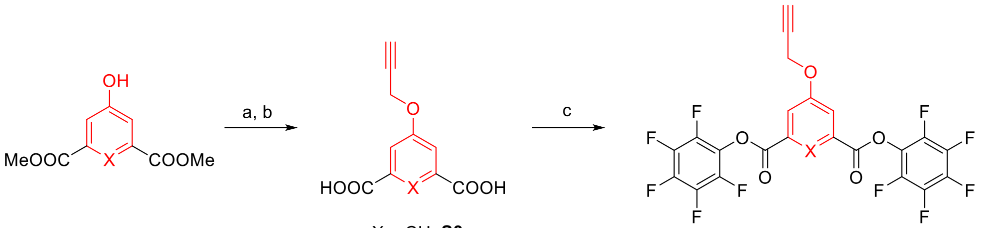

Scheme S2. The synthesis of activated ester linkers. a) Propargyl bromide, K_2CO_3, MeCN; b) NaOH, MeOH; c) DCC, THF.

5-propargyloxy isophthalic acid (S3)

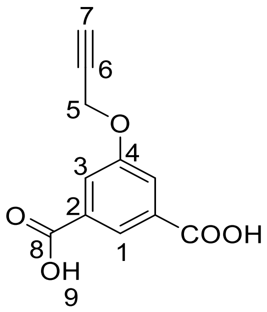

Dimethyl 5-(hydroxy)isophthalate (10 g, 54.9 mmol, 1.0 eqv) and potassium carbonate (15 g, 108 mmol, 2.0 eqv) were suspended in acetonitrile (300 mL). Propargyl bromide (80% in PhMe, 5.40 mL, 56.0 mmol, 1.02 eqv) was then added and the reaction refluxed overnight. The solution was then filtered and concentrated under reduced pressure to give a solid which was then dissolved in methanol (150 mL). Sodium hydroxide (5.00 g, 125 mmol, 2.3 eqv) was added to the solution and the mixture was stirred for 2 hours. The solution was then concentrated under reduced pressure. The residue was dissolved in water (100 mL) then acidified to pH 2-3 with aqueous HCl (1 M) and the resulting precipitate collected by filtration. The solid was washed with water and then dried under reduced pressure to give the pure title compound as a colourless solid (10.7 g, 47.8 mmol, 89 %).

^{1}H NMR (500 MHz, DMSO-d_6) δ 13.34 (s, 2H, O9H), 8.11 (t, J = 1.4 Hz, 1H, C1H), 7.72 (d, J = 1.4 Hz, 2H, C3H), 4.95 (d, J = 2.4 Hz, 2H, C5H), 3.64 (t, J = 2.3 Hz, 1H, C7H). **^{13}C NMR** (126 MHz, DMSO-d_6) δ 166.3 (C8), 157.3 (C4), 132.6 (C2), 122.9 (C1), 119.6 (C3), 79.0 (C6), 78.7 (C7), 56.0 (C5). These data are in accordance with the literature[3].

Bis(pentafluorophenyl) 5-(propargyloxy)isophthalate (16a)

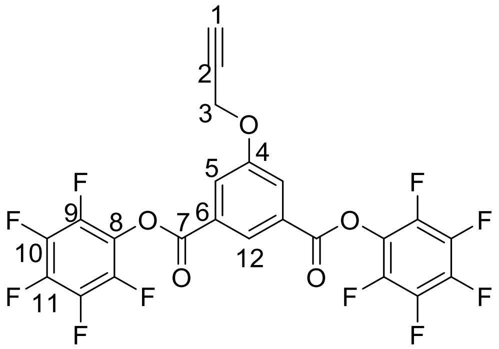

DCC (9.80 g, 47.6 mmol, 2.1 eqv) and pentafluorophenol (10.87 g, 54.8 mmol, 2.4 eqv) were dissolved in anhydrous THF (500 mL) and 5-(propargyloxy)isophthalic acid **S3** (5 g, 22.7 mmol, 1.0 eqv) was added in small portions. The reaction was stirred under an inert atmosphere for 18 hours. The solvent was concentrated under reduced pressure, the resultant solid was suspended in diethyl ether and the solid removed by filtration and washed thoroughly with diethyl ether. The filtrate was concentrated under reduced pressure and the crude residue was recrystalized from hot hexane to give the title compound as a colourless solid (9.10 g, 17.9 mmol, 79%).

^{1}H NMR (400 MHz, $CDCl_3$) δ 8.64 (t, *J* = 1.5 Hz, 1H, C12H), 8.08 (d, *J* = 1.4 Hz, 2H, C5H), 4.86 (d, *J* = 2.36 Hz, 2H, C3H), 2.60 (t, *J* = 2.53 Hz, 4H, C1H). **^{13}C NMR** (101 MHz, $CDCl_3$) δ 161.31 (C7), 158.2 (C4), 142.2 (C8), 140.5 (C9), 139.0 (C11), 137.3 (C10), 128.8 (C6), 125.7 (C12), 123.4 (C5), 79.9 (C2), 78.8 (C1), 56.6 (C3). **HRMS** for $C_{23}H_7F_{10}O_5$ $[M+H]^+$ Calculated m/z = 553.0119 Found m/z = 553.0131.

4-(propargyloxy)pyridine-2,6-dicarboxylic acid (S4)

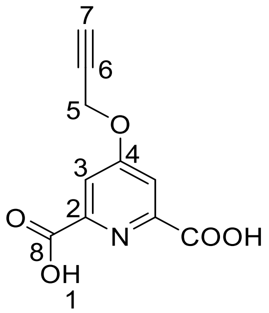

Dimethyl 4-hydroxypyridine-2,6-dicarboxylate (5 g, 27.5 mmol, 1.0 eqv) and potassium carbonate (7.5 g, 54 mmol, 2.0 eqv) were suspended in acetonitrile (100 mL). Propargyl bromide (80% in PhMe, 2.70 mL, 28 mmol, 1.02 eqv) was then added and the reaction refluxed overnight. The solution was then filtered and concentrated under reduced pressure to give a solid which was dissolved in methanol (150 mL). Sodium hydroxide (2.5 g, 62 mmol, 2.3 eqv) was added and the solution stirred for 2 hours. The solution was then concentrated under reduced pressure. The residue was dissolved in water (100 mL) then acidified to pH 2-3 with aqueous HCl (1M) and the resulting precipitate collected by filtration. The solid was washed with water and then dried under reduced pressure to give the title compound as a colourless solid (5.4 g, 23.9 mmol, 87 %).

^{1}**H NMR** (500 MHz, DMSO-d_6) δ 7.78 (s, 2H, C3H), 5.10 (d, *J* = 2.3 Hz, 2H, C5H), 3.76 (t, *J* = 2.3 Hz, 1H, C7H). ^{13}**C NMR** (126 MHz, DMSO-d_6) δ 165.2 (C8), 149.8 (C3), 114.0 (C3), 79.8 (C6), 77.7 (C7), 56.4 (C5). These data are in accordance with the literature[4].

Bis(pentafluorophenyl) 4-(propargyloxy)pyridine-2,6-dicarboxylate (16b)

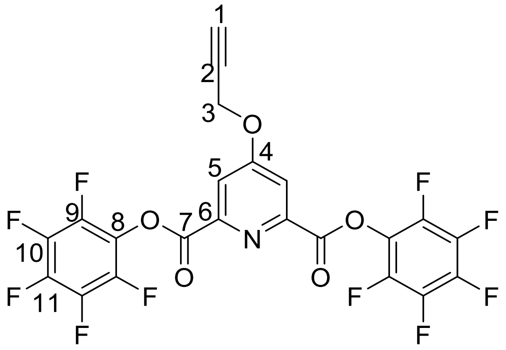

DCC (4.9 g, 23.8 mmol, 2.1 eqv) and pentafluorophenol (5.4 g, 27.4 mmol, 2.4 eqv) were dissolved in anhydrous THF (200 mL) and 4-(propargyloxy)pyridine-2,6-dicarboxylic acid **S4** (2.5 g, 11.4 mmol, 1.0 eqv) was added in small portions. The reaction was stirred under an inert atmosphere for 18 hours. The solution was evaporated under reduced pressure, the resultant solid was suspended in diethyl ether and the solid removed by filtration and washed thoroughly with diethyl ether. The filtrate was concentrated under reduced pressure and the crude residue was recrystalized from hot hexane to give the title compound as a colourless solid (3.8 g, 7.5 mmol, 66 %).

^{1}**H NMR** (500 MHz, $CDCl_3$) δ 8.12 (s, 2H, C5H), 4.96 (d, *J* = 2.4 Hz, 2H, C3H), 2.68 (s, 1H, C1H). ^{13}**C NMR** (126 MHz, $CDCl_3$) δ 166.0 (C7), 160.6 (C4), 147.8 (C6), 117.4 (C5), 78.5 (C2), 75.7 (C1), 57.0 (C3). **HRMS** for $C_{22}H_6F_{10}NO_5$ $[M+H]^+$ Calculated m/z = 554.0081 Found m/z = 554.0077.

1.4 Synthesis of receptors 8a, 8b, 9a, 9b 10a and 10b

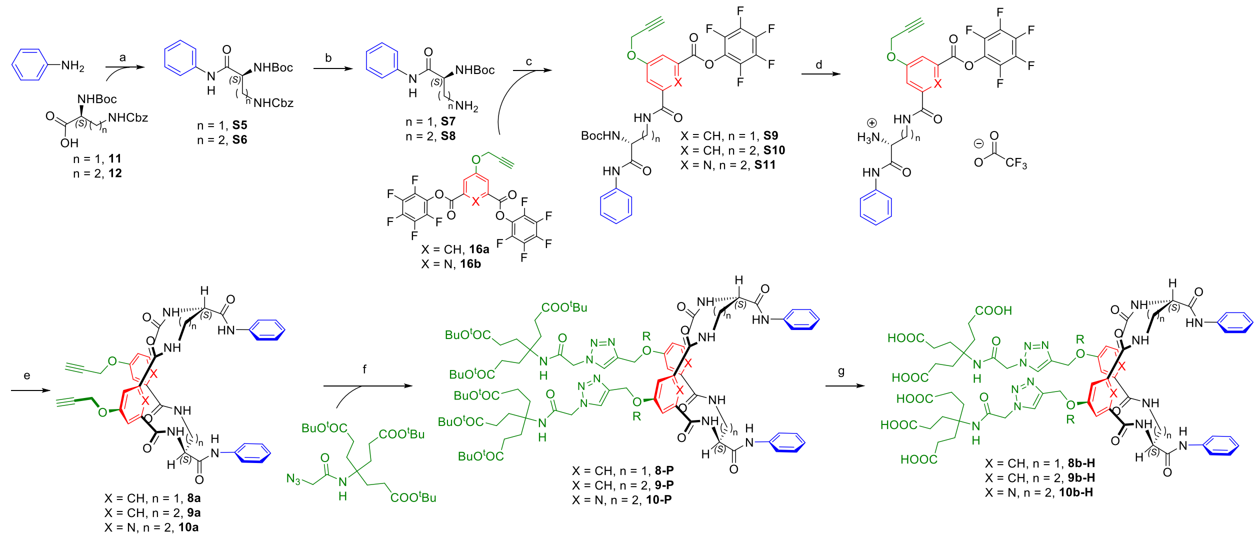

Scheme S3. Synthesis of receptors **8a**, **9a**, **10a**, **8b-H**, **9b-H** and **10b-H**: a) HBTU, TEA, CH_2Cl_2; b) Pd/C, MeOH, H_2; c) DIPEA, THF; d) TFA, CH_2Cl_2; e) DIPEA, TBACl, THF; f) $[Cu(CH_3CN)_4]PF_6$, 2,6-luditine, MeCN: g) TFA, CH_2Cl_2.

Benzyl *tert*-butyl (3-oxo-3-(phenylamino)propane-1,2-diyl)(*S*)-dicarbamate (S5)

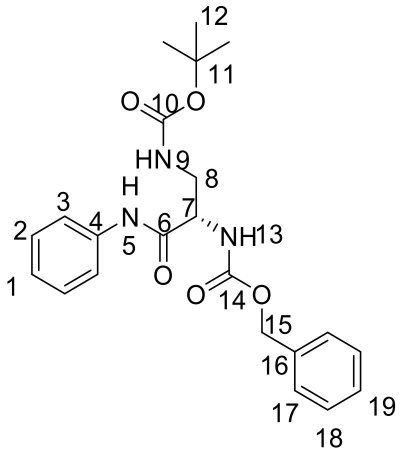

To a solution of commercially available Boc-Dap(Z)-OH **11** (500 mg, 1.48 mmol, 1.0 eqv) in DCM (15 ml) was added TEA (412 µL, 2.96 mmol, 2.0 eqv) and HBTU (672 mg, 1.77 mmol, 1.2 eqv). The mixture was stirred at room temperature for 20 minutes before adding aniline (202 µL, 2.22 mmol, 1.5 eqv). The reaction mixture was stirred at room temperature for overnight, then washed with 0.1 M aqueous HCl (2 x 10 ml), followed by brine (1 x 10 ml) and then dried over $MgSO_4$. The solvent was removed *in vacuo*, and the residue was purified by column chromatography (EtOAc:MeOH = 1:9) to give the title compound (580 mg, 1.4 mmol, 95 %) as a white solid.

^{1}H NMR (500 MHz, DMSO-d_6) δ 9.96 (s, 1H, N5H), 7.61 – 7.55 (m, 2H, C3H), 7.36 – 7.26 (m, 8H, C2,17,18,19,N9H), 7.05 (m, 1H, C1H), 6.89 (d, J = 7.9 Hz, 1H, N13H), 5.05 – 4.95 (m, 2H, C15H), 4.19 (q, J = 6.7 Hz, 1H, C7H), 3.43 – 3.37 (m, 2H, C8H), 1.38 (s, 9H, C12H). **^{13}C NMR** (126 MHz, DMSO-d_6) δ 169.0 (C6),

156.3 (C14), 155.2 (C10), 138.9 (C4), 137.1 (C16), 128.6 (C2), 128.3 (C17), 127.7 (C18), 127.6 (C19), 123.3 (C1), 119.5 (C3), 78.4 (C11), 65.3 (C15), 55.3 (C7), 42.2 (C8), 28.2 (C12). **HRMS** for $C_{22}H_{27}N_3O_5Na$ $[M+Na]^+$ Calculated m/z = 436.1848 Found m/z = 436.1869.

Benzyl *tert*-butyl (4-oxo-4-(phenylamino)butane-1,3-diyl)(*S*)-dicarbamate (S6)

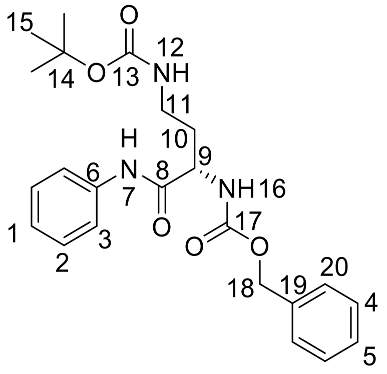

To a solution of commercially available Boc-Dab(Z)-OH **12** (1.0 g, 2.84 mmol, 1.0 eqv) in DCM (15 ml) was added TEA (790 µL, 5.68 mmol, 2.0 eqv) and HBTU (1294 mg, 3.41 mmol, 1.2 eqv). The above mixture was stirred at room temperature for 20 minutes before adding aniline (396 mg, 4.26 mmol, 1.5 eqv). The reaction mixture was stirred at room temperature overnight, then washed with 0.1 M aqueous HCl (2 x 10 ml), followed by brine (1 x 10 ml) and then dried over $MgSO_4$. The solvent was removed *in vacuo*, and the residue was then purified by reverse phase column chromatography (H_2O:acetone = 20:80) to give the title compound (680 mg, 1.59 mmol, 56%) as a white solid.

^{1}H NMR (400 MHz, DMSO-d_6) δ 9.98 (s, 1H, N7H), 7.60 – 7.58 (m, 3H, N16H, C3H), 7.39 – 7.26 (m, 7H, C2,4,5,20H), 7.10 – 7.01 (m, 1H, C1H), 6.74 (t, *J* = 5.1, 1H, N12H), 5.03 (s, 2H, C18H), 4.17 (q, *J* = 7.8 Hz, 1H, C9H), 3.03 – 2.98 (m, 2H, C10H), 1.86 – 1.69 (m, 2H, C10H), 1.37 (s, 9H, C15H). **^{13}C NMR** (101 MHz, DMSO-d_6) δ 170.6 (C8), 156.0 (C13), 155.5 (C17), 138.8 (C6), 136.9 (C19), 128.7 (C4), 128.3 (C20), 127.8 (C5), 127.7 (C2), 123.4 (C1), 119.2 (C3), 77.7 (C14), 65.5 (C18), 53.4 (C9), 37.0 (C10), 32.0 (C11), 28.2 (C15). **HRMS** for $C_{23}H_{29}N_3O_5Na$ $[M+Na]^+$ Calculated m/z = 450.1999 Found m/z = 450.2003.

***Tert*-butyl (*S*)-(2-amino-3-oxo-3-(phenylamino)propyl)carbamate (S7)**

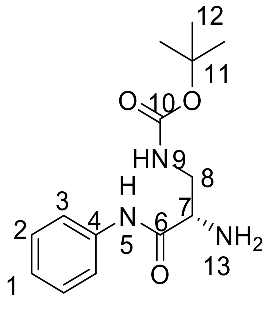

Benzyl *tert*-butyl (3-oxo-3-(phenylamino)propane-1,2-diyl)(*S*)-dicarbamate **S5** (500 mg, 1.21 mmol, 1.0 eqv) and 5 % Pd/C (129 mg, 0.02 mmol, 0.05 eqv) were placed in a three-neck flask, which was then evacuated and backfilled with N_2 3 times. MeOH (20 mL) was added, then the flask was evacuated and backfilled with H_2 3 times. The mixture was allowed to react at room temperature for 12 hours before exchanging H_2 with N_2. The

reaction mixture was filtered under a N_2 atmosphere through celite and the cake was washed with DCM (30 ml). The solvent was removed *in vacuo* to give the title compound (321 mg, 1.15 mmol, 95 %) as a colourless oil.

^{1}H NMR (500 MHz, $CDCl_3$) δ 9.74 (s, 1H, C5H), 7.54 (d, *J* = 7.2 Hz, 2H, C3H), 7.33 – 7.25 (m, 2H, C2H), 7.08 (t, *J* = 7.4 Hz, 1H, C1H), 6.05 (s, 2H, C13H), 4.40 – 4.31 (m, 1H, C7H), 3.33 – 3.25 (m, 1H, C8H), 3.13 – 3.07 (m, 1H, C8H), 1.44 (s, 9H, C12). **^{13}C NMR** (126 MHz, $CDCl_3$) δ 168.8 (C6), 156.7 (C10), 137.8 (C4), 129.1 (C2), 124.6 (C1), 120.2 (C3), 80.9 (C11), 54.6 (C7), 43.8 (C9), 28.4 (C12). **HRMS** for $C_{14}H_{22}N_3O_3$ $[M+H]^+$ Calculated m/z = 280.1661 Found m/z = 280.1665.

Boc-Dap phenylamino PFP benzoate (S9)

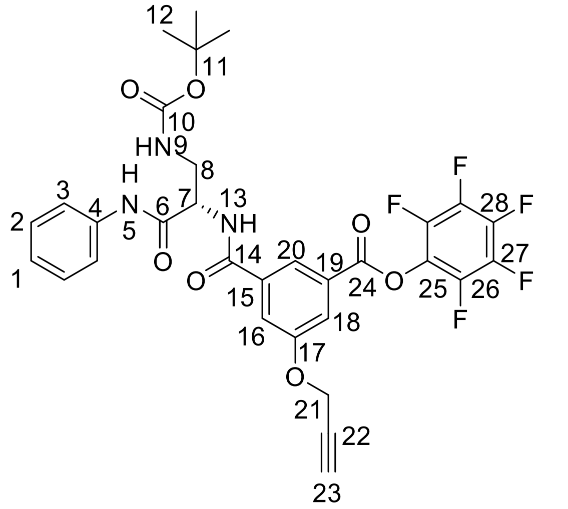

To a solution of bis(pentafluorophenyl) 5-(propargyloxy)isophthalate **16a** (988 mg, 1.79 mmol, 2.5 eqv) in THF (10 ml) was added DIPEA (623 µL, 3.58 mmol, 5.0 eqv). A solution of *tert*-butyl (*S*)-(2-amino-3-oxo-3-(phenylamino)propyl)carbamate **S7** (200 mg, 716 µmol, 1.0 eqv) in THF (10 ml) was added over 18 hours by a syringe pump. The reaction mixture was allowed to stirred at room temperature for 6 hours after the completion of the addition. The solvent was removed *in vacuo*, and the residue was then purified by column chromatography (hexane:EtOAc = 50:50) to give the title compound (301 mg, 0.46 mmol, 65 %) as a white solid.

^{1}H NMR (600 MHz, $CDCl_3$) δ 9.21 (s, 1H, C5H), 8.20 (t, *J* = 1.5 Hz, 1H, C20H), 7.86 – 7.81 (m, 2H, C16H, N9H), 7.76 - 7.70 (m, 1H, C18H), 7.49 – 7.42 (m, 2H, C3H), 7.24 – 7.20 (m, 2H, C2H), 7.09 – 7.03 (m, 1H, C1H), 6.27 (s, 1H, N13H), 4.72 (s, 1H, C21H), 4.59 (s, 1H, C7H) 4.05 – 3.97 (m, 1H, C8H), 3.84 – 3.76 (m, 1H, C8H), 2.53 (t, *J* = 2.4 Hz, 1H, C23H), 1.42 (s, 9H). **^{13}C NMR** (151 MHz, $CDCl_3$) δ 169.1 (C6), 167.4 (C14), 161.7 (C24), 158.1 (C10), 156.9 (C17), 142.1 (25), 140.6 (C26), 139.0 (C27), 137.4 (C4), 137.2 (C28), 136.1 (C15), 129.1 (C2), 128.7 (C19), 124.9 (C1), 122.2 (C20), 120.4 (C3,16), 120.0 (C18), 81.1 (C11), 77.4 (C22), 76.8 (C23), 56.4 (C21), 56.0 (C7), 43.1 (C8), 28.3 (C12). **HRMS** for $C_{31}H_{27}F_5N_3O_7$ $[M+H]^+$ Calculated m/z = 648.1769 Found m/z = 648.1757.

Boc-Dab phenylamino PFP benzoate (S10)

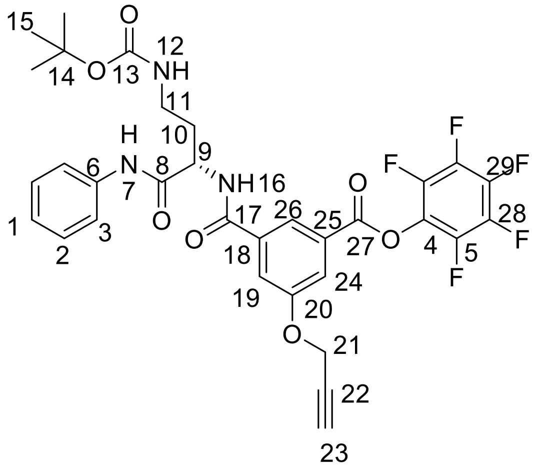

Benzyl *tert*-butyl (4-oxo-4-(phenylamino)butane-1,3-diyl)(*S*)-dicarbamate **S6** (700 mg, 1.17 mmol, 1.0 eqv) and 5 % Pd/C (227 mg, 0.12 mmol mmol, 0.1 eqv) were placed in a three-neck flask, which was then evacuated and backfilled with N_2 3 times. MeOH (20 mL) was added, then the flask was evacuated and backfilled with H_2 3 times. The mixture was allowed to react at room temperature for 12 hours before exchanging H_2 with N_2. The reaction mixture was filtered under a N_2 atmosphere through celite and the cake was washed with DCM (30 ml). The solvent was removed *in vacuo* to give the intermediate compound **S8** as a colourless oil. To a solution of bis(pentafluorophenyl) 5-(propargyloxy)isophthalate **16a** (1.61 g, 2.92 mmol, 2.5 eqv) in THF (10 ml) was added DIPEA (1.02 mL, 5.85 mmol, 5.0 eqv). A solution of the intermediate **S8** in THF (10 ml) was added to the above solution over 18 hours by a syringe pump. The mixture was allowed to react at room temperature for 6 hours after the completion of the addition. The solvent was removed *in vacuo*, and the residue was then purified by column chromatography (hexane:EtOAc = 40:60) to give the title compound (619 mg, 0.94 mmol, 80 %) as a white solid.

^{1}H NMR (600 MHz, $CDCl_3$) δ 10.03 (s, 1H, N7H), 8.30 (s, 1H, C24H), 7.90 (s, 1H, C26H), 7.82 (m, 2H, C19H, N16H), 7.65 (d, *J* = 8.0 Hz, 2H, C3H), 7.33 (t, *J* = 7.8 Hz, 2H, C2H), 7.12 (t, *J* = 7.3 Hz 1H), 4.86 – 4.82 (m, 1H, C9H), 4.81 (d, *J* = 2.4 Hz, 2H, C21H), 3.66 -3.61 (m, 1H, C11H), 3.15 -3.11 (m, 1H, C11H), 2.57 (t, *J* = 2.6 Hz, C23H), 2.19 – 2.13 (m, 1H, C10H), 2.06 – 1.98 (m, 1H, C10H), 1.47 (s, 9H, C15H). **^{13}C NMR** (151 MHz, $CDCl_3$) δ 169.3 (C8), 165.2 (C17), 161.8 (C27), 158.2 (C13,20), 142.3 (C4), 140.6 (C28), 138.9 (C5), 138.0 (C6), 137.3 (C29), 136.3 (C18), 129.1 (C2), 128.8 (C25), 124.6 (C1), 122.1 (C24), 120.3 (C19), 120.2 (C26), 120.0 (C3), 80.8 (C14), 77.4 (C23), 76.8 (C22), 56.5 (C21), 51.7 (C9), 37.2 (C11), 35.1 (C10), 28.5 (C15). **HRMS** for $C_{32}H_{29}F_5N_3O_7$ $[M+H]^+$ Calculated m/z = 662.1920 Found m/z = 662.1914.

Boc-Dab phenylamino PFP picolinate (S11)

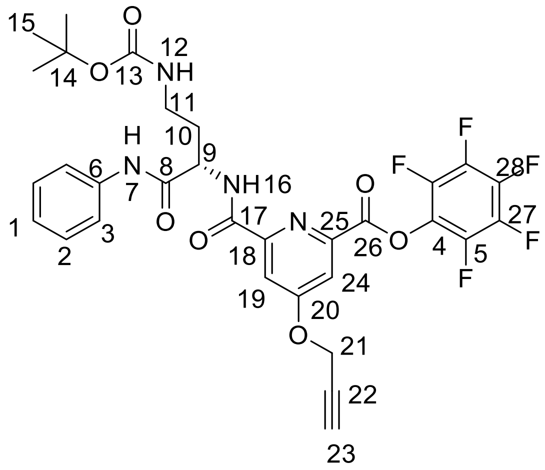

Benzyl *tert*-butyl (4-oxo-4-(phenylamino)butane-1,3-diyl)(*S*)-dicarbamate **S6** (700 mg, 1.17 mmol, 1.0 eqv) and 5 % Pd/C (227 mg, 0.12 mmol mmol, 0.1 eqv) were placed in a three-neck flask, which was then evacuated and backfilled with N_2 3 times. MeOH (20 mL) was added under N_2 atmosphere, then the flask was evacuated and backfilled with H_2 3 times. The mixture was allowed to react at room temperature for 12 hours before exchanging H_2 with N_2. The reaction mixture was filtered under a N_2 atmosphere through celite and the cake was washed with DCM (30 ml). The solvent was removed *in vacuo* to give the intermediate compound **S8** as a colourless oil. To a solution of bis(pentafluorophenyl) pyridine-2,6-dicarboxylate (**16b**) (1.61 g, 2.92 mmol, 2.5 eqv) in THF (10 ml) was added DIPEA (1.02 mL, 5.85 mmol, 5.0 eqv). A solution of the intermediate **S8** in THF (10 ml) was added to the above solution over 18 hours by a syringe pump. The mixture was allowed to react at room temperature for 6 hours after the completion of the addition. The solvent was removed *in vacuo*, and the residue was then purified by column chromatography (hexane:EtOAc = 47:53) to give the title compound (317 mg, 0.48 mmol, 41 %) as a white solid.

^{1}H NMR (500 MHz, $CDCl_3$) δ 9.86 (s, 1H, N7H), 8.89 (s, 1H, N16H), 8.04 (d, *J* = 2.4 Hz, 1H, C19H), 7.95 (d, *J* = 2.5 Hz, 1H, C24H), 7.68 – 7.63 (m, 2H, C3H), 7.30 – 7.33 (m, 2H, C2H), 7.13 – 7.06 (m, 1H, C1H), 5.21 (s, 1H, N12H), 4.88 (d, *J* = 2.4 Hz, 2H, C21H), 3.60 - 3.11 (m, 2H, C11H), 2.62 (t, *J* = 2.4 Hz, 1H, C23H), 2.14 - 2.06 (m, 2H, C10H), 1.45 (s, 9H, C15H). **^{13}C NMR** (126 MHz, $CDCl_3$) δ 168.9 (C8), 166.2 (C17), 163.0 (C26), 160.7 (C13), 157.8 (C20), 152.3 (C18), 145.9 (C25), 142.3 (C4), 140.9 (C27), 139.1 (C5), 138.1 (C6), 137.1 (C28), 129.1 (C2), 124.5 (C1), 120.0 (C3), 116.7 (C24), 112.4 (C19), 80.5 (C14), 77.9 (C23), 76.2 (C22), 56.7 (C21), 51.6 (C9), 37.4 (C11), 35.2 (C10), 28.5 (C15). **HRMS** for $C_{31}H_{28}F_5N_4O_7$ $[M+H]^+$ Calculated m/z = 663.1873 Found m/z = 663.1876.

18-membered isophthaloyl macrocycle (8a)

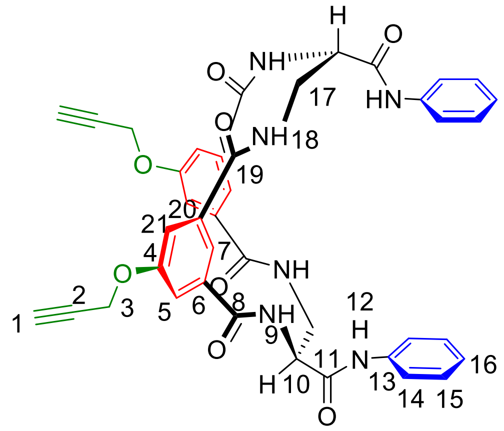

To a solution of Boc-Dap phenylamino PFP benzoate **S9** (100 mg, 0.18 mmol, 1.0 eqv) in DCM (3 ml) was added TFA (1 ml). The reaction was stirred at room temperature for 2 hours before the volatiles were removed by N_2 flow. The residue was dissolved into THF (10 ml) and stored in a syringe for later use. Separately, a solution of DIPEA (317 μL, 1.82 mmol, 10.0 eqv) and tetrabutylammonium chloride (253 mg, 0.91 mmol, 5.0 eqv) in THF (150 ml) was prepared in a round bottomed flask. The solution in the syringe was added slowly to the round bottomed flask at room temperature over 24 hours. The solution was then stirred at room temperature for 4 hours before the solvent was removed *in vacuo*. The residue was then purified by a combination of reverse (H_2O:acetone = 36:64) and normal phase (EtOAc:MeOH = 95:5) column chromatography to give the title compound (39 mg, 54 μmol, 59%) as a white solid.

^{1}H NMR (600 MHz, DMSO-*d*$_6$) δ 10.23 (s, 1H, N12H), 9.00 (s, 1H, N9H), 8.52 (s, 1H, N18H), 8.15 (s, 1H, C7H), 7.66 – 7.61 (m, 2H, C14H), 7.53 (s, 2H, C5,21H), 7.36 – 7.30 (m, 2H, C15H), 7.11 – 7.05 (m, 1H, C16H), 4.91 (d, *J* = 2.2 Hz, 1H, C3H), 4.70 – 4.62 (m, 1H, C10H), 4.16 – 4.06 (m, 1H, C17H), 3.67 – 3.62 (m, 1H, C17H), 3.59 (t, *J* = 2.4 Hz, 1H, C1H). **^{13}C NMR** (151 MHz, MeOD) δ 171.0 (C11), 170.2 (C8), 169.2 (C19), 159.6 (C4), 139.2 (C13), 137.2 (C6), 136.7 (C20), 129.8 (C15), 125.7 (C16), 121.9 (C14), 121.8 (C7), 118.5 (C5), 118.3 (C21), 79.0 (C1), 77.6 (C2), 58.5 (C10), 57.2 (C3), 41.7 (C17). **HRMS** for $C_{40}H_{34}N_6O_8Na$ $[M+Na]^+$ Calculated m/z = 749.2336 Found m/z = 749.2338.

20-membered isophthaloyl macrocycle (9a)

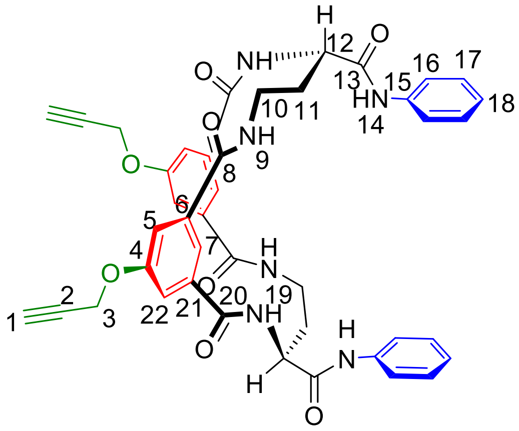

To a solution of Boc-Dab phenylamino PFP benzoate **S10** (100 mg, 0.15 mmol, 1.0 eqv) in DCM (3 ml) was added TFA (1 ml). The reaction was stirred at room temperature for 2 hours before the volatiles were removed by N_2 flow. The residue was dissolved into THF (10 ml) and stored in a syringe for later use. Separately, a solution of DIPEA (262 µL, 1.5 mmol, 10.0 eqv) and tetrabutylammonium chloride (210 mg, 0.76 mmol, 5.0 eqv) in THF (150 ml) was prepared in a round bottomed flask. The solution in the syringe was added slowly to the round bottomed flask at room temperature over 24 hours. The solution was then stirred at room temperature for 4 hours before the solvent was removed *in vacuo*. The residue was then purified by a combination of reverse (H_2O:acetone = 30:70) and normal phase (EtOAc:MeOH = 85:15) column chromatography to give the title compound (25 mg, 34 µmol, 45%) as a white solid.

^{1}H NMR (600 MHz, DMSO-d_6) δ 10.07 (s, 1H, N14H), 8.87 (d, J = 7.1 Hz, 1H, N19H), 8.68 (t, J = 5.8 Hz, 1H, N9H), 8.30 (s, 1H, C7H), 7.62 (d, J = 8.2 Hz, 2H, C16H), 7.55 (d, J = 7.0, 2H, C5,22H), 7.31 (t, J = 7.7 Hz, 2H, C17H), 7.06 (t, J = 7.4, 1H, C18H), 4.86 (d, J = 2.6 Hz, H, C2H), 4.72 – 4.69 (m, 1H, C12H), 3.68 - 3.63 (m, 1H, C10H), 3.59 (t, J = 2.3 Hz, 1H), 3.51 – 3.45 (m, 1H, C10H), 2.32 – 2.12 (m, 2H, C11H). **^{13}C NMR** (151 MHz, DMSO-d_6) δ 170.5 (C13), 165.3 (C20), 165.0 (C8), 156.9 (C4), 139.0 (C15), 135.3 (C6), 135.0 (C21), 128.7 (C17), 123.4 (C18), 119.4 (C7,16), 116.4 (C5), 116.2 (C22), 78.8 (C2), 78.6 (C1), 55.8 (C3), 54.3 (C12), 37.1 (C10), 31.0 (C11). **HRMS** for $C_{42}H_{39}N_6O_8$ $[M+H]^+$ Calculated m/z = 755.2824 Found m/z = 755.2818.

20-membered dipicolinoyl macrocycle (10a)

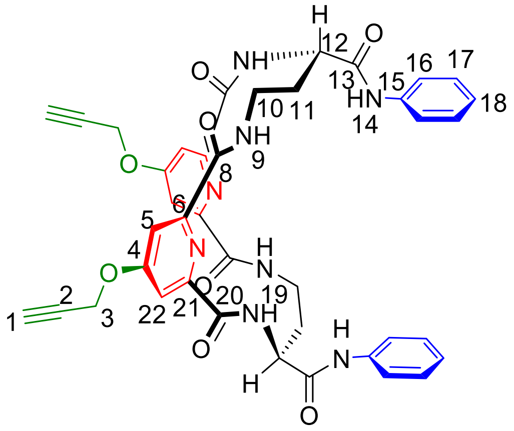

To a solution of Boc-Dab phenylamino PFP picolinate **S11** (100 mg, 0.15 mmol, 1.0 eqv) in DCM (3 ml) was added TFA (1 ml). The reaction was stirred at room temperature for 2 hours before the volatiles were removed by N_2 flow. The residue was dissolved in THF (10 ml) and stored in a syringe for later use. Separately, a solution of, DIPEA (262 µL, 1.5 mmol, 10.0 eqv) in THF (150 ml) was prepared in a round bottomed flask. The solution in the syringe was added slowly to the round bottomed flask at room temperature over 24 hours. The solution was then stirred at room temperature for 4 hours before the solvent was removed *in vacuo*. The residue was then purified by reverse phase column chromatography (H_2O:acetone = 40:60) to give the title compound (39 mg, 51 µmol, 68%) as a white solid.

^{1}H NMR (500 MHz, DMSO-d_6) δ 10.22 (s, 1H, N14H), 9.02 (d, *J* = 9.6 Hz, 1H, N19H), 8.92 (d, *J* = 8.2 Hz, 1H, N9H), 7.70 – 7.55 (m, 2H, C16H), 7.39 (d, *J* = 2.6 Hz, 1H, C5H), 7.34 (d, *J* = 2.6 Hz, 1H, C22H), 7.33 – 7.28 (m, 2H, C17H), 7.09 – 7.02 (m, 1H, C18H), 4.89 (d, *J* = 2.2 Hz, 2H, C3H), 4.87 – 4.81 (m, 1H, C12H), 4.35 - 4.28 (m, 1H, C10H), 4.38 – 3.32 (m, 1H, C10H), 3.67 (t, *J* = 2.4 Hz, 1H), 2.54 – 2.45 (m, 1H, C11H), 2.25 – 2.16 (m, 1H, C11H). **^{13}C NMR** (126 MHz, DMSO-d_6) δ 170.4 (C13), 165.1 (C20), 162.5 (C8), 162.0 (C4), 150.5 (C6), 150.0 (C21), 139.0 (C15), 128.7 (C17), 123.4 (C18), 119.3 (C16), 110.3 (C5), 109.7 (C22), 79.5 (C1), 77.7 (C2), 67.0, 56.1 (C3), 54.5 (C12), 38.2 (C10), 30.7 (C11). **HRMS** for $C_{40}H_{37}N_8O_8$ $[M+H]^+$ Calculated m/z = 757.2729 Found m/z = 757.2730.

18-membered isophthaloyl G_1-ester macrocycle (8-P)

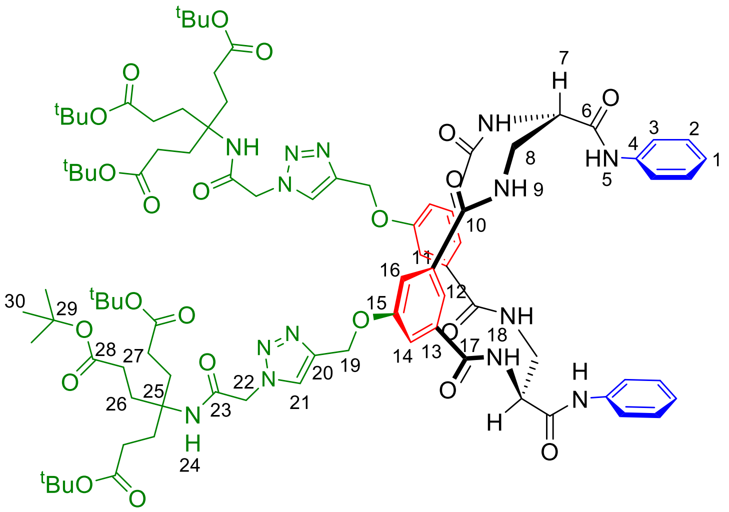

To a solution of macrocycle **8a** (4 mg, 5.5 μmol, 1.0 eqv) in de-gassed MeCN (5 ml) was added G_1 azido tri-ester **S1** (8 mg, 16.5 μmol, 3.0 eqv) and 2,6-lutidine (5 mg, 5.5 μmol, 10.0 eqv). The solution was stirred at room temperature for 15 minutes after which tetrakis(acetonitrile)copper(I) hexafluorophosphate (9 mg, 24 μmol, 5.0 eqv) was added. The reaction mixture was stirred at 60 °C overnight after which the solvent was removed *in vacuo*. The residue was dissolved in DCM (15 ml) and washed with 0.1 M aqueous HCl (2 x 10 ml). The organic phase was then washed with brine, dried over $MgSO_4$ and concentrated *in vacuo*. The residue was purified by reverse phase column chromatography (H_2O:acetone = 11:89) to give the title compound (6.4 mg, 3.7 μmol, 67 %) as a white solid.

^{1}H NMR (600 MHz, MeOD) δ 8.09 (s, 1H, C12H), 8.08 (s, 1H, C20H), 7.63 (s, 1H, C14H), 7.59 (d, *J* = 7.9 Hz, 2H, C3H), 7.55 (s, 1H, C16H), 7.31 (t, *J* = 7.8 Hz, 2H, C2H), 7.11 (t, *J* = 7.4 Hz, 1H, C1H), 5.22 (s, 2H, C19H), 5.11 (s, 2H, C22H), 4.79 – 4.74 (m, 1H, C7H), 4.33 (t, *J* = 12.5 Hz, 1H, C8H), 3.72 (d, *J* = 13.8 Hz, 1H, C8H), 2.24 – 2.18 (m, 6H, C27H), 1.97 – 1.91 (m, 6H, C26H), 1.41 (s, 27H, C30H). **^{13}C NMR** (151 MHz, MeOD) δ 174.3 (C28), 171.3 (C6), 170.2 (C17), 169.1 (C10), 166.8 (C23), 160.3 (C15), 139.3 (C4), 137.3 (C11), 136.7 (C13), 129.9 (C2), 127.4 (C21), 125.7 (C1), 121.9 (C3), 121.4 (C20), 120.9 (C12), 118.5 (C14), 118.0 (C16), 81.8 (C29), 62.9 (C19), 49.6 (C25), 59.4 (C7), 53.4 (C22), 41.7 (C8), 30.6 (C27), 30.4 (C26), 28.4 (C30). **HRMS** for $C_{88}H_{118}N_{14}O_{22}Na_2$ $[M+2Na]^{2+}$ Calculated m/z = 884.4171 Found m/z = 884.4164.

20-membered isophthaloyl G$_1$-ester macrocycle (9-P)

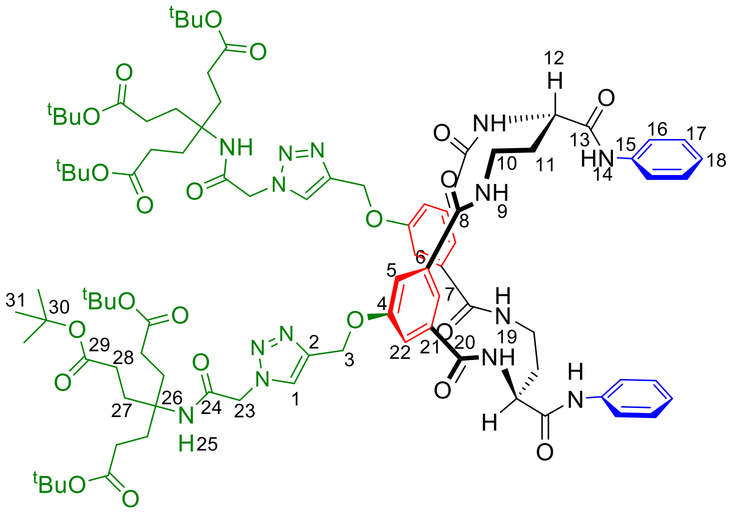

To a solution of 20-membered isophthaloyl macrocycle **9a** (10 mg, 9.7 μmol, 1.0 eqv) in de-gassed MeCN (5 ml) was added G$_1$ azido tri-ester **S1** (15 mg, 29 μmol, 3.0 eqv) and 2,6-lutidine (10 mg, 11 μmol, 10.0 eqv). The solution was stirred at room temperature for 15 minutes after which tetrakis(acetonitrile)copper(**I**) hexafluorophosphate (18 mg, 48 μmol, 5.0 eqv) was added. The reaction mixture was stirred at 60 °C for overnight then the solvent was removed *in vacuo*. The residue was dissolved in DCM (15 ml) and washed with 0.1 M aqueous HCl (2 x 10 ml). The organic phase was then washed with brine, dried over $MgSO_4$ and concentrated *in vacuo*. The residue was purified by reverse phase column chromatography (H_2O:acetone = 10:90) to give the title compound (11 mg, 5 μmol, 53 %) as a white solid.

^{1}H NMR (500 MHz, Methanol-d_4) δ 8.30 (s, 1H, C2H), 8.09 (s, 1H, C7H), 7.68 – 7.42 (m, 4H, C5,22,16H), 7.31 – 7.27 (m, 2H, C17H), 7.09 (d, *J* = 7.6 Hz, 1H, C18H), 5.20 (s, 2H, C3H), 5.14 (s, 2H, C23H), 4.84 – 4.82 (m, 1H, C12H), 3.83 (s, 1H, C10H), 3.42 (s, 1H, C10H), 2.46 – 2.31 (m, 2H, C11H), 2.23 (t, *J* = 8.0 Hz, 6H, C28H), 1.96 (t, *J* = 8.2 Hz, 6H, C27H), 1.42 (s, 27H, C31H). **^{13}C NMR** (126 MHz, Methanol-d_4) δ 174.3 (C29), 172.4 (C13), 168.4 (C24), 166.8 (C8), 163.1 (C20), 159.7 (C4), 144.4 (C1), 139.5 (C15), 136.5 (C6,21), 129.9 (C17), 127.3 (C7), 125.5 (C18), 121.6 (C16), 121.1 (C2), 118.0 (C5), 117.6 (C22), 81.8(C30), 62.9 (C3), 59.4 (C26), 55.8 (C12), 53.4 (C23), 38.3 (C10), 32.5 (C11), 30.6 (C28), 30.5 (C27), 28.4 (C31). **HRMS** for $C_{90}H_{123}N_{14}O_{22}$ $[M+H]^+$ Calculated m/z = 1751.8936 Found m/z = 1751.8984.

20-membered dipicolinoyl G$_1$-ester macrocycle (10-P)

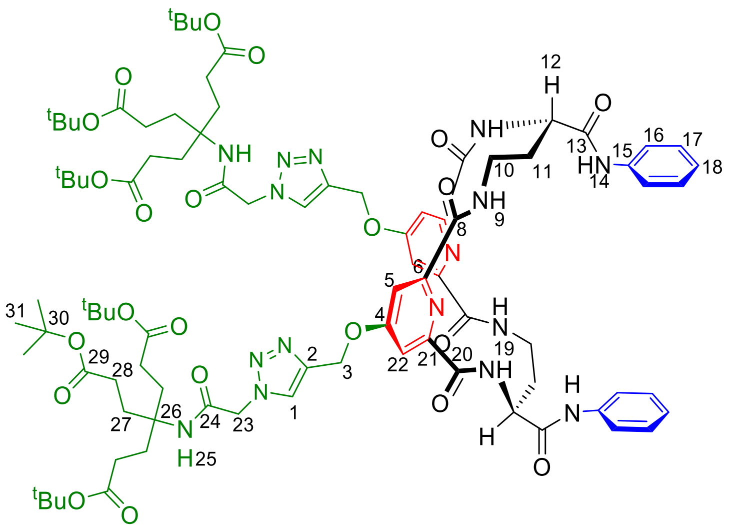

To a solution of macrocycle **10a** (10 mg, 9.7 µmol, 1.0 eqv) in de-gassed MeCN (5 ml) was added G$_1$ azido tri-ester **S1** (15 mg, 29 µmol, 3.0 eqv) and 2,6-lutidine (10 mg, 11 µmol, 10.0 eqv). The solution was stirred at room temperature for 15 minutes after which tetrakis(acetonitrile)copper(I) hexafluorophosphate (18 mg, 48 µmol, 5.0 eqv) was added. The reaction mixture was stirred at 60 °C for overnight before the solvent was removed *in vacuo*. The residue was dissolved in DCM (15 ml) and washed with 0.1 M aqueous HCl (2 x 10 ml). The organic phase was then washed with brine, dried over $MgSO_4$ and concentrated *in vacuo*. The residue was purified by reverse phase column chromatography (H_2O:acetone = 6:94) to give the title compound (10 mg, 4.8 µmol, 48 %) as a white solid.

^{1}**H NMR** (500 MHz, Methanol-*d*$_4$) δ 8.18 (s, 1H, C2H), 7.63 – 7.58 (m, 2H, C16H), 7.57 – 7.50 (m, 2H, C5,22H), 7.36 – 7.30 (m, 2H, C17H), 7.16 – 7.09 (m, 1H, C18H), 5.26 (s, 2H, C3H), 5.16 (s, 2H, C23H), 5.10 – 5.04 (m, 1H, C12H), 4.37 (t, *J* = 12.6 Hz, 1H, C10H), 3.43 – 3.37 (m, 1H, C10H), 2.51 – 2.35 (m, 2H, C11H), 2.29 – 2.20 (m, 6H, C28H), 2.00 – 1.93 (m, 6H, C27H), 1.43 (s, 27H, C31H). ^{13}**C NMR** (126 MHz, Methanol-*d*$_4$) δ 174.4 (C29), 172.2 (C13), 168.1 (C24), 166.8 (C8), 165.2 (C20), 164.7 (C4), 151.8 (C6), 151.4 (C21), 143.1 (C1), 139.4 (C15), 129.9 (C17), 127.8 (C2), 125.6 (C18), 121.5 (C16), 112.1 (C5), 111.9 (C22), 81.7 (C30), 63.1 (C3), 59.4 (C26), 56.1 (C12), 53.5 (C23), 39.4 (C10), 31.8 (C11), 30.6 (C28), 30.5 (C27), 28.4 (C31). **HRMS** for $C_{88}H_{121}N_{16}O_{22}$ $[M+H]^+$ Calculated m/z = 1753.8841 Found m/z = 1753.8885.

18-membered isophthaloyl G$_1$-acid macrocycle (8b-H)

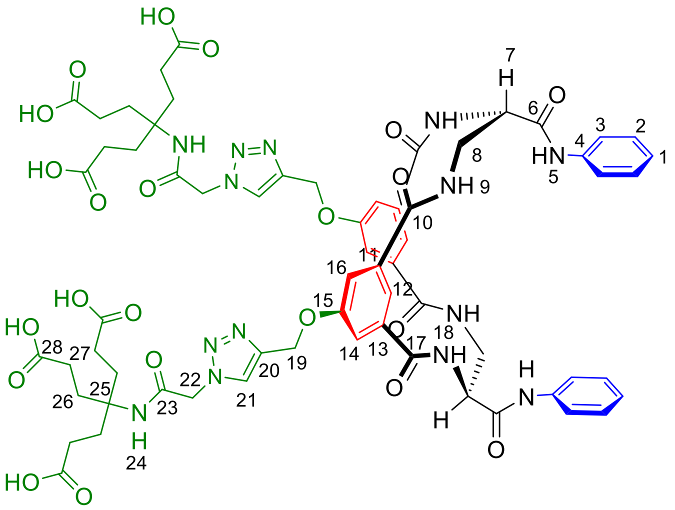

To a solution of macrocycle **8-P** (10 mg, 5.7 µmol) in DCM (3 ml) was added TFA (1 mL). The reaction mixture was stirred at 0 ℃ for 3 hours before the solvent was removed by N_2 flow to give the title compound (6.8 mg, 4.9 µmol, 86 %) as a white solid. The compound was characterized in D_2O after deprotonation.

20-membered isophthaloyl G$_1$-acid macrocycle (9b-H)

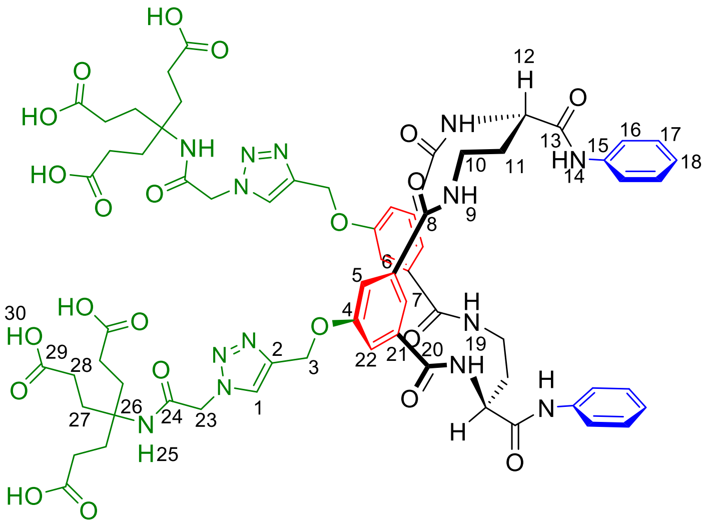

To a solution of macrocycle **9-P** (20 mg, 11.4 µmol) in DCM (3 ml) was added TFA (1 mL). The reaction mixture was stirred at 0 ℃ for 3 hours before the volatiles were removed by N_2 flow to give the title compound (13 mg, 9.4 µmol, 82 %) as a white solid.

^{1}H NMR (600 MHz, DMSO-d_6) δ 12.08 (s, 3H, O30H), 10.07 (s, 1H, N14H), 8.89 (d, J = 7.0 Hz, 1H, N19H), 8.79 (t, J = 5.4 Hz, 1H, N9H), 8.48 (s, 3H, C7H), 8.14 (s, 1H, C2H), 7.78 (s, 1H, N25), 7.66 – 7.57 (m, 4H, C5,16,22H), 7.30 (t, J = 7.8 Hz, 2H, C17H), 7.05 (t, J = 7.4 Hz, 1H, C18H), 5.21 (s, 1H, C23H), 5.09 (s, 1H, C3H), 4.71 – 4.65 (m, 1H, C12H), 3.61 (s, 2H, C10H), 2.42 – 2.39 (m, 1H, C11H), 2.22 – 2.10 (m, 7H, C11, 28H), 1.87 – 1.81 (m, 6H, C27H). **^{13}C NMR** (151 MHz, DMSO-d_6) δ 174.3 (C29), 170.6 (C13), 165.1 (C8), 165.0

(C20), 164.7 (C4), 157.9 (C24), 142.0 (C1), 139.0 (C15), 135.2 (C6), 134.9 (C21), 128.7 (C17), 126.1 (C2), 123.3 (C18), 119.3 (C16), 118.5 (C7), 116.4 (C5), 116.1 (C22), 61.5 (C3), 57.0 (C26), 54.7 (C12), 51.7 (C23), 37.6 (C10), 30.6 (C11), 29.0 (C28), 27.9 (C27). **HRMS** for $C_{66}H_{75}N_{14}O_{22}$ $[M+H]^+$ Calculated m/z = 1415.5180 Found m/z = 1415.5195.

20-membered dipicolinoyl G$_1$-acid macrocycle (10b-H)

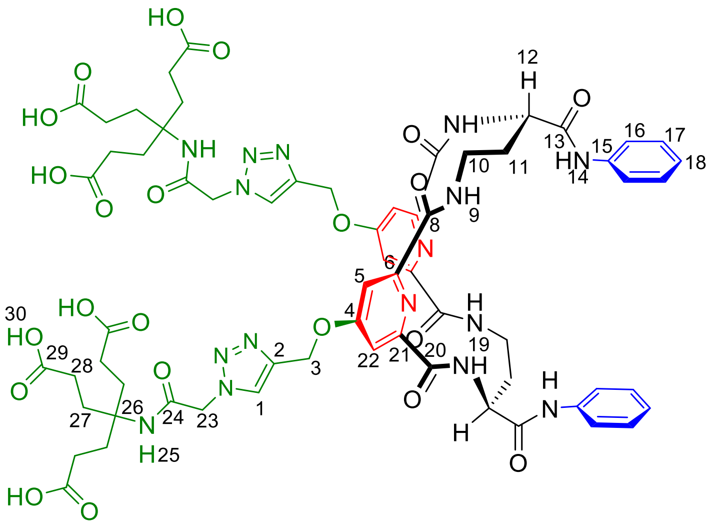

To a solution of 20-membered dipicolinoyl G$_1$-ester macrocycle **10-P** (15 mg, 7.4 μmol) in DCM (3 ml) was added TFA (1 mL). The reaction mixture was stirred at 0 ℃ for 3 hours before the volatiles were removed by N_2 flow to give the title compound (12 mg, 6.9 μmol, 89 %) as a white solid.

^{1}H NMR (500 MHz, DMSO-d_6) δ 12.07 (s, 3H, O30H), 10.24 (s, 1H, N14H), 9.19 (d, J = 9.2 Hz, 1H, N9H), 9.06 (d, J = 8.0 Hz, 1H, N19H), 8.14 (s, 1H, C2H), 7.77 (s, 1H, N25H), 7.64 (d, J = 7.7 Hz, 2H, C16H), 7.49 (d, J = 2.6 Hz, 1H, C5H), 7.43 (d, J = 2.6 Hz, 1H, C22H), 7.34 – 7.28 (m, 2H, C17H), 7.08 – 7.03 (m, 1H, C18H), 5.27 (s, 2H, C23H), 5.08 (s, 2H, C3H), 4.83 (t, J = 9.3 Hz, 1H, C12H), 4.33 – 4.24 (m, 1H, C10H), 2.70 – 2.61 (m, 1H, C11H), 2.21 – 2.11 (m, 7H, C11,28H), 1.87 – 1.80 (m, 6H, C27H). **^{13}C NMR** (126 MHz, DMSO-d_6) δ 174.3 (C29), 170.5 (C13), 165.9 (C4), 164.6 (C24), 162.6 (C8), 162.3 (C20), 150.7 (C6), 150.1 (C21), 141.1 (C1), 139.1 (C15), 128.7 (C17), 126.5 (C2), 123.1 (C18), 119.3 (C16), 109.6 (C5), 110.2 (C22), 61.5 (C23), 57.0 (C26), 54.7 (C12), 51.5 (C3), 37.9 (C10), 30.2 (C11), 29.0 (C28), 27.9 (C27). **HRMS** for $C_{64}H_{73}N_{16}O_{22}$ $[M+H]^+$ Calculated m/z = 1417.5085 Found m/z = 1417.5082.

Water soluble 18/20-membered isophthaloyl/dipicolinoyl G_1 macrocycles (8b, 9b and 10b)

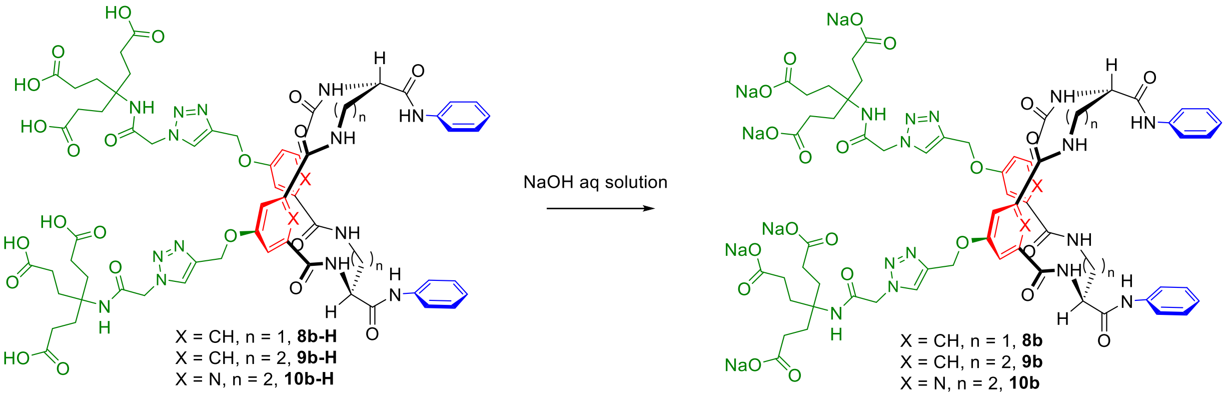

Scheme S4. Synthesis of receptors **8b**, **9b** and **10b**.

To a suspension of 18/20-membered isophthaloyl/dipicolinoyl G_1-acid macrocycles **8b-H**, **9b-H** or **10b-H** in water (10 ml) was added 10 mM NaOH aq solution to adjust the pH to 7.4. The resulting clear colourless solution was freeze-dried to yield the water soluble 18/20-membered isophthaloyl/dipicolinoyl G_1 macrocycles **8b**, **9b** or **10b** (quant yield) in their sodium form as white solids. Samples for characterisation and further studies were prepared by dissolution in D_2O or 9:1 H_2O/D_2O. The process of freeze-drying and dissolution was found to have no effect on the pH.

1.5 Synthesis of receptors 5, 6, 20, 21, 5-G₁ and 6-G₁

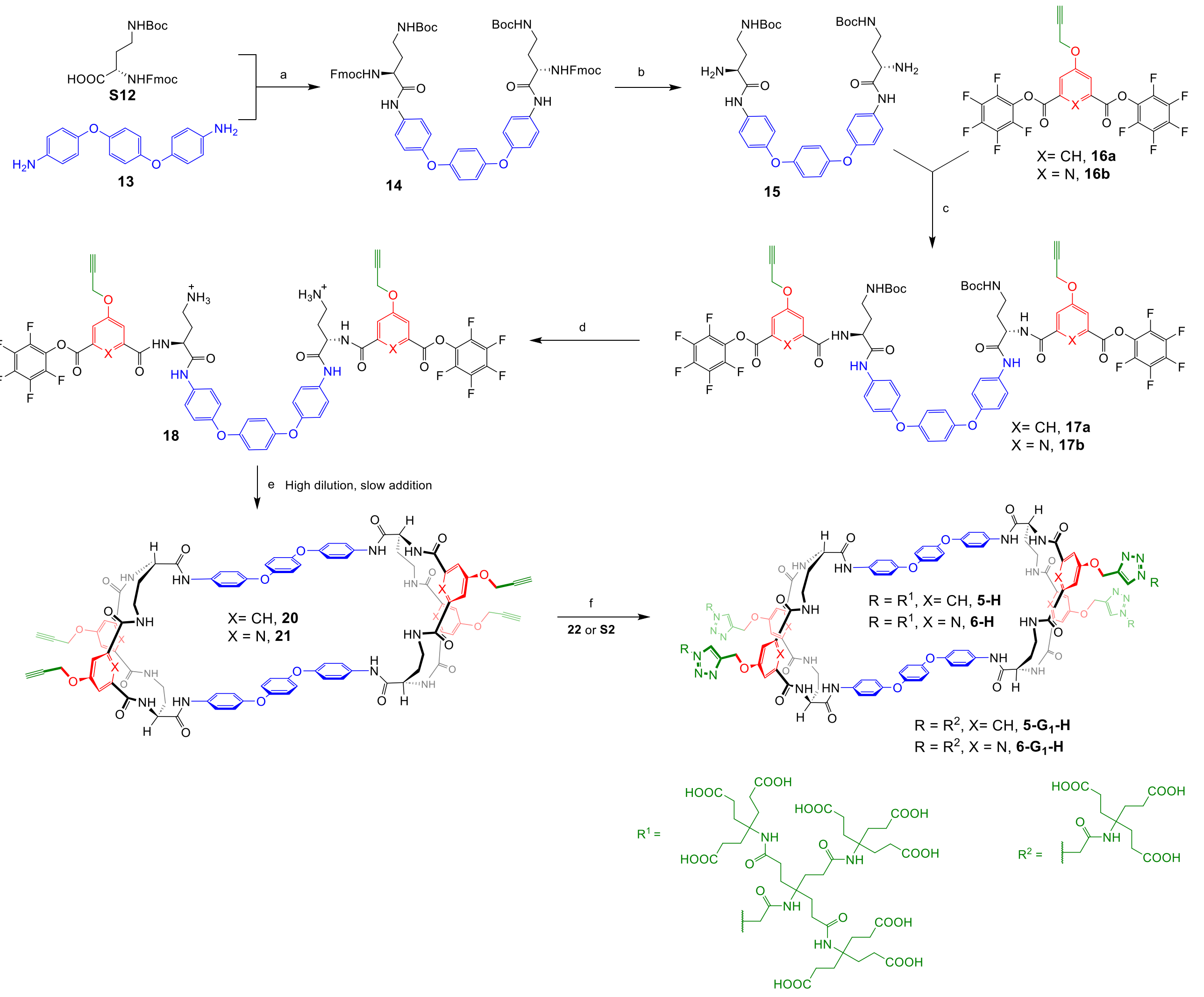

Scheme S5. Synthesis of receptors **5-H**, **6-H**, **20, 21, 5-G_1-H** and **6-G_1-H**. a) HBTU, Na_2CO_3, THF; b) NaOH, MeOH, water; c) DIPEA, THF; d) TFA, DCM; e) DIPEA, THF, (X = N); DIPEA, TBACl, THF, (X = CH); f) $[(CH_3CN)_4Cu]PF_6$, 2,6-lutidine, DMF, MeCN.

Bis(Fmoc-Dab(Boc)-phenoxyphenyl) diamide (14)

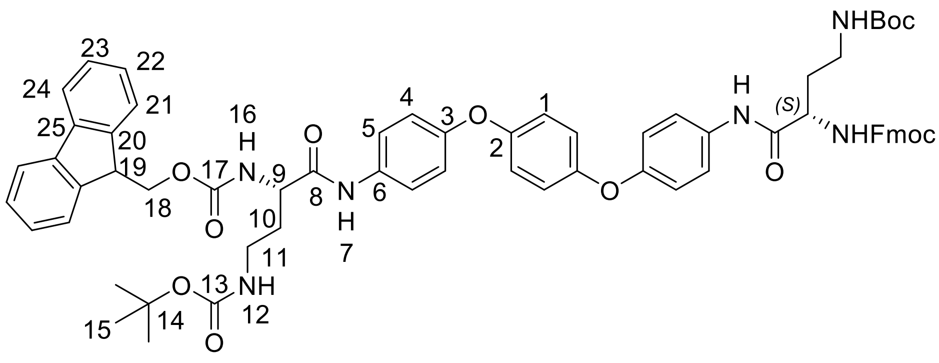

To a solution of commercially available Fmoc-Dab(Boc)-OH **S12** (100 mg, 227 µmol, 2.1 eqv) in dry THF (20 ml) was added HBTU (123 mg, 324 µmol, 3.0 eqv), Na_2CO_3 (57 mg, 540 µmol, 5.0 eqv) and 1,4-Bis(4-aminophenoxy)benzene **13** (32 mg, 108 µmol, 1.0 eqv). The reaction mixture was stirred at 60 °C for 12 hours before the solvent was removed *in vacuo.* The residue was suspended in water (50 ml), then filtered and washed with water (100 ml). The filtration cake was collected and dried in the oven to give the title compound (147 mg, 106 µmol, 98 %) as a white solid.

^{1}H NMR (500 MHz, DMSO-*d*$_6$) δ 10.07 (s, 1H, N7H), 7.89 (d, *J* = 7.6 Hz, 2H, C24H), 7.76 – 7.70 (m, 3H, N16H, C5H), 7.64 – 7.59 (m, 2H, C21H), 7.44 – 7.38 (m, 2H, C23H), 7.36 – 7.29 (m, 2H, C22H), 7.02 – 6.97 (m, 4H, C1, 4H), 6.80 – 6.76 (m, 1H, N12H), 4.32 – 4.20 (m, 3H, C18, 19H), 4.16 (q, *J* = 7.7 Hz, 1H, C9H), 3.02 (q, *J* = 6.7 Hz, 2H, C11H), 1.91 – 1.71 (m, 2H, C10H), 1.37 (s, 9H, C15H). **^{13}C NMR** (126 MHz, DMSO-*d*$_6$) δ 170.5 (C8), 164.6 (C17), 156.0 (C13), 155.5 (C2), 152.5 (C3), 143.8 (C20), 140.7 (C25), 134.5 (C6), 127.6 (C22), 127.1 (C23), 125.4 (C5), 120.9 (C21), 120.1 (C24), 119.7 (C1), 118.9 (C4), 77.7 (C14), 65.7 (C18), 53.3 (C9), 46.6 (C19), 37.1 (C11), 32.0 (C10), 28.2 (C15). **HRMS** for $C_{66}H_{69}N_6O_{12}$ $[M+H]^+$ Calculated m/z = 1137.4960 Found m/z = 1137.4971.

Bis(Boc-Dab-phenoxyphenyl) diamide (15)

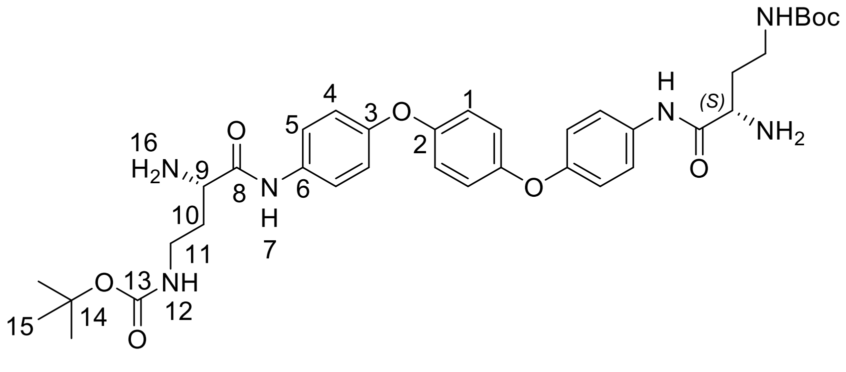

Bis(Fmoc-Dab(Boc)-phenoxyphenyl) diamide **14** (250 mg, 216 µmol, 1.0 eqv) was suspended in MeOH (30 ml) before 1 M NaOH aq solution (10 ml) was then added. The mixture was refluxed for 2 hours then concentrated *in vacuo*. The remaining aqueous phase was then filtered, and the cake was washed with water (3 x 20 ml) and

then hexane (3 x 20 ml). The filtration cake was collected and dried to give the title compound (114 mg, 164 μmol, 76 %) as a colourless gum.

^{1}H NMR (500 MHz, DMSO-*d*$_6$) δ 7.67 – 7.60 (m, 2H, C5H), 7.00 – 6.94 (m, 4H, C1,4H), 6.80 (t, *J* = 5.7 Hz, 1H, N12H), 3.32 – 3.26 (m, 1H, C9H), 3.05 (q, *J* = 6.7 Hz, 2H, C11H), 1.83 – 1.74 (m, 1H, C10H), 1.56 – 1.48 (m, 1H, C10H), 1.37 (s, 9H, C15H). **^{13}C NMR** (126 MHz, DMSO-*d*$_6$) δ 173.6 (C8), 155.6 (C13), 152.6 (C3), 152.4 (C2), 134.6 (C6), 120.8 (C5), 119.7 (C4), 118.8 (C1), 77.5 (C14), 53.5 (C9), 37.2 (C11), 35.0 (C10), 28.3 (C15). **HRMS** for $C_{36}H_{49}N_6O_8$ [M+H]$^+$ Calculated m/z = 693.3612 Found m/z = 693.3600.

Bis(Boc-Dab phenylamino PFP benzoate) (17a)

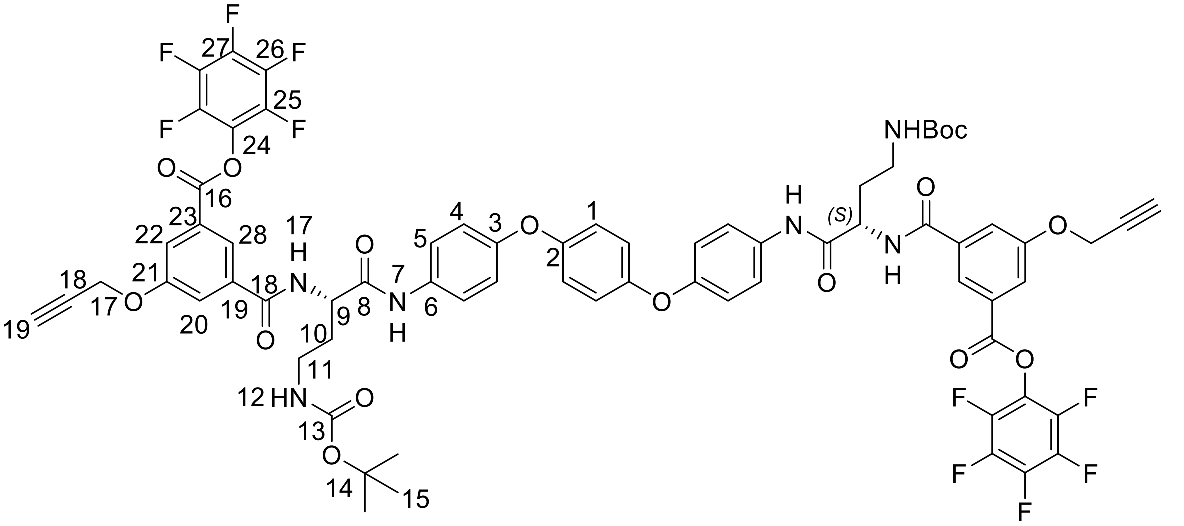

To a solution of bis(pentafluorophenyl)-5-(propargyloxy)isophthalate **16a** (799 mg, 1.4 mmol, 5.0 eqv) in THF (30 ml) was added DIPEA (250 μL, 1.4 mmol, 5.0 eqv). A solution of bis(Boc-Dab-phenoxyphenyl) diamide **15** (200 mg, 289 μmol, 1.0 eqv) in THF (10 ml) was added over 1 hour by a syringe pump. The reaction was stirred at room temperature for 4 hours after the completion of the addition. The solvent was removed *in vacuo*, and the residue was then purified by column chromatography (hexane:EtOAc = 10:90) to give the title compound (373 mg, 261 μmol, 90 %) as a white solid.

^{1}H NMR (500 MHz, CDCl$_3$) δ 10.08 (s, 1H, N7H), 8.28 (s, 1H, C28H), 7.94 – 7.86 (m, 3H, N17, C20H), 7.81 (s, 1H, C22H), 7.59 (d, *J* = 8.6 Hz, 2H, C5H), 6.98 – 6.90 (m, 4H, C1,4H), 5.37 – 5.27 (m, 1H, N12H), 4.89 – 4.82 (m, 1H, C9H), 4.79 (s, 2H, C17H), 3.65 – 3.54 (m, 1H, C11H), 3.20 – 3.11 (m, 1H, C11H), 2.56 (t, *J* = 2.3 Hz, 1H, C19H), 2.19 – 2.00 (m, 2H, C10H), 1.44 (s, 9H, C15H). **^{13}C NMR** (126 MHz, CDCl$_3$) δ 177.9 (C8), 169.3 (C18), 165.3 (C16), 161.7 (C21), 158.1 (C13), 154.2 (C2), 153.0 (C3), 142.3 (C24), 140.3 (C26), 139.1 (C27), 137.0 (C25), 136.2 (C6), 133.4 (C19), 128.7 (C23), 122.1 (C28), 121.6 (C5), 120.4 (C22), 120.1 (C20, C1), 119.1 (C4), 80.7 (C14), 77.2 (C18), 76.8 (C19), 56.5 (C17), 51.7 (C9), 37.2 (C11), 34.9 (C10), 28.5 (C15). **HRMS** for $C_{70}H_{58}F_{10}N_6O_{16}Na$ [M+Na]$^+$ Calculated m/z = 1451.3647 Found m/z = 1451.3622.

Bis(Boc-Dab phenylamino PFP picolinate) (17b)

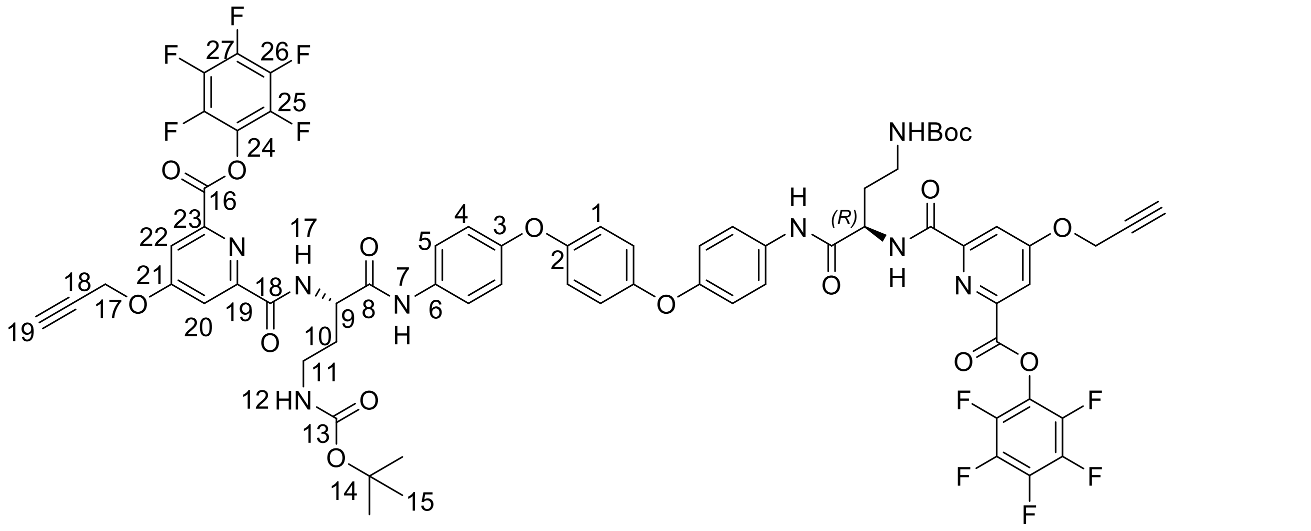

To a solution of bis(pentafluorophenyl) 4-(propargyloxy)pyridine-2,6-dicarboxylate **16b** (799 mg, 1.4 mmol, 5.0 eqv) in THF (30 ml) was added bis(Boc-Dab-phenoxyphenyl) diamide **15** (200 mg, 289 μmol, 1.0 eqv) THF solution (10 ml) over 1 hour by a syringe pump. The reaction was allowed to stirred at room temperature for 1 hour after the completion of the addition. The solvent was removed *in vacuo*, and the residue was then purified by column chromatography (hexane:EtOAc = 15:85) to give the title compound (339 mg, 237 μmol, 82 %) as a white solid.

^{1}H NMR (600 MHz, $CDCl_3$) δ 9.80 (s, 1H, N7H), 8.85 (d, *J* = 7.9 Hz, 1H, N17H), 8.05 (d, *J* = 2.5 Hz, 1H, C20), 7.95 (d, *J* = 2.5 Hz, 1H, C22), 7.65 – 7.60 (m, 2H, C5H), 6.99 – 6.95 (m, 2H, C4H), 6.94 (s, 2H, C1H), 4.90 (d, *J* = 2.4 Hz, 2H, C17H), 4.77 (td, *J* = 9.0, 4.6 Hz, 1H, C9H), 3.61 (ddd, *J* = 14.5, 10.7, 3.4 Hz, 1H, C11H), 3.12 (dt, *J* = 14.9, 4.5 Hz, 1H, C11H), 2.63 (t, *J* = 2.4 Hz, 1H, C19H), 2.16 – 2.10 (m, 1H, C10H), 2.06 – 2.00 (m, 1H, C10H), 1.50 (s, 9H, C15H). **^{13}C NMR** (151 MHz, $CDCl_3$) δ 168.7 (C8), 166.2 (C16), 162.8 (C18), 160.8 (C13), 158.0 (C21), 154.1 (C2), 153.1 (C23), 152.4 (C19), 151.8 (C3), 145.9 (C24), 133.6 (C6), 121.6 (C5), 120.0 (C1), 119.2 (C4), 116.7 (C22), 112.4 (C20), 80.7 (C18), 77.9 (C19), 76.2 (C14), 56.7 (C19), 51.5 (C9), 37.4 (C11), 35.2 (C10), 28.5 (C15). **HRMS** for $C_{68}H_{56}F_{10}N_8O_{16}Na$ $[M+Na]^+$ Calculated m/z = 1453.3552 Found m/z = 1453.3544.

Isophthaloyl tricycle 20

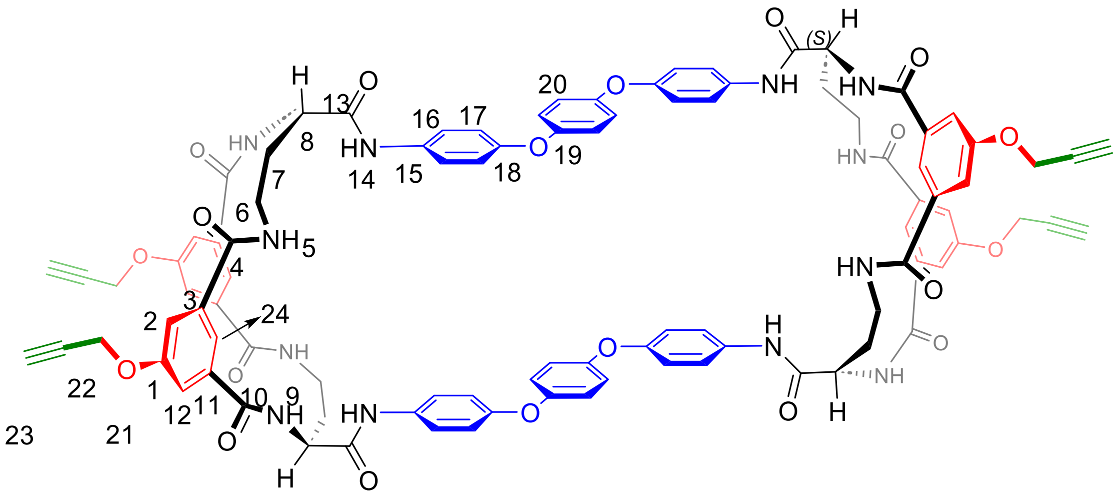

To a solution of bis(Boc-Dab phenylamino PFP benzoate) **17a** (100 mg, 70 μmol, 1.0 eqv) in DCM (3 ml) was added TFA (1 ml). The reaction was stirred at room temperature for 2 hours before the volatiles were removed by N_2 flow. The residue was dissolved in THF (10 ml) and stored in a syringe for later use. Separately, a solution of DIPEA (304 μL, 1.8 mmol, 25.0 eqv) and TBACl (97 mg, 350 μmol, 5.0 eqv) in THF (70 ml) was prepared in a round bottomed flask. The solution in the syringe was added slowly to the round bottomed flask at room temperature over 24 hours. The solution was then stirred at room temperature for 4 hours before the solvent was removed *in vacuo*. The residue was then purified by reverse (H_2O:acetone = 45:56) and normal (DCM:MeOH = 68:32) phase column chromatography to give the title compound (30 mg, 18 μmol, 50%) as a white solid.

^{1}H NMR (500 MHz, DMSO-d_6) δ 10.13 (s, 1H, N14H), 9.30 (d, *J* = 4.5 Hz, 1H, N9H), 8.98 (s, 1H, N5H), 8.67 (s, 1H, C24H), 7.63 – 7.55 (m, 6H, C2,12,16H), 7.03 – 6.94 (m, 4H, C17,20H), 4.84 (d, *J* = 2.3 Hz, 2Hm C21H), 4.61 (s, 1H, C9H), 3.57 (t, *J* = 2.3 Hz, 1H, C23H), 3.51 (s, 2H, C6H), 2.27 (s, 1H, C7H), 2.18 (s, 1H, C7H). **^{13}C NMR** (126 MHz, DMSO-d_6) δ 170.0 (C13), 165.5 (C4), 164.8 (C10), 157.1 (C1), 152.7 (C19), 152.6 (C18), 134.8 (C3,11), 134.6 (C15), 121.2 (C16), 119.9 (C17), 119.5 (C24), 118.8 (C20), 116.6 (C12), 116.5 (C2), 78.8 (C22), 78.7 (C23), 55.8 (C21), 54.5 (C8), 36.8 (C6), 31.6 (C7). **HRMS** for $C_{96}H_{81}N_{12}O_{20}$ [M+H]$^+$ Calculated m/z = 1721.5690 Found m/z = 1721.5685.

Dipicolinoyl tricycle 21

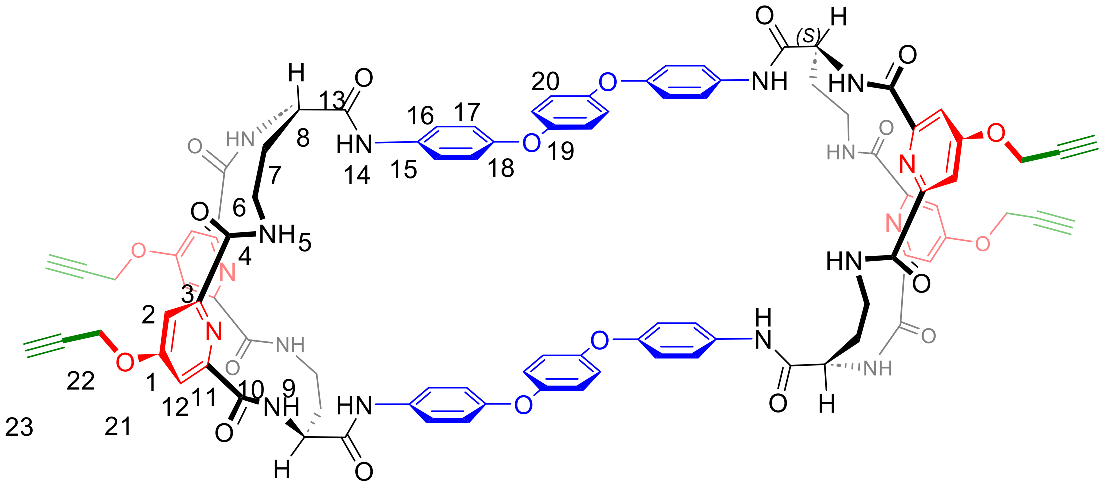

To a solution of bis(Boc-Dab phenylamino PFP picolinate) **17b** (100 mg, 70 µmol, 1.0 eqv) in DCM (3 ml) was added TFA (1 ml). The reaction was stirred at room temperature for 2 hours before the volatiles were removed by N_2 flow. The residue was dissolved into THF (10 ml) and stored in a syringe for later use. Separately, a solution of DIPEA (304 µL, 1.8 mmol, 25.0 eqv) in THF (70 ml) was prepared in a round bottomed flask. The solution in the syringe was added slowly to the round bottomed flask at room temperature over 24 hours. The solution was stirred at room temperature for 4 hours before the solvent was removed *in vacuo*. The residue was then purified by reverse phase column chromatography (H_2O:acetone = 32:68) to give the title compound (36 mg, 21 µmol, 60%) as a white solid.

^{1}H NMR (600 MHz, DMSO-d_6) δ 10.21 (s, 1H, N13), 8.98 – 8.90 (m, 2H, N5,9H), 7.68 – 7.62 (m, 2H, C16H), 7.39 (d, J = 2.6 Hz, 1H, C2H), 7.36 (d, J = 2.6 Hz, 1H, C12H), 7.03 – 7.00 (m, 2H, C17H), 6.91 (s, 2H, C20H), 4.91 – 4.86 (m, 3H, C21,8H), 4.20 (m, 1H, C6H), 3.67 (t, J = 2.3 Hz, 1H, C23H), 3.33 (m, 1H, C6H), 2.46 (m, 1H, C7H), 2.27 (m, 1H, C7H). **^{13}C NMR** (151 MHz, DMSO-d_6) δ 170.6 (C13), 165.6 (C1), 162.8 (C4), 162.6 (C10), 153.5 (C18), 152.6 (C19), 151.0 (C3), 150.5 (C11), 135.4 (C15), 121.5 (C16), 120.2 (C17), 119.4 (C20), 110.5 (C2), 110.3 (C12), 80.0 (C23), 78.1 (C22), 56.6 (C21), 54.8 (C8), 38.5 (C6), 30.5 (C7). **HRMS** for $C_{92}H_{78}N_{16}O_{20}$ $[M+H]^+$ Calculated m/z = 1725.5500 Found m/z = 1725.5520.

Isophthaloyl G_1-acid tricycle 5-G_1-H

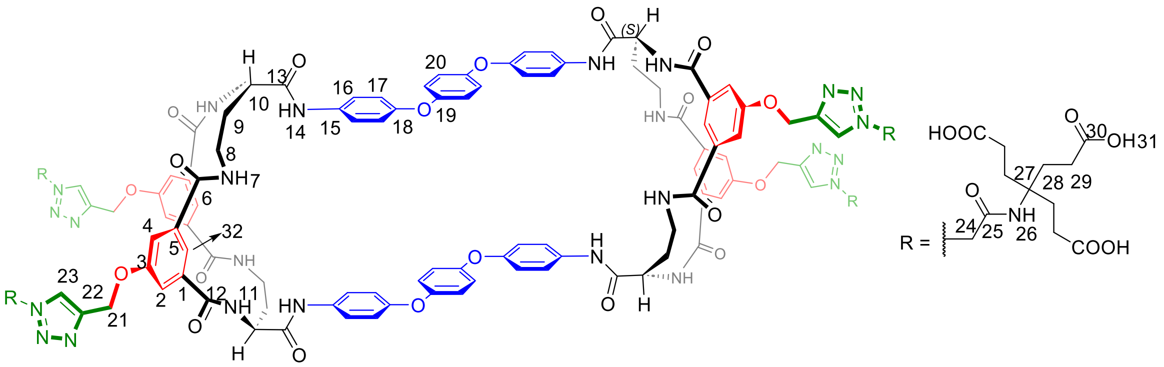

To a solution of tricycle **20** (15.0 mg, 8.7 μmol, 1.0 eqv) in de-gassed MeCN and DMF mixture (2 ml + 2 ml) was added G_1 azido tri-acid **S2** (14.4 mg, 43.6 μmol, 5.0 eqv) and 2,6-lutidine (9.3 mg, 87 μmol, 10.0 eqv). The solution was stirred at room temperature for 15 minutes before tetrakis(acetonitrile)copper(**I**) hexafluorophosphate (16.2 mg, 43.6 μmol, 5.0 eqv) was added. The reaction mixture was stirred at 60 °C for overnight before the solvent was removed *in vacuo*. The residue was then purified by reverse phase column chromatography (0.01 M aqueous HCl:acetone = 20:80) to give the title compound (22.5 mg, 7.4 μmol, 85 %) as a colourless oil.

^{1}H NMR (600 MHz, DMSO-d_6) δ 12.09 (s, 6H, O31H), 10.12 (s, 1H, N14H), 9.11 (s, 1H, N11H), 8.92 (s, 1H, N7H), 8.75 (s, 1H, C32H), 8.12 (s, 1H, C23H), 7.79 (s, 1H, N26H), 7.63 – 7.59 (m, 3H, C17,2H), 7.56 (s, 1H, C4H), 7.01 – 6.97 (m, 4H, C16,20H), 5.17 (s, 2H, C21H), 5.09 (s, 2H, C24H), 4.69 (s, 1H, C10H), 3.68 – 3.65 (m, 1H, C8H), 3.53 – 3.50 (m, 1H, C8H), 2.49 – 2.43 (m, 1H, C9H), 2.21 – 2.14 (m, 7H, C9,29H), 1.88 – 1.81 (m, 6H, C28H). **^{13}C NMR** (151 MHz, DMSO-d_6) δ 174.3 (C30), 170.0 (C13), 166.1 (C25), 164.7 (C3), 158.1 (C6,12), 152.7 (C18,19), 142.0 (C22), 134.6 (C1,5), 134.4 (C15), 126.1 (C23), 121.4 (C17), 119.8 (C16), 118.9 (C20), 118.1 (C32), 116.7 (C2), 116.2 (C4), 61.5 (C21), 57.0 (C10), 51.8 (C24), 40.4 (C27), 34.2 (C8), 30.4 (C9), 29.1 (C29), 28.0 (C28). **HRMS** for $C_{144}H_{149}N_{28}O_{48}$ $[M-3H]^{3-}$ Calculated m/z = 1012.6693 Found m/z = 1012.6707.

Dipicolinoyl G_1-acid tricycle 6-G_1-H

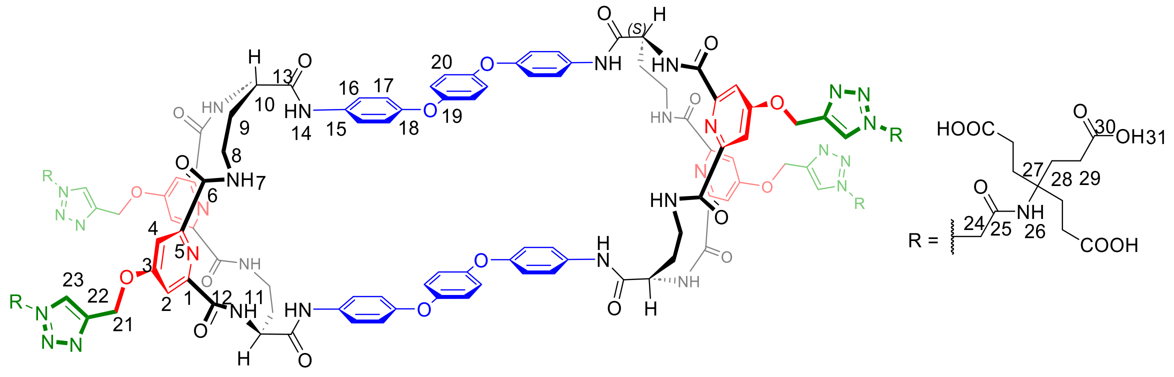

To a solution of tricycle **21** (10.0 mg, 5.8 μmol, 1.0 eqv) in de-gassed MeCN and DMF mixture (2 ml + 2 ml) was added G_1 azido tri-acid **S2** (9.6 mg, 29.0 μmol, 5.0 eqv) and 2,6-lutidine (6.2 mg, 58.1 μmol, 10.0 eqv). The solution was stirred at room temperature for 15 minutes before tetrakis(acetonitrile)copper(**I**) hexafluorophosphate (10.8 mg, 29.0 μmol, 5.0 eqv) was added. The reaction mixture was stirred at 60 °C for overnight before the solvent was removed *in vacuo*. The residue was then purified by reverse phase column chromatography (0.01 M aqueous HCl:acetone = 30:70) to give the title compound (13.3 mg, 4.4 μmol, 75 %) as a colourless oil.

^{1}H NMR (600 MHz, DMSO-*d*$_6$) δ 12.08 (s, 6H, O31H), 10.27 (s, 1H, N14H), 9.43 – 9.27 (m, 2H, N7,11H) 8.15 (s, 1H, C23H), 7.84 (s, 1H, N26H), 7.67 (d, *J* = 8.9 Hz, 2H, C17H), 7.50 (d, *J* = 2.1 Hz, 1H, C2H), 7.46 (d, *J* = 2.2 Hz, 1H, C4H), 7.00 (d, *J* = 8.8 Hz, 2H, C16H), 6.94 (s, 2H, C20H), 5.27 (s, 2H, C21H), 5.09 (s, 2H, C24H), 4.86 (s, 1H, C10H), 4.09 (s, 1H, C8H), 3.50 (s, 1H, C8H), 2.80 (s, 1H, C9H), 2.25 – 2.11 (m, 7H, C9,29H), 1.91 – 1.78 (m, 6H, C28H). **^{13}C NMR** (151 MHz, DMSO-*d*$_6$) δ 175.0 (C30), 170.5 (C13), 166.4 (C6,12), 165.1 (C25), 163.0 (C3), 153.4 (C1), 152.7 (C5), 151.3 (C18), 150.8 (C19), 141.6 (C22), 135.4 (C15), 127.0 (C23), 121.6 (C17), 119.9 (C16), 119.6 (C20), 110.6 (C2,4), 70.2 (C27), 62.1 (C21), 57.5 (C8), 55.2 (C10), 52.3 (C24), 40.9 (C9), 29.6 (C28), 28.6 (C29). **HRMS** $C_{140}H_{146}N_{32}O_{48}$ $[M-2H]^{2-}$ Calculated m/z = 1521.9999 Found m/z = 1521.9989.

Isophthaloyl G$_2$-acid tricycle 5-H

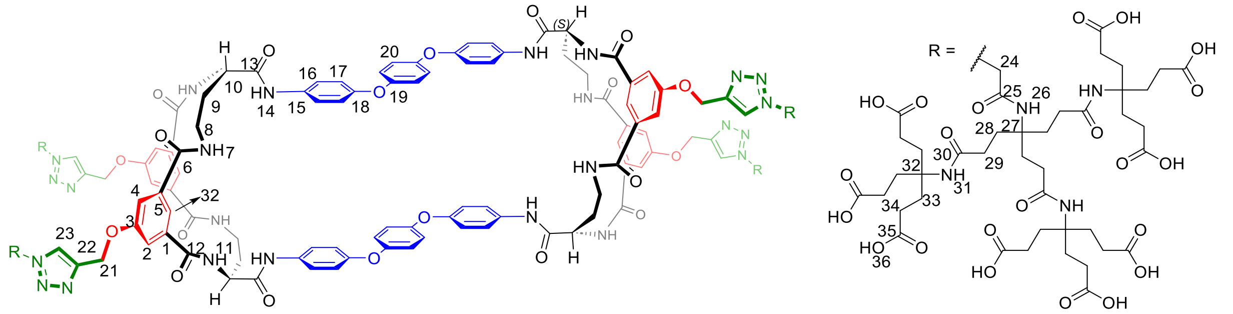

To a solution of isophthaloyl tricycle (15 mg, 8.7 μmol, 1.0 eqv) in de-gassed MeCN and DMF (3 ml + 3 ml) was added G$_2$ azido tri-acid **22** (53 mg, 52 μmol, 6 eqv) and 2,6-lutidine (10 mg, 52 μL, 10 eqv). The solution was stirred at room temperature for 15 minutes before tetrakis(acetonitrile)copper(**I**) hexafluorophosphate (19 mg, 52 μmol, 6 eqv) was added. The reaction mixture was stirred at 60 °C for overnight before the solvent was removed *in vacuo*. The residue was then purified by reverse phase column chromatography (0.01 M aqueous HCl:acetone = 40:60) to the title compound (28 mg, 4.8 μmol, 55 %) as a colourless oil.

^{1}H NMR (500 MHz, DMSO-*d*$_6$) δ 12.00 (s, 9H, O36H), 10.12 (s, 1H, N14H), 9.59 (s, 1H, N7H), 9.15 (s, 1H, N11H), 8.84 (s, 1H, C37H), 8.15 (s, 1H, C23H), 7.80 (s, 1H, N26H), 7.77 (s, 1H, C2H), 7.67 (s, 1H, C4H), 7.65 – 7.53 (m, 2H, C16H), 7.20 (s, 3H, N31H), 7.05 – 6.93 (m, 4H, C17,20H), 5.21 (s, 2H, C21H), 5.10 (s, 2H, C24H), 4.57 (s, 1H, C11H), 2.16 – 2.00 (m, 24H, C29,34H), 1.88 – 1.74 (m, 24H, C28,33H). **^{13}C NMR** (151 MHz, DMSO-*d*$_6$) δ 174.5 (C35), 172.0 (C30), 170.0 (C13), 165.2 (C25), 164.4 (C6,12), 159.9 (C3), 152.6 (C18,19), 142.0 (C22), 134.8 (C1,5,15), 126.1 (C23), 121.4 (C17), 119.9 (C16), 118.8 (C20), 116.5 (C2,4), 111.7 (C37), 61.4(C21), 57.6 (C27), 56.3 (C32), 51.8 (C24), 40.4 (C10), 34.2 (C8), 30.9 (C29), 30.4 (C9), 30.1 (C28), 29.0 (C34), 28.1 (C33). **LRMS** for $C_{264}H_{329}N_{40}O_{108}$ $[M-3H]^{3-}$ Calculated m/z = 1930 Found m/z = 1930.

Dipicolinoyl G$_2$-acid tricycle 6-H

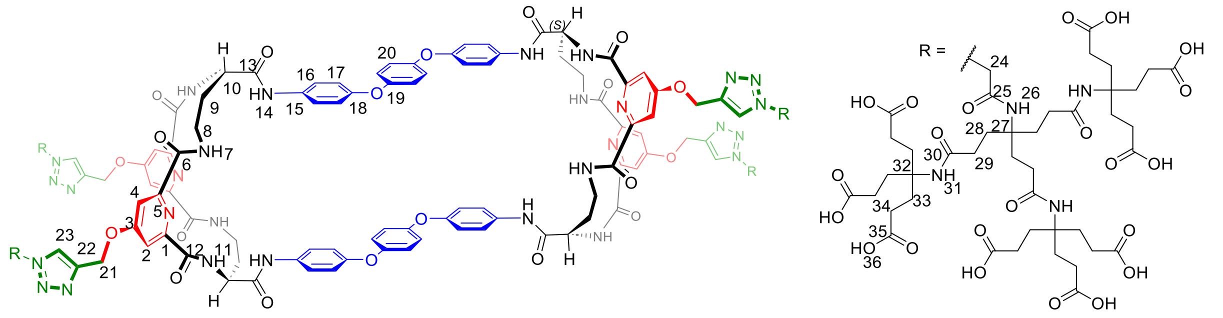

To a solution of dipicolinoyl tricycle **21** (15 mg, 8.7 μmol, 1.0 eqv) in de-gassed MeCN and DMF (3 ml + 3 ml) was added G$_2$ azido tri-acid **22** (53 mg, 52 μmol, 6 eqv) and 2,6-lutidine (10 mg, 52 μL, 10 eqv). The solution was stirred at room temperature for 15 minutes before tetrakis(acetonitrile)copper(**I**) hexafluorophosphate (19 mg, 52 μmol, 6 eqv) was added. The reaction mixture was stirred at 60 °C for overnight before the solvent was removed *in vacuo*. The residue was then purified by reverse phase column chromatography (0.01 M aqueous HCl:acetone = 40:60) to give the title compound (23 mg, 3.9 μmol, 45 %) as a colourless oil.

^{1}H NMR (500 MHz, DMSO-*d*$_6$) δ 10.30 (s, 1H, N14H), 9.53 (s, 2H, N7,11H), 8.15 (s, 1H, C23H), 7.82 (s, 1H, N26H), 7.67 (d, *J* = 8.7 Hz, 2H, C16H), 7.56 (d, *J* = 10.7 Hz, 2H, C2,4H), 7.24 (s, 3H, N31H), 7.00 (d, *J* = 8.6 Hz, 2H, C17H), 6.95 (s, 2H, C20H), 5.31 (s, 2H, C21H), 5.10 (s, 2H, C24H), 4.85 (s, 1H, N10H), 4.07 – 3.98 (m, 1H, C8H), 2.93 – 2.86 (m, 1H, C8H), 2.17 – 2.02 (m, 26H, C9,28,33H), 1.87 – 1.73 (m, 24H, C29,34H). **^{13}C NMR** (126 MHz, DMSO-*d*$_6$) δ 174.9 (C35), 172.5 (C30), 170.5 (C13), 166.4 (C25), 164.9 (C3), 163.2 (C6), 163.0 (C12), 153.3 (C18), 152.7 (C19), 151.3 (C5), 150.8 (C1), 141.7 (C22), 135.3 (C15), 126.9 (C23), 121.7 (C16), 119.8 (C17), 119.7 (C20), 110.8 (C2,4), 62.0 (C21), 58.0 (C27), 56.8 (C32), 55.5 (C10), 52.3 (C24), 36.3 (C8), 33.8 (C9), 31.4 (C29), 30.6 (C28), 29.5 (C34), 28.5 (C33). **HRMS** $C_{260}H_{324}N_{44}O_{108}$ $[M-4H]^{4-}$ Calculated m/z = 1448.2826 Found m/z = 1448.2838.

Water soluble isophthaloyl/dipicolinoyl G_1/G_2 tricycles 5, 6, 5-G_1 and 6-G_1

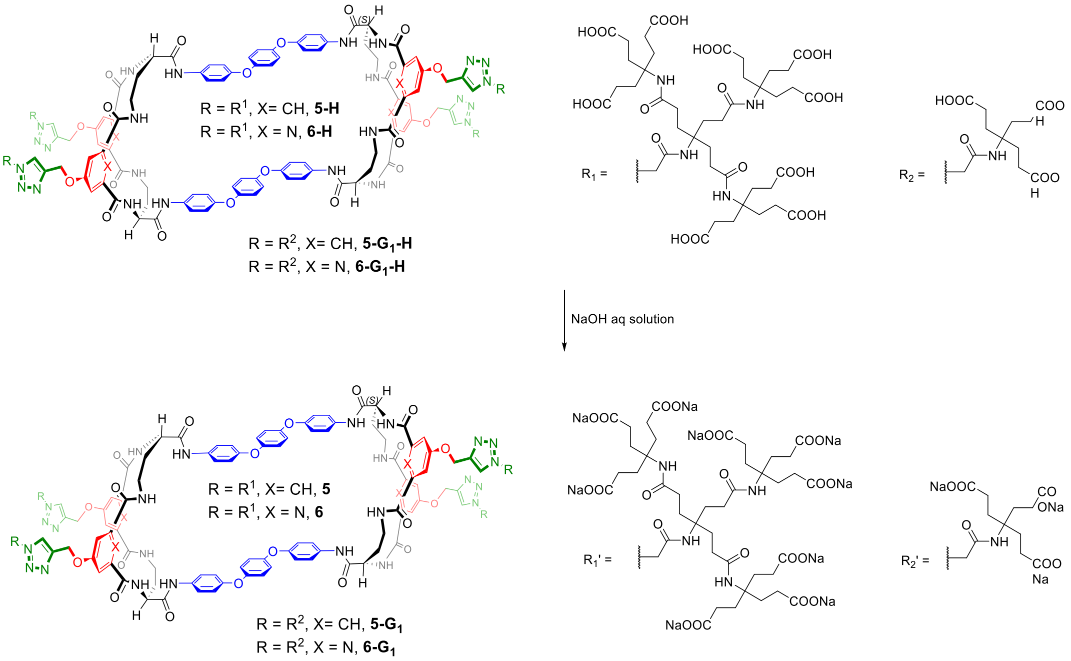

Scheme S6. Synthesis of receptors **5**, **6**, **5-G_1** and **6-G_1**.

To a suspension of isophthaloyl/dipicolinoyl G_1/G_2 acid tricycle in water (10 ml) was added 10 mM NaOH aq solution to adjust the pH to ~7.4. The resulting solution was freeze-dried to yield the water soluble isophthaloyl/dipicolinoyl G_1/G_2 tricycle (quantitative yield) in their sodium form as white solids. Samples for characterisation and further studies were prepared by dissolution in D_2O or 9:1 H_2O/D_2O. The process of freeze-drying and dissolution was found to have no effect on the pH.

1.6 Characterisation of receptors in their operating environments

Receptor 8a

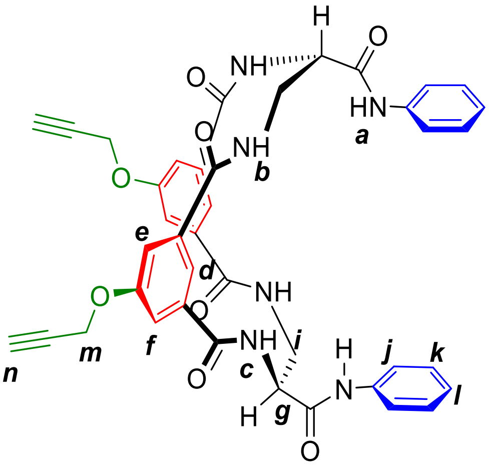

The assignment of the NMR spectrum in DMSO-d^6 was made with the help of 2D COSY and HSQC (see Figures below).

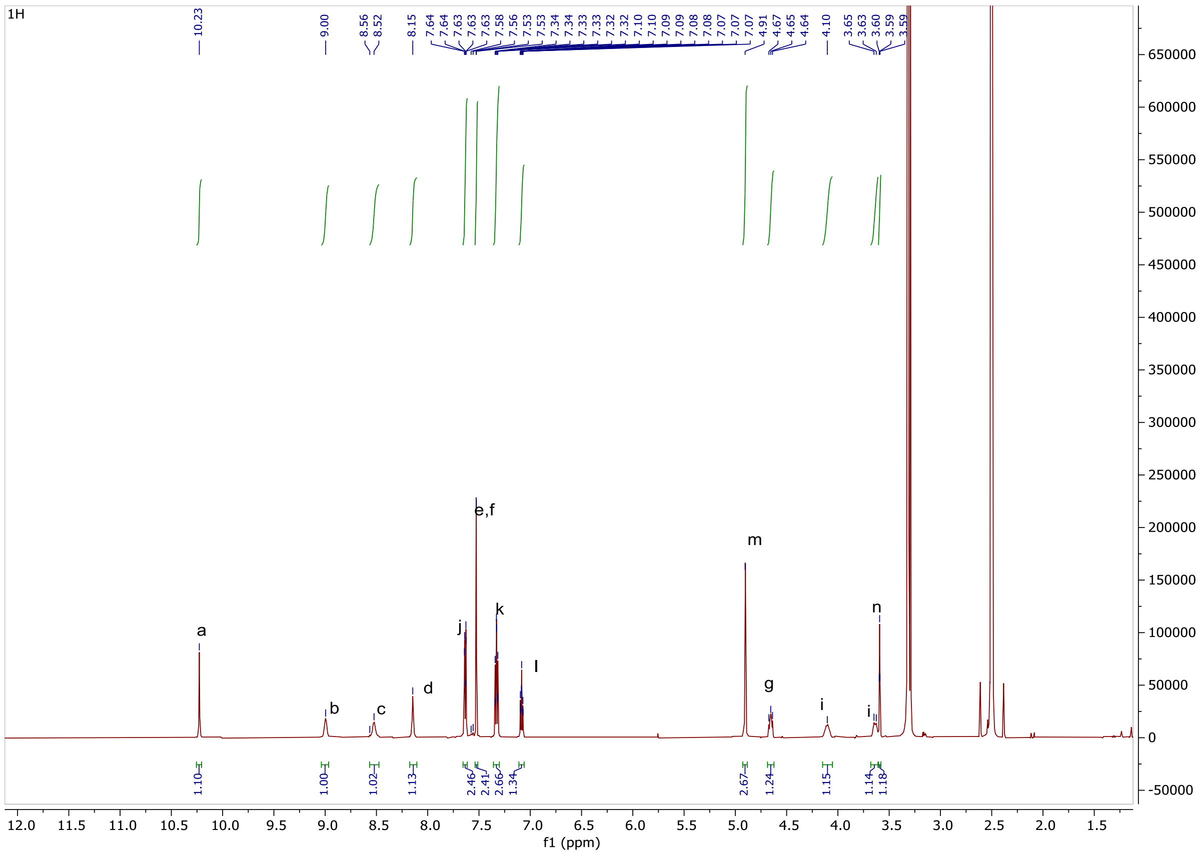

Figure S1. ^{1}H NMR spectrum (600 MHz) of receptor **8a** in DMSO-d^6.

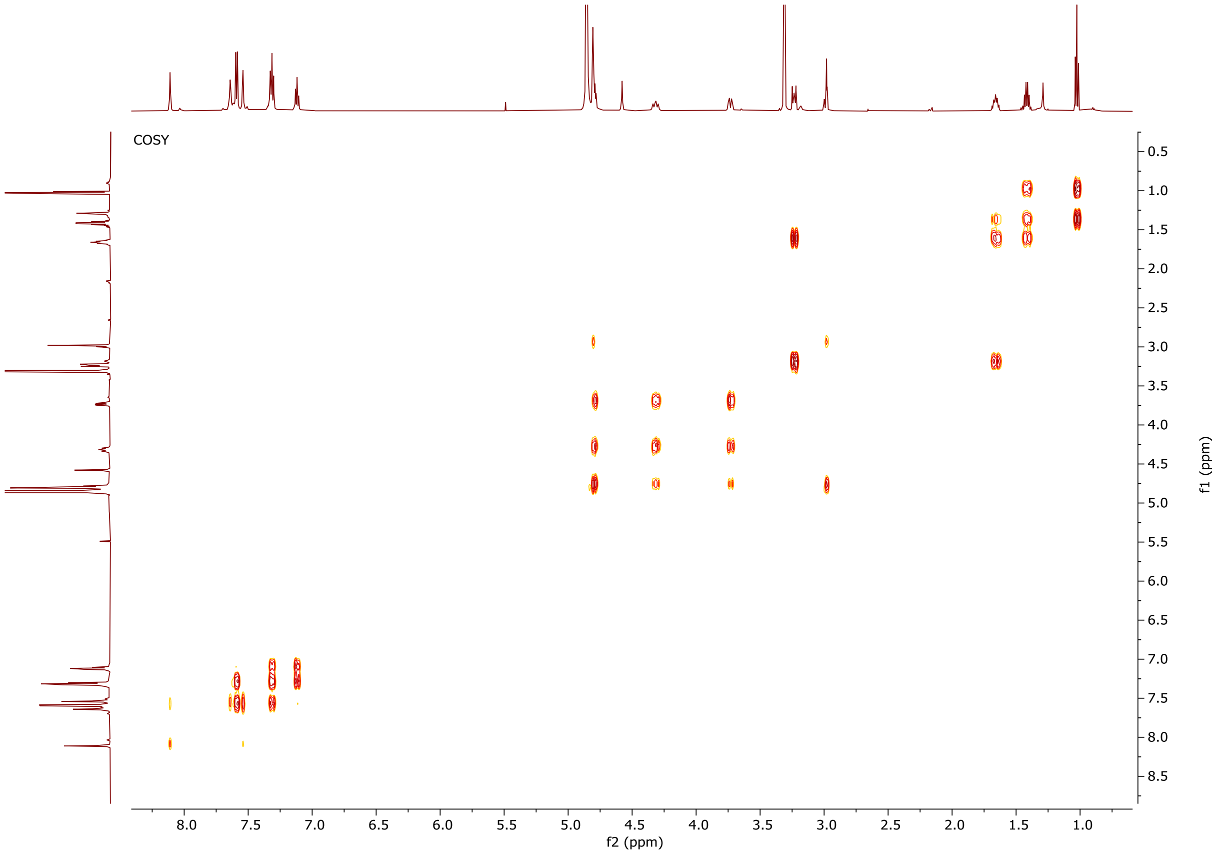

Figure S2. 2D COSY NMR spectrum (600 MHz) of receptor **8a** in DMSO-*d*^{6}.

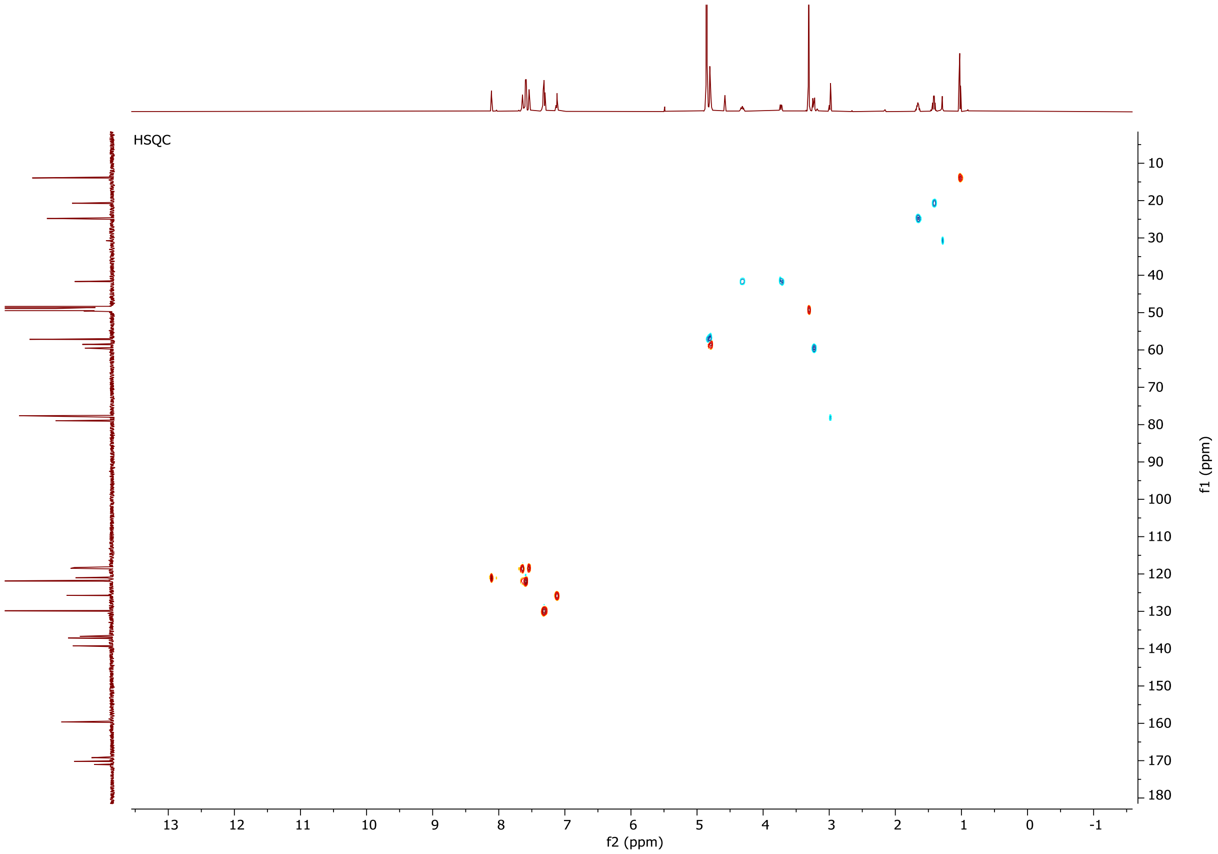

Figure S3. 2D HSQC NMR spectrum (600 MHz) of receptor **8a** in DMSO-*d*^{6}.

Receptor 8b

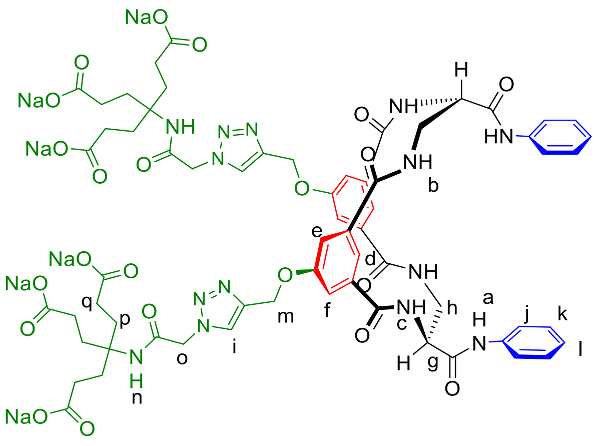

The receptor was characterized in H_2O/D_2O. The assignment of the NMR spectrum was made with the help of 2D TOCSY (see Figures below)).

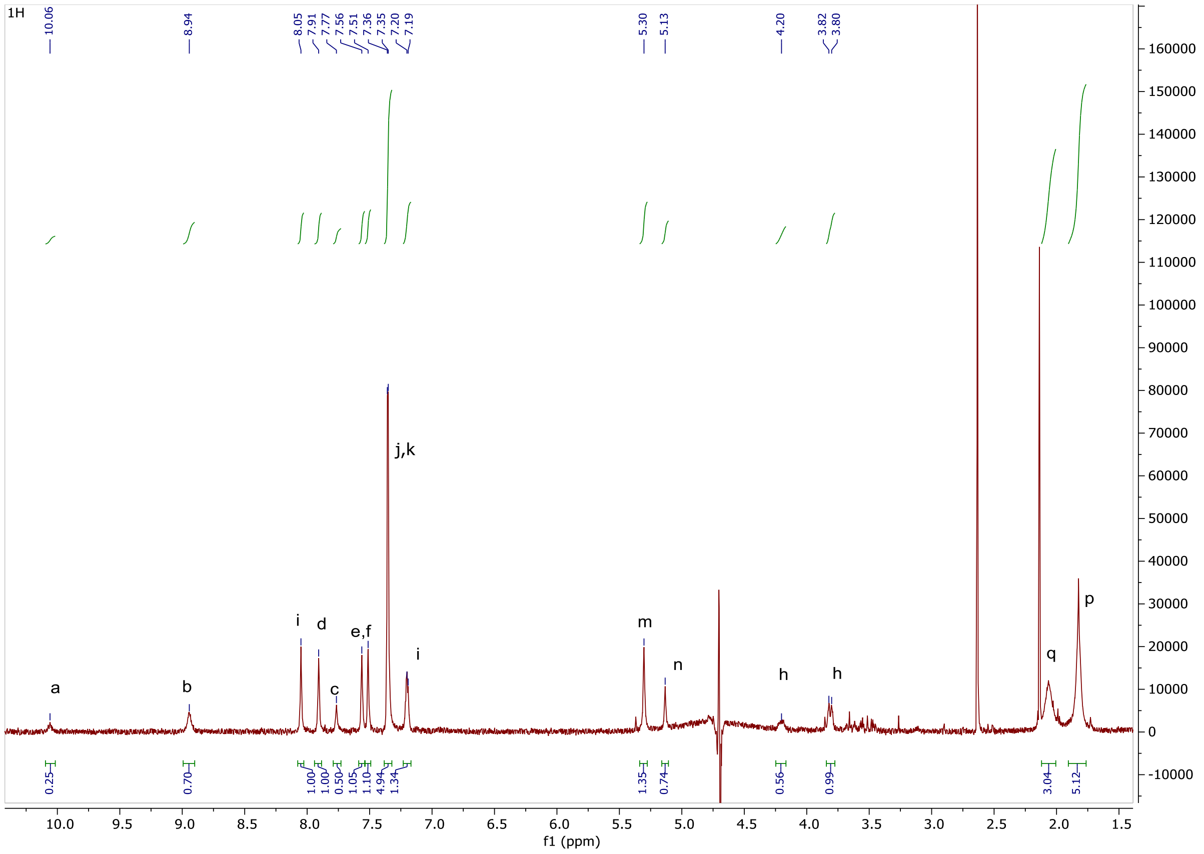

Figure S4. ^{1}H NMR spectrum (600 MHz) of receptor **8b** in 9:1 H_2O/D_2O.

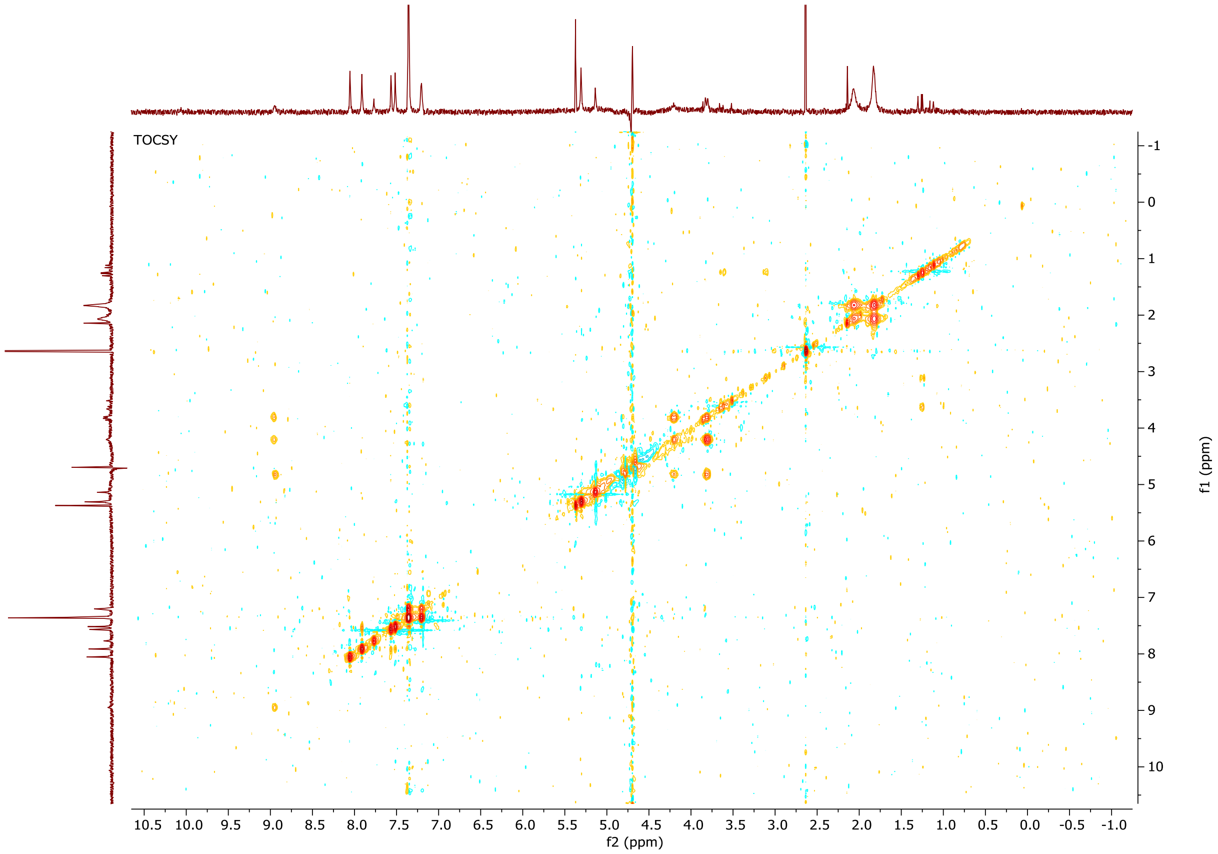

Figure S5. 2D TOCSY NMR spectrum (600 MHz) of receptor **8b** in 9:1 H_2O/D_2O.

Receptor 9a

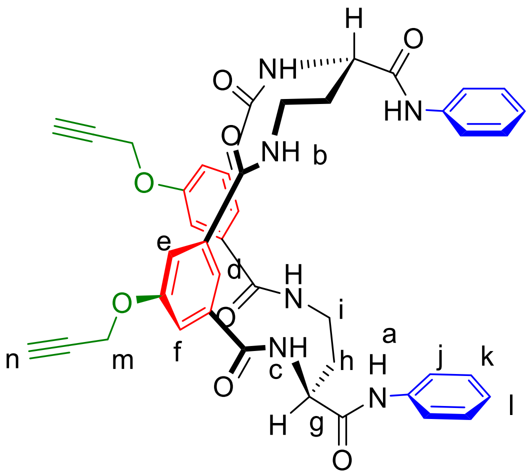

The assignment of the NMR spectrum in DMSO-d^6 was made with the help of 2D COSY and HSQC (see Figures below).

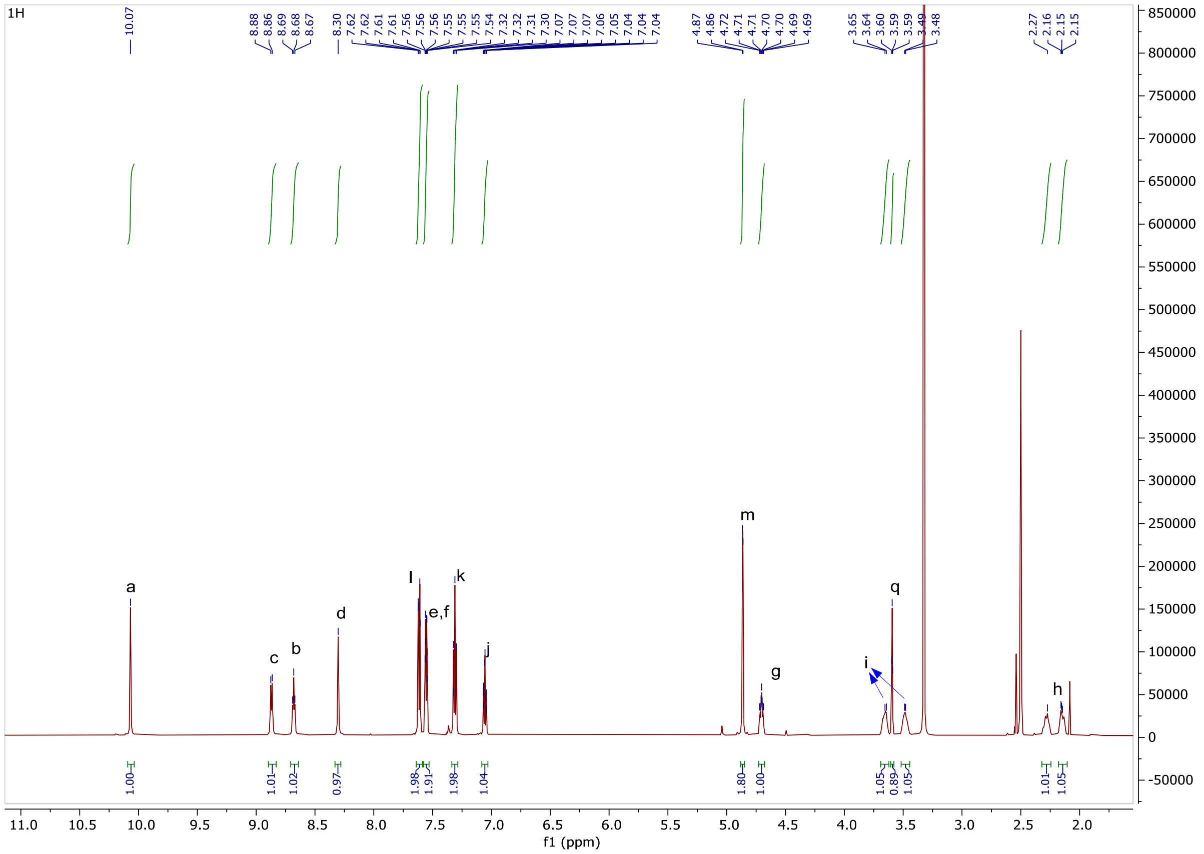

Figure S6. ^{1}H NMR spectrum (600 MHz) of receptor **9a** in DMSO-d^6.

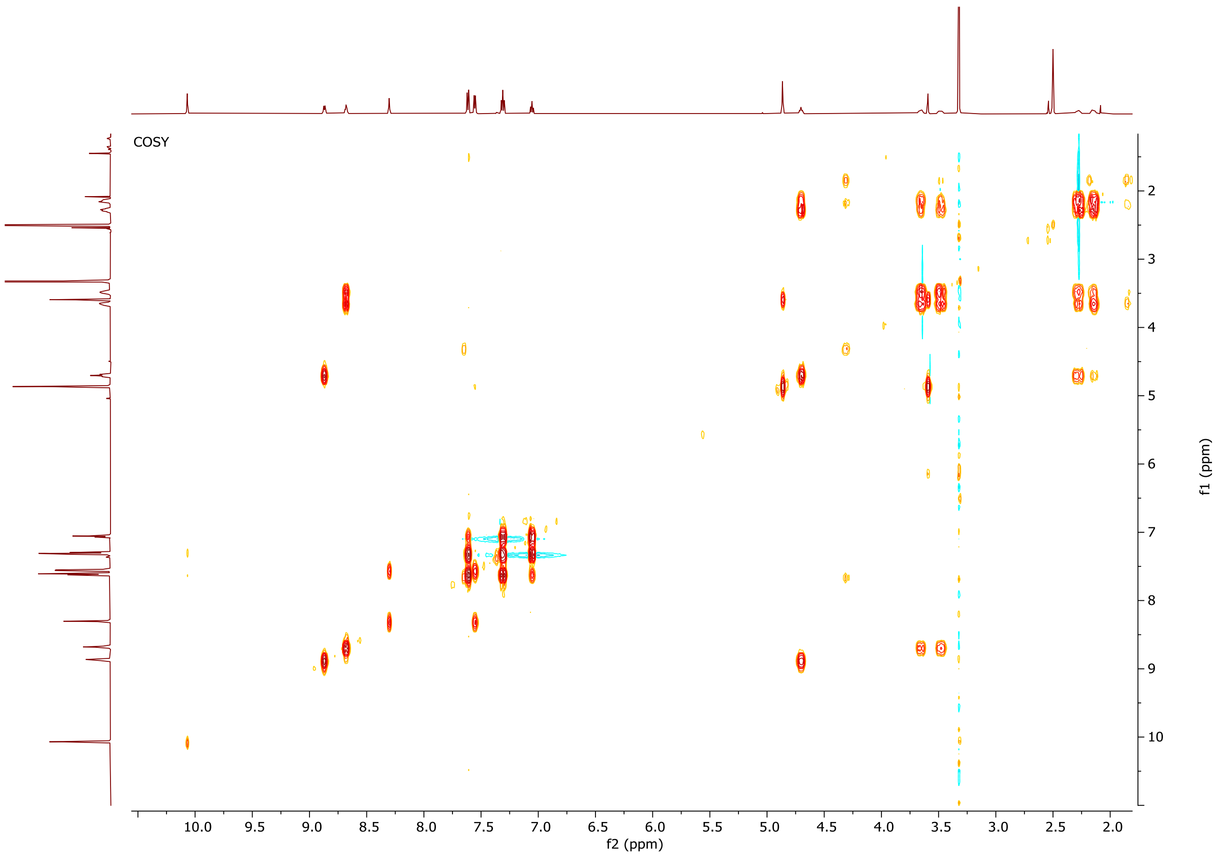

Figure S7. 2D COSY NMR spectrum (600 MHz) of receptor **9a** in DMSO-*d*^{6}.

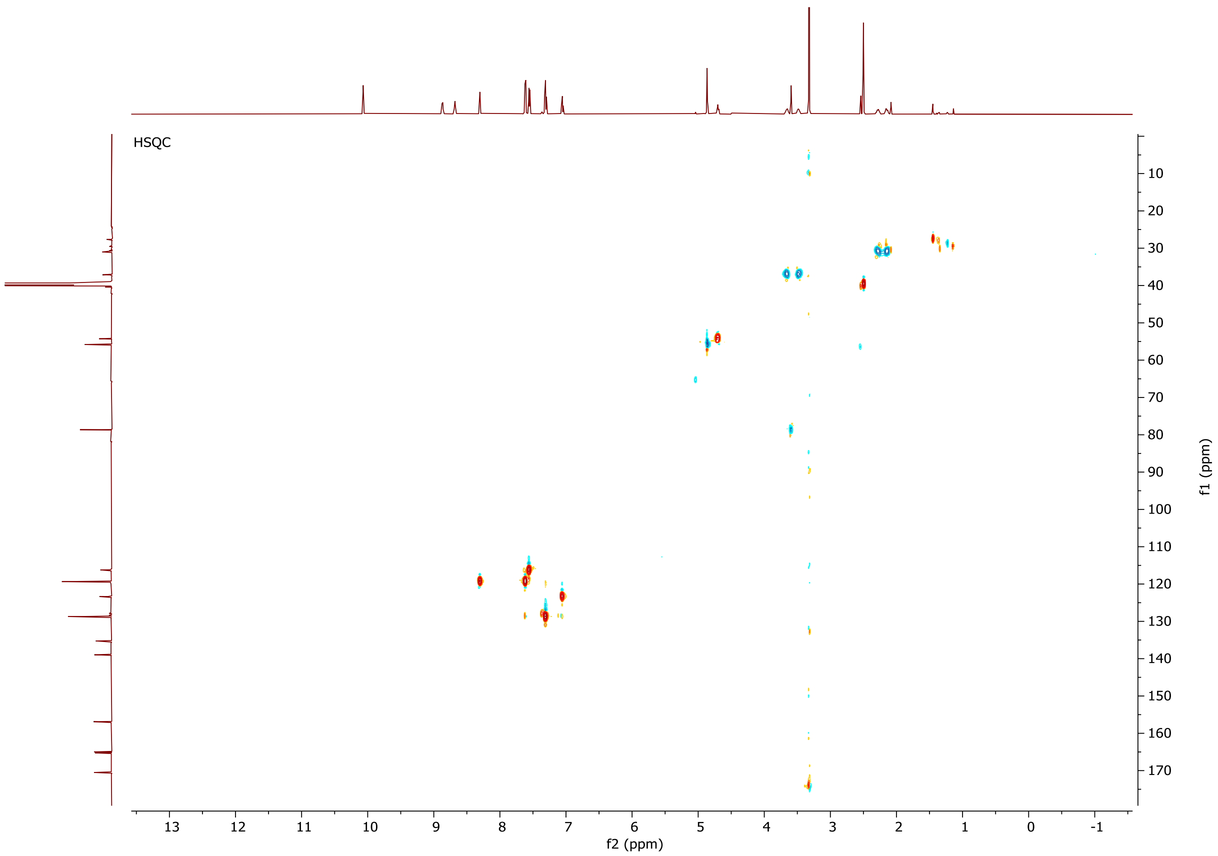

Figure S8. 2D HSQC NMR spectrum (600 MHz) of receptor **9a** in DMSO-*d*^{6}.

Receptor 9b

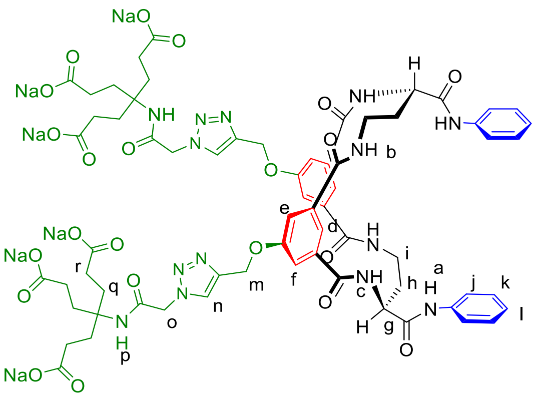

The receptor was characterized in 100 % D_2O and 9:1 H_2O/D_2O. The assignment of the NMR spectrum was made with the help of 2D COSY, TOCSY and HSQC (see Figures below).

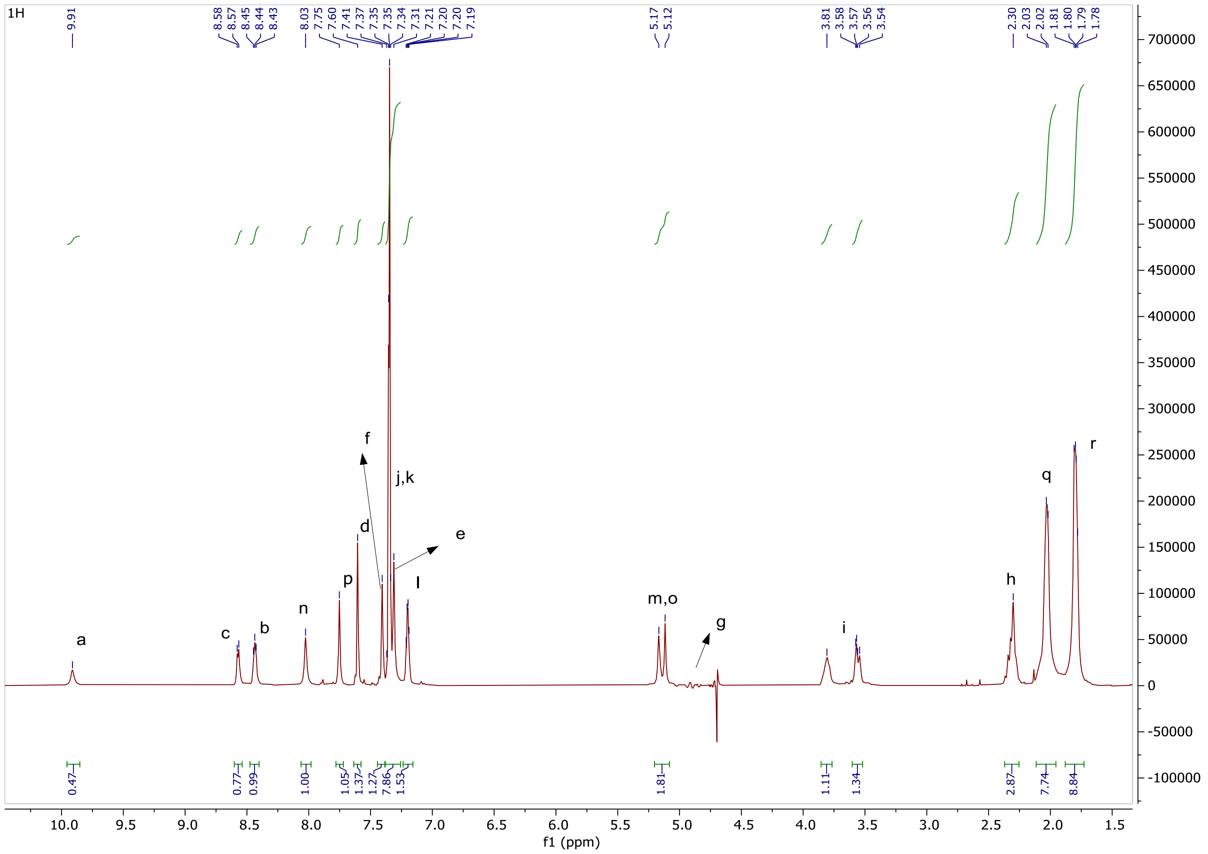

Figure S9. ^{1}H NMR spectrum (600 MHz) of receptor **9b** in 9:1 H_2O/D_2O.

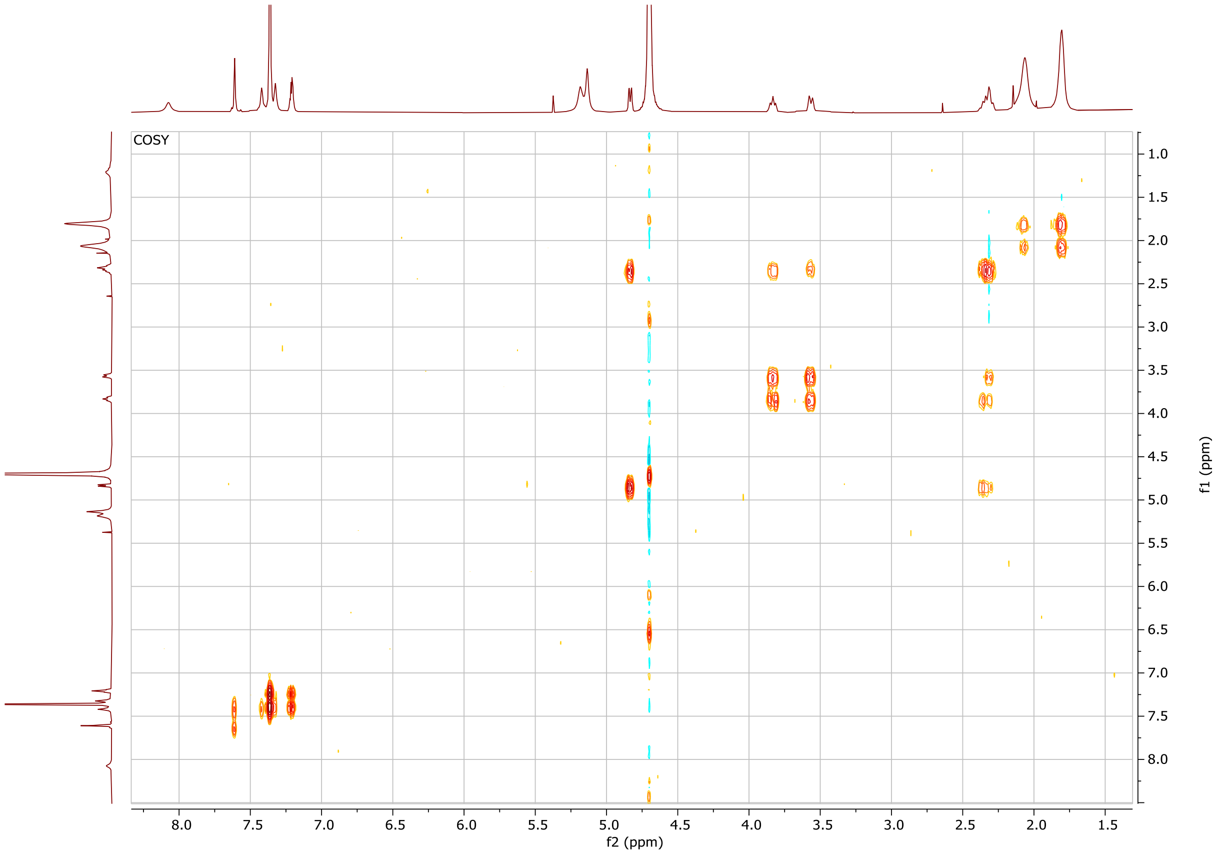

Figure S10. 2D COSY NMR spectrum (600 MHz) of receptor **9b** in D_2O.

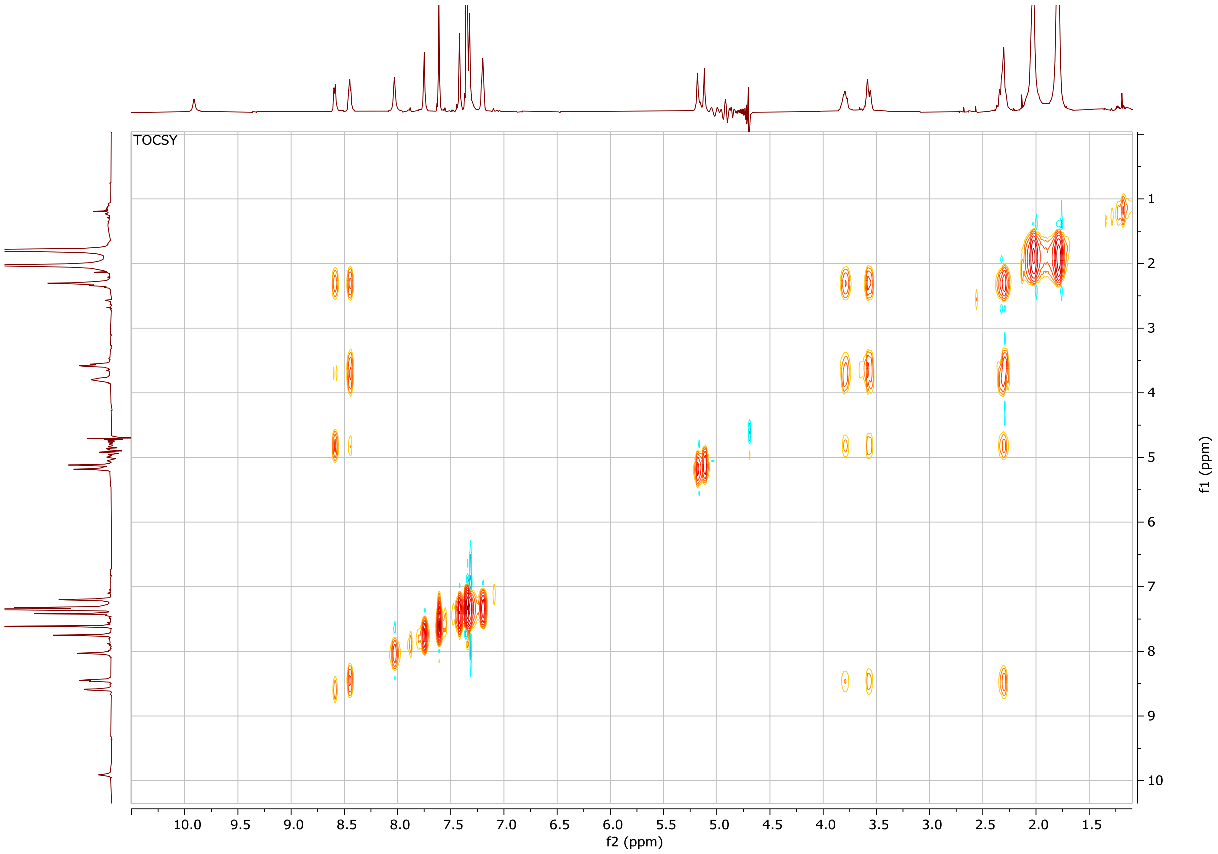

Figure S11. 2D TOCSY NMR spectrum (600 MHz) of receptor **9b** in 9:1 H_2O/D_2O.

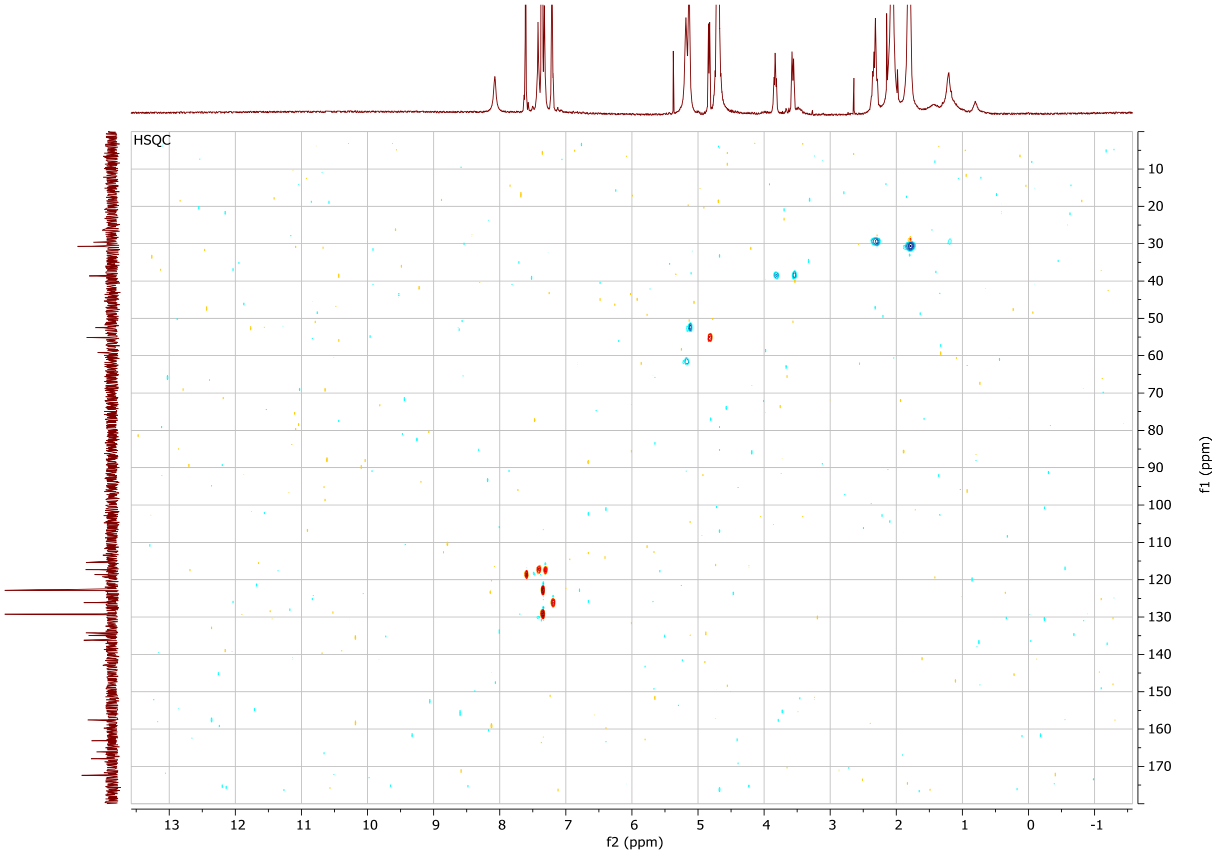

Figure S12. 2D HSQC NMR spectrum (600 MHz) of receptor **9b** in D_2O.

Receptor 10a

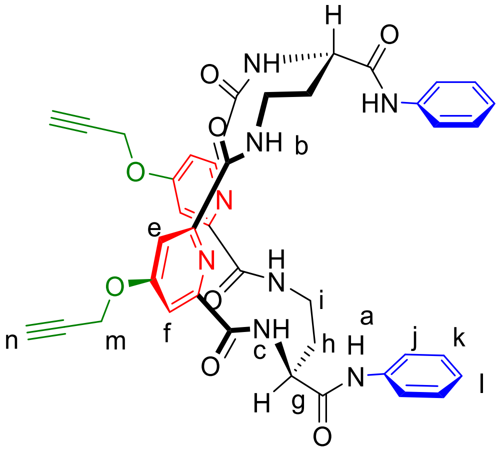

The assignment of the NMR spectrum in DMSO-d^6 was made with the help of 2D COSY and HSQC (see Figures below).

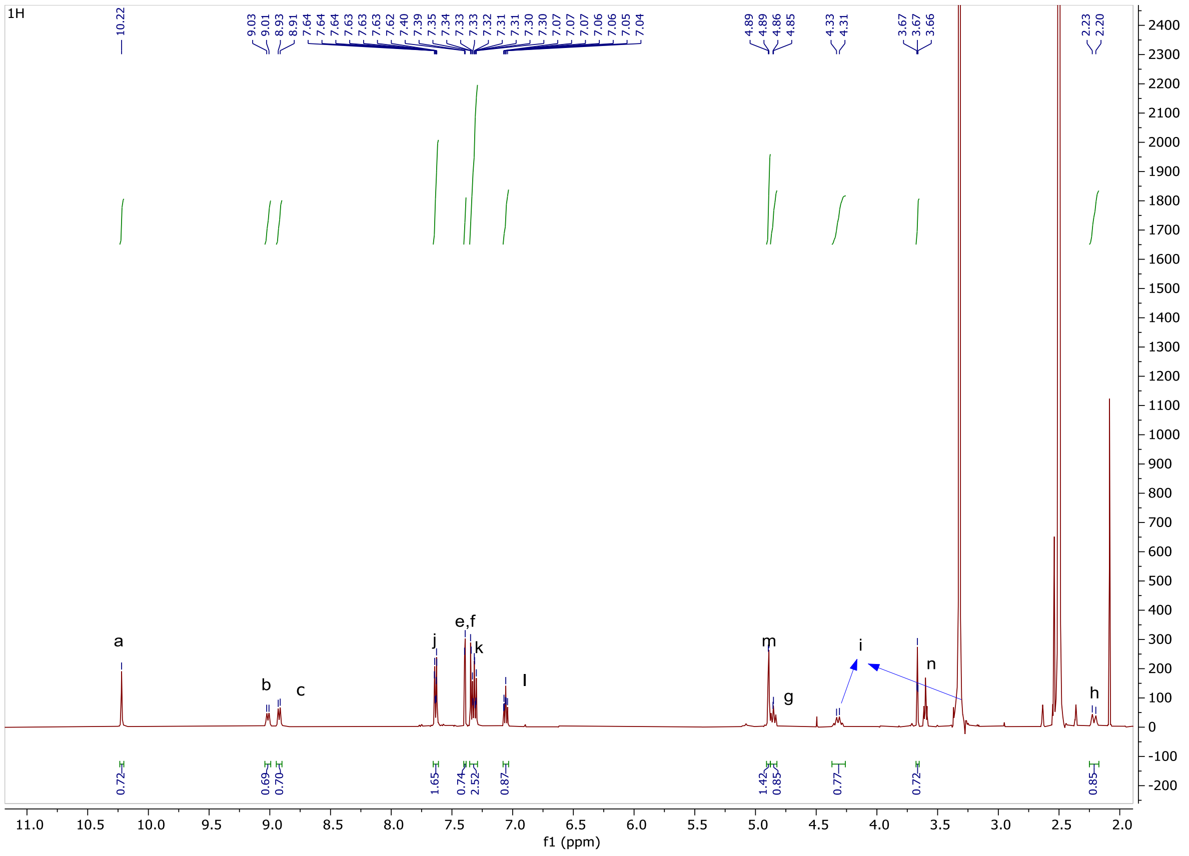

Figure S13. ^{1}H NMR spectrum (500 MHz) of receptor **10a** in DMSO-d^6.

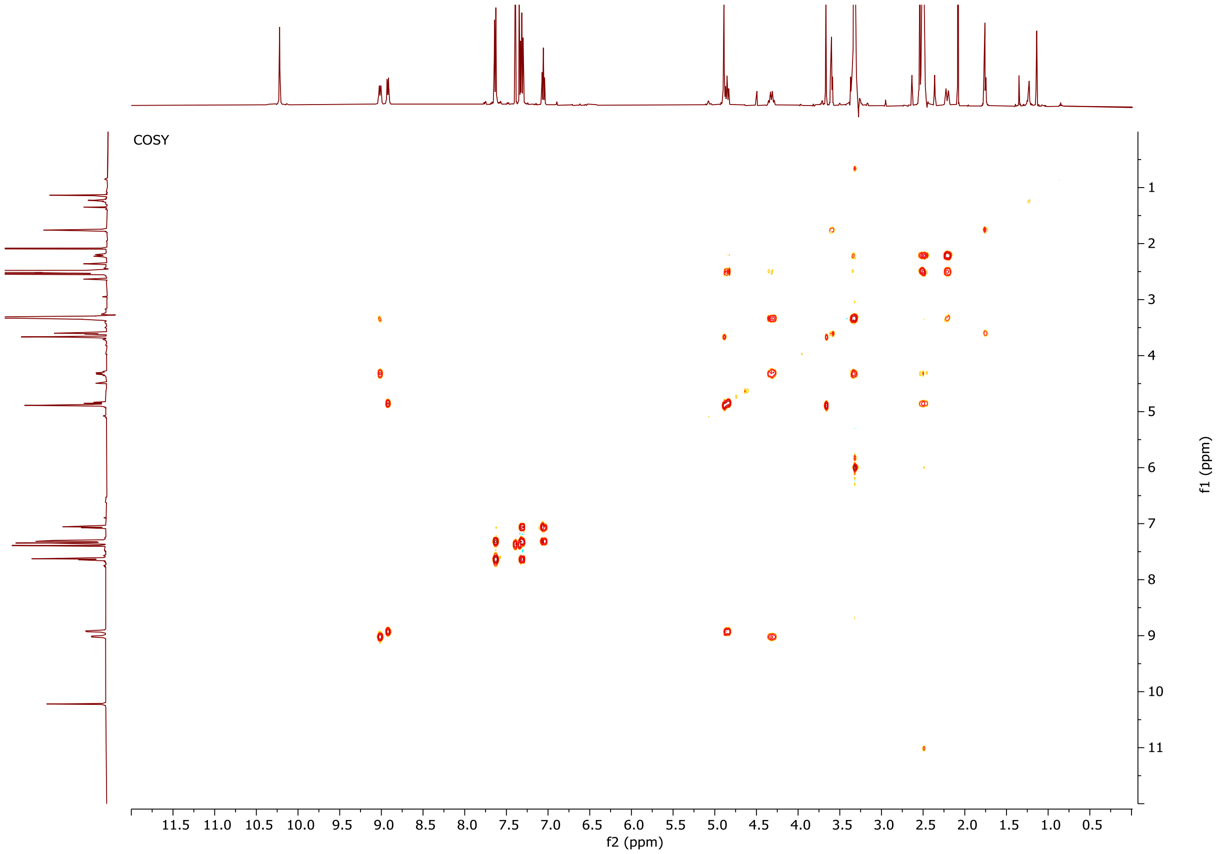

Figure S14. 2D COSY NMR spectrum (500 MHz) of receptor **10a** in DMSO-*d*^{6}.

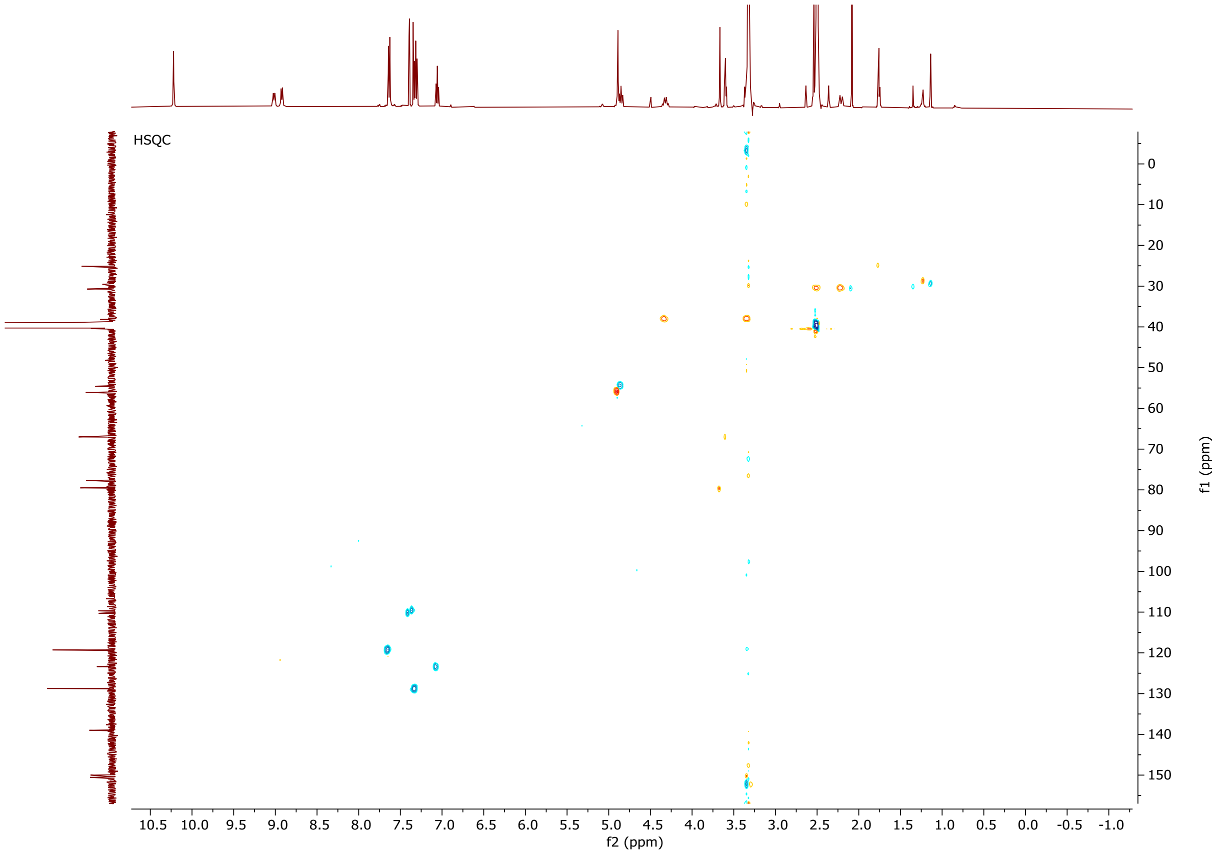

Figure S15. 2D HSQC NMR spectrum (500 MHz) of receptor **10a** in DMSO-*d*^{6}.

Receptor 10b

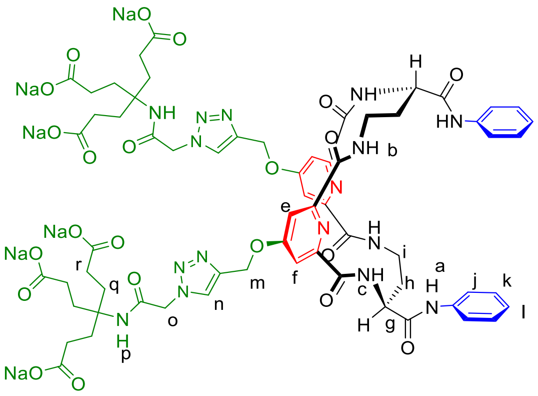

The receptor was characterized in D_2O. The assignment of the NMR spectrum was made with the help of 2D COSY, TOCSY and HSQC (see Figures below).

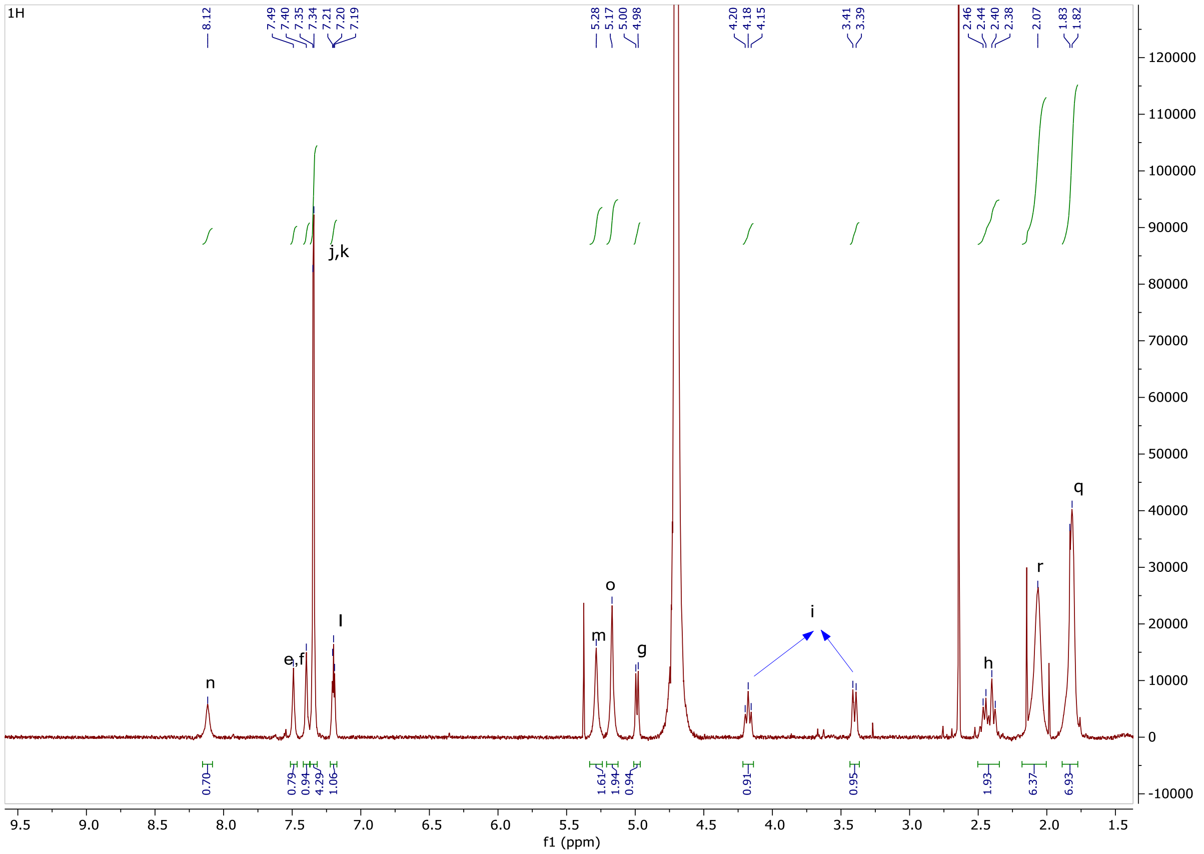

Figure S16. ^{1}H NMR spectrum (600 MHz) of receptor **10b** in D_2O.

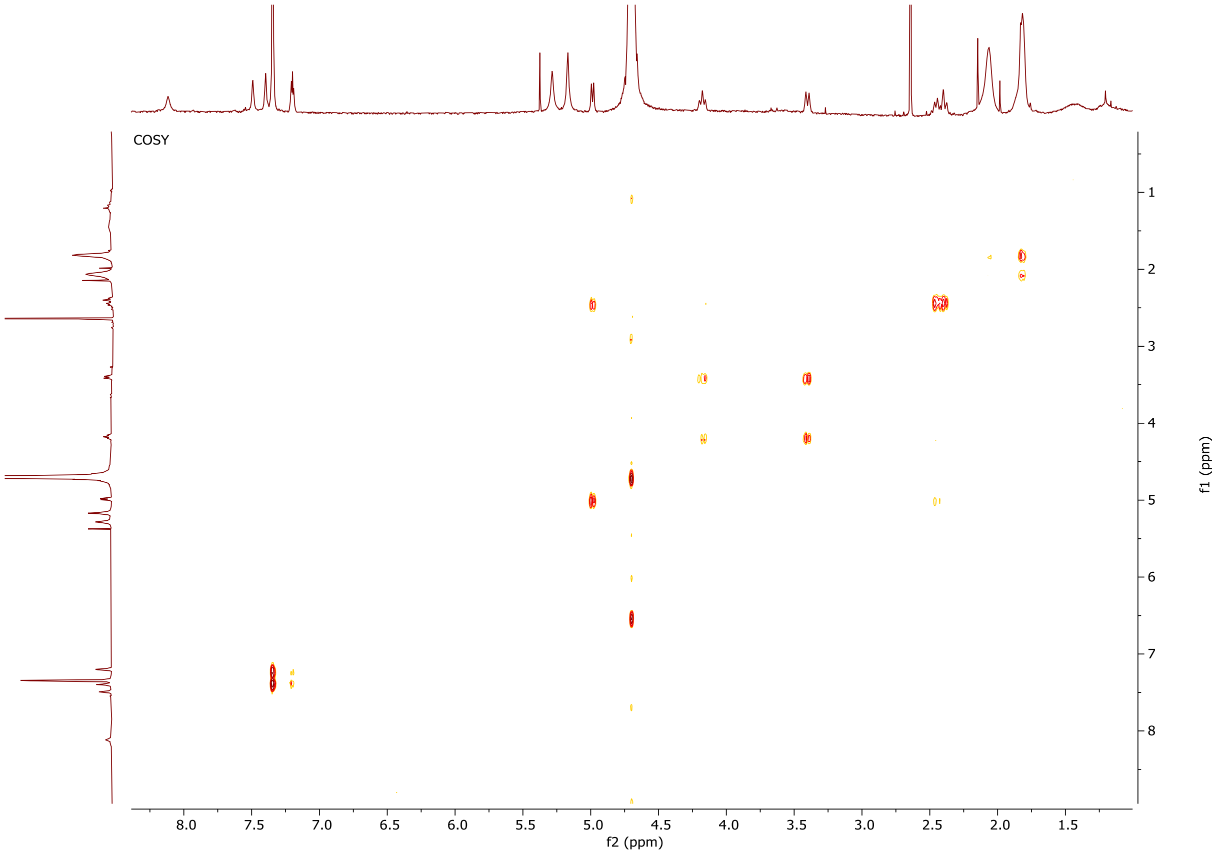

Figure S17. 2D COSY NMR spectrum (600 MHz) of receptor **10b** in D_2O.

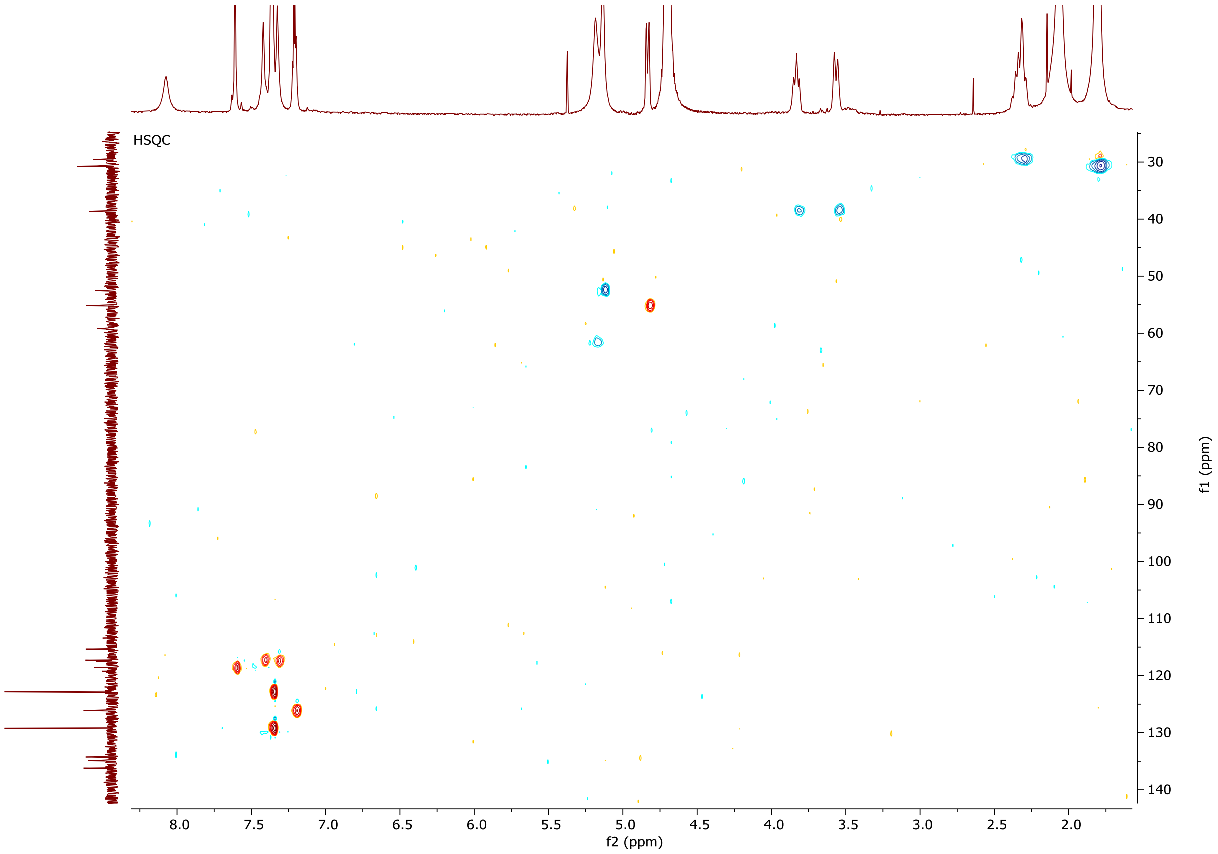

Figure S18. 2D HSQC NMR spectrum (600 MHz) of receptor **10b** in D_2O.

Receptor 20

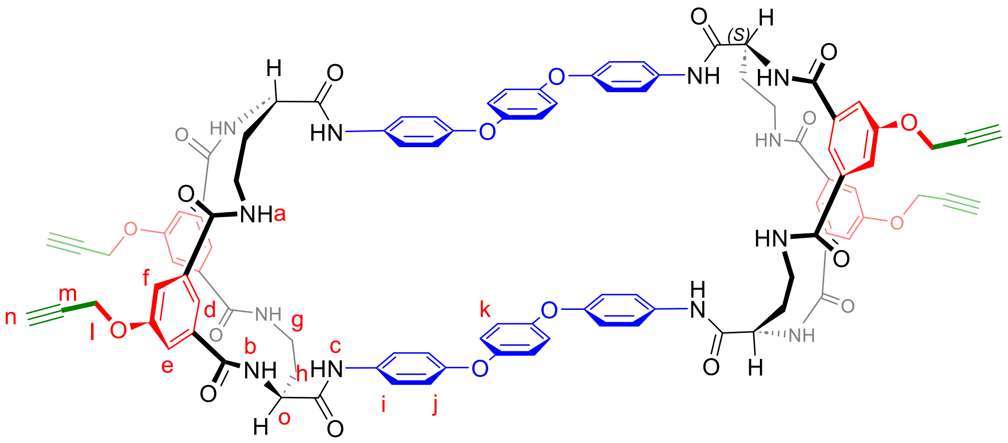

The assignment of the NMR spectrum in DMSO-d^6 was made with the help of 2D COSY and HSQC (see Figures below).

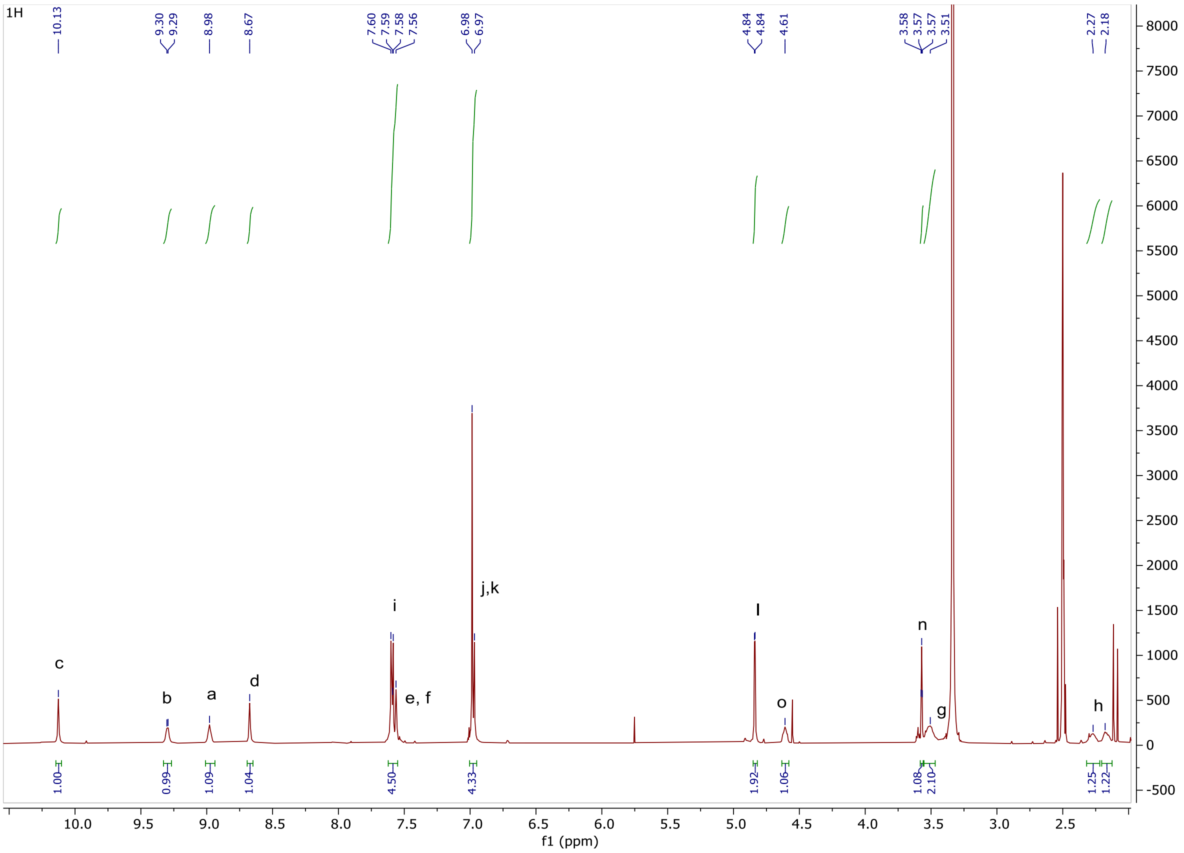

Figure S19. ^{1}H NMR spectrum (500 MHz) of receptor **20** in DMSO-d^6.

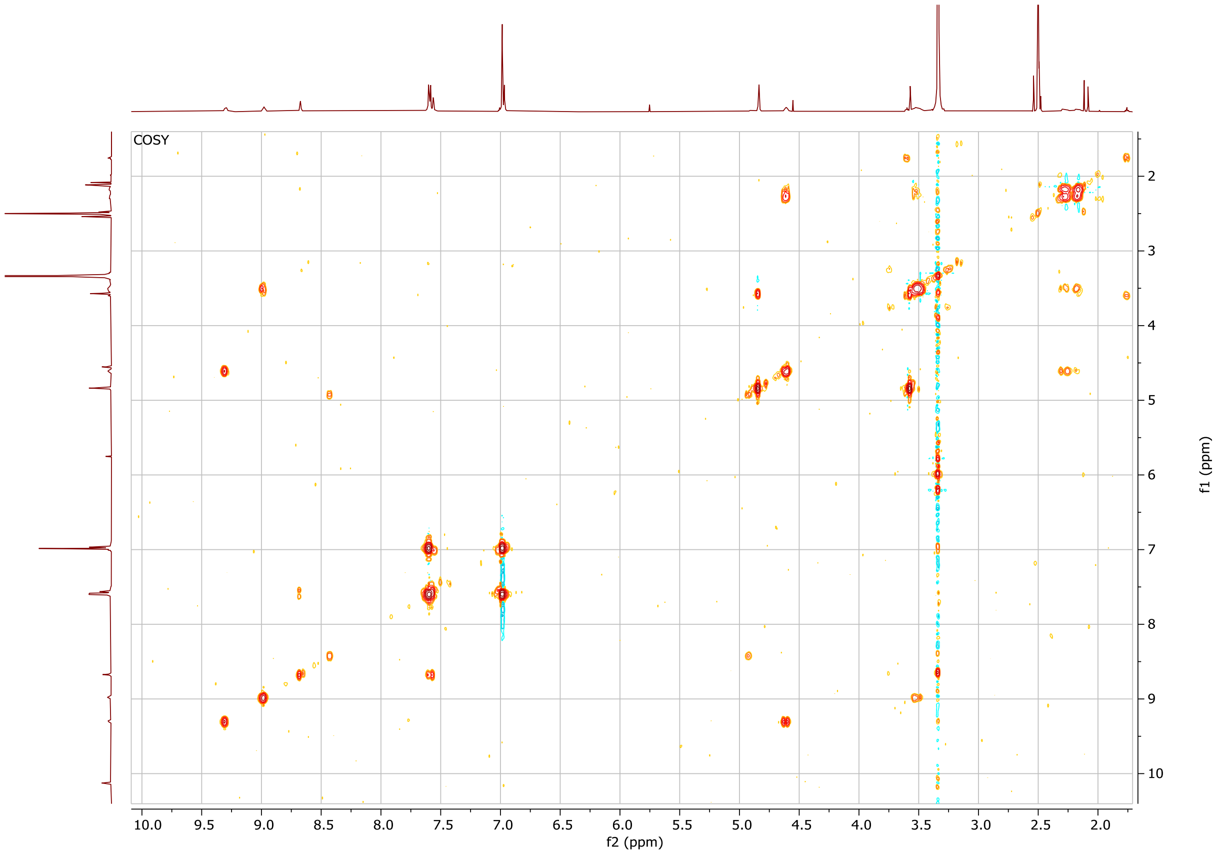

Figure S20. 2D COSY NMR spectrum (500 MHz) of receptor **20** in DMSO-*d*[6].

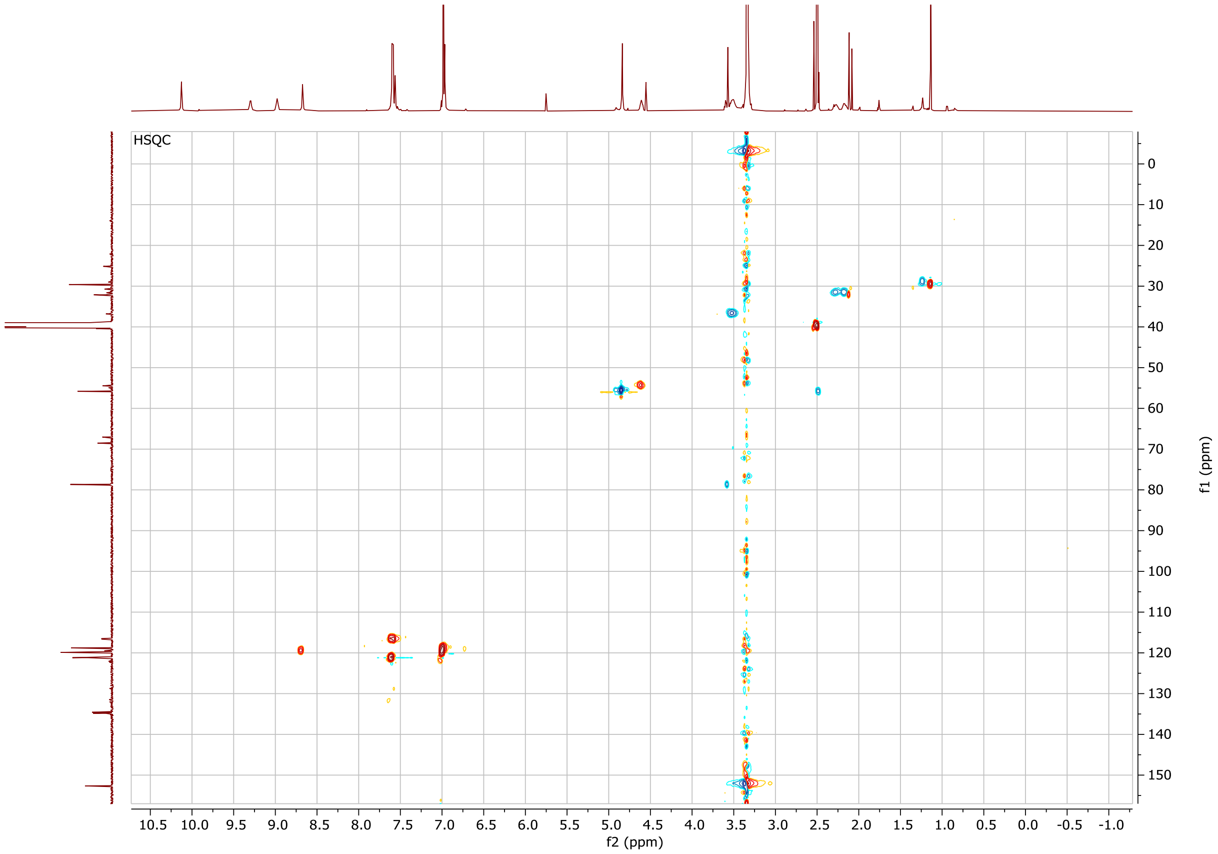

Figure S21. 2D HSQC NMR spectrum (500 MHz) of receptor **20** in DMSO-*d*[6].

Receptor 21

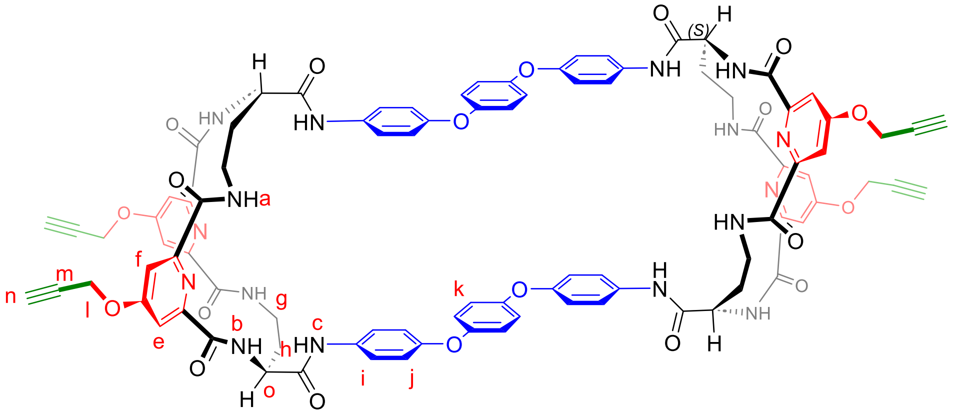

The assignment of the NMR spectrum in DMSO-d^6 was made with the help of 2D COSY and HSQC (see Figures below).

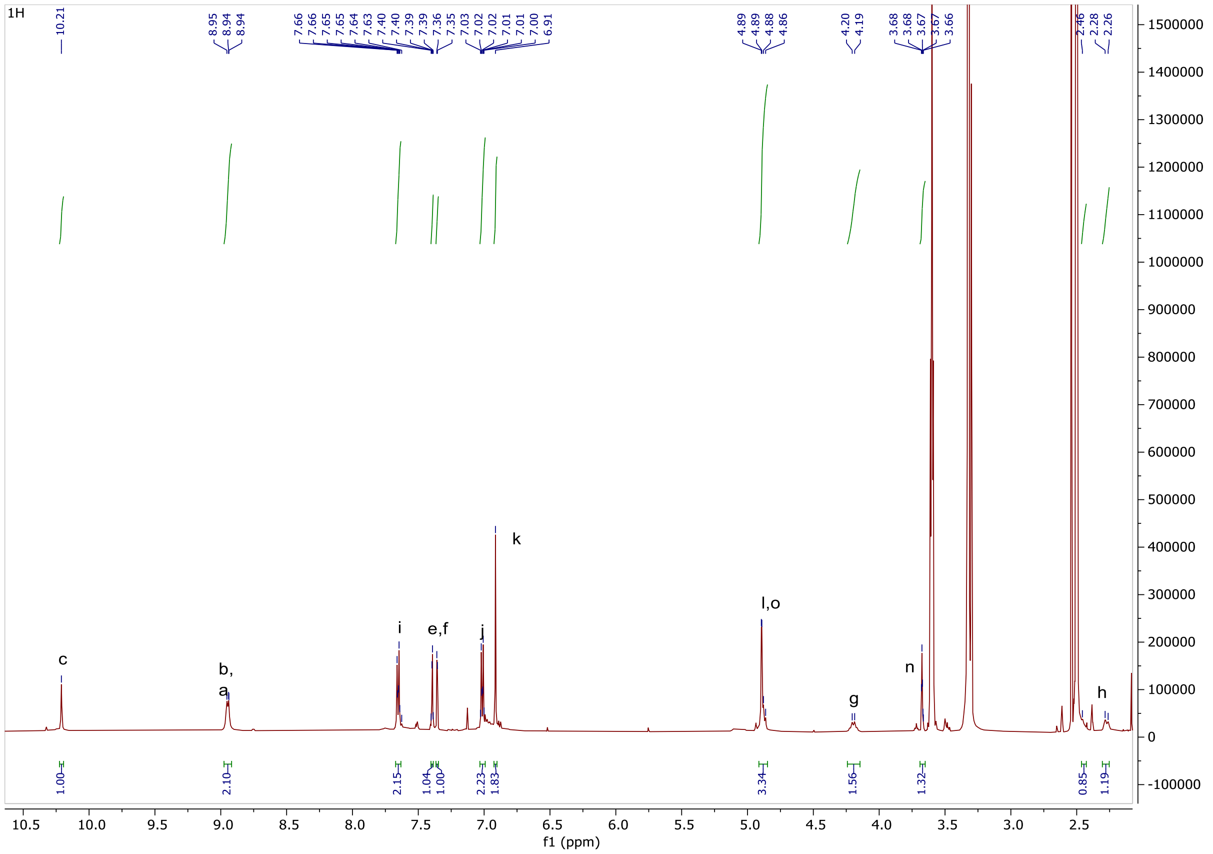

Figure S22. ^{1}H NMR spectrum (500 MHz) of receptor **21** in DMSO-d^6.

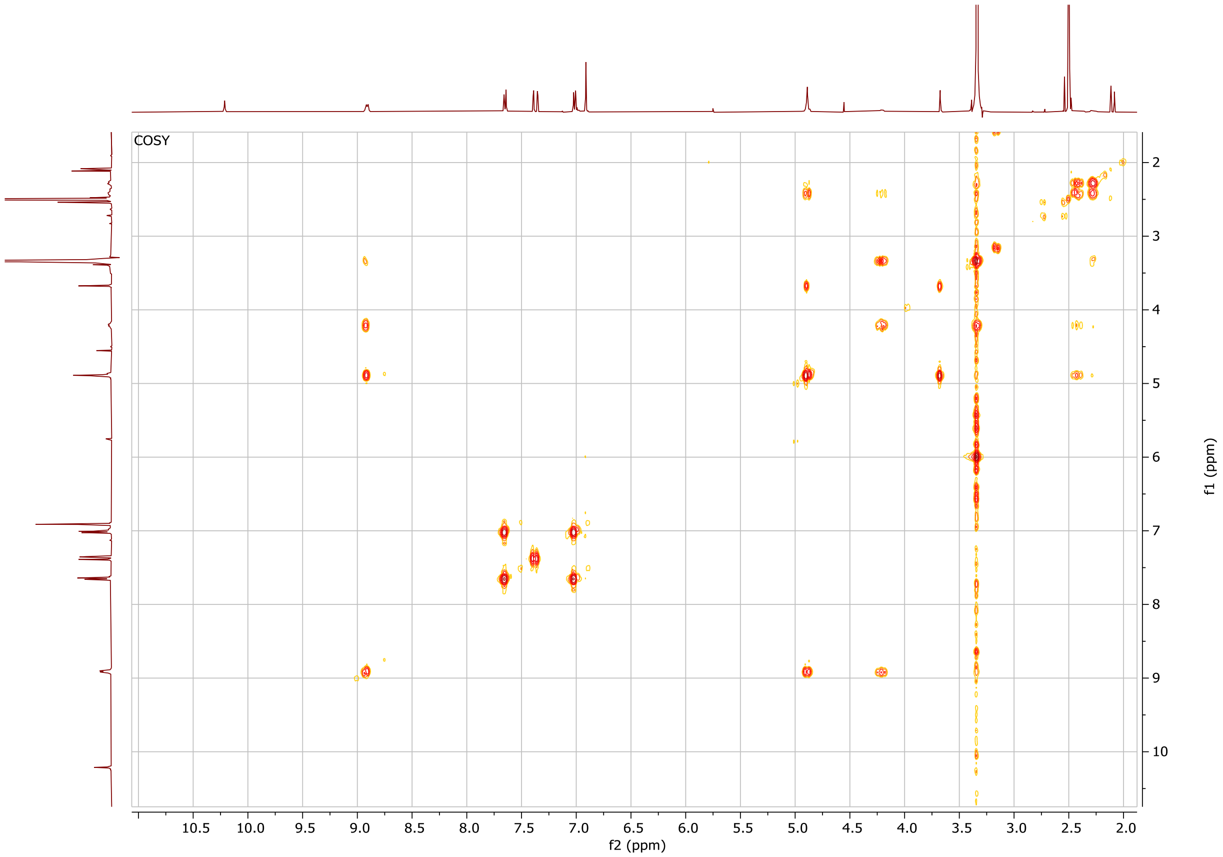

Figure S23. 2D COSY NMR spectrum (500 MHz) of receptor **21** in DMSO-d^6.

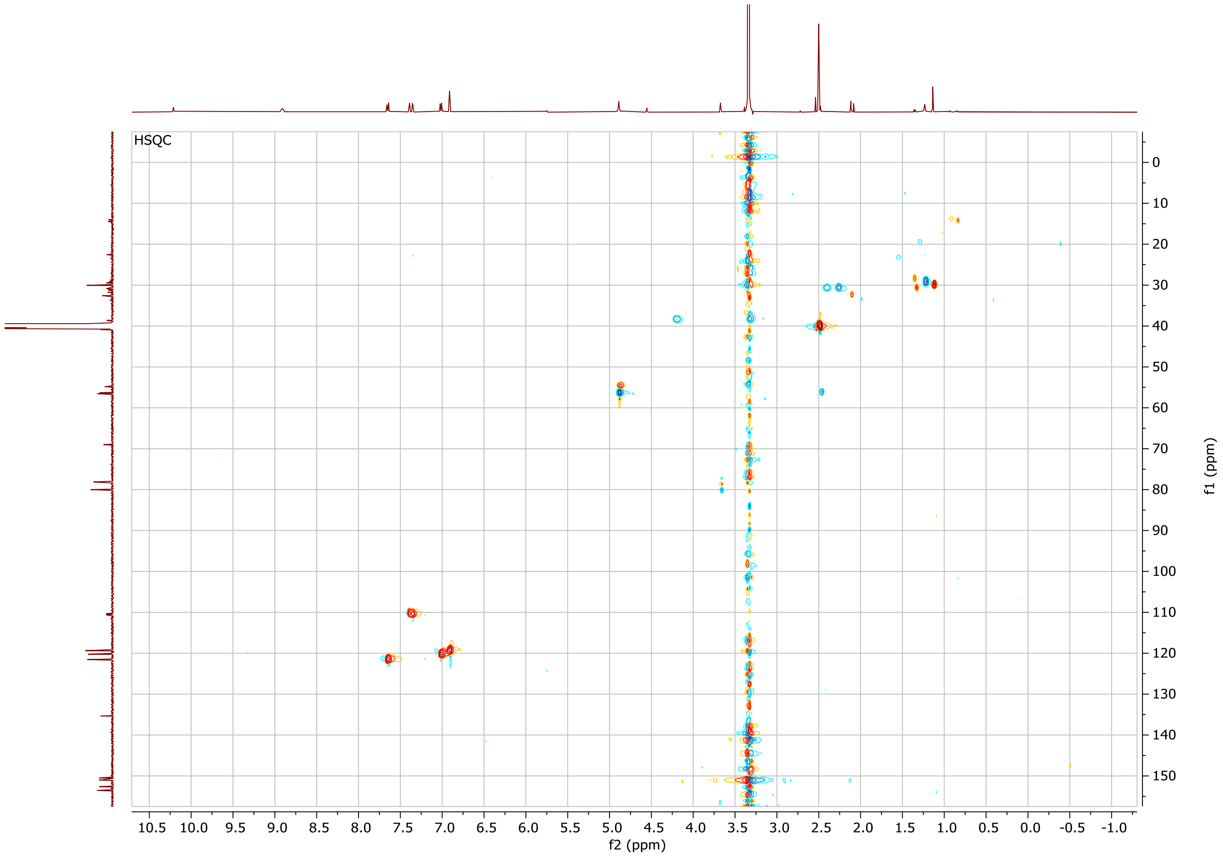

Figure S24. 2D HSQC NMR spectrum (500 MHz) of receptor **21** in DMSO-d^6.

Receptor 5

The receptor was characterized in 9:1 H_2O/D_2O. The assignment of the NMR spectrum was made with the help of 2D TOCSY and HSQC (see Figures below).

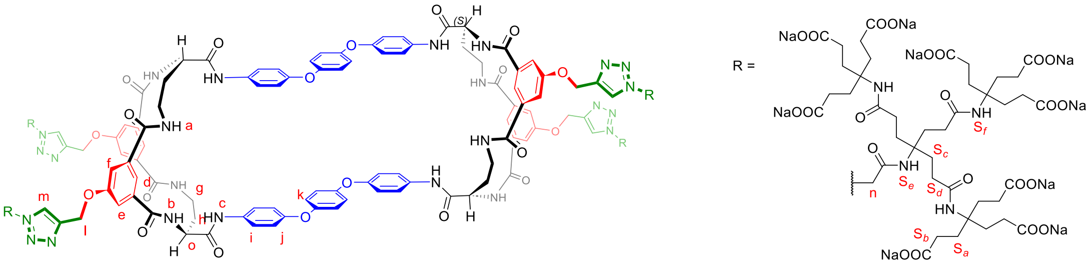

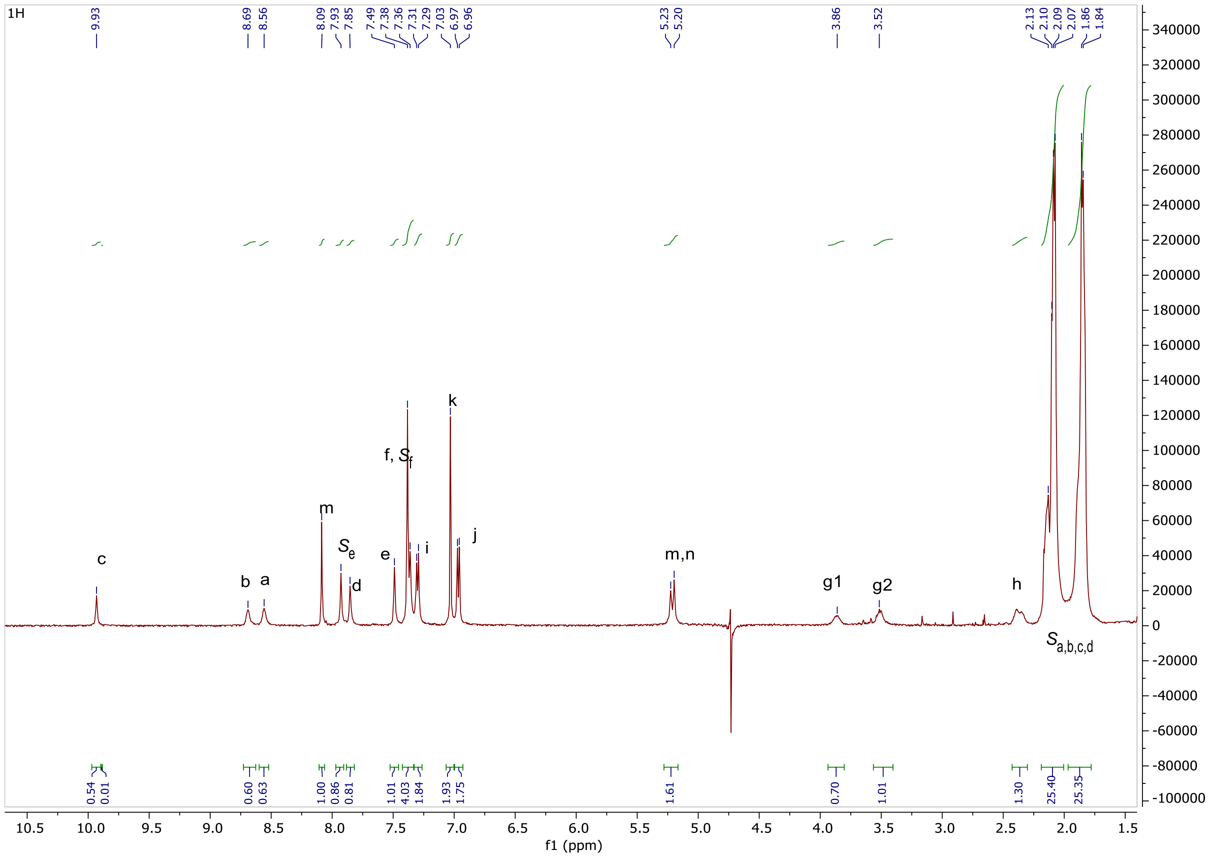

Figure S25. ^1H NMR spectrum (600 MHz) of receptor **5** in 9:1 H_2O/D_2O.

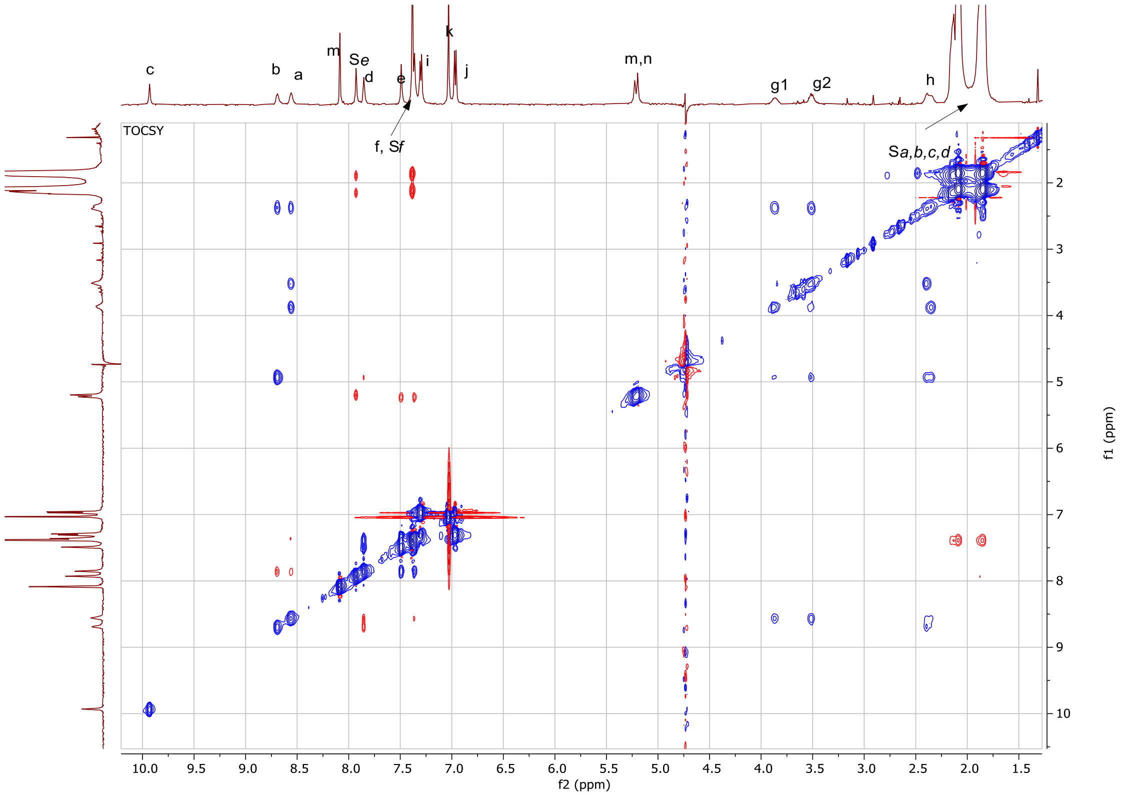

Figure S26. 2D TOCSY NMR spectrum (600 MHz) of receptor **5** in 9:1 H_2O/D_2O.

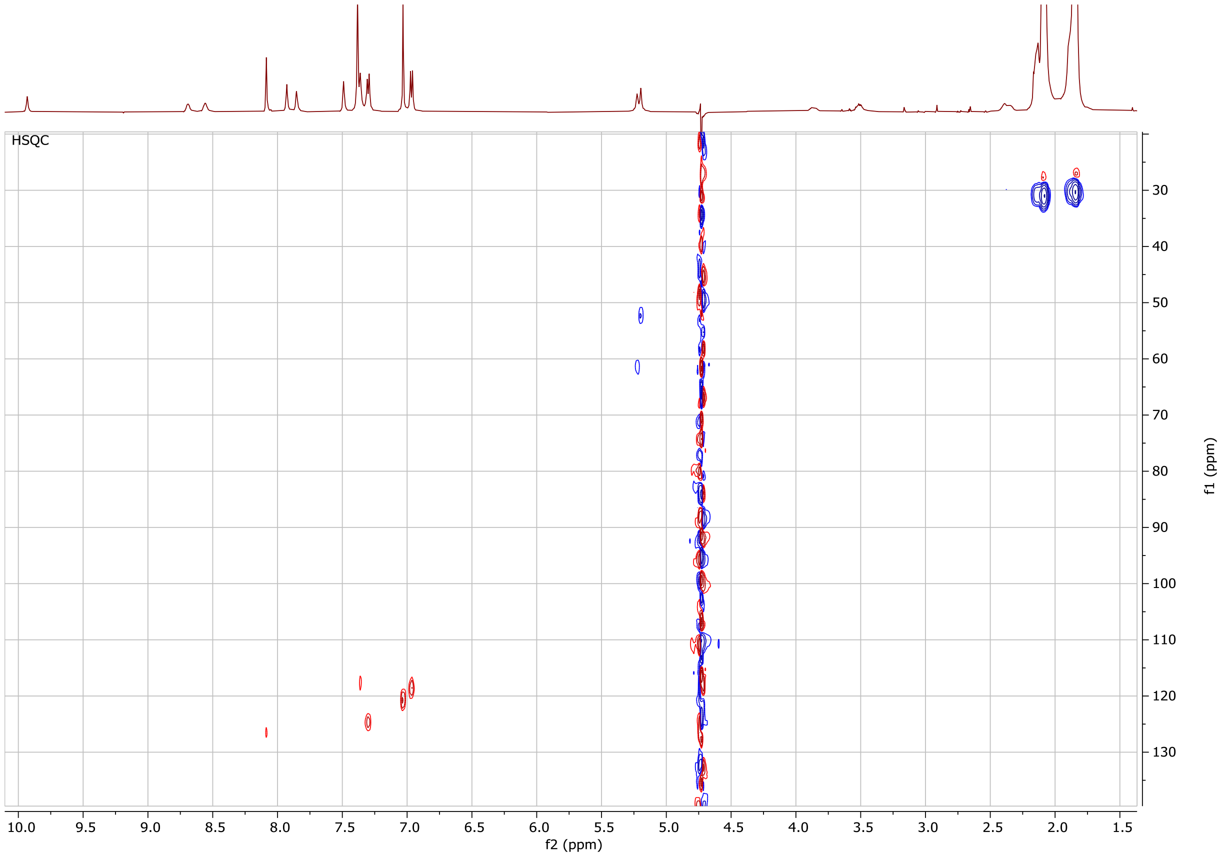

Figure S27. 2D HSQC NMR spectrum (600 MHz) of receptor **5** in 9:1 H_2O/D_2O.

Receptor 6

The receptor was characterized in 9:1 H_2O/D_2O. The assignment of the NMR spectrum was made with the help of 2D TOCSY and HSQC (see Figures below).

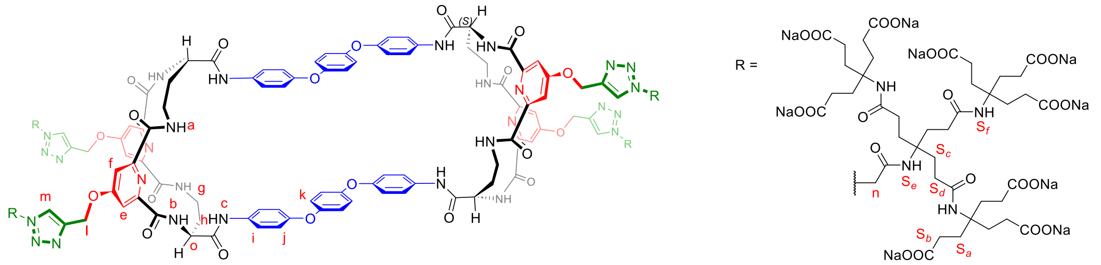

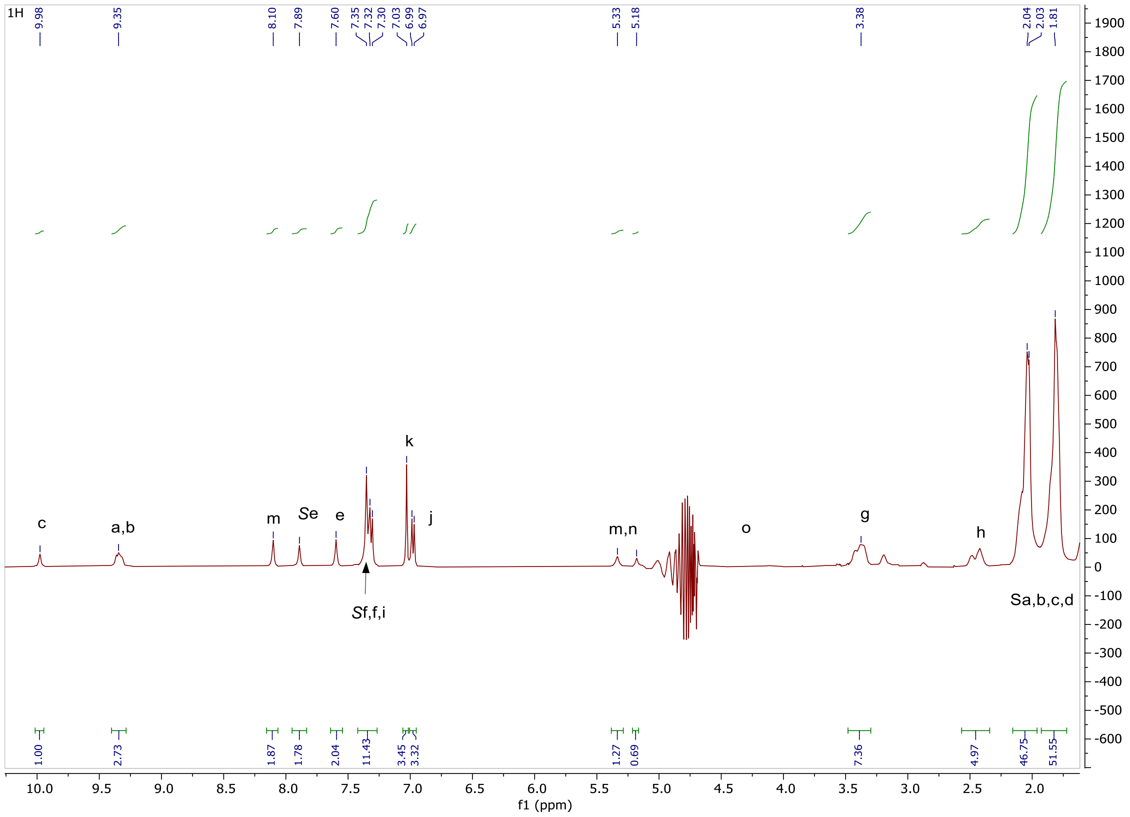

Figure S28. ^{1}H NMR spectrum (600 MHz) of receptor **6** in 9:1 H_2O/D_2O.

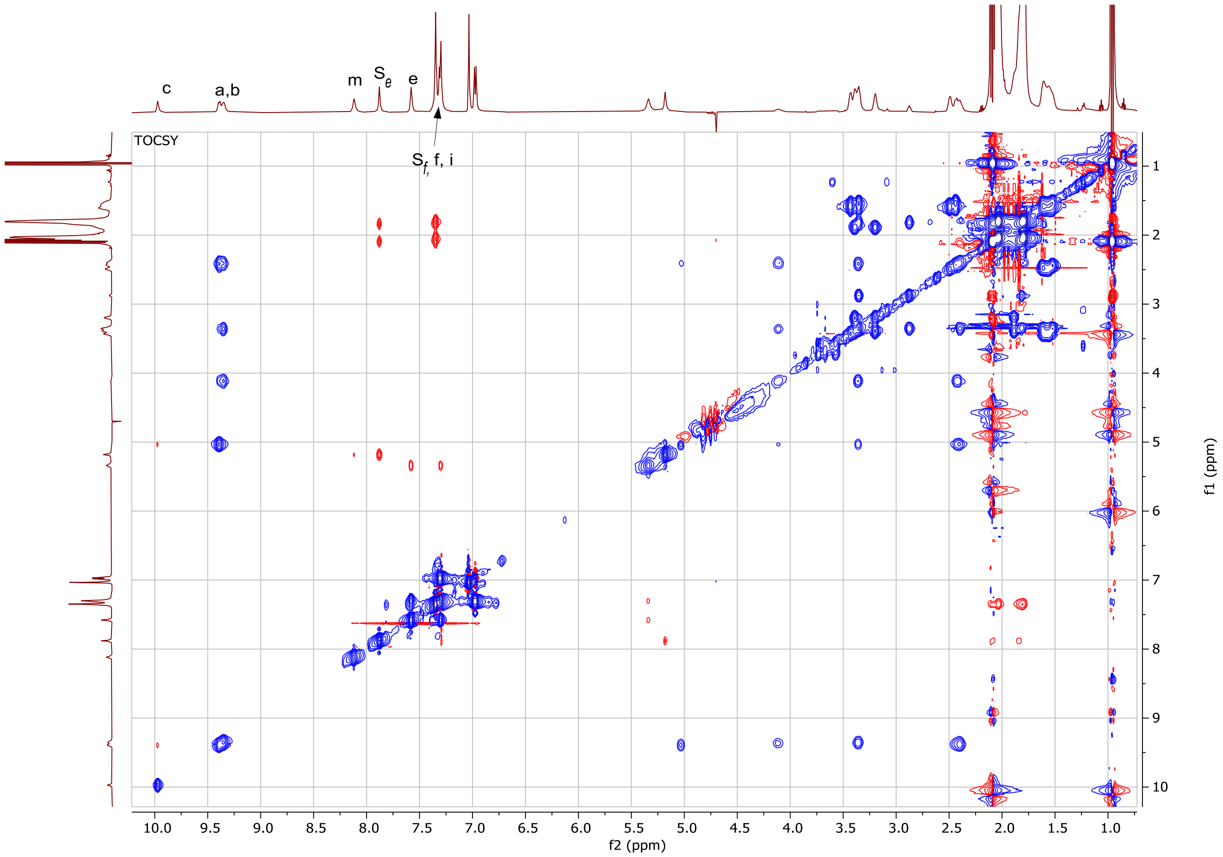

Figure S29. 2D TOCSY NMR spectrum (600 MHz) of receptor **6** with sodium propionate in 9:1 H_2O/D_2O. The addition of propionate helps the assignment by resolving peaks between δ 7.20 – 7.40 ppm.

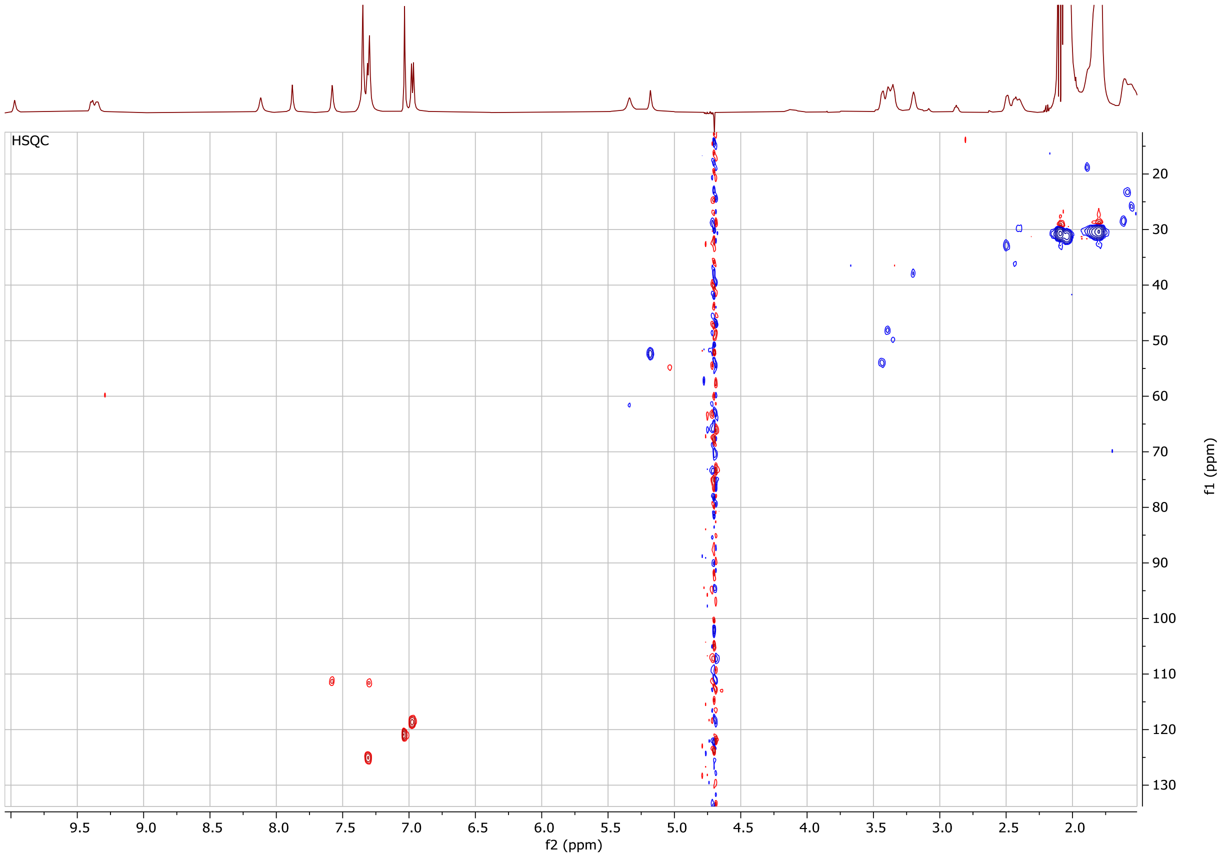

Figure S30. 2D HSQC NMR spectrum (600 MHz) of receptor **6** with sodium propionate in 9:1 H_2O/D_2O. The addition of propionate helps the assignment by resolving peaks between δ 7.20 – 7.40 ppm.

1.7 Dilution studies

The solid receptors were dissolved in corresponding solvents (DMSO-d^6 or D_2O or 9:1 H_2O/D_2O) to make solutions with specific starting concentrations for each dilution study. 500 µL of the above solution was transferred into an NMR tube and the ^{1}H NMR spectrum was acquired. The receptor solution was then diluted in the NMR tube by adding the same solvent, and a series of ^{1}H NMR spectra was obtained.

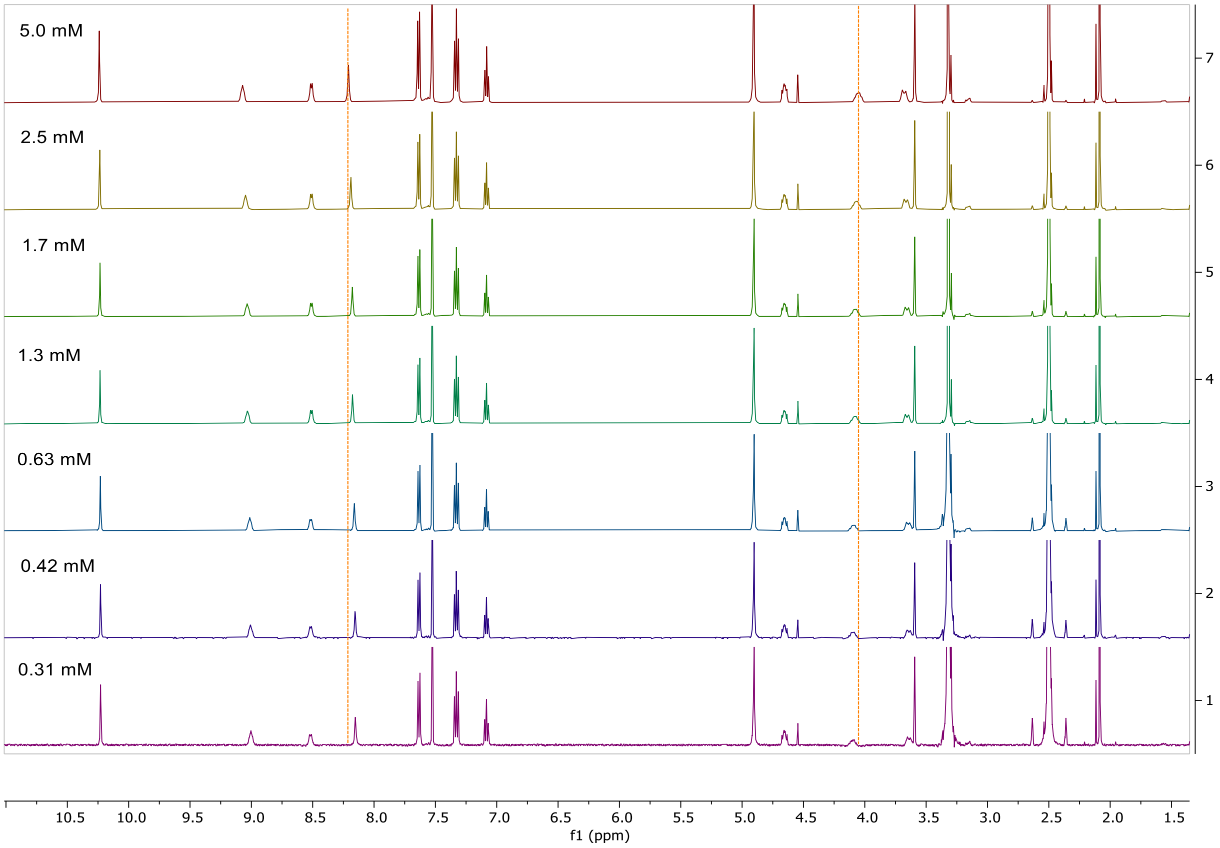

Figure S31. ^{1}H NMR spectra showing receptor **8a** at various concentrations in DMSO-d^6. See yellow reference line for movements of proton d at ~8.2 p.p.m. The receptor is taken to be monomeric below 0.42 mM.

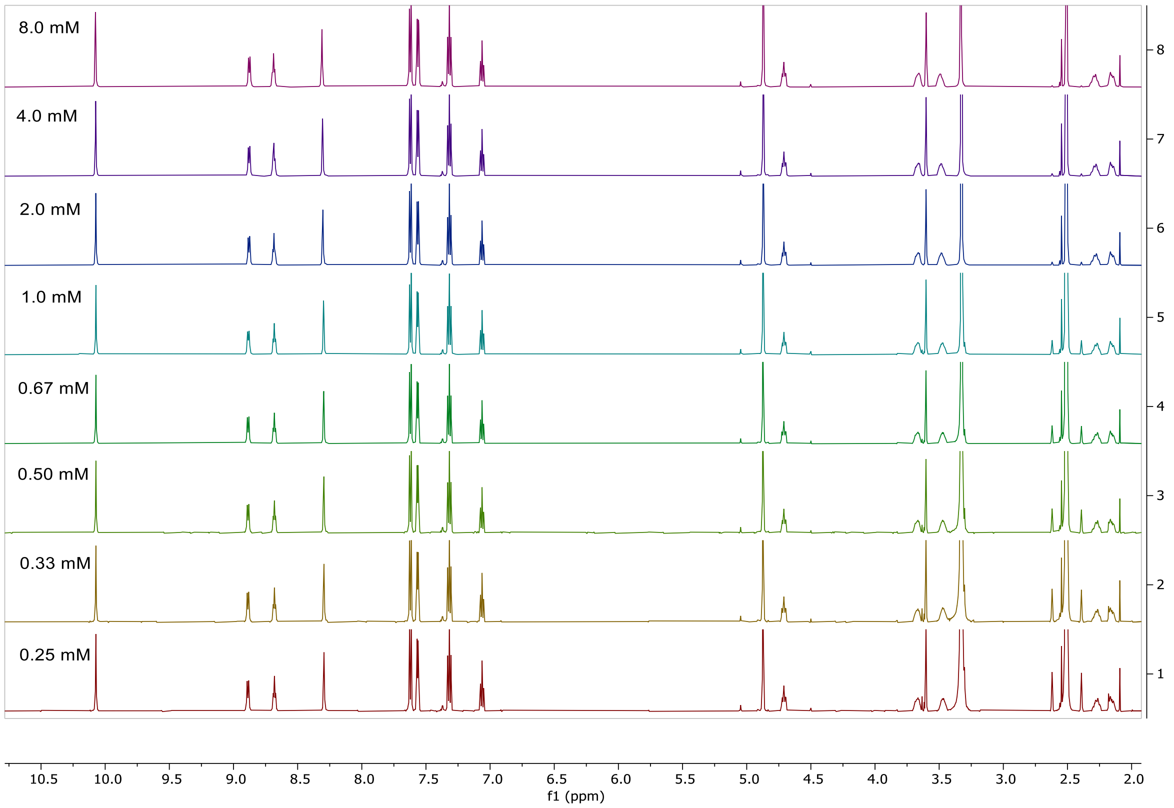

Figure S32. ^{1}H NMR spectra showing receptor **9a** at various concentrations in DMSO-*d*[6]. The receptor is taken to be monomeric below 0.67 mM.

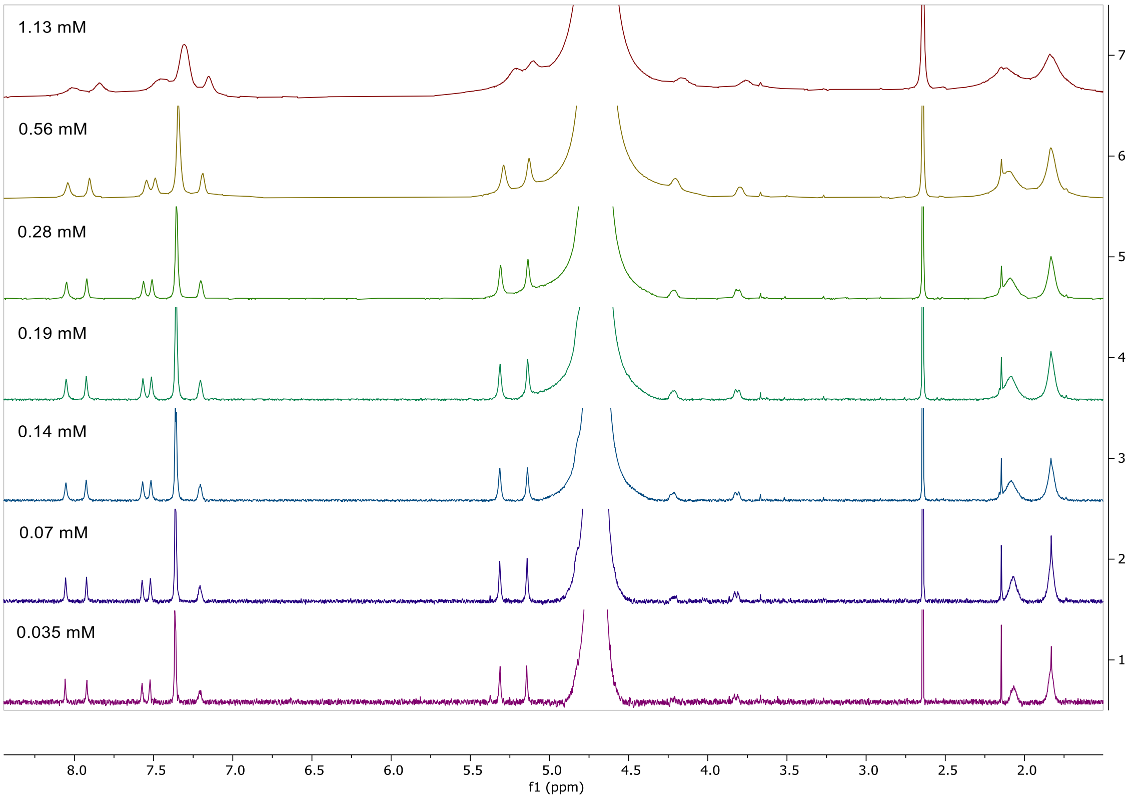

Figure S33. ^{1}H NMR spectra showing receptor **8b** at various concentrations in D_2O. The receptor is taken to be monomeric below 0.07 mM.

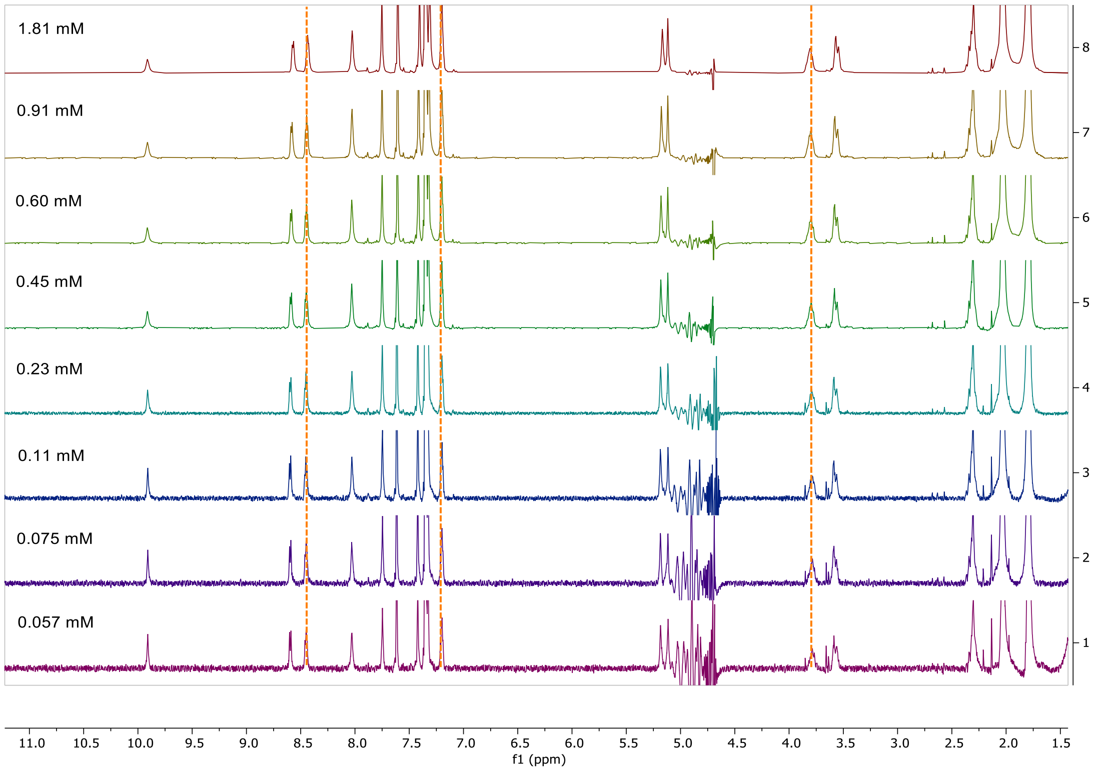

Figure S34. ^{1}H NMR spectra showing receptor **9b** at various concentrations in 9:1 H_2O/D_2O. Yellow reference lines have been added to the spectrum. The receptor is taken to be monomeric below 0.075 mM.

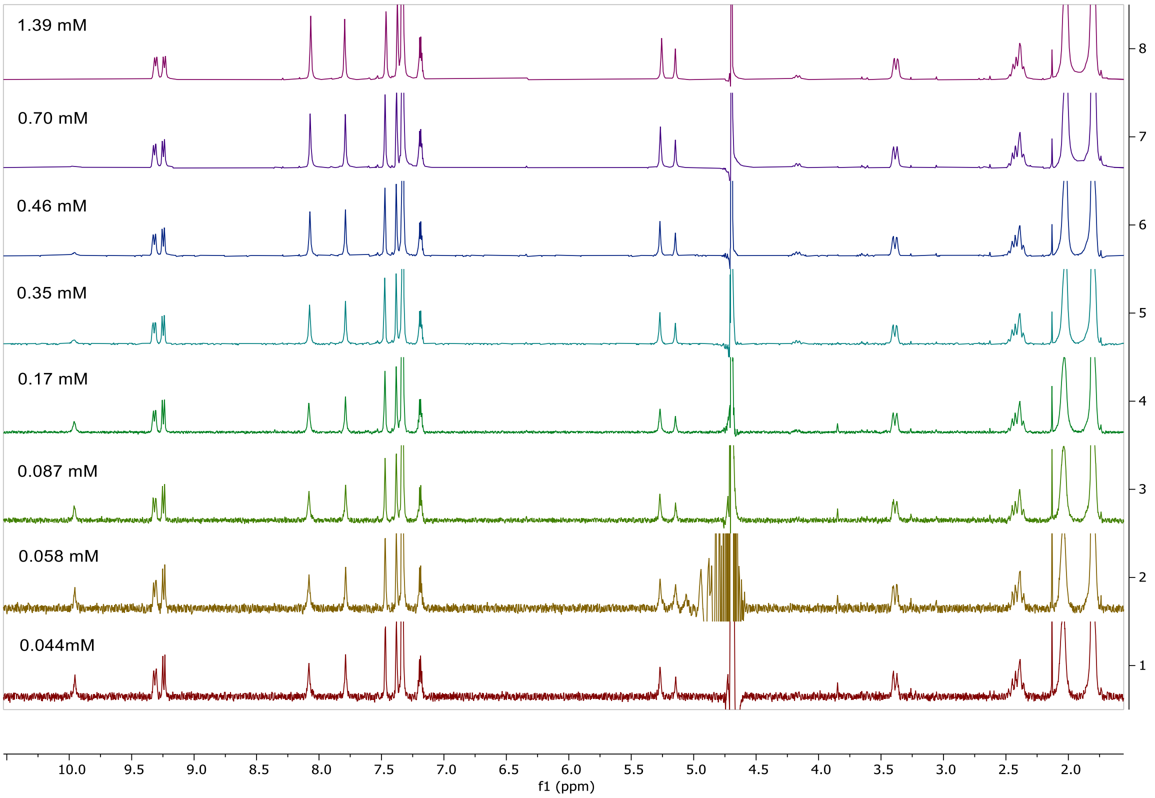

Figure S35. ^{1}H NMR spectra showing receptor **10b** at various concentrations in 9:1 H_2O/D_2O. The receptor is taken to be monomeric below 0.058 mM.

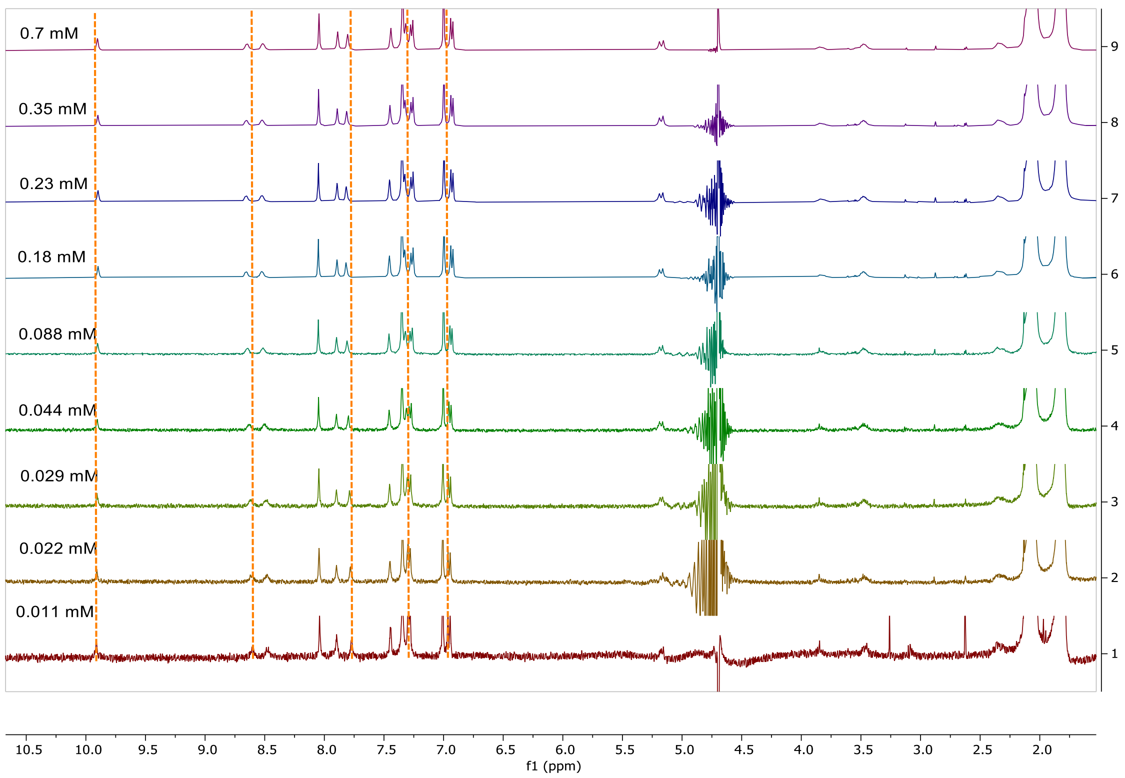

Figure S36. ^{1}H NMR spectra showing receptor **5** at various concentrations in 9:1 H_2O/D_2O (yellow reference lines have been added to the spectrum). The receptor is taken to be monomeric below 0.022 mM.

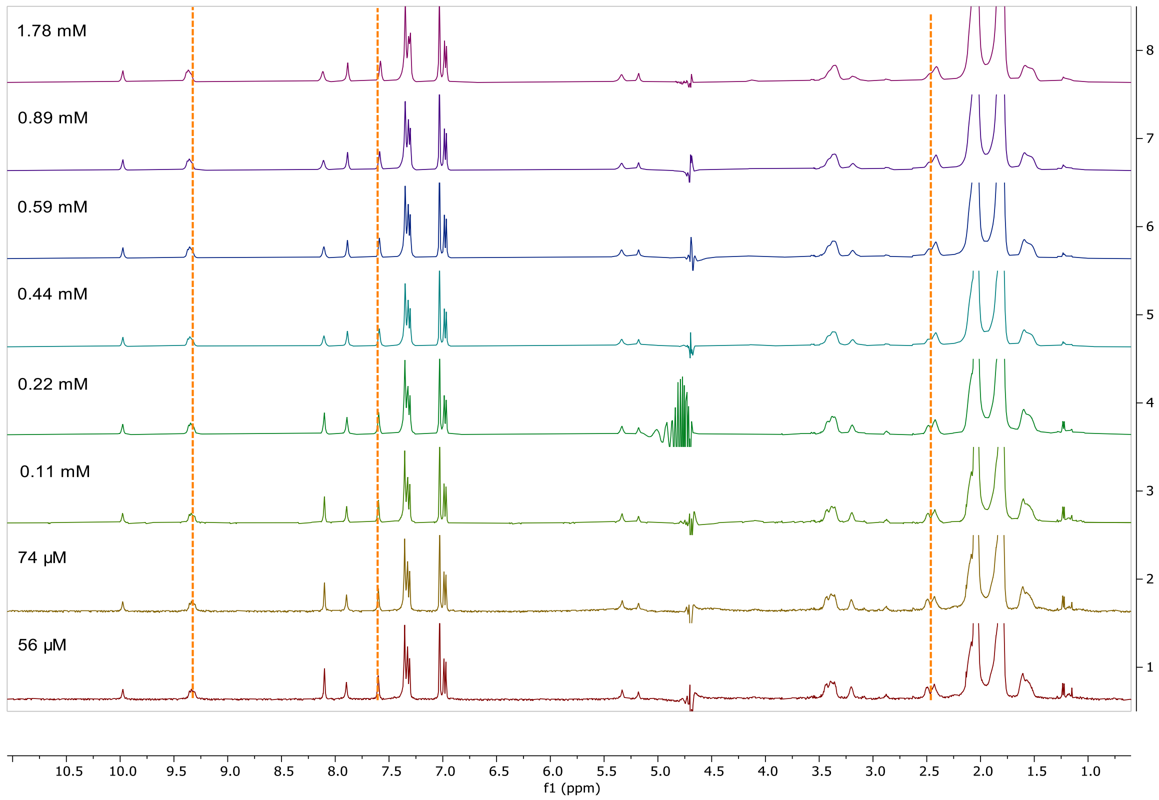

Figure S37. ^{1}H NMR spectra showing receptor **6** at various concentrations in 9:1 H_2O/D_2O (yellow reference lines have been added to the spectrum). The receptor is taken to be monomeric below 0.074 mM.

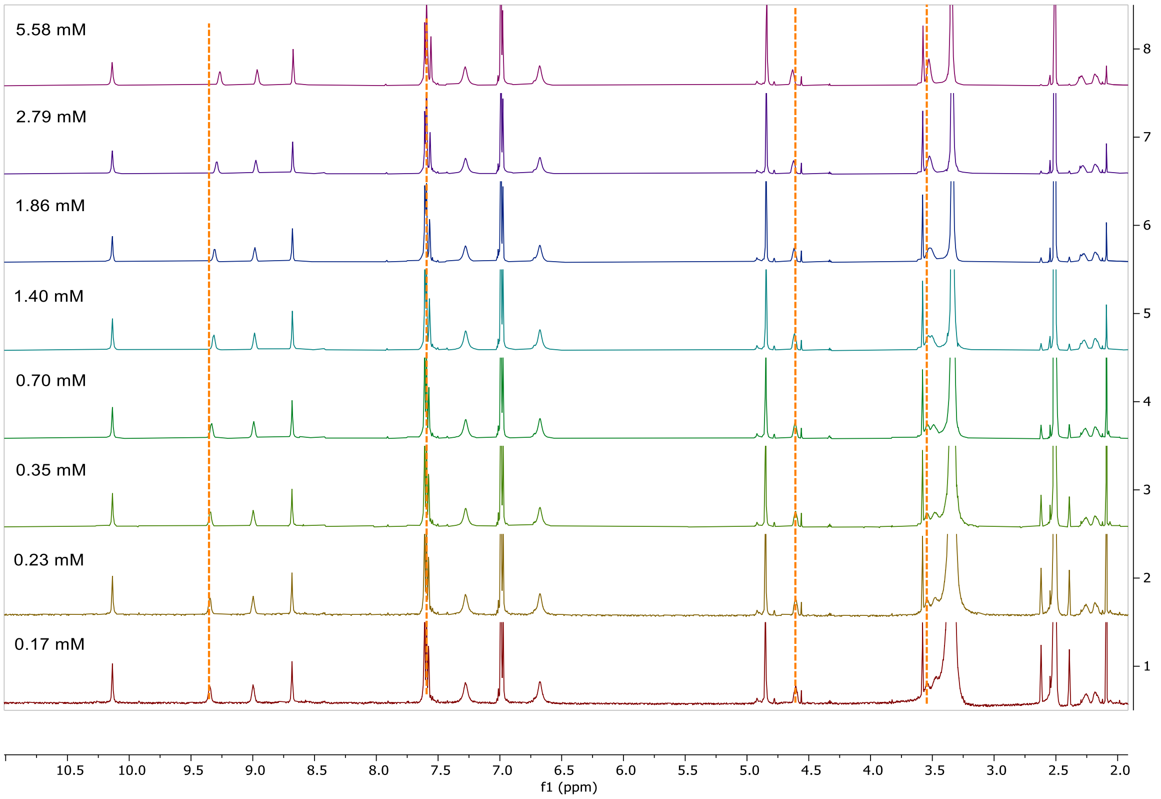

Figure S38. ^{1}H NMR spectra showing receptor **20** at various concentrations in DMSO-d^{6} (yellow reference lines have been added to the spectrum). The receptor is taken to be monomeric below 0.35 mM.

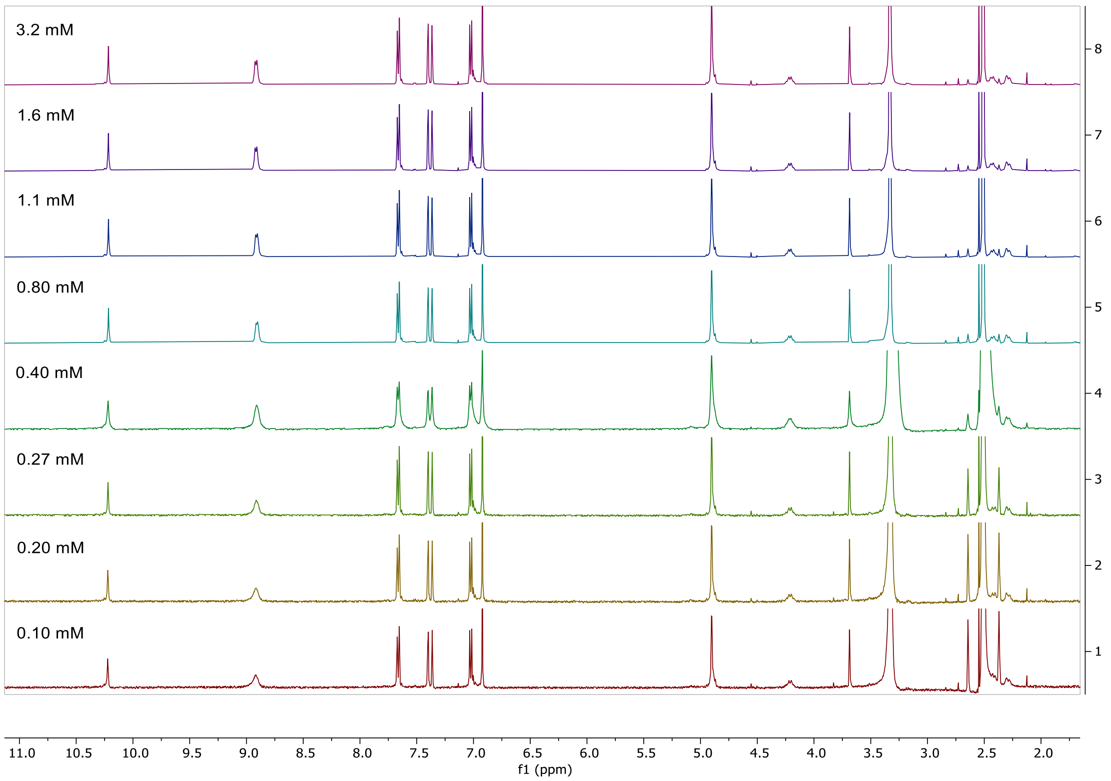

Figure S39. ^{1}H NMR spectra showing receptor **21** at various concentrations in DMSO-*d*[6]. The receptor is taken to be monomeric below 0.40 mM.

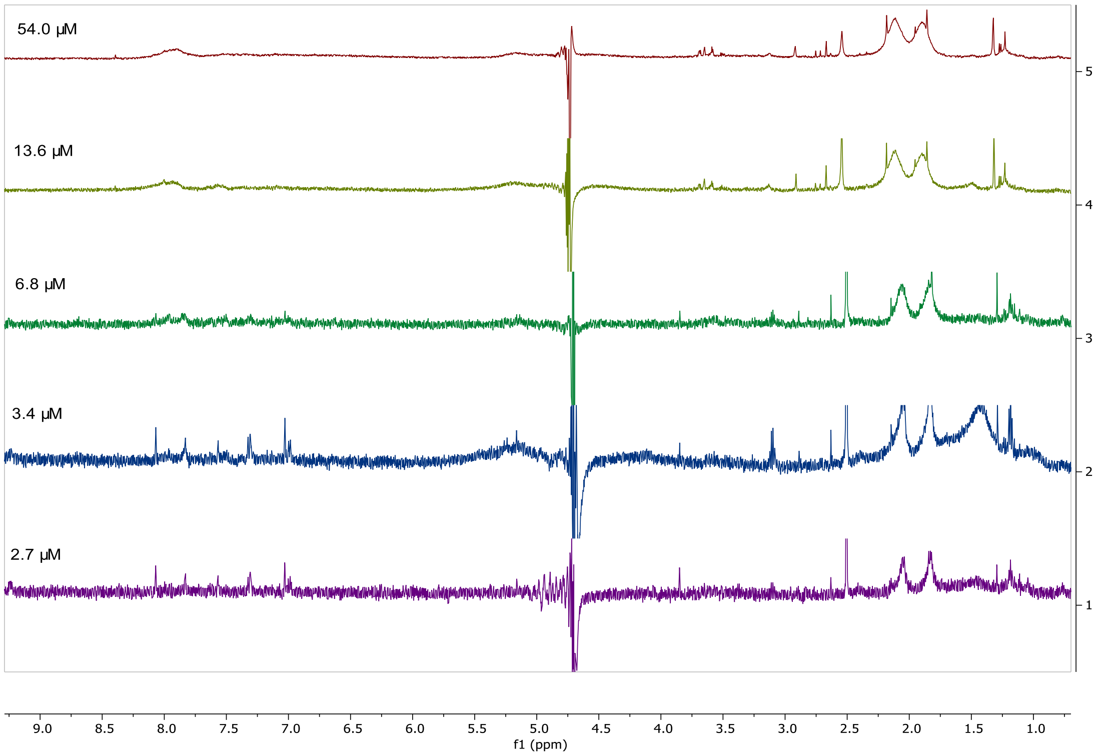

Figure S40. ^{1}H NMR spectra showing receptor **6-G$_1$** at various concentrations in 9:1 H_2O/D_2O. The monomeric concentration is taken to be below below 3.4 μM, which is too low for binding studies by ^{1}H NMR with the equipment available.

1.8 pH titrations of receptors 5 and 6

pH near-neutral solutions of receptors **5** or **6** (21 μM or 22 μM) were made by dissolving the solid receptors into a specific amount to in 9:1 H_2O/D_2O (typically 1000 μL). Solid NaOH was then dissolved in the above receptor solutions to make 10 mM, 100 mM and 1 M NaOH aqueous solution (100 μL each). The pH titration started by transferring the neutral receptor solution (500 μL) into an NMR tube and adding an aliquot of the prepared NaOH solutions. The NMR tube was shaken after the addition and then the ^1H NMR spectrum was acquired at 298 K. After the acquisition, the titration solution was transferred to a vial for pH measurement before putting it back into the same NMR tube for the next addition.

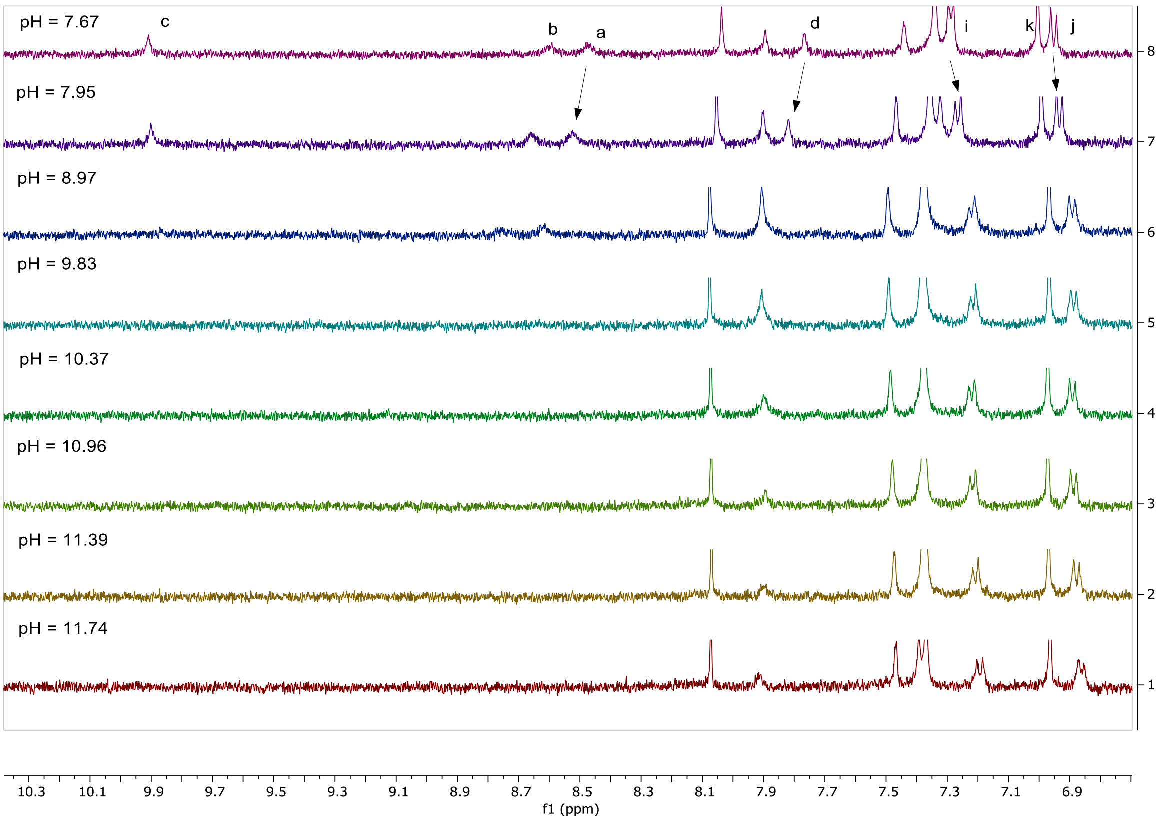

Figure S41. ^1H NMR spectra showing receptor **5** (21 μM) at various pH in 9:1 H_2O/D_2O.

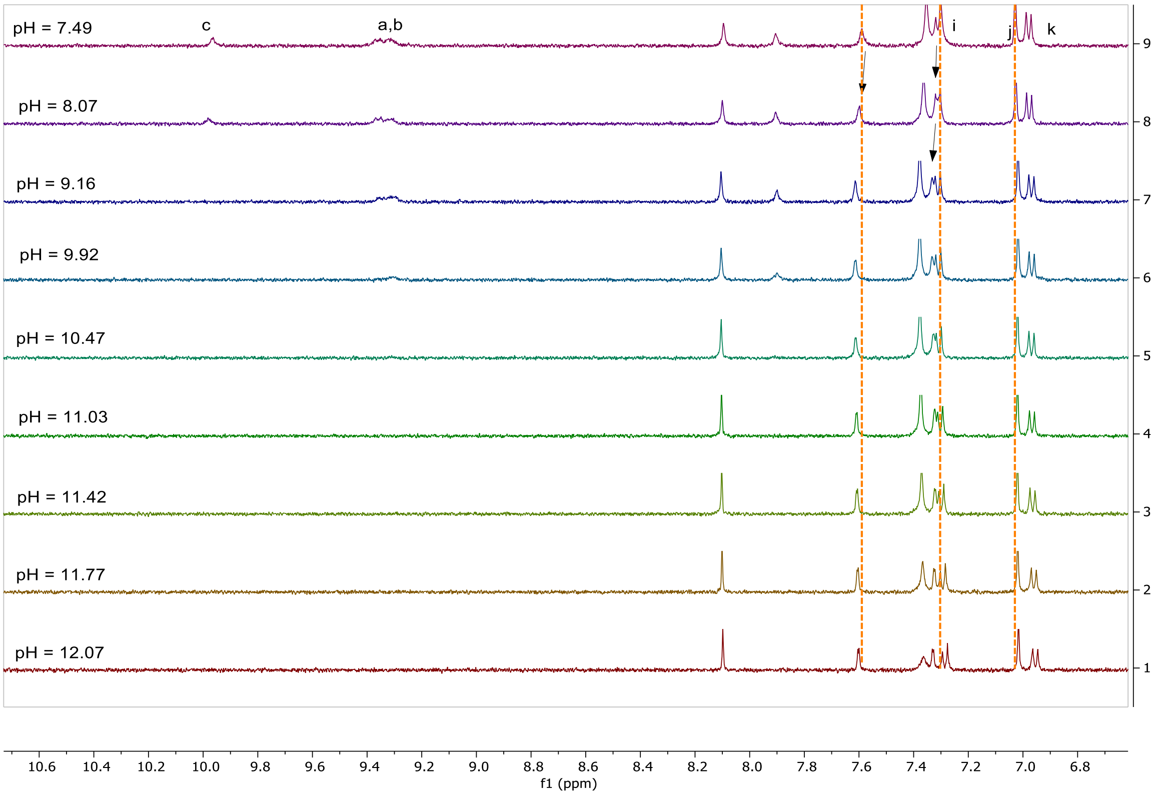

Figure S42. ^{1}H NMR spectra showing receptor **6** (22 μM) at various pH in 9:1 H_2O/D_2O

.

1.9 Receptors with charge-neutral solubilising groups

In addition to receptors with anionic solubilising groups, charge-neutral macrocycles **S13** and **S14** were also prepared and studied during the course of this work. In both cases, ^{1}H NMR dilution studies indicated that aggregation persisted down to concentrations too low for NMR binding studies.

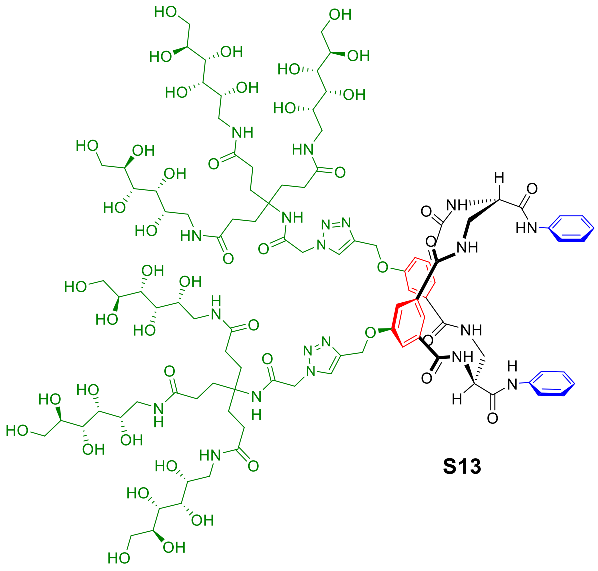

S13

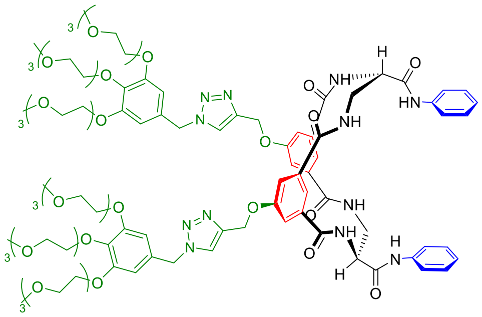

S14

2. Binding studies

2.1 Nuclear Magnetic Resonance (NMR) titrations

^{1}H NMR titrations were performed on 600 MHz Bruker Neo Cryo and 500 MHz Bruker Avance III HD (equipped with a Proton optimised 5mm TCI Prodigy probe) spectrometers. ^{1}H NMR spectra in water were obtained through the acquisition of 48 to 64 scans using an Excitation Sculpting Water Suppression method.

For titrations in organic solvent (DMSO-d^6), solutions of receptor at known concentrations were prepared in an NMR tube. Aliquots of a solution of receptor at the same concentration and guest were added. The receptor concentration was therefore held constant while the guest concentration was increased. The NMR tube was shaken after each addition and the ^{1}H NMR spectra acquired at 298 K.

Titrations in aqueous medium were conducted at a constant near-neutral pH in D_2O or 9:1 H_2O/D_2O. The host stock solution was first prepared by dissolving a weighed amount of host solid in D_2O or 9:1 H_2O/D_2O and the pH was checked to be near-neutral (the pH ranged from 7.35 to 7.50 for different hosts). For carboxylate guests, the guest stock solution was then prepared by dissolving sodium carboxylate salt in water and adjusting pH to match the host solution as closely as possible ($pH_{guest} = pH_{host} \pm 0.15$) by adding the conjugate acid. Other guest (NaCl, *L*- and *D*-Ala, *L*-Ala-*L*-Ala) stock solutions were prepared by dissolving the compound in water and confirming the neutrality of pH. Before each aqueous binding experiment, two solutions for titration were prepared using the above near-neutral stock solutions. The first consisted of 550 µL of host solution at a concentration below the association threshold (ranging from 10 µM to 133 µM for different hosts). The second solution consisted of 500 µL of host, at the same concentration as the first solution, mixed with guest (50 mM to 500 mM). Both solutions were made in 1.5 mL vials, and the near-neutral pH was confirmed (ranging from 7.30 to 7.65 for different host and host-guest solutions). During the titration, the host solution (500 µL) was transferred to an NMR tube and aliquots of host-guest mixture were added precisely using Gilson pipettes. This method holds the receptor concentration constant while the guest concentration was increased, with the pH staying near-neutral. The NMR tube was shaken after each addition and the ^{1}H NMR spectra acquired at 298 K. At the end of each titration, the solution in the NMR tube was transferred into a vial and the pH was measured again confirming negligible changes ($\Delta pH < \pm 0.1$) during the experiment.

Association constants were determined by monitoring the change in chemical shift ($\Delta\delta$) for selected protons belonging to the receptor species. The $\Delta\delta$ values were analysed to give binding constants K_a using Bindfit[5], employing the 1:1 binding model for monomeric receptors **8** – **10** and the 1:2 non-cooperative binding models for dimeric receptors **20**, **21**, **5** and **6**.

Receptor 8a & TBA acetate (DMSO-d^6)

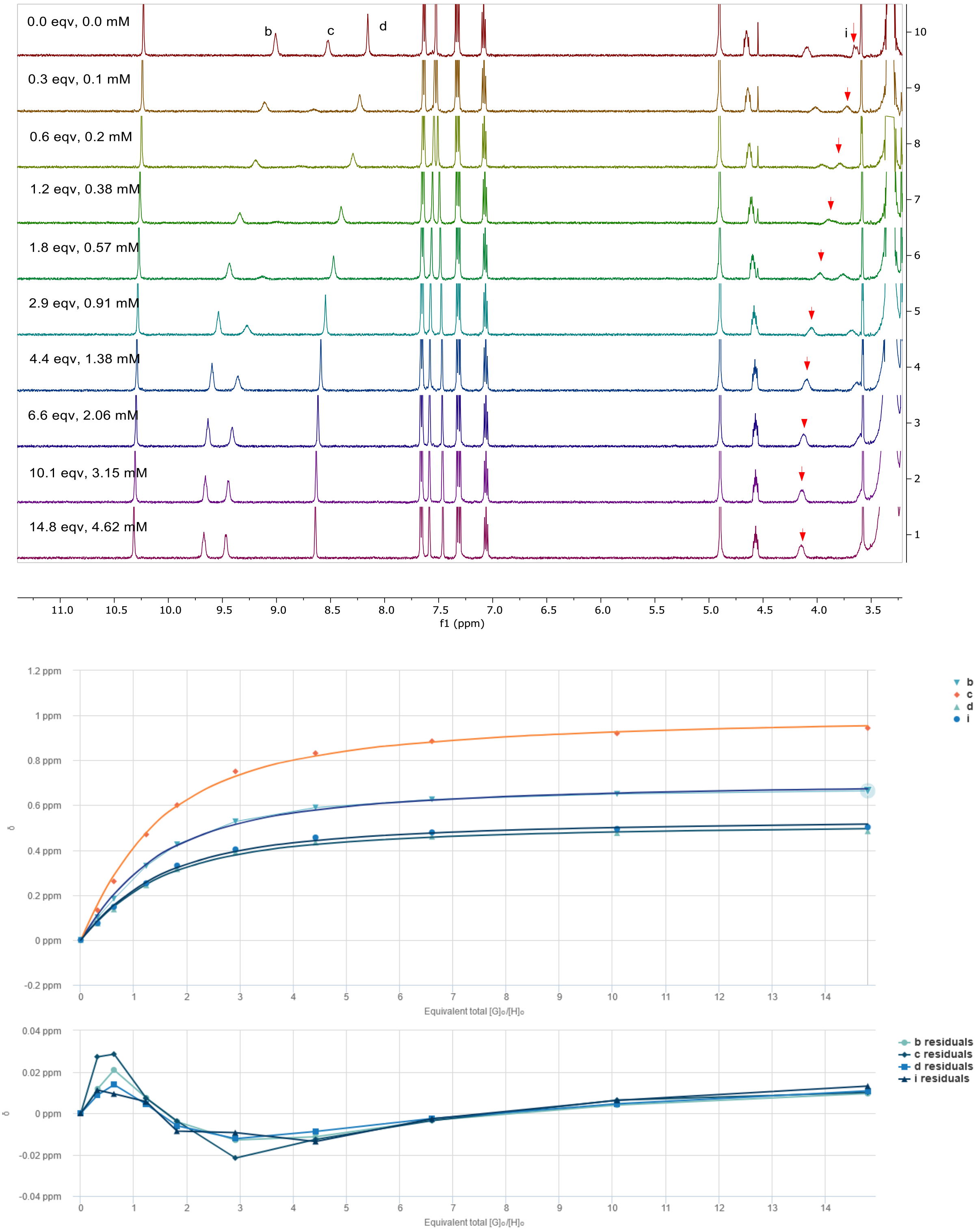

Figure S43. Top). ^{1}H NMR spectra (500 MHz, DMSO-d^6) for receptor **8a** (0.31 mM) titrated with a combined solution of tetrabutylammonium acetate (20 mM) and receptor **8a** (0.31 mM). The equivalents and concentrations of guest added are listed in the graph. Bottom). Global fitting of the binding isotherms (protons b, c, d and i) from Bindfit to a 1:1 model K_a = 3759 M^{-1} (± 4.0 %). Full fitted data is available online at: http://app.supramolecular.org/bindfit/view/33a29e26-f8dc-47dd-9593-130fa22860e7

Receptor 8a & TBA chloride (DMSO-d^6)

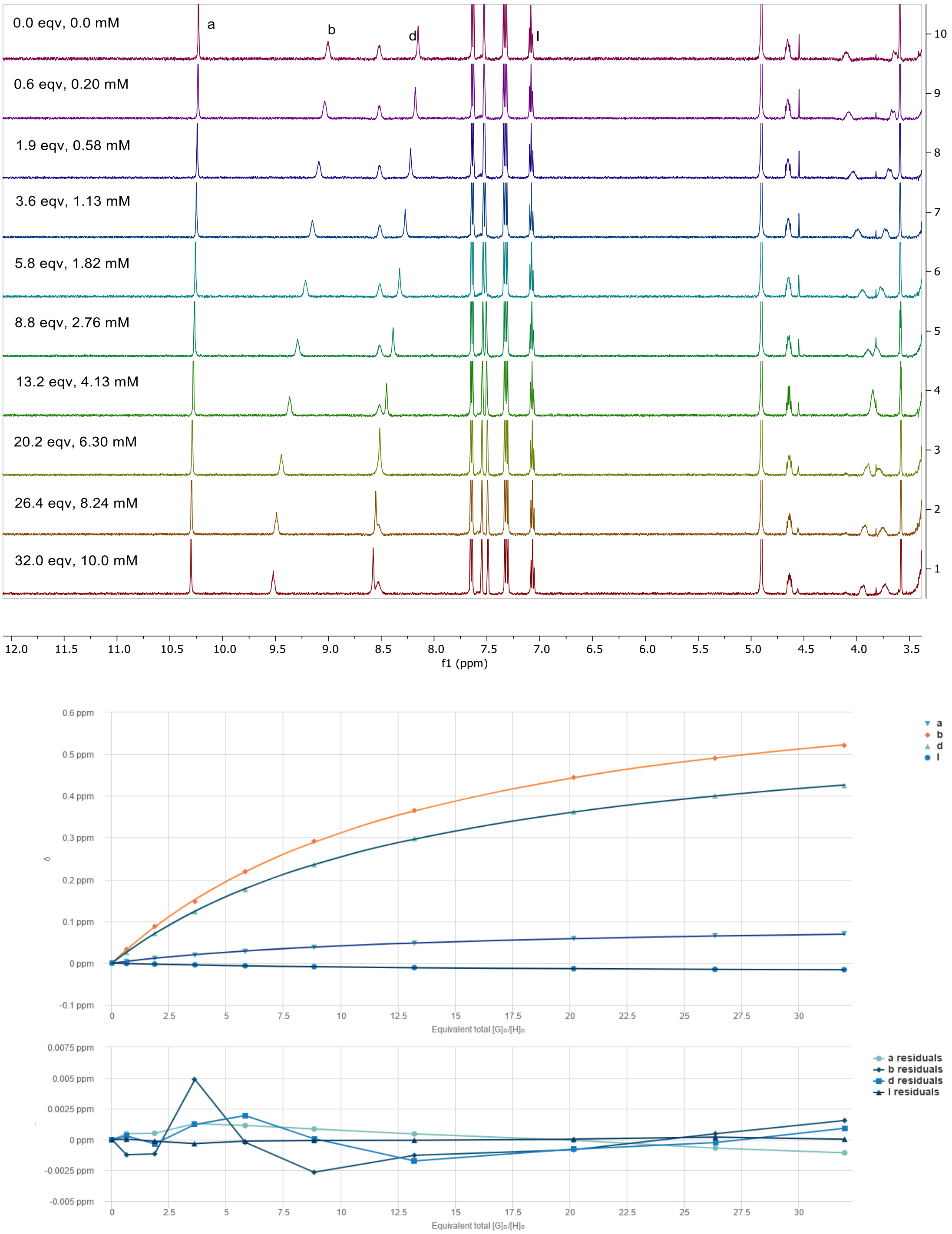

Figure S44. Top). ^{1}H NMR spectra (500 MHz, DMSO-d^6) for receptor **8a** (0.31 mM) titrated with a combined solution of tetrabutylammonium chloride (20 mM) and receptor **8a** (0.31 mM). The equivalents and concentrations of guest added are listed in the graph. Bottom). Global fitting of the binding isotherms (protons a, b, d and l) from Bindfit to a 1:1 model K_a = 240 M^{-1} (± 0.5 %). Full fitted data is available online at: http://app.supramolecular.org/bindfit/view/bc5f579a-5564-48df-bf4a-105830f6d889

Receptor 8b & chloride (H_2O/D_2O)

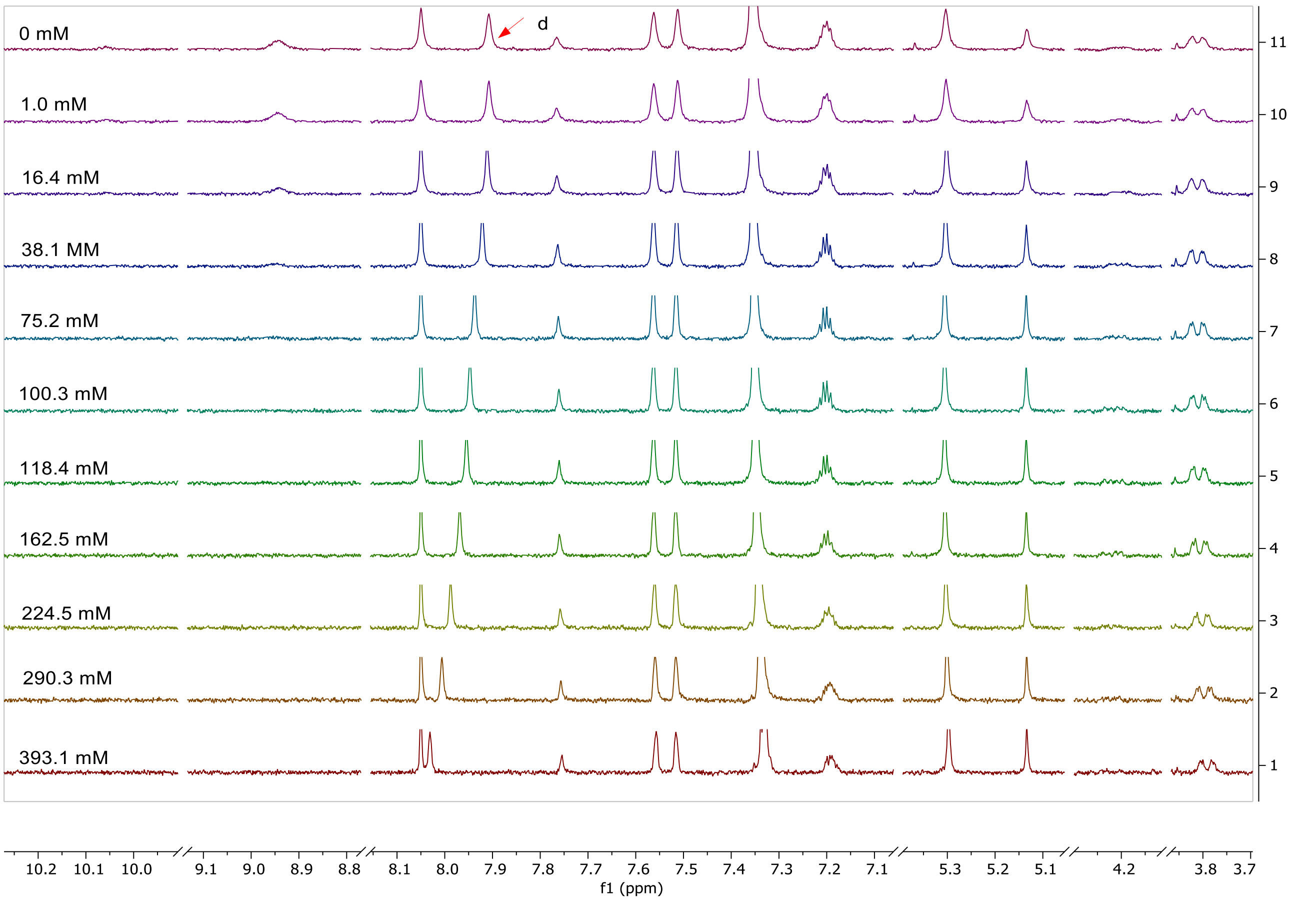

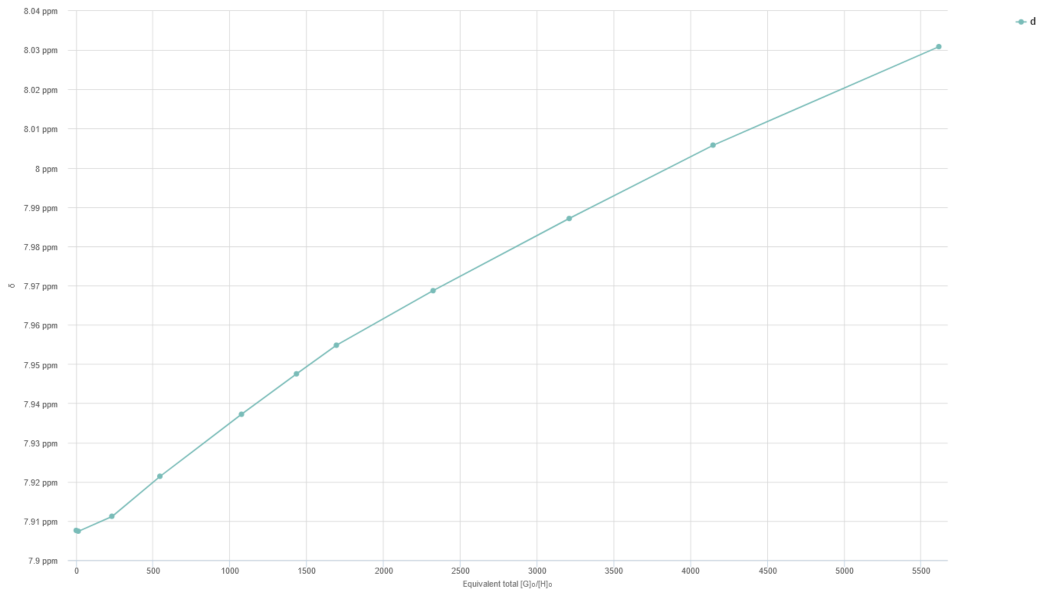

Figure S45. Top). ^{1}H NMR spectra (600 MHz, 1:1 H_2O/D_2O) for receptor **8b** (0.07 mM) titrated with a combined solution of sodium chloride (800 mM) and receptor **8b** (0.07 mM). The equivalents and concentrations of guest added are listed in the graph. pH = 7.60 at the end of titration. Bottom) The chemical shift changes from proton d which cannot be fitted into any model.

Receptor 9a & TBA acetate (DMSO-d^6)

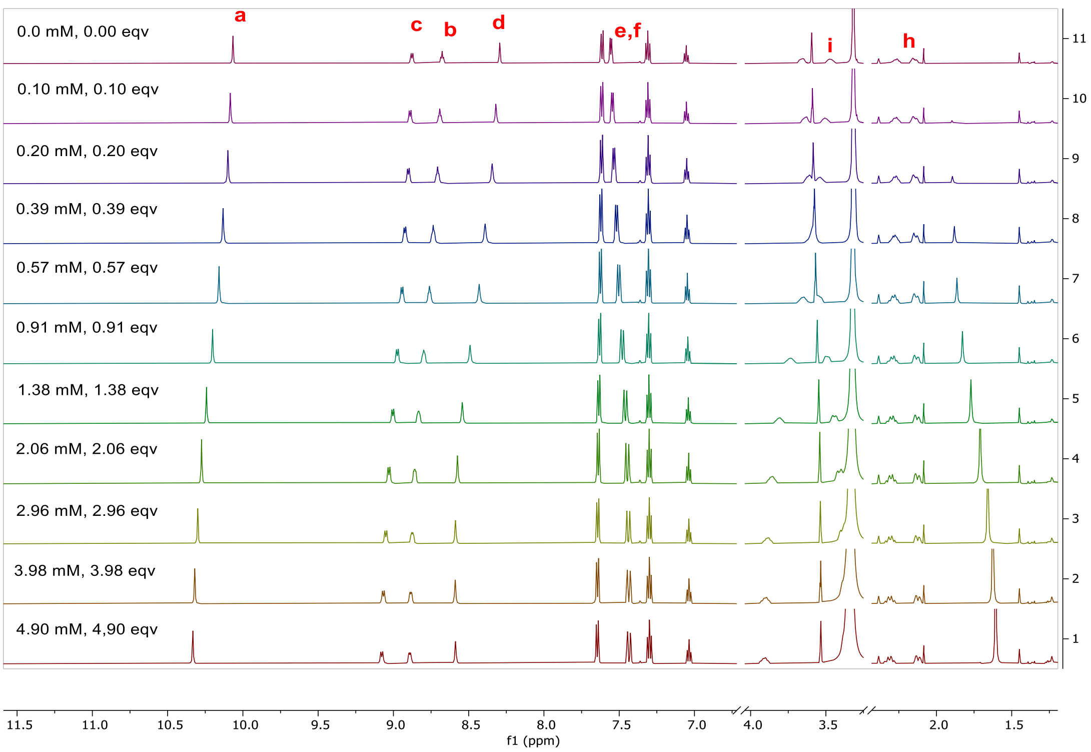

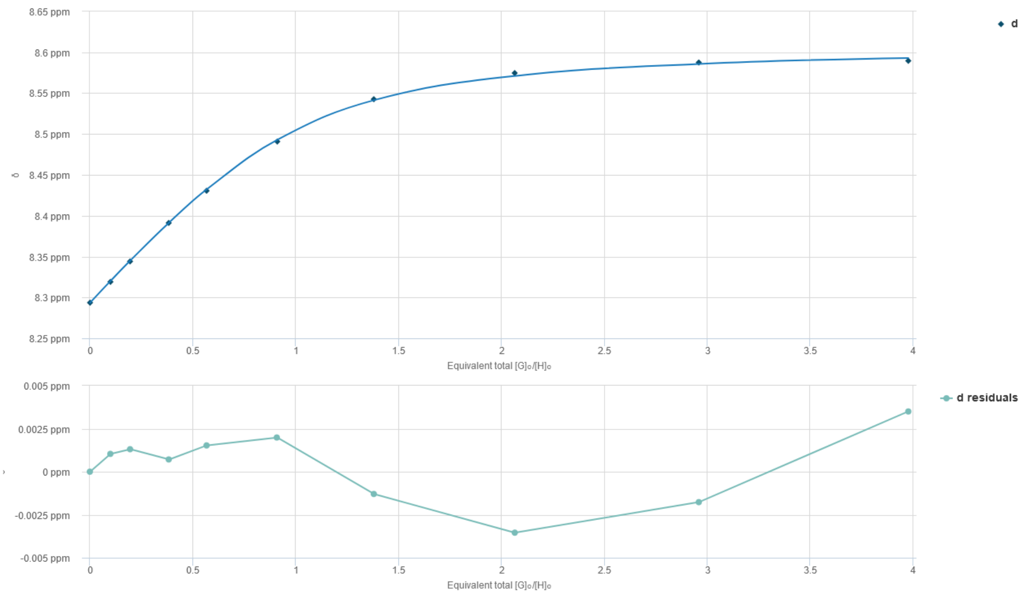

Figure S46. Top). ^{1}H NMR spectra (600 MHz, DMSO-d^6) for receptor **9a** (1 mM) titrated with a combined solution of tetrabutylammonium acetate (10 mM) and receptor **9a** (1 mM). The equivalents and concentrations of guest added are listed in the graph; Bottom). Fitting of the binding isotherm (proton d) in Bindfit to a 1:1 model K_a = 6173 M^{-1} (± 7.1 %). Full fitted data is available online at: http://app.supramolecular.org/bindfit/view/11429b47-ec19-4284-be58-35df7f8fb354

Receptor 9a & TBA chloride (DMSO-d^6)

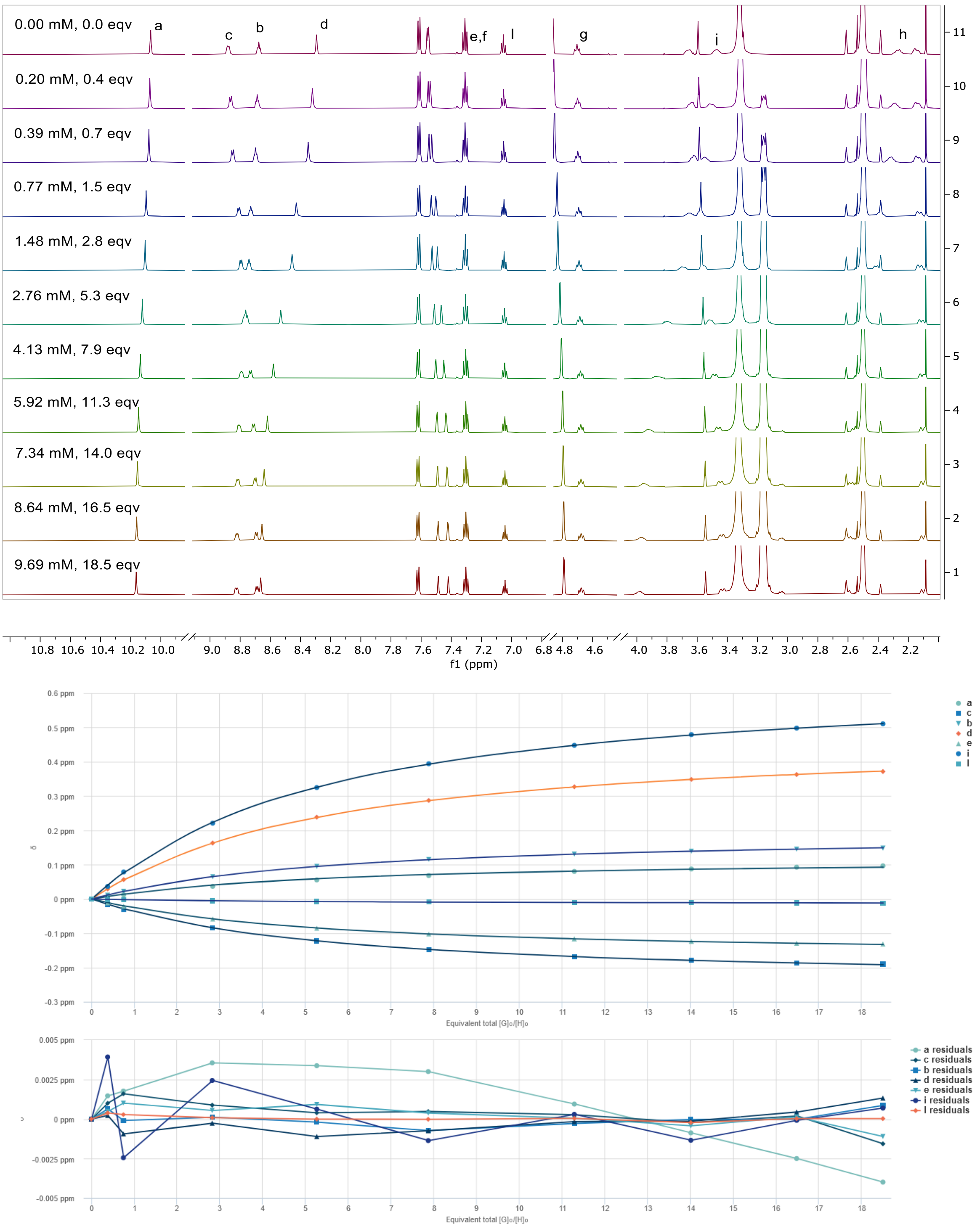

Figure S47. Top) ^{1}H NMR spectra (600 MHz, DMSO-d^6) for receptor **9a** (524 μM) titrated with a combined solution of tetrabutylammonium chloride (20 mM) and receptor **9a** (524 μM). The equivalents and concentrations of guest added are listed in the graph. Bottom). Global fitting of the binding isotherms (protons a, c, b, d, e, i and o) from Bindfit to a 1:1 model K_a = 405.2 M^{-1} (± 0.56 %). Full fitted data is available online at: http://app.supramolecular.org/bindfit/view/bdda8616-dcbf-46ca-8823-90cb8373d105

Receptor 9b & acetate (H_2O/D_2O)

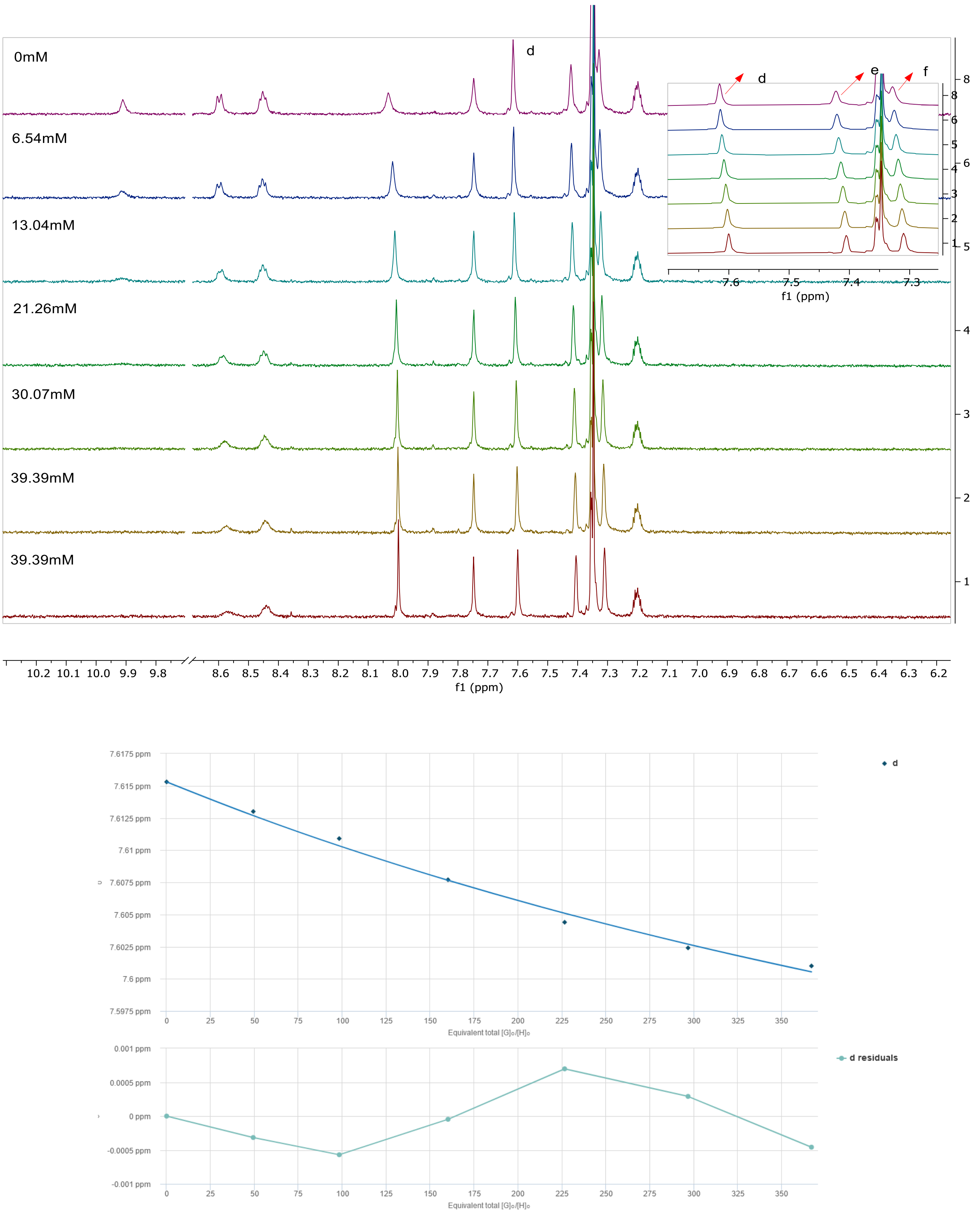

Figure S48. Top) ^{1}H NMR (600 MHz, 9:1 H_2O/D_2O) spectra for receptor **9b** (133 μM) titrated with a combined solution of sodium acetate (100 mM) and receptor **9b** (133 μM). The concentration of guest is shown on each spectrum. pH = 7.53 at the end of titration. Bottom) Fitting of the binding isotherm (proton d) from Bindfit to a 1:1 model K_a = 8.0 M^{-1} (± 7.6 %). Full fitted data is available online at: http://app.supramolecular.org/bindfit/view/62ac903b-f59f-4555-be81-15928ad94088

Receptor 9b & chloride (H_2O/D_2O)

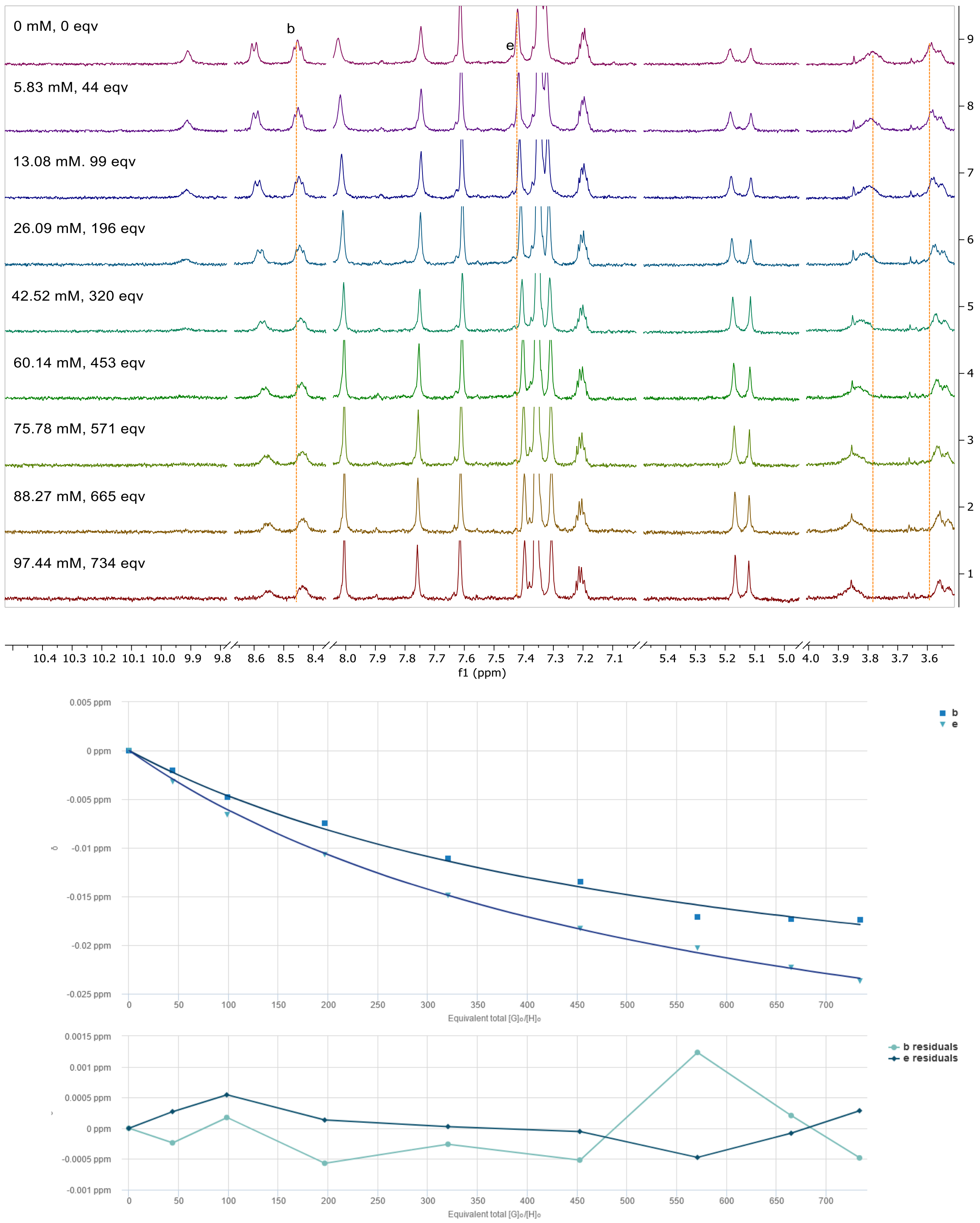

Figure S49. Top). ^{1}H NMR spectra (600 MHz, 9:1 H_2O/D_2O) for receptor **9b** (133 μM) titrated with a combined solution of sodium chloride (200 mM) and receptor **9b** (133 μM). The equivalents and concentrations of guest added are listed in the graph. pH = 7.56 at the end of titration. Bottom). Global fitting of the binding isotherms (protons b, e) from Bindfit to a 1:1 model K_a = 12.85 M^{-1} (± 3.26 %). Full fitted data is available online at: http://app.supramolecular.org/bindfit/view/0c39573d-f3c2-4f73-9260-8c1356858a77

Receptor 10a & TBA acetate (DMSO-d^6)

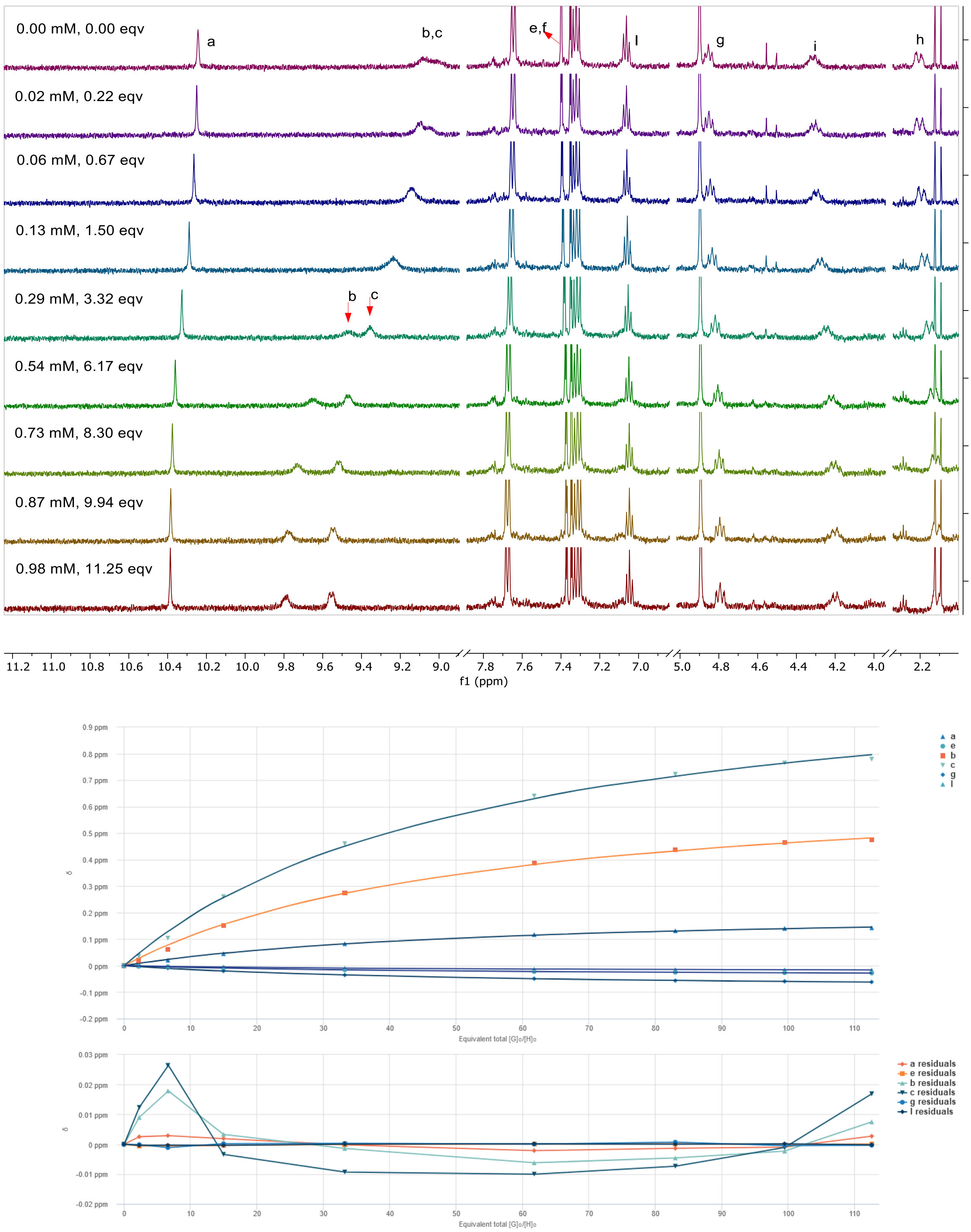

Figure S50. Top) ^{1}H NMR spectra (500 MHz, DMSO-d^6) for receptor **10a** (87.5 μM) titrated with a combined solution of tetrabutylammonium acetate (2 mM) and receptor **10a** (87.5 μM). The equivalents and concentrations of guest added are listed in the graph. Bottom) Global fitting of the binding isotherms (protons a, b, c, e, g and l) from Bindfit to a 1:1 model K_a = 2192 M^{-1} (± 1.7 %). Full fitted data is available online at: http://app.supramolecular.org/bindfit/view/c2a3e941-7f5e-4529-b500-e8893345559f

Receptor 10a & TBA chloride (DMSO-d^6)

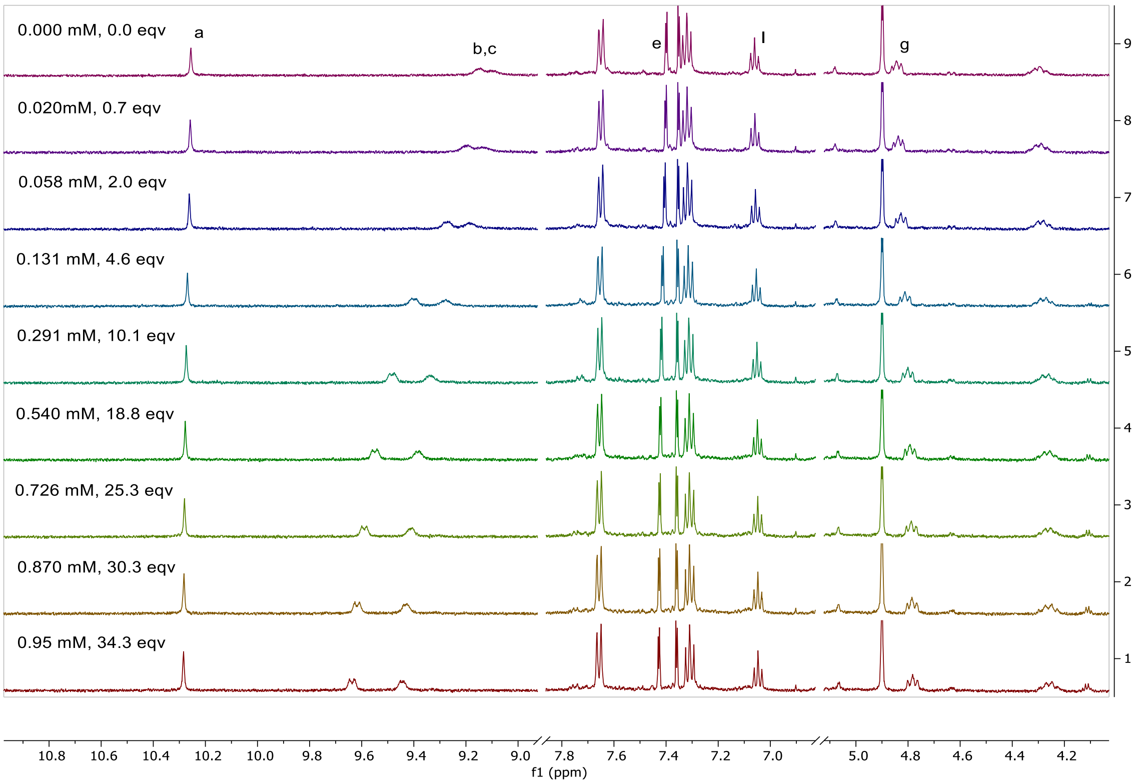

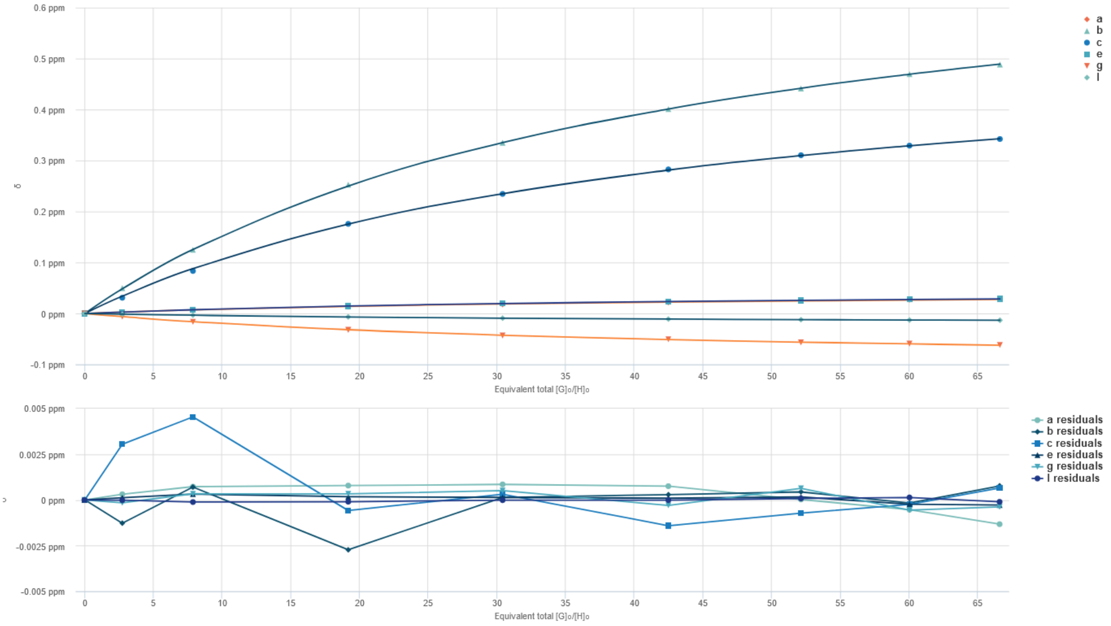

Figure S51. Top) ^{1}H NMR spectra (500 MHz, DMSO-d^6) for receptor **10a** (143 μM) titrated with a combined solution of tetrabutylammonium chloride (20 mM) and receptor **10a**(143 μM). The equivalents and concentrations of guest added are listed in the graph. Bottom) Global fitting of the binding isotherms (protons a, c, b, e, g and l) from Bindfit to a 1:1 model K_a = 169.7 M^{-1} (± 0.36 %). Full fitted data is available online at: http://app.supramolecular.org/bindfit/view/2ae8d51d-e58d-4510-a724-f75950f64405

Receptor 10b & acetate (H_2O/D_2O)

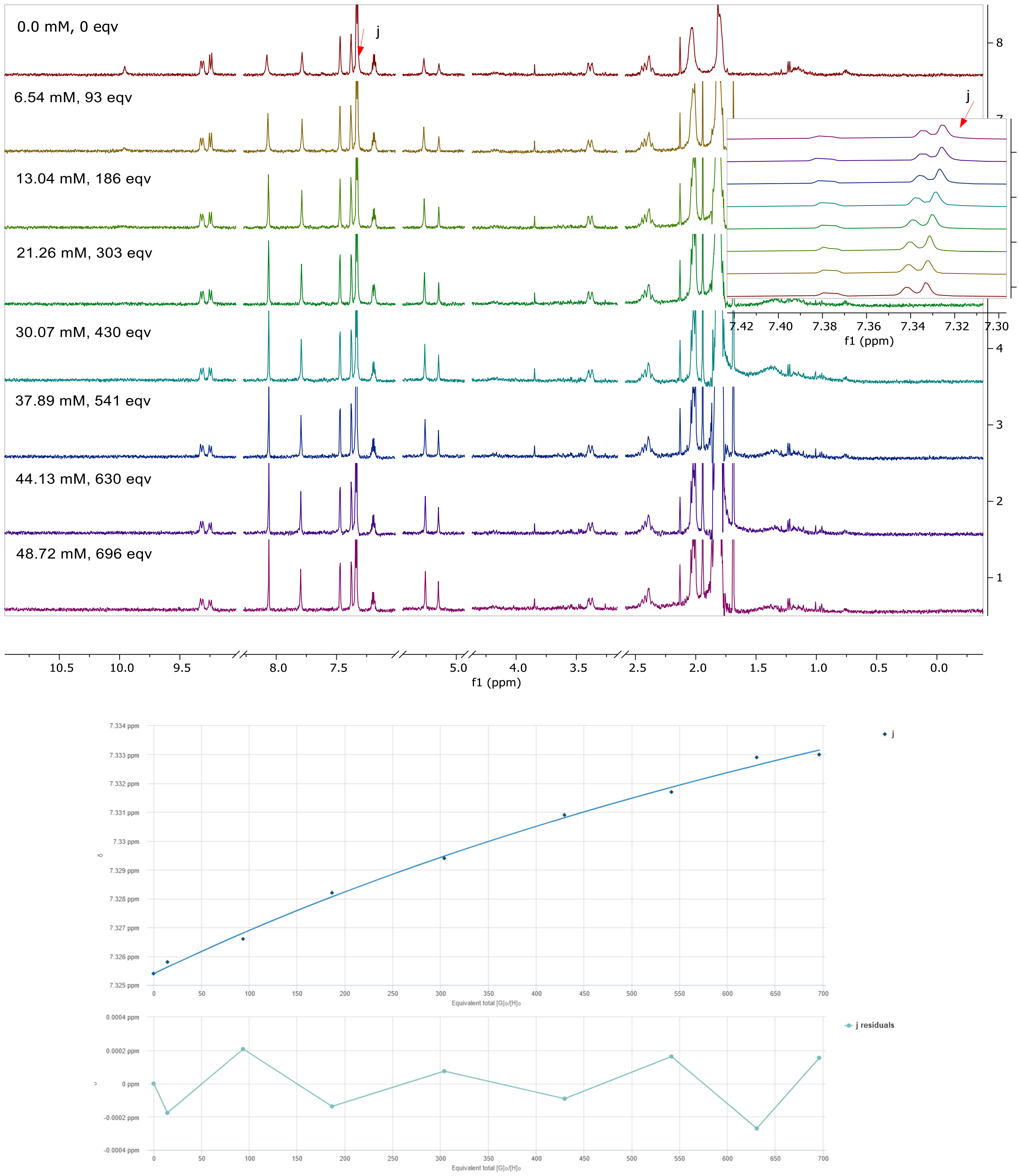

Figure S52. Top) ^{1}H NMR (500 MHz, 9:1 H_2O/D_2O) spectra for receptor **10b** (66.7 μM) titrated with a combined solution of sodium acetate (100 mM) and receptor **10b** (66.7 μM). pH = 7.60 at the end of titration. Bottom). Fitting of the binding isotherm (proton j) from Bindfit to a 1:1 model K_a = 8.84 M^{-1} (± 4.28 %). Full fitted data is available online at: http://app.supramolecular.org/bindfit/view/465327ae-6c72-4438-a821-538ef5ed5f58

Receptor 10b & chloride (H_2O/D_2O)

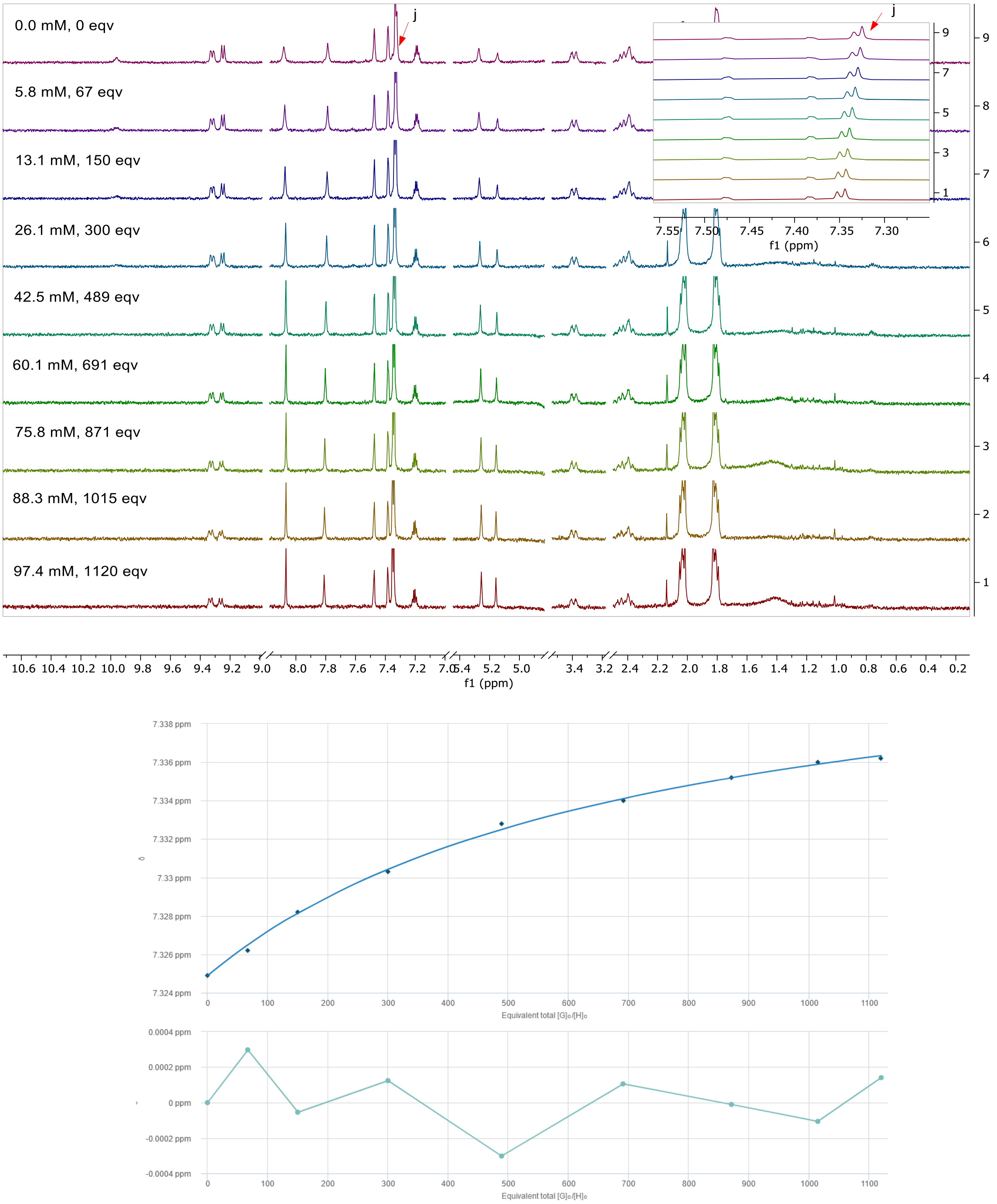

Figure S53. Top) ^{1}H NMR (500 MHz, 9:1 H_2O/D_2O) spectra for receptor **10b** (66.7 μM) titrated with a combined solution of sodium chloride (100 mM) and receptor **10b** (66.7 μM). The concentration of guest is shown on each spectrum. pH = 7.47 at the end of titration.Bottom) Fitting of the binding isotherm (proton j) from Bindfit to a 1:1 model K_a = 15.97 M^{-1} (± 4.16 %). Full fitted data is available online at: http://app.supramolecular.org/bindfit/view/1647bddf-5737-4dac-b08d-eea0da2c309b

Receptor 20 & TBA acetate (DMSO-d^6)

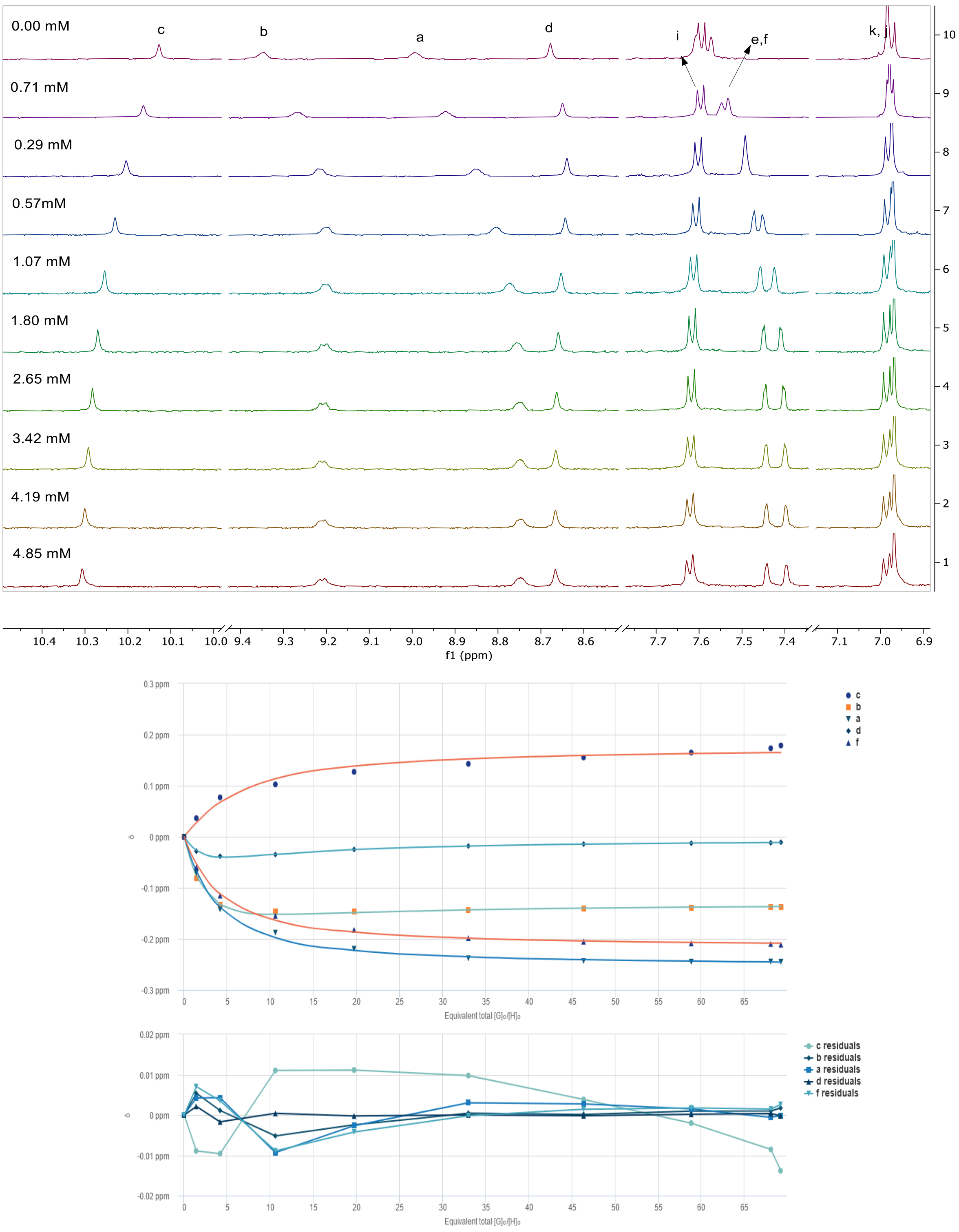

Figure S54. Top) ^{1}H NMR (600 MHz, DMSO-d^6) spectra for receptor **20** (70 μM) titrated with a combined solution of tetrabutylammonium acetate (10 mM) and receptor **20** (70 μM). The concentration of guest is shown on each spectrum. Bottom) Global fitting of the binding isotherms (protons a, b, c, d, f) from Bindfit to a 1:2 non-cooperative model K_a = 7564.4 M^{-1} (± 7.4 %). Full fitted data is available online at: http://app.supramolecular.org/bindfit/view/90113f79-6294-4d3b-aad1-e8f96f95ef06

Receptor 20 & TBA benzoate (DMSO-d^6)

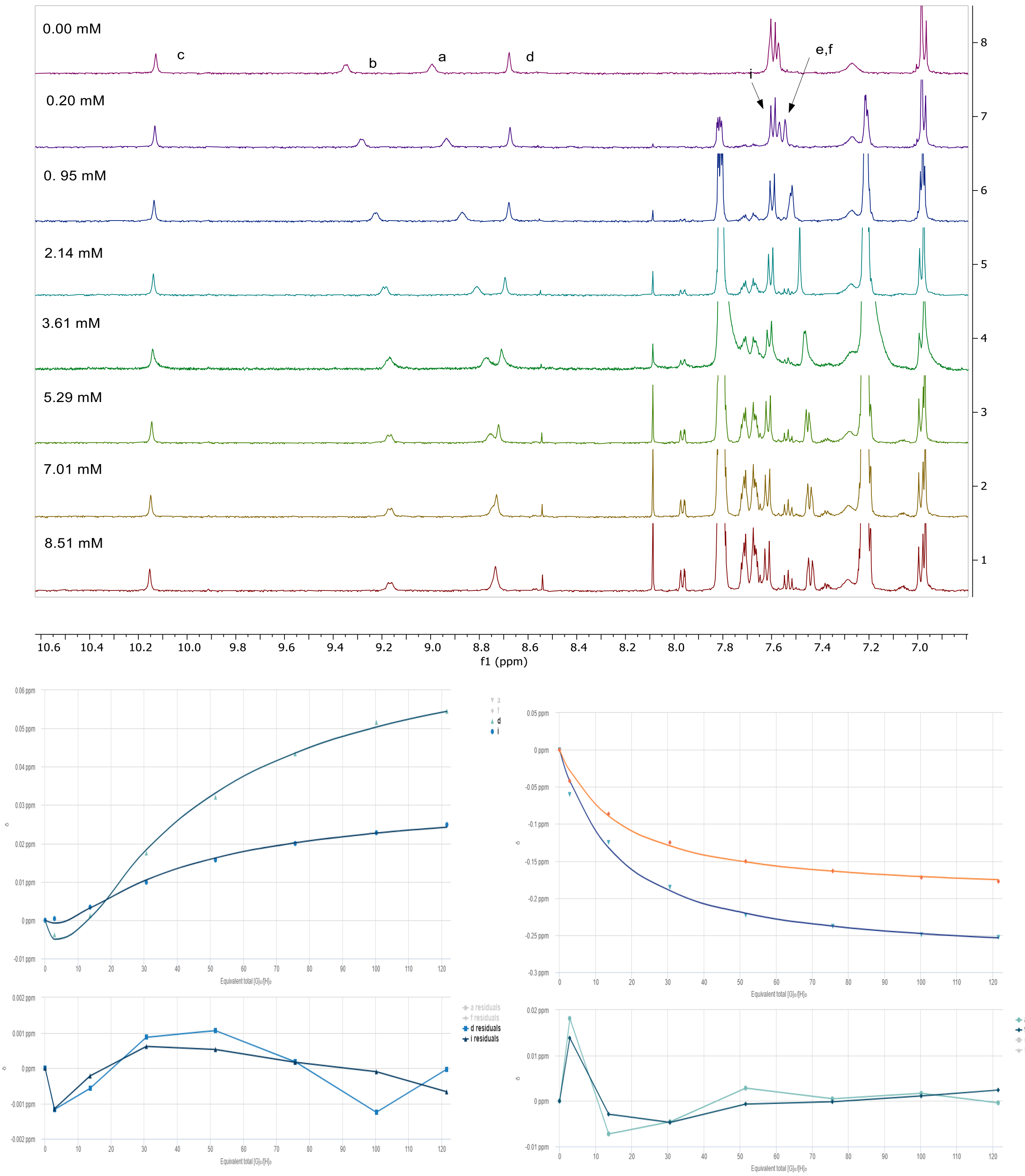

Figure S55. Top) ^{1}H NMR (500 MHz, DMSO-d^6) spectra for receptor **20** (70 µM) titrated with a combined solution of tetrabutylammonium benzoate (14.4 mM) and receptor **20** (70 µM). The concentration of guest is shown on each spectrum. Bottom) Global fitting of the binding isotherms (protons a, d, i and f) from Bindfit to a 1:2 non-cooperative model K_a = 1650.2 M^{-1} (± 6.9 %). Full fitted data is available online at: http://app.supramolecular.org/bindfit/view/3bf198b5-89aa-4952-91b4-4ac1e54f20ef

Receptor 20 & TBA chloride (DMSO-d^6)

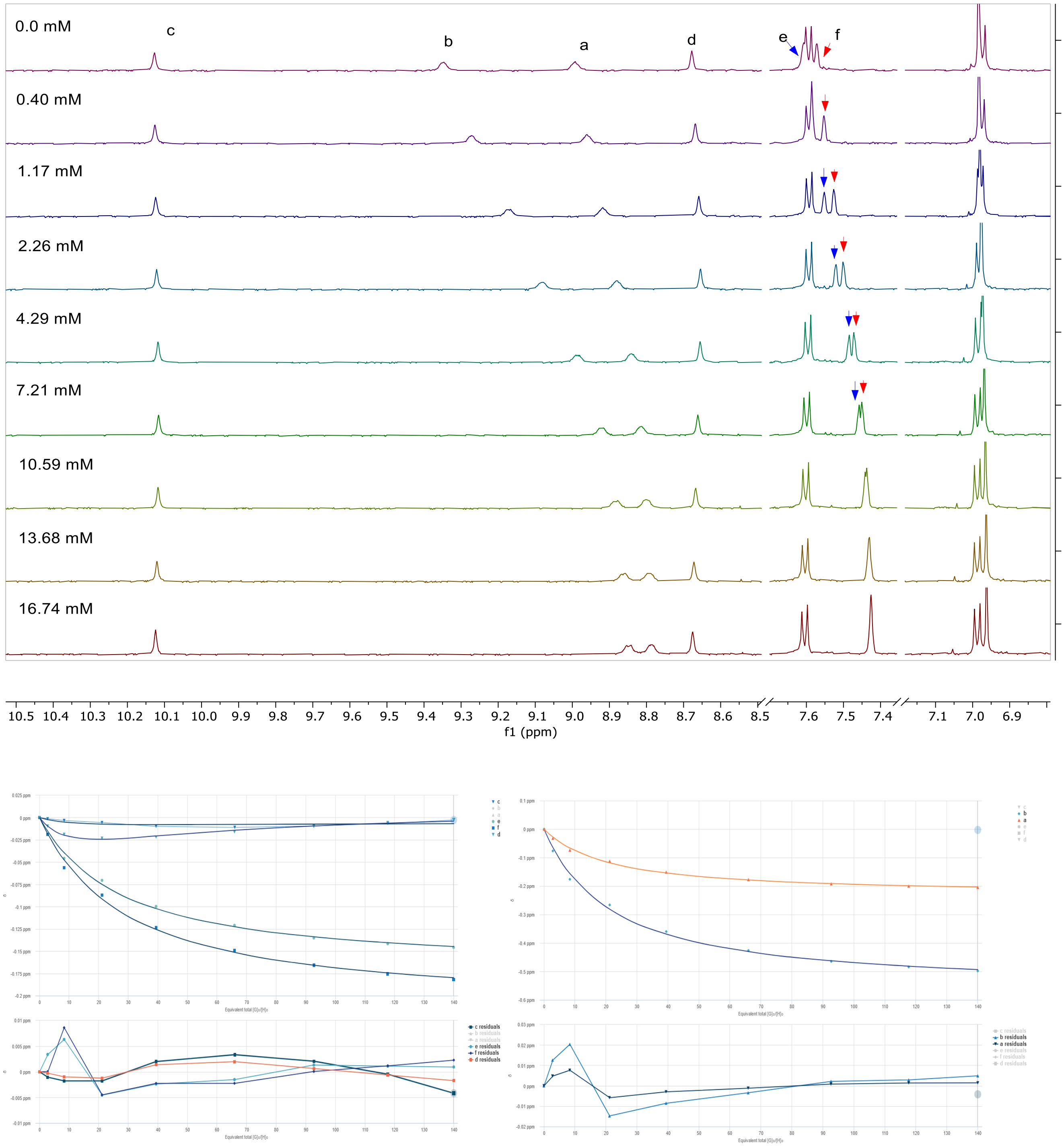

Figure S56. Top) ^{1}H NMR (600 MHz, DMSO-d^6) spectra for receptor **20** (70 μM) titrated with a combined solution of TBA chloride (40 mM) and receptor **20** (70 μM). The concentration of guest is shown on each spectrum. Bottom) Global fitting of the binding isotherms (protons a, b, c, d, e and f) from Bindfit to a 1:2 non-cooperative model K_a = 1241.3 M^{-1} (± 3.5 %). Full fitted data is available online at:
http://app.supramolecular.org/bindfit/view/aef14313-31d8-4dde-9c31-b34823ed8900

Receptor 21 & TBA acetate (DMSO-d^6)

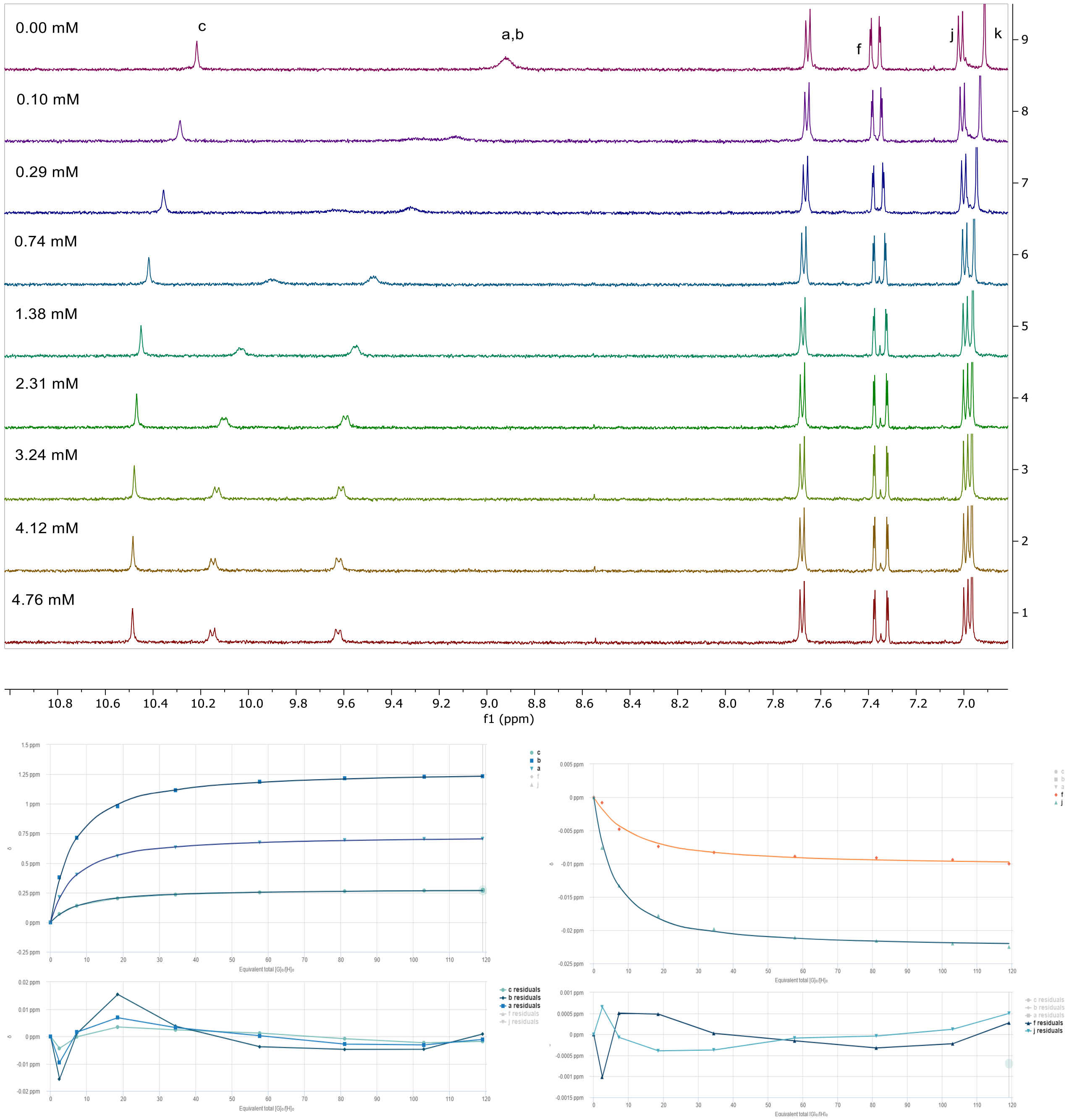

Figure S57. Top) ^{1}H NMR (500 MHz, DMSO-d^6) spectra for receptor **21** (40 µM) titrated with a combined solution of TBA Acetate (10 mM) and receptor **21** (40 µM). The concentration of guest is shown on each spectrum. Bottom) Global fitting of the binding isotherms (protons a, b, c, f, j and k) from Bindfit to a 1:2 non-cooperative model K_a = 8354.8 M^{-1} (± 1.6 %). Full fitted data is available online at:
http://app.supramolecular.org/bindfit/view/f85458bb-d149-47b4-ad6c-7beabe15d1fe

Receptor 21 & TBA benzoate (DMSO-d^6)

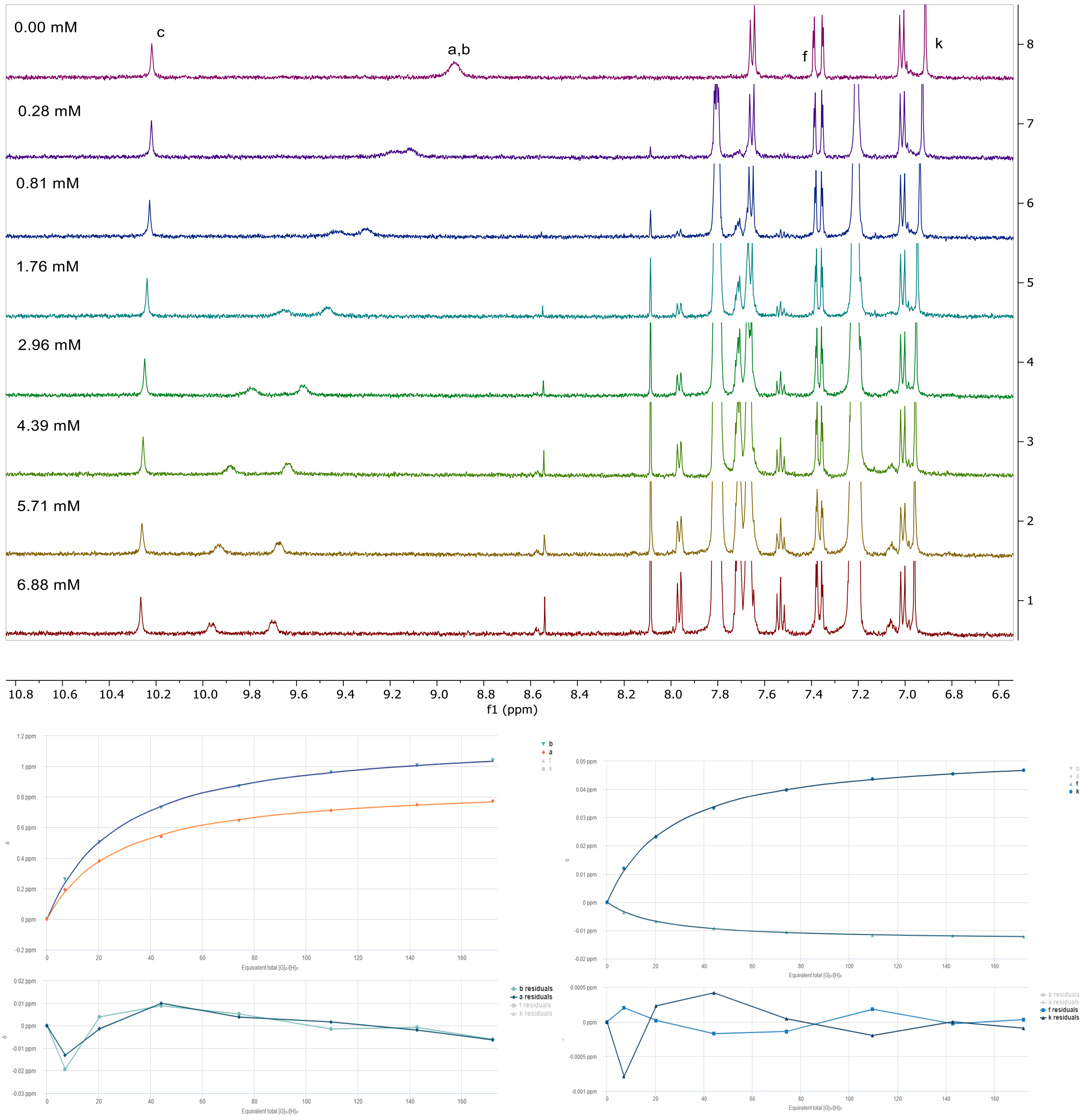

Figure S58. Top) ^{1}H NMR (500 MHz, DMSO-d^6) spectra for receptor **21** (40 μM) titrated with a combined solution of TBA Benzoate (14.4 mM) and receptor **21** (40 μM). The concentration of guest is shown on each spectrum. Bottom) Global fitting of the binding isotherms (protons a, b, f and k) from Bindfit to a 1:2 non-cooperative model K_a = 1820.6 M^{-1} (± 1.8 %). Full fitted data is available online at: http://app.supramolecular.org/bindfit/view/28aace97-2a48-4dec-993a-b5f988cecd3a

Receptor 21 & TBA chloride (DMSO-d^6)

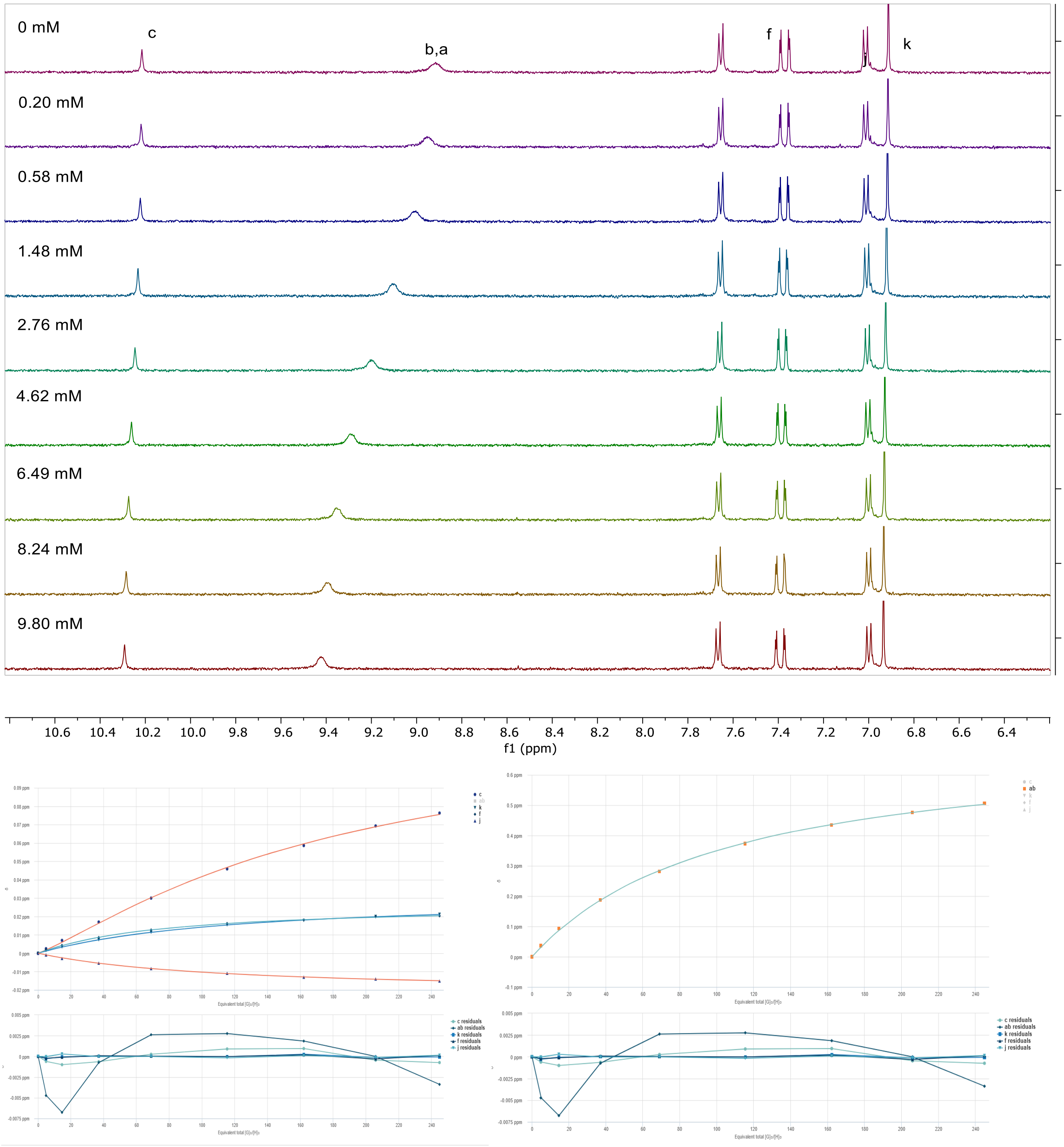

Figure S59. Top) ^{1}H NMR (500 MHz, DMSO-d^6) spectra for receptor **21** (40 μM) titrated with a combined solution of TBA chloride (20mM) and receptor **21** (40 μM). The concentration of guest is shown on each spectrum. Bottom) Global fitting of the binding isotherms (protons a, b, c, j, f and k) from Bindfit to a 1:2 non-cooperative model K_a = 458.6 M^{-1} (± 0.87 %). Full fitted data is available online at: http://app.supramolecular.org/bindfit/view/a33fa31c-141c-4383-8b9b-1e88ad02c2d1

Receptor 5 & formate (D_2O)

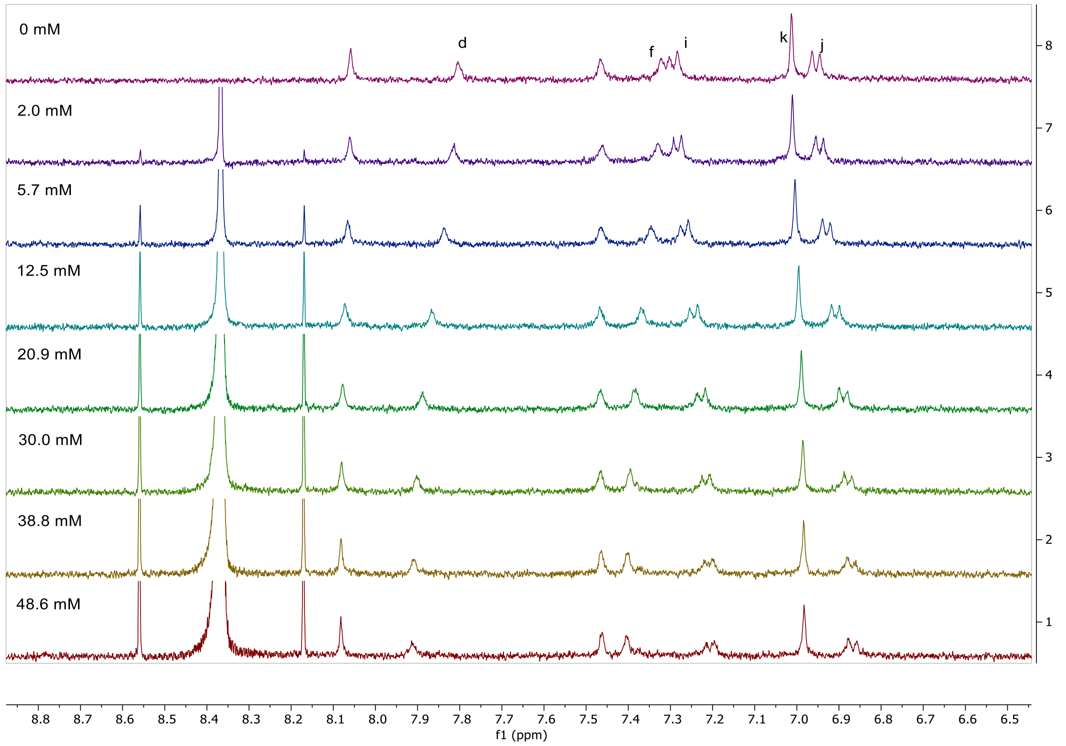

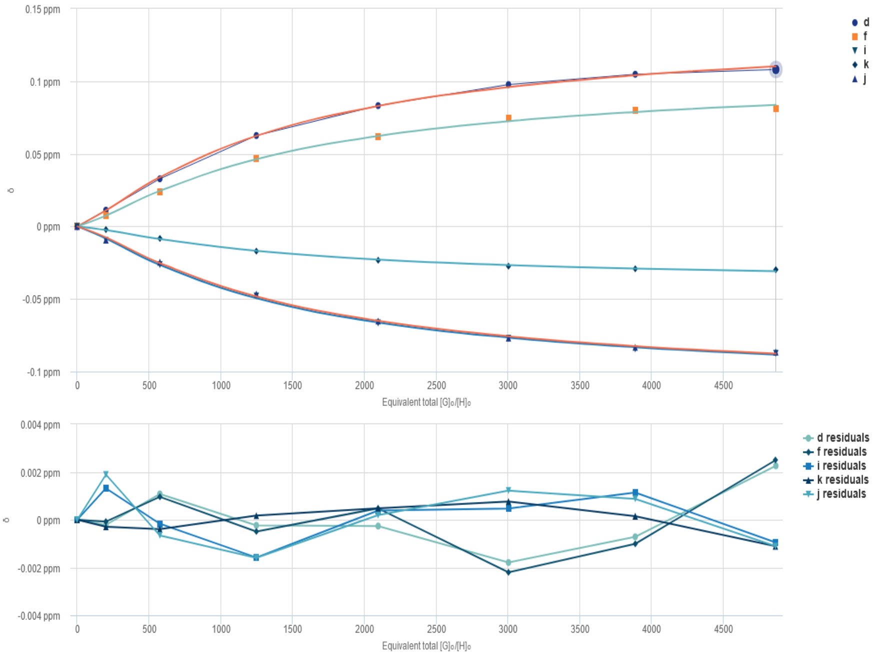

Figure S60. Top) ^{1}H NMR (500 MHz, D_2O) spectra for receptor **5** (10 μM) titrated with a combined solution of sodium formate (102 mM) and receptor **5** (10 μM). The concentration of guest is shown on each spectrum. pH = 7.59 at the end of the titration. Bottom) Global fitting of the binding isotherms (protons d, f, i , j and k) from Bindfit to a 1:2 non-cooperative model K_a = 268.7 M^{-1} (± 1.8 %). Full fitted data is available online at: http://app.supramolecular.org/bindfit/view/c364696e-6e86-4596-875a-479a3a95a37e

Receptor 5 & acetate (H_2O/D_2O)

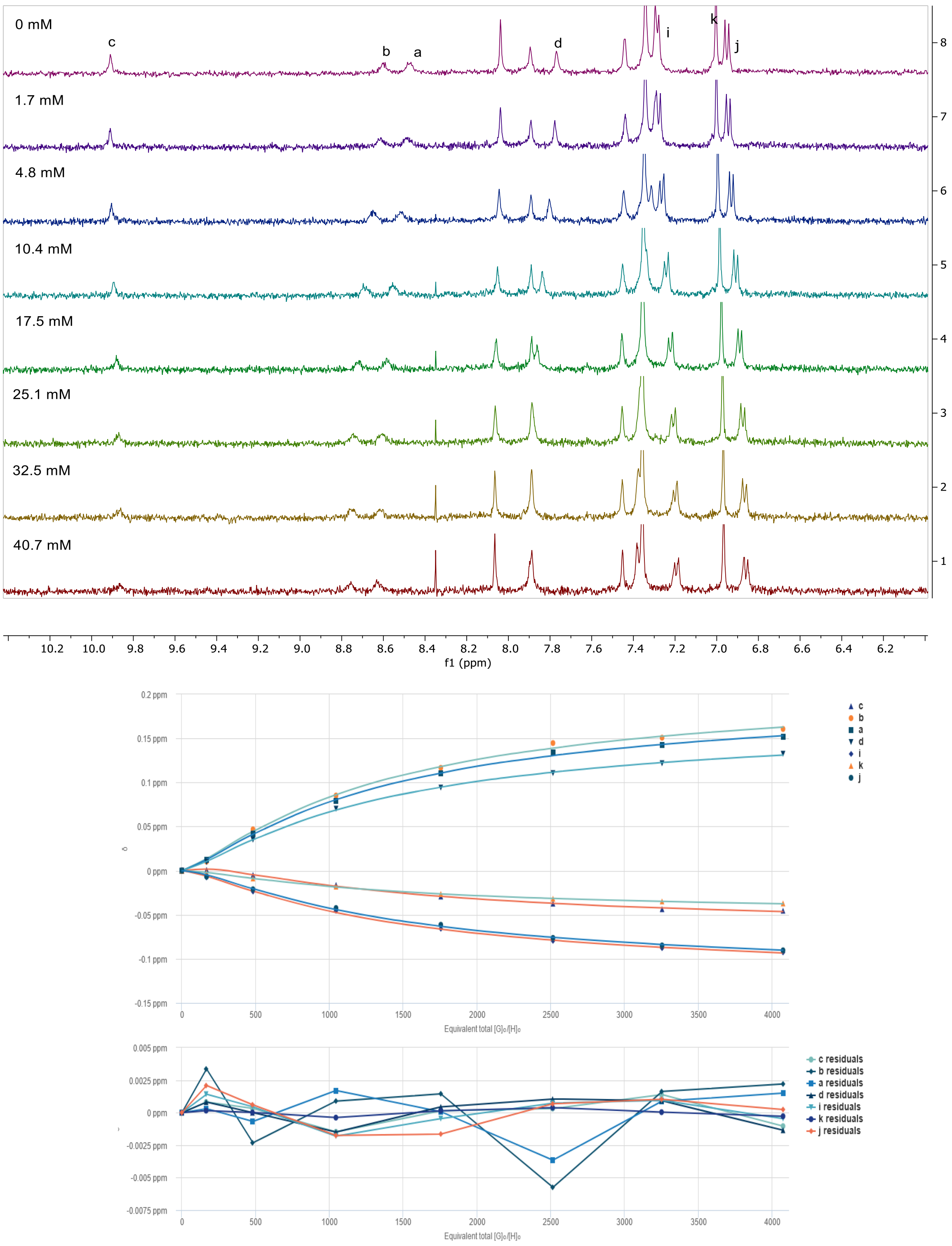

Figure S61. Top) ^{1}H NMR (500 MHz, 9:1 H_2O/D_2O) spectra for receptor **5** (10 μM) titrated with a combined solution of sodium acetate (85 mM) and receptor **5** (10 μM). The concentration of guest is shown on each spectrum. pH = 7.41 at the end of the titration. Bottom) Global fitting of the binding isotherms (protons a, b, c, d, i, j and k) from Bindfit to a 1:2 non-cooperative model K_a = 279.2 M^{-1} (± 1.4 %). Full fitted data is available online at: http://app.supramolecular.org/bindfit/view/61d190f1-8139-465c-b285-8893d28f4643

Receptor 5 & propionate (D_2O)

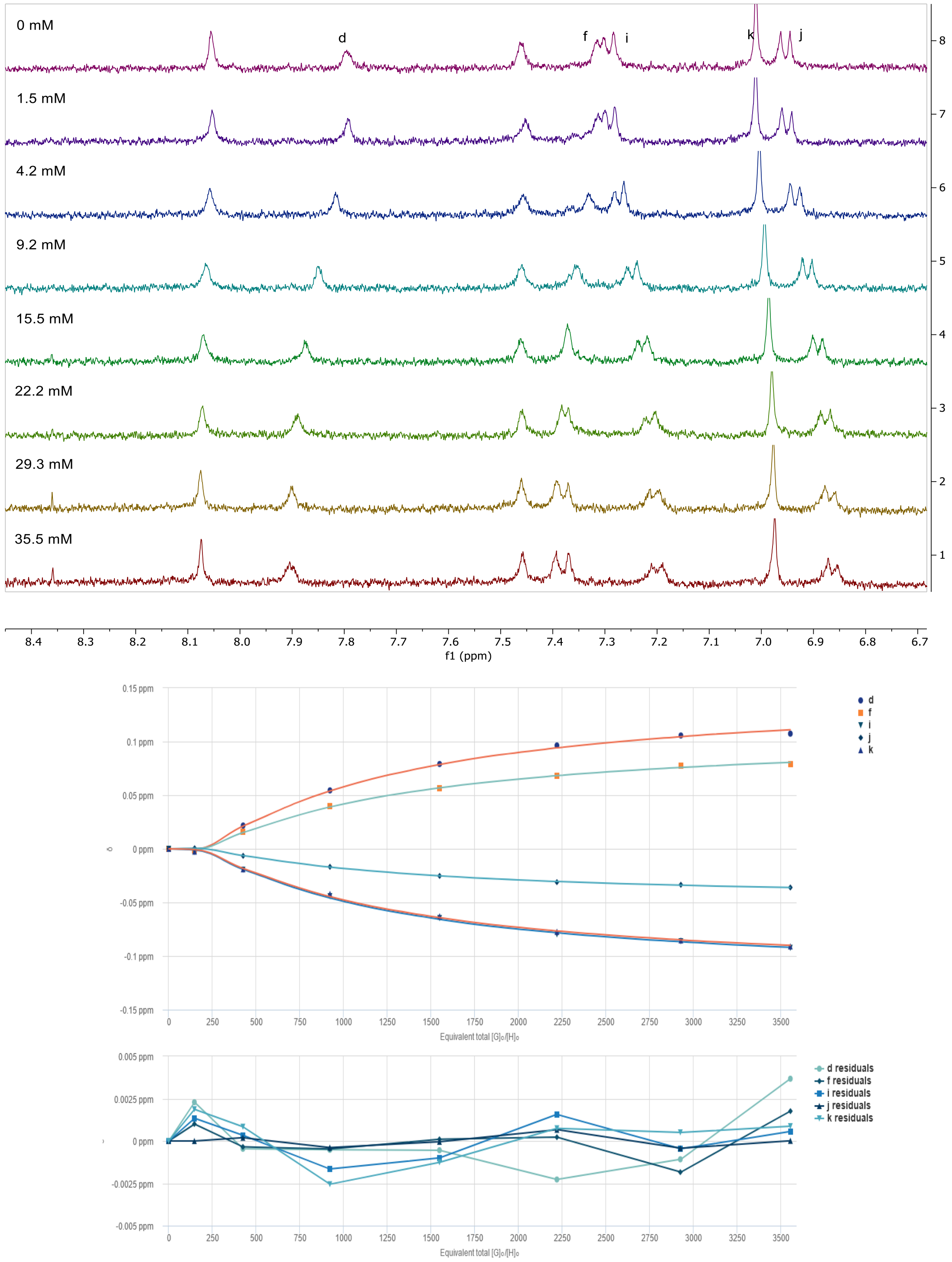

Figure S62. Top) ^{1}H NMR (500 MHz, D_2O) spectra for receptor **5** (10 µM) titrated with a combined solution of sodium propionate (75 mM) and receptor **5** (10 µM). The concentration of guest is shown on each spectrum. pH = 7.52 at the end of the titration. Bottom) Global fitting of the binding isotherms (protons d, f, i, j and k) from Bindfit to a 1:2 non-cooperative model K_a = 415.0 M^{-1} (± 1.8 %). Full fitted data is available online at: http://app.supramolecular.org/bindfit/view/a60d53a7-07de-4be7-bcf4-7442284016f5

Receptor 5 & benzoate (H_2O/D_2O)

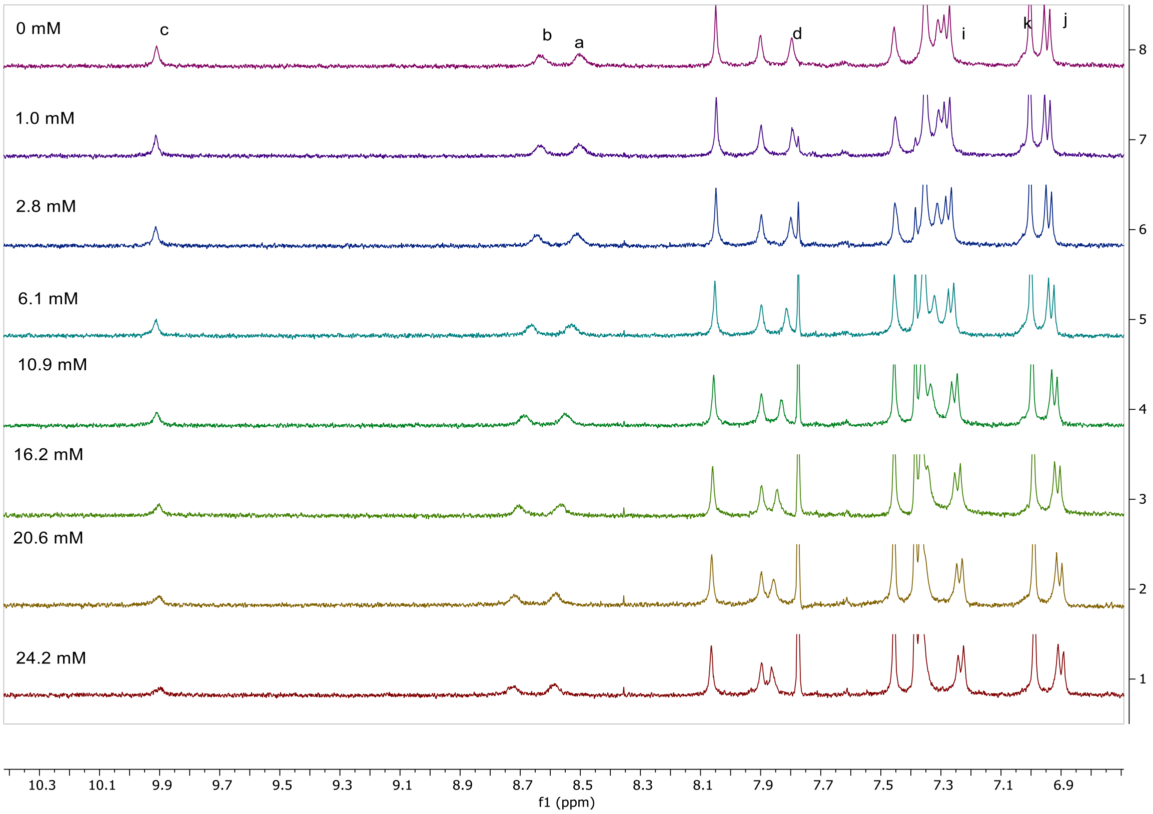

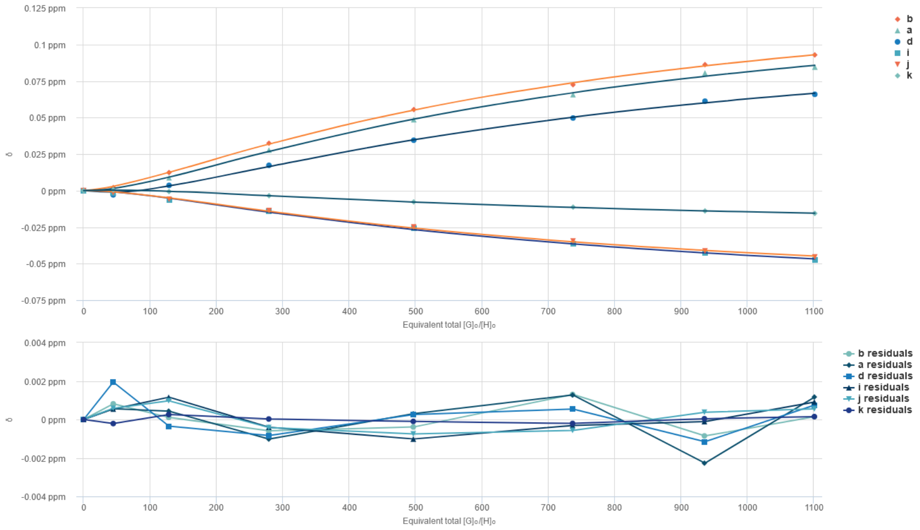

Figure S63. Top) ^{1}H NMR (500 MHz, 9:1 H_2O/D_2O) spectra for receptor **5** (22 μM) titrated with a combined solution of sodium benzoate-d^5 (50 mM) and receptor **5** (22 μM). The concentration of guest is shown on each spectrum. pH = 7.48 at the end of the titration. Bottom) Global fitting of the binding isotherms (protons a, b, d, i , j and k) from Bindfit to a 1:2 non-cooperative model K_a = 230.7 M^{-1} (± 1.0 %). Full fitted data is available online at: http://app.supramolecular.org/bindfit/view/83499776-e330-4edd-bbe7-8692016bf1e7

Receptor 5 & *L* and *D*-Lactate (D_2O)

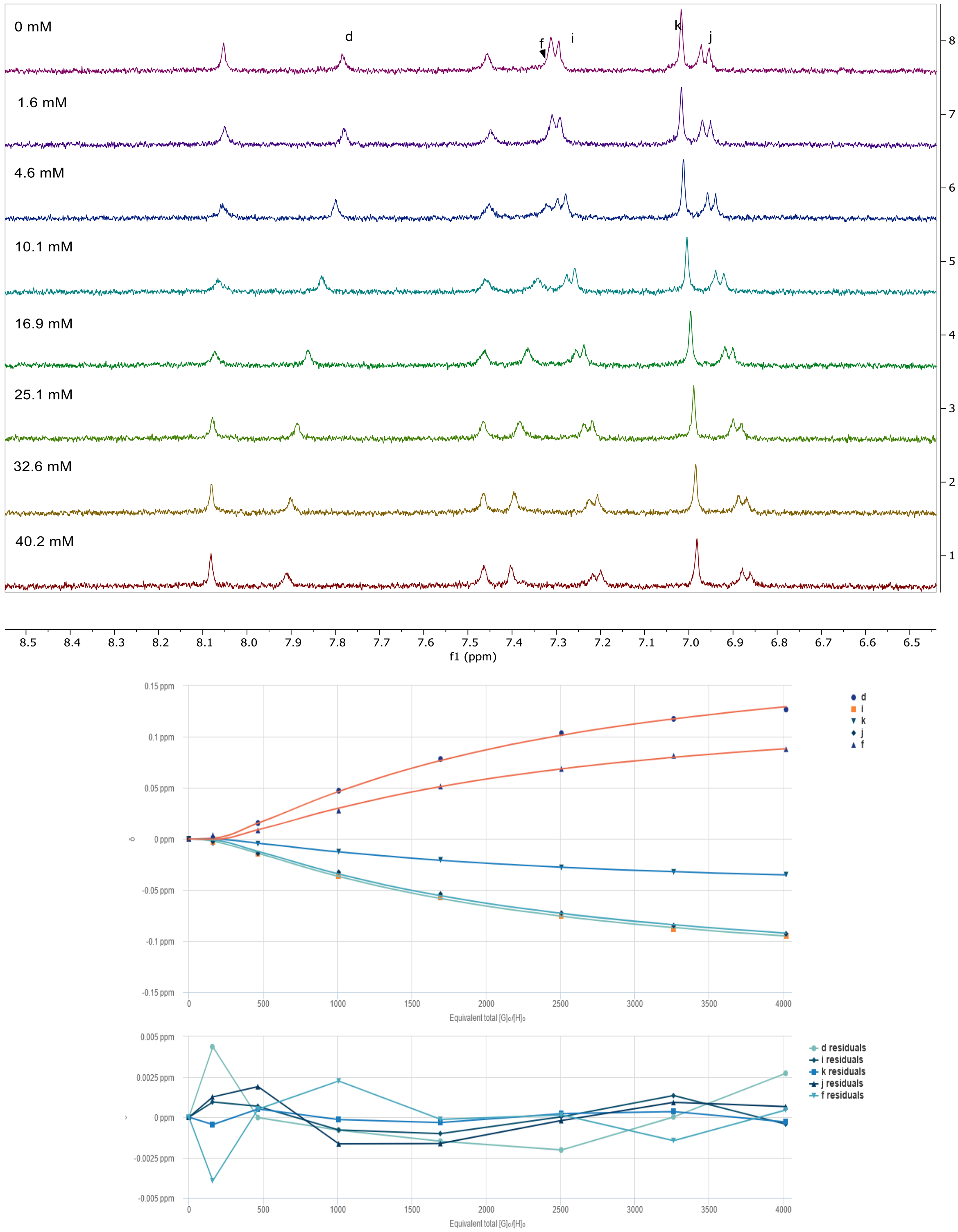

Figure S64. Top) ^{1}H NMR (500 MHz, D_2O) spectra for receptor **5** (10 μM) titrated with a combined solution of sodium *L*-Lactate (82 mM) and receptor **5** (10 μM). The concentration of guest is shown on each spectrum. pH = 7.58 at the end of the titration. Bottom) Global fitting of the binding isotherms (protons d, f, i , j and k) from Bindfit to a 1:2 non-cooperative model K_a = 204.9 M^{-1} (± 1.6 %). Full fitted data is available online at: http://app.supramolecular.org/bindfit/view/75f1b5ed-b6dc-4254-ab2d-bf846df47fbd

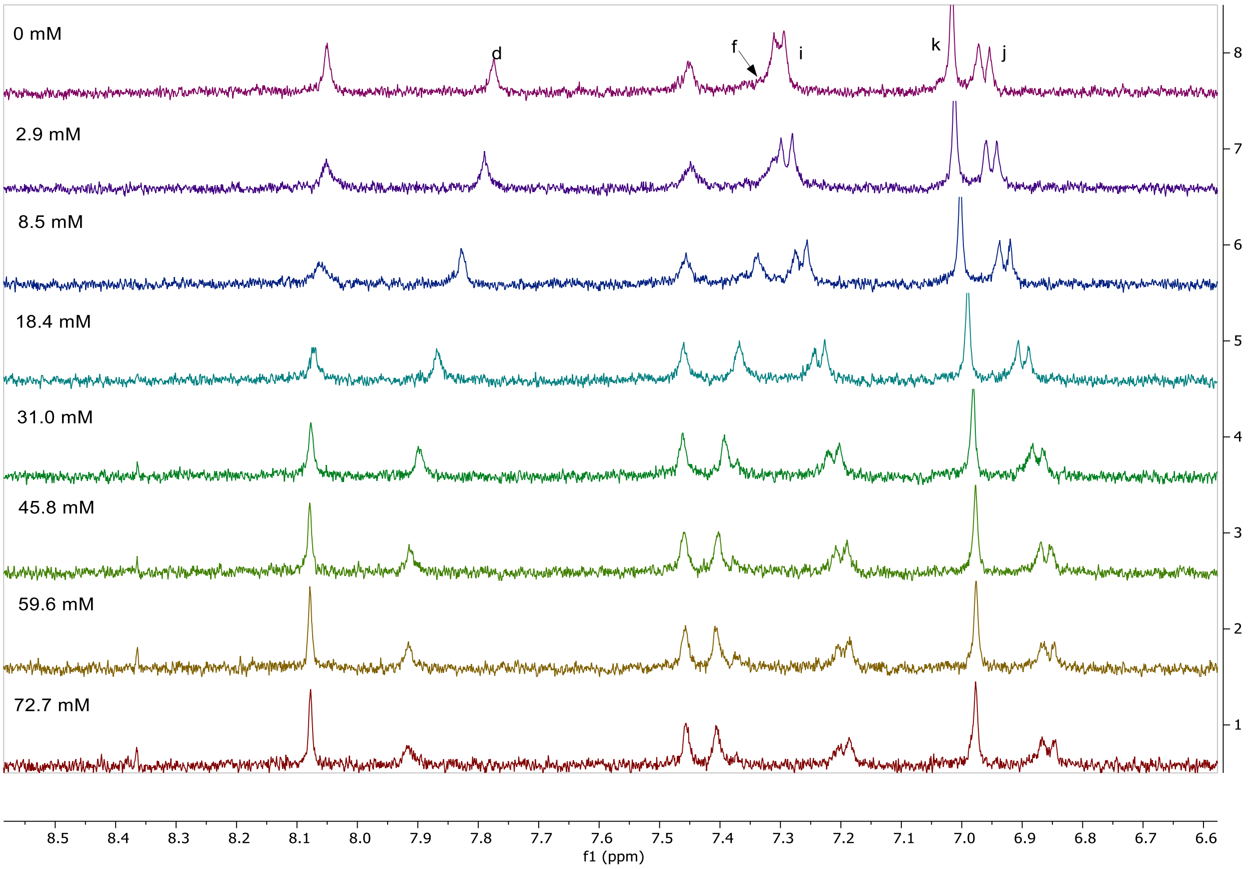

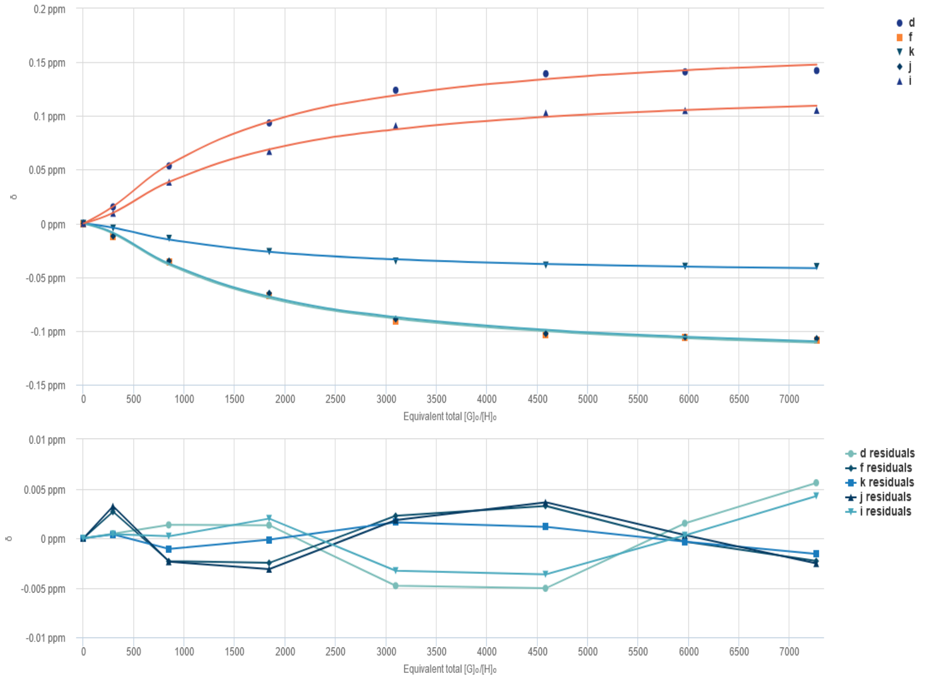

Figure S65. Top) ^{1}H NMR (500 MHz, D_2O) spectra for receptor **5** (10 μM) titrated with a combined solution of sodium *D*-Lactate (150 mM) and receptor **5** (10 μM). The concentration of guest is shown on each spectrum. pH = 7.57 at the end of the titration Bottom) Global fitting of the binding isotherms (protons d, f, i , j and k) from Bindfit to a 1:2 non-cooperative model K_a = 304.5 M^{-1} (± 3.5 %). Full fitted data is available online at: http://app.supramolecular.org/bindfit/view/10e4eb1e-063b-4243-9274-b83df416f8d0

Receptor 5 & *O*-Ac-*L*-Lactate (D_2O)

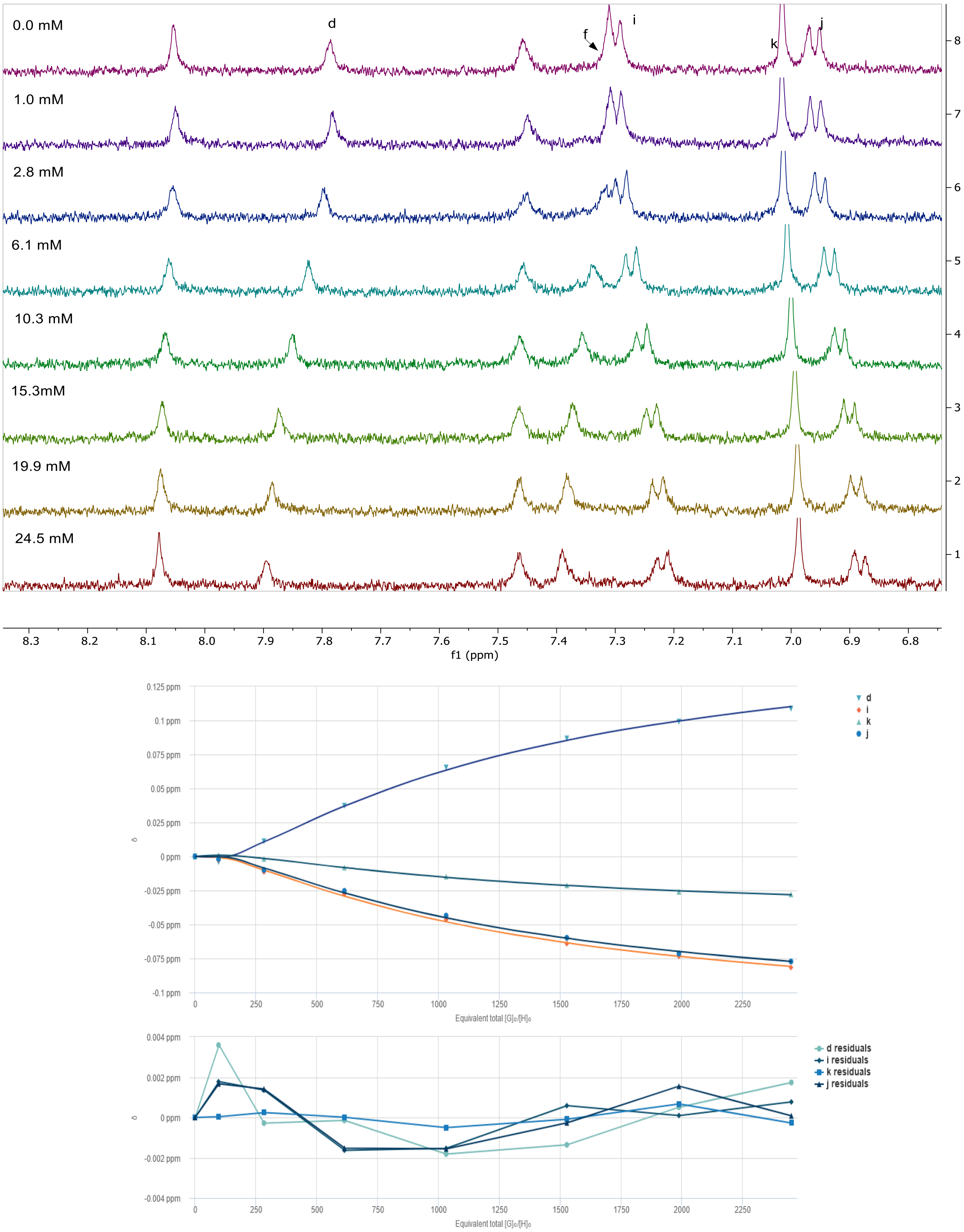

Figure S66. Top) ^{1}H NMR (500 MHz, D_2O) spectra for receptor **5** (10 µM) titrated with a combined solution of sodium *O*-Ac-*L*-Lactate (50 mM) and receptor **5** (10 µM). The concentration of guest is shown on each spectrum. pH = 7.39 at the end of the titration. Bottom) Global fitting of the binding isotherms (protons d, i , j and k) from Bindfit to a 1:2 non-cooperative model K_a = 330.8 M^{-1} (± 1.8 %). Full fitted data is available online at: http://app.supramolecular.org/bindfit/view/8a5adc39-1efc-409d-91f2-f3a868131b10

Receptor 5 & *N*-Ac-*L* and *D*-Alanine (D_2O)

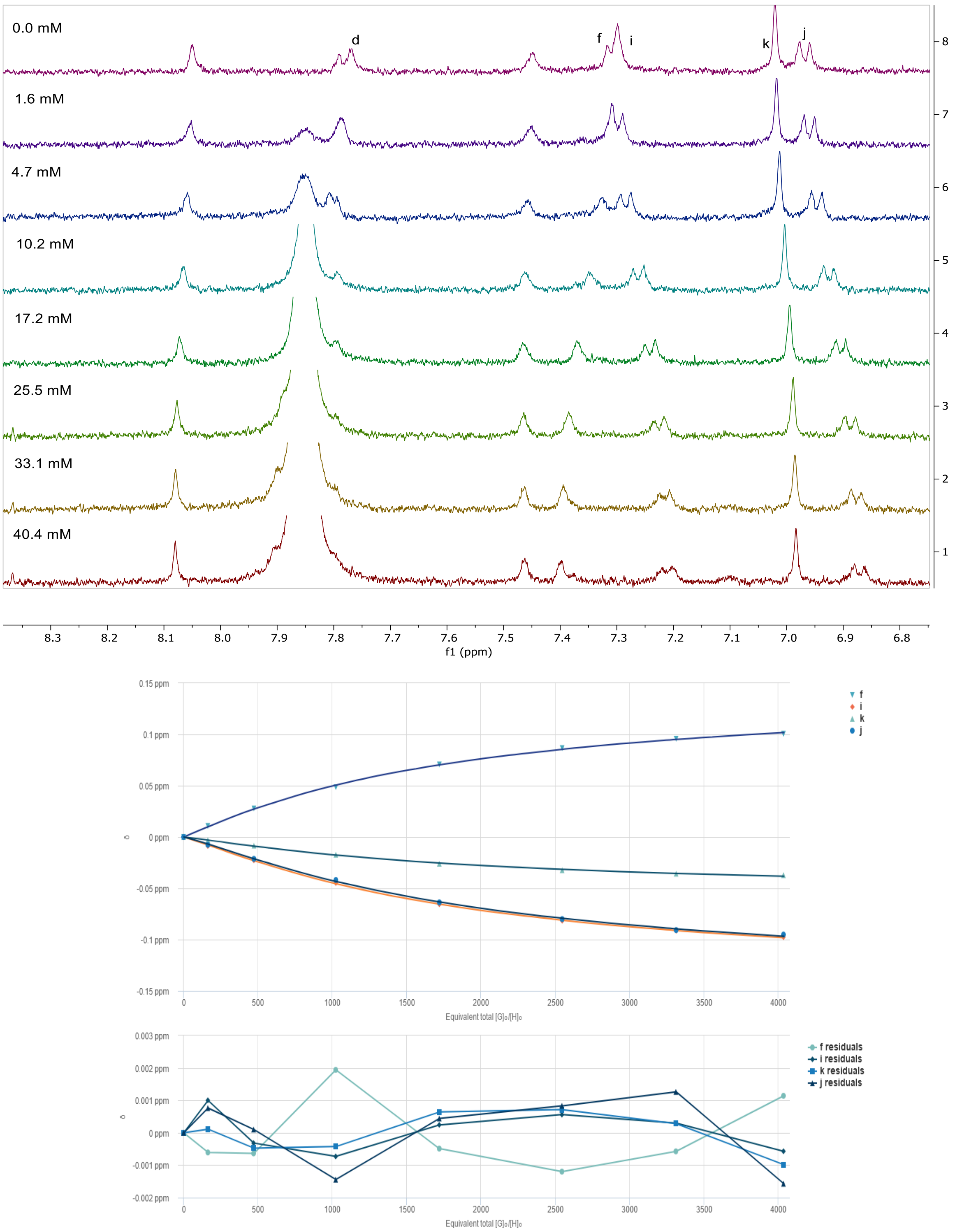

Figure S67. Top) ^{1}H NMR (500 MHz, D_2O) spectra for receptor **5** (10 μM) titrated with a combined solution of sodium *N*-Ac-*L*-Alanine (83 mM) and receptor **5** (10 μM). The concentration of guest is shown on each spectrum. pH = 7.47 at the end of the titration. Bottom) Global fitting of the binding isotherms (protons f, i , j and k) from Bindfit to a 1:2 non-cooperative model K_a = 177.3 M^{-1} (± 1.3 %). Full fitted data is available online at: http://app.supramolecular.org/bindfit/view/45c5ef57-73b9-4916-b5ed-2c96e6b45a23

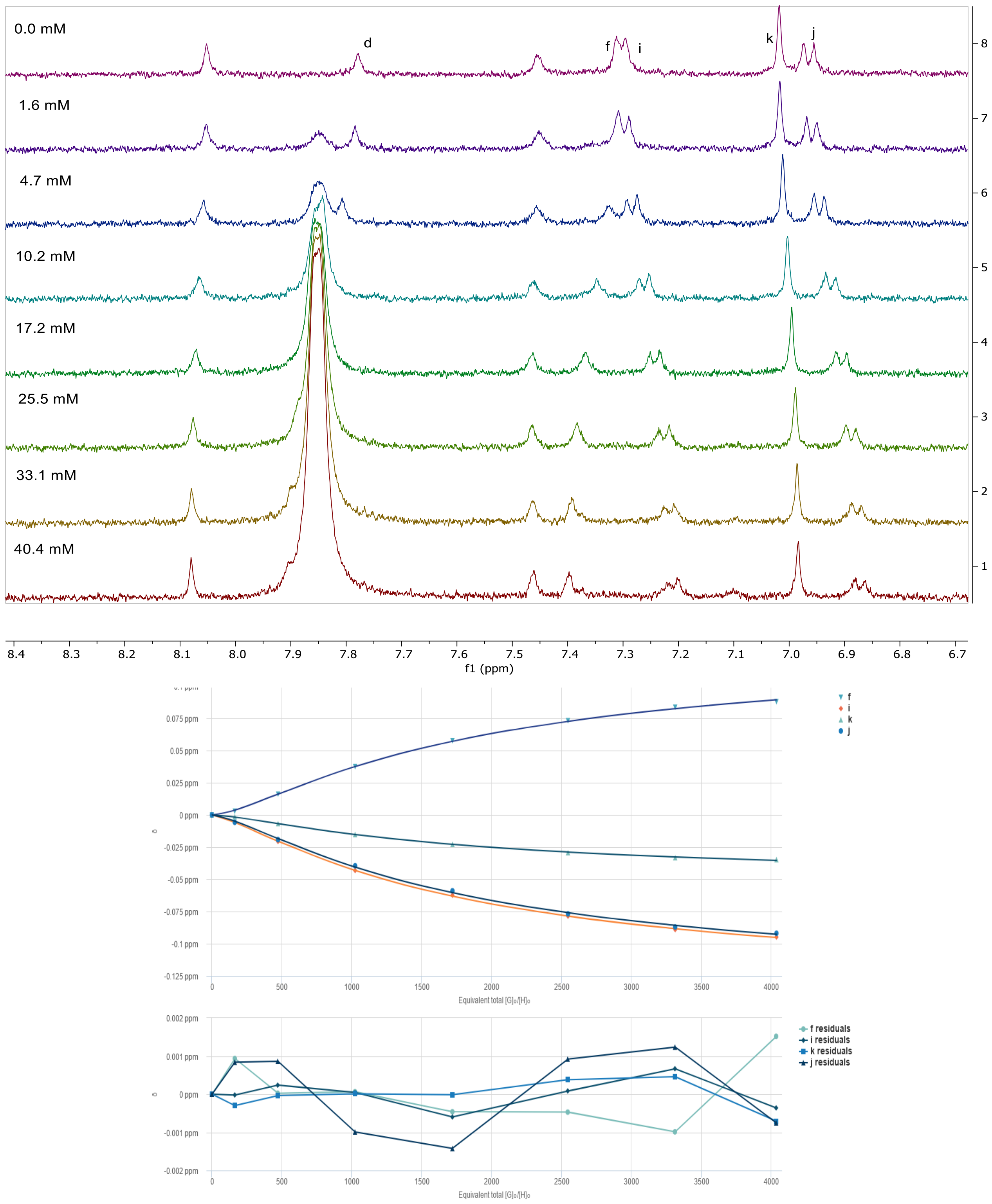

Figure S68. Top) ^{1}H NMR (500 MHz, D_2O) spectra for receptor **5** (10 μM) titrated with a combined solution of sodium *N*-Ac-*D*-Alanine (83 mM) and receptor **5** (10 μM). The concentration of guest shown on each spectrum. pH = 7.42 at the end of the titration. Bottom) Global fitting of the binding isotherms (protons f, i , j and k) from Bindfit to a 1:2 non-cooperative model K_a = 205.5 M^{-1} (± 1.1 %). Full fitted data is available online at: http://app.supramolecular.org/bindfit/view/37f30eeb-a939-405f-a25b-c8da02febd96

Receptor 5 & chloride (H_2O/D_2O)

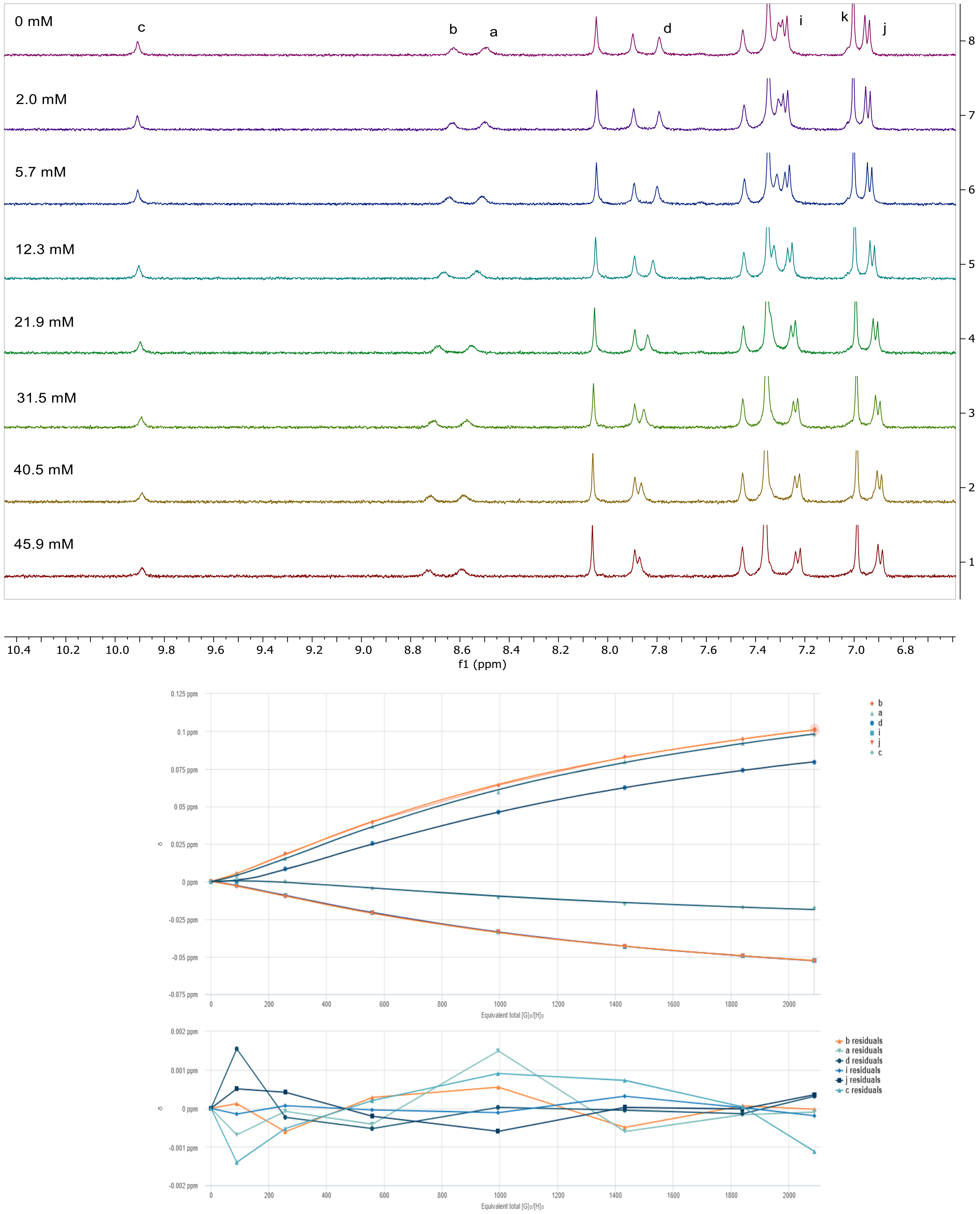

Figure S69. Top) ^{1}H NMR (500 MHz, 9:1 H_2O/D_2O) spectra for receptor **5** (22 μM) titrated with a combined solution of sodium chloride (100 mM) and receptor **5** (22 μM). The concentration of guest is shown on each spectrum. pH = 7.43 at the end of the titration. Bottom) Global fitting of the binding isotherms (protons a, b, c, d, i and j) from Bindfit to a 1:2 non-cooperative model K_a = 114.9 M^{-1} (± 0.6 %). Full fitted data is available online at: http://app.supramolecular.org/bindfit/view/3cde4c30-29e0-4b7e-85f3-65e4c55a62f3

Receptor 5 & bromide (H_2O/D_2O)

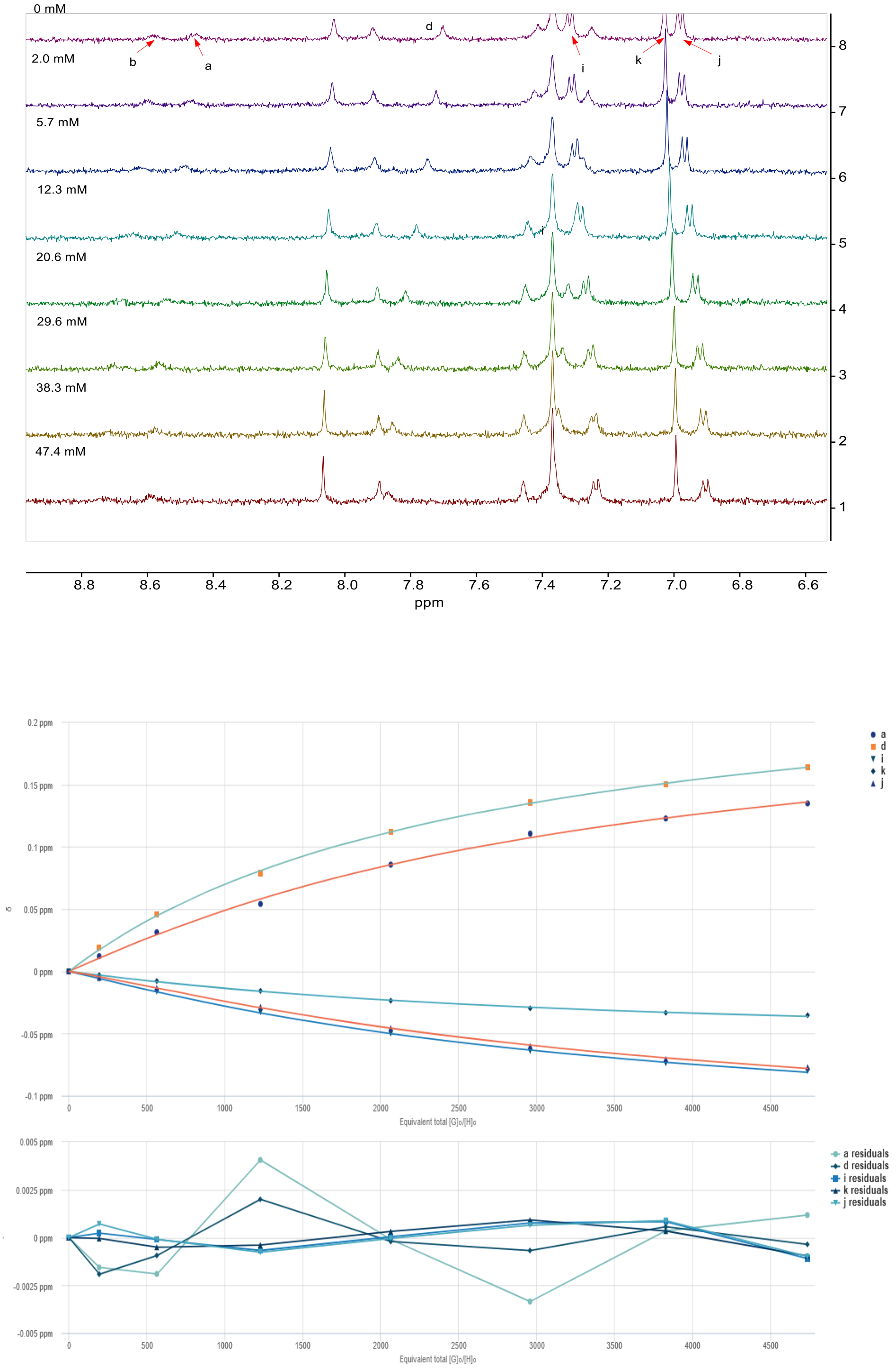

Figure S70. Top) ^{1}H NMR (600 MHz, 9:1 H_2O/D_2O) spectra for receptor **5** (10 µM) titrated with a combined solution of sodium bromide (100 mM) and receptor **5** (10 µM). The concentration of guest is shown on each spectrum. pH = 7.46 at the end of the titration. Bottom) Global fitting of the binding isotherms (protons a, d, i, j and k) from Bindfit to a 1:2 non-cooperative model K_a = 94.0 M^{-1} (± 1.2 %). Full fitted data is available online at: http://app.supramolecular.org/bindfit/view/f91ffe80-6282-4d5c-8863-74e0800841d2

Receptor 5 & iodide (H_2O/D_2O)

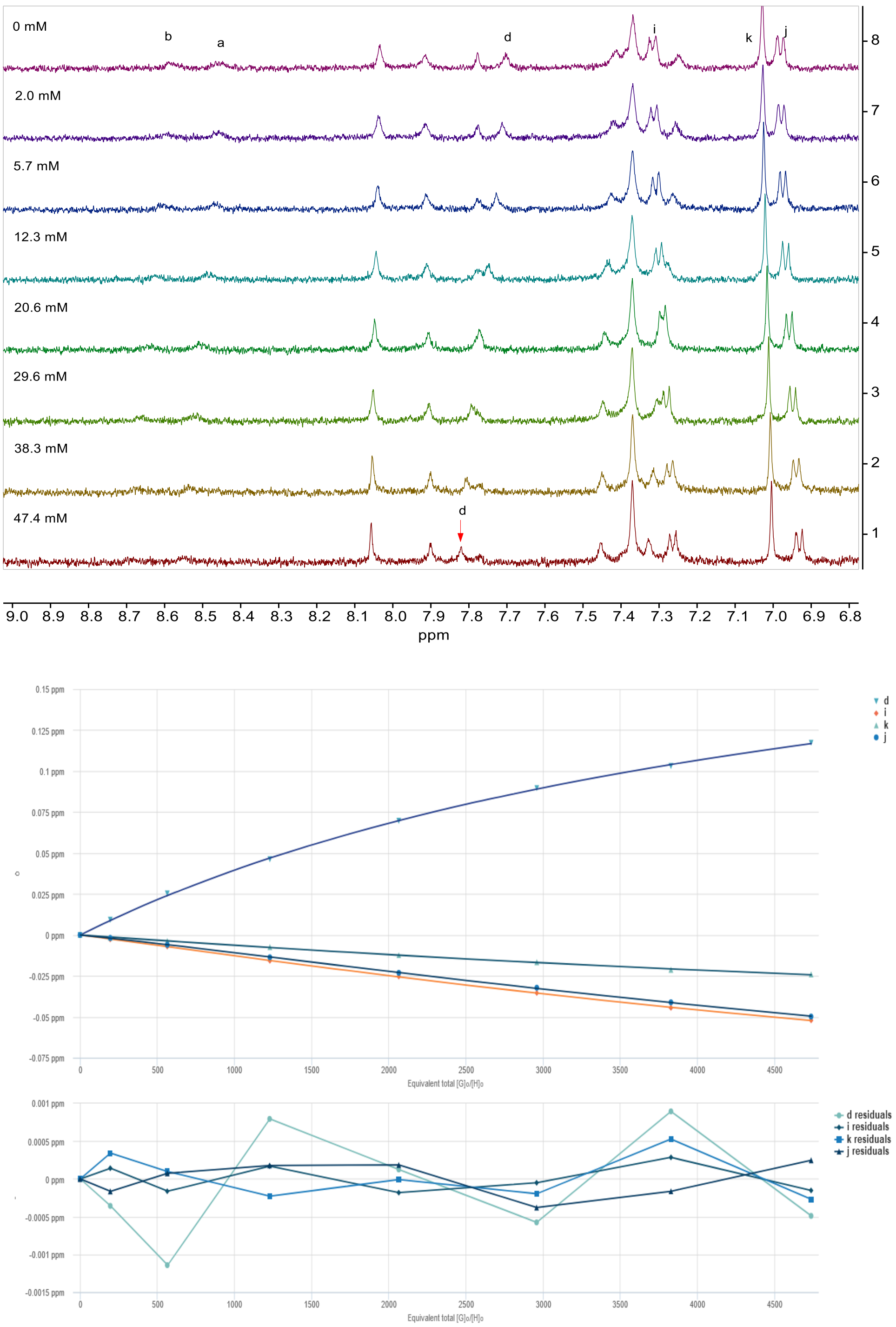

Figure S71. Top) ^{1}H NMR (600 MHz, 9:1 H_2O/D_2O) spectra for receptor **5** (10 μM) titrated with a combined solution of sodium iodide (100 mM) and receptor **5** (10 μM). The concentration of guest is shown on each spectrum. pH = 7.36 at the end of the titration. Bottom) Global fitting of the binding isotherms (protons d, i, j and k) from Bindfit to a 1:2 non-cooperative model $K_a = 33.2\ M^{-1}$ (± 0.6 %). Full fitted data is available online at: http://app.supramolecular.org/bindfit/view/b9826ab7-df6b-49af-b727-dd5417fe8dd1

Receptor 5 & nitrate (H_2O/D_2O)

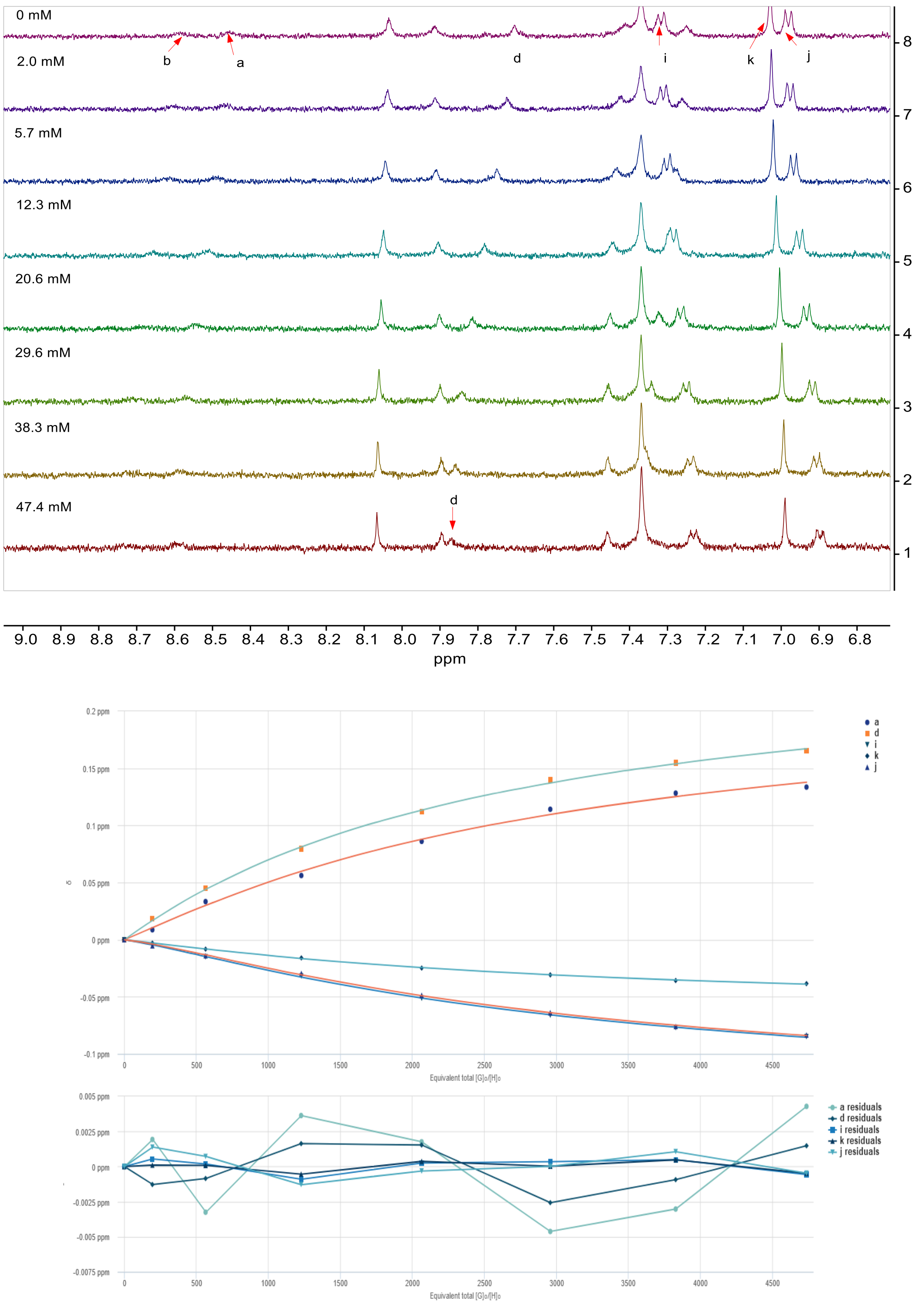

Figure S72. Top) ^{1}H NMR (600 MHz, 9:1 H_2O/D_2O) spectra for receptor **5** (10 μM) titrated with a combined solution of sodium nitrate (100 mM) and receptor **5** (10 μM). The concentration of guest is shown on each spectrum. pH = 7.43 at the end of the titration. Bottom) Global fitting of the binding isotherms (protons a, d, i, j and k) from Bindfit to a 1:2 non-cooperative model K_a = 107.0 M^{-1} (± 1.7 %). Full fitted data is available online at: http://app.supramolecular.org/bindfit/view/a85acf20-49fa-46bc-b8f1-437538c1d15d

Receptor 5 & sulfate (H_2O/D_2O)

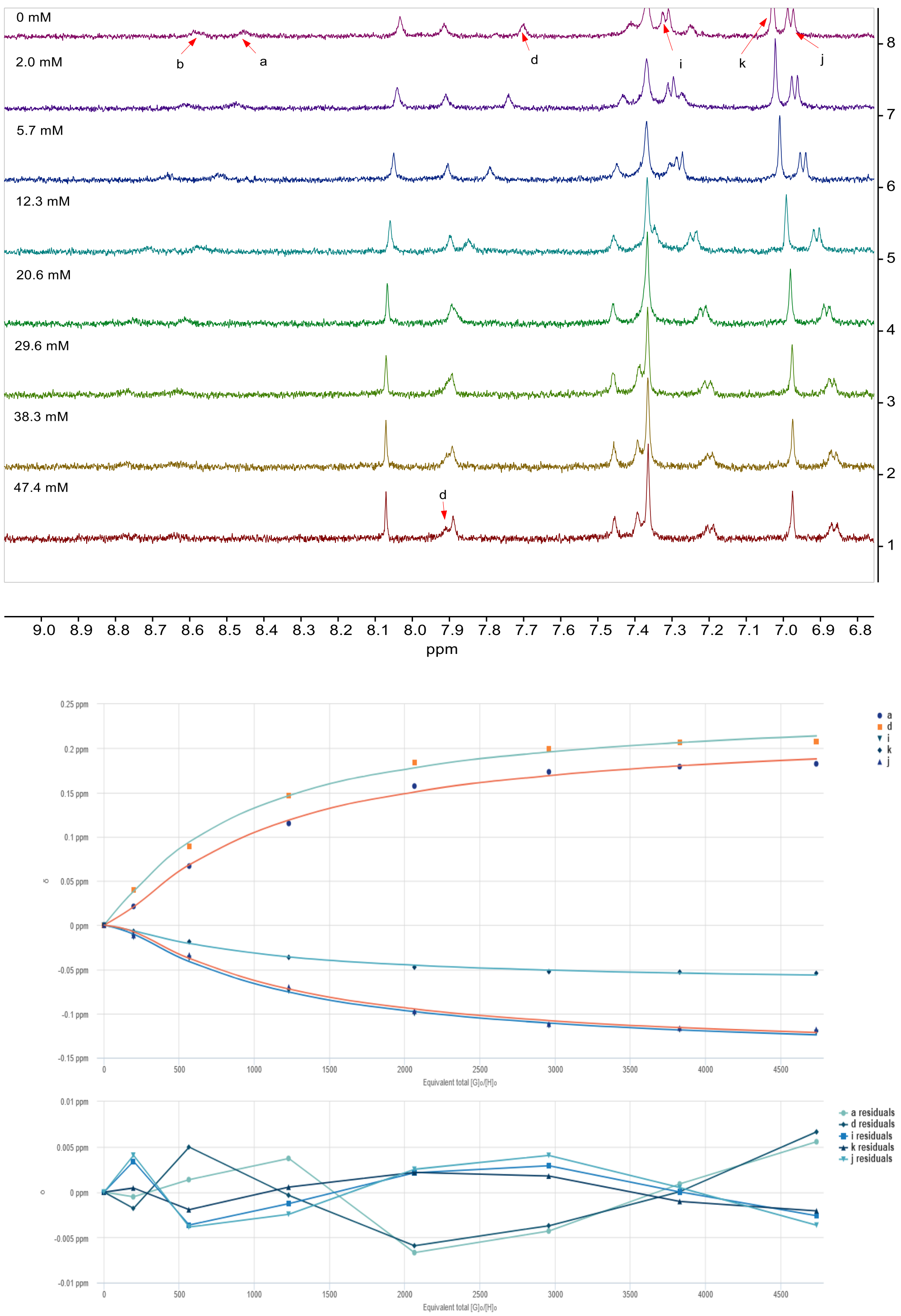

Figure S73. Top) ^{1}H NMR (600 MHz, 9:1 H_2O/D_2O) spectra for receptor **5** (10 μM) titrated with a combined solution of sodium sulfate (100 mM) and receptor **5** (10 μM). The concentration of guest is shown on each spectrum. pH = 7.56 at the end of the titration. Bottom) Global fitting of the binding isotherms (protons a, d, i, j and k) from Bindfit to a 1:2 non-cooperative model K_a = 412.3 M^{-1} (± 3.3 %). Full fitted data is available online at: http://app.supramolecular.org/bindfit/view/d78c9e1e-cc2f-492a-a44f-5e242d387eba

Receptor 5 & *L/D*-Alanine (D_2O)

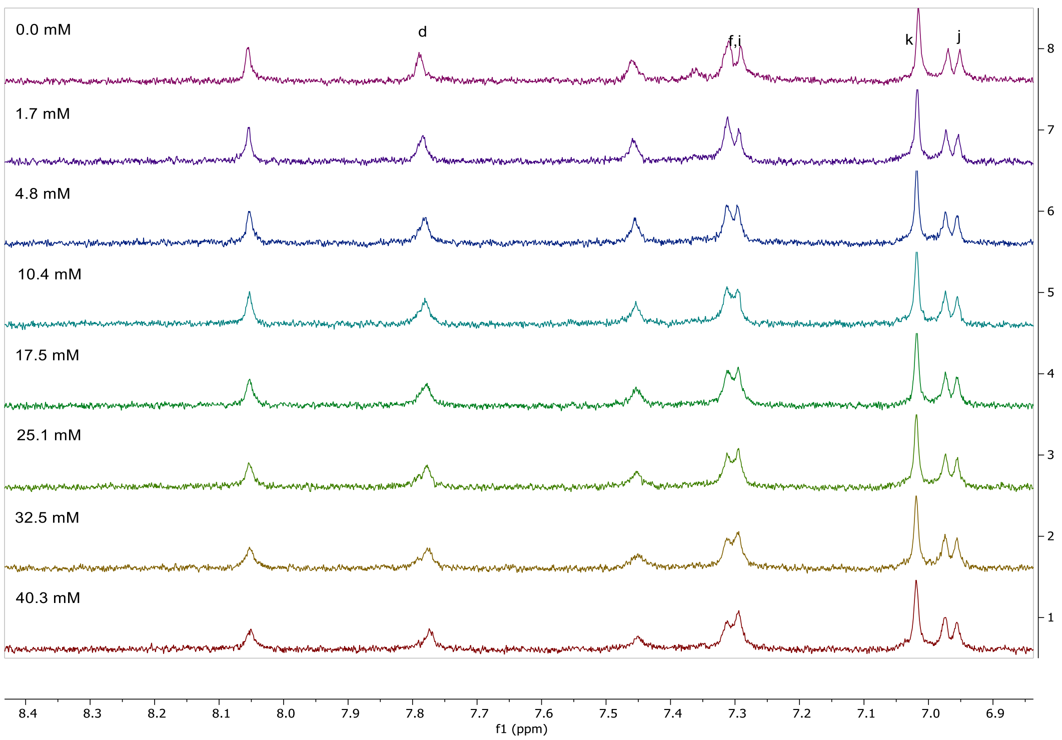

Figure S74. ^{1}H NMR (500 MHz, D_2O) spectra for receptor **5** (10 µM) titrated with a combined solution of sodium *L*-Alanine (85 mM) and receptor **5** (10 µM). The concentration of guest is shown on each spectrum. pH = 7.50 at the end of the titration. No peak movements were observed.

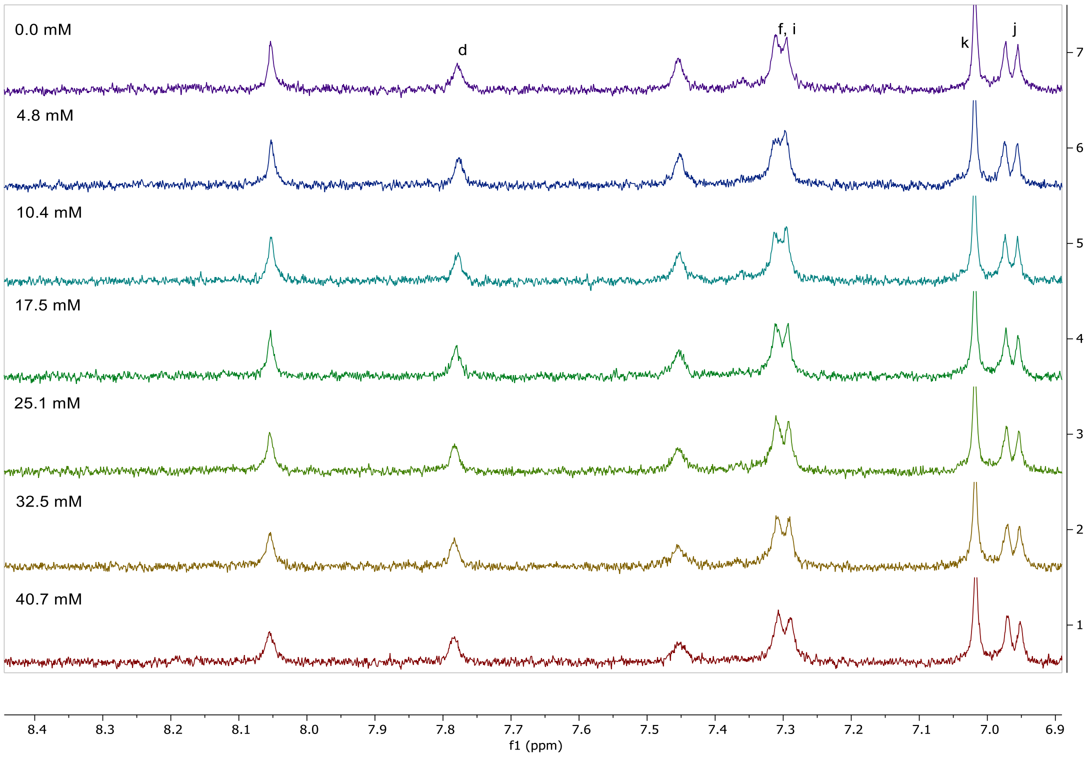

Figure S75. ^{1}H NMR (500 MHz, D_2O) spectra for receptor **5** (10 µM) titrated with a combined solution of sodium *D*-Alanine (85 mM) and receptor **5** (10 µM). The concentration of guest is shown on each spectrum. pH = 7.55 at the end of the titration. No peak movements were observed.

Receptor 5 & *L*-Ala-*L*-Ala (D_2O)

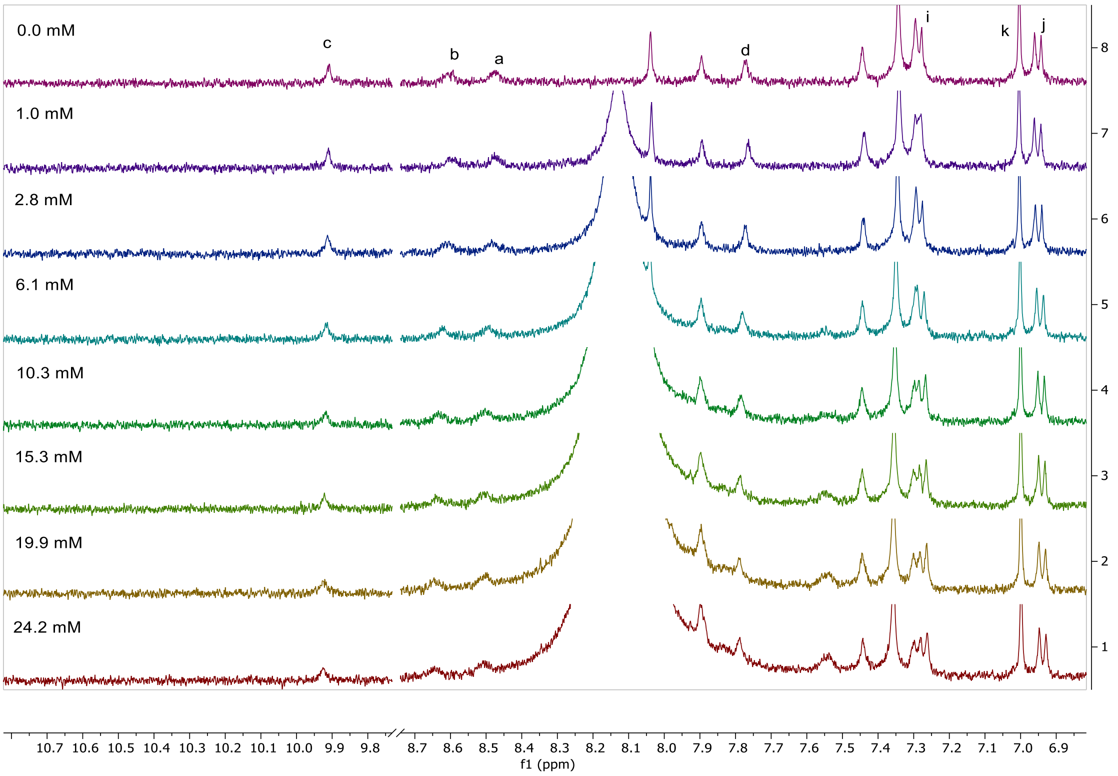

Figure S76. ^{1}H NMR (500 MHz, D_2O) spectra for receptor **5** (10 μM) titrated with a combined solution of sodium *L*-Ala-*L*-Ala (50 mM) and receptor **5** (10 μM). The concentration of guest is shown on each spectrum. pH = 7.41 at the end of the titration. Very small peak movements were observed (< 0.02 ppm), compared to other titration spectra for receptor **5**, it is very likely that receptor **5** does not binding *L*-Ala-*L*-Ala.

Receptor 6 & acetate (H_2O/D_2O)

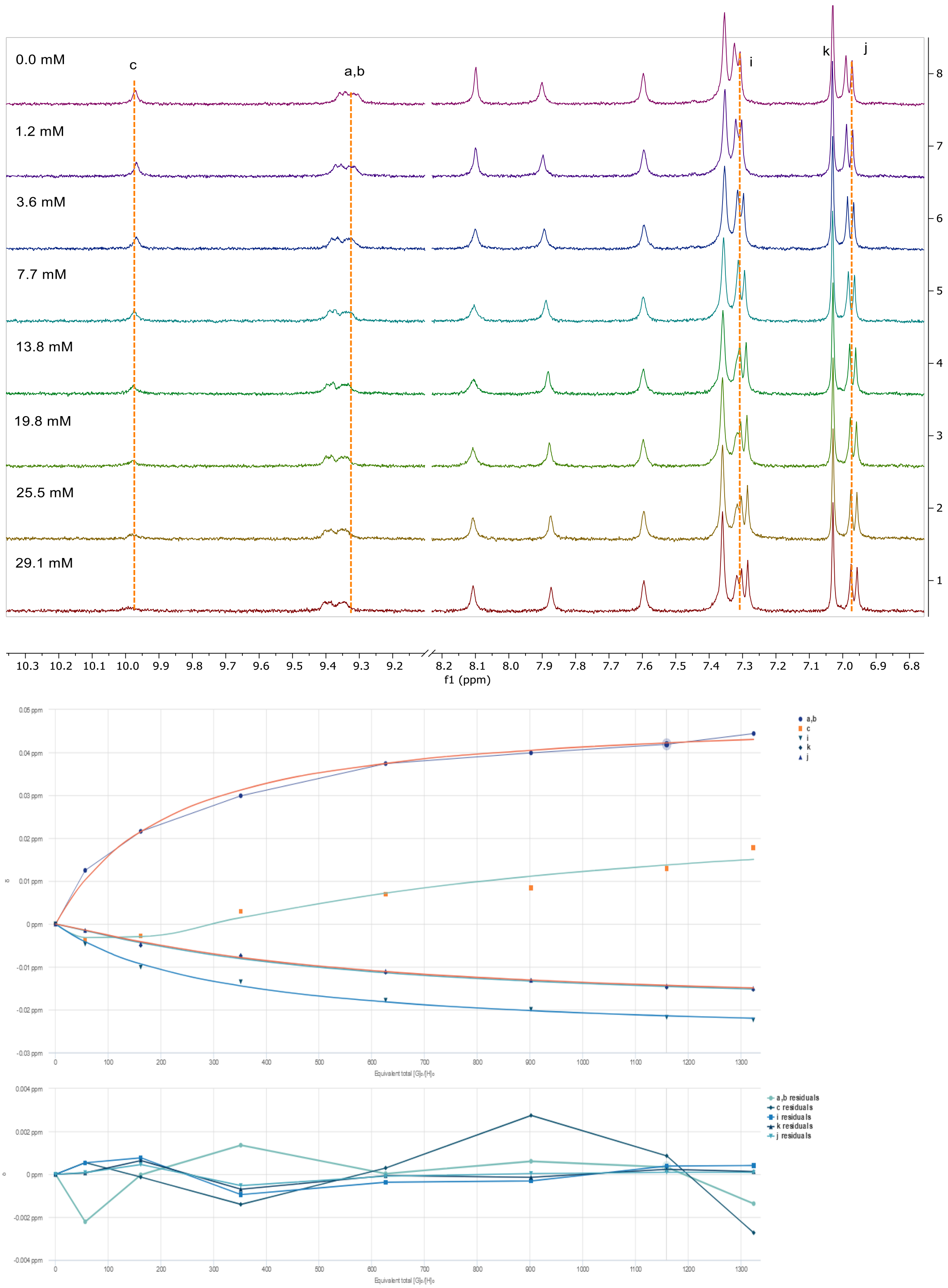

Figure S77. Top) ^{1}H NMR (500 MHz, 9:1 H_2O/D_2O) spectra for receptor **6** (22 μM) titrated with a combined solution of sodium acetate (63 mM) and receptor **6** (22 μM). The concentration of guest is shown on each spectrum. pH = 7.48 at the end of the titration. Bottom) Global fitting of the binding isotherms (protons a, b, c, i , j and k) from Bindfit to a 1:2 non-cooperative model K_a = 315.2 M^{-1} (± 5.4 %). Full fitted data is available online at: http://app.supramolecular.org/bindfit/view/2db09263-8085-4139-8a90-9a1b09596f04

Receptor 6 & propionate (H_2O/D_2O)

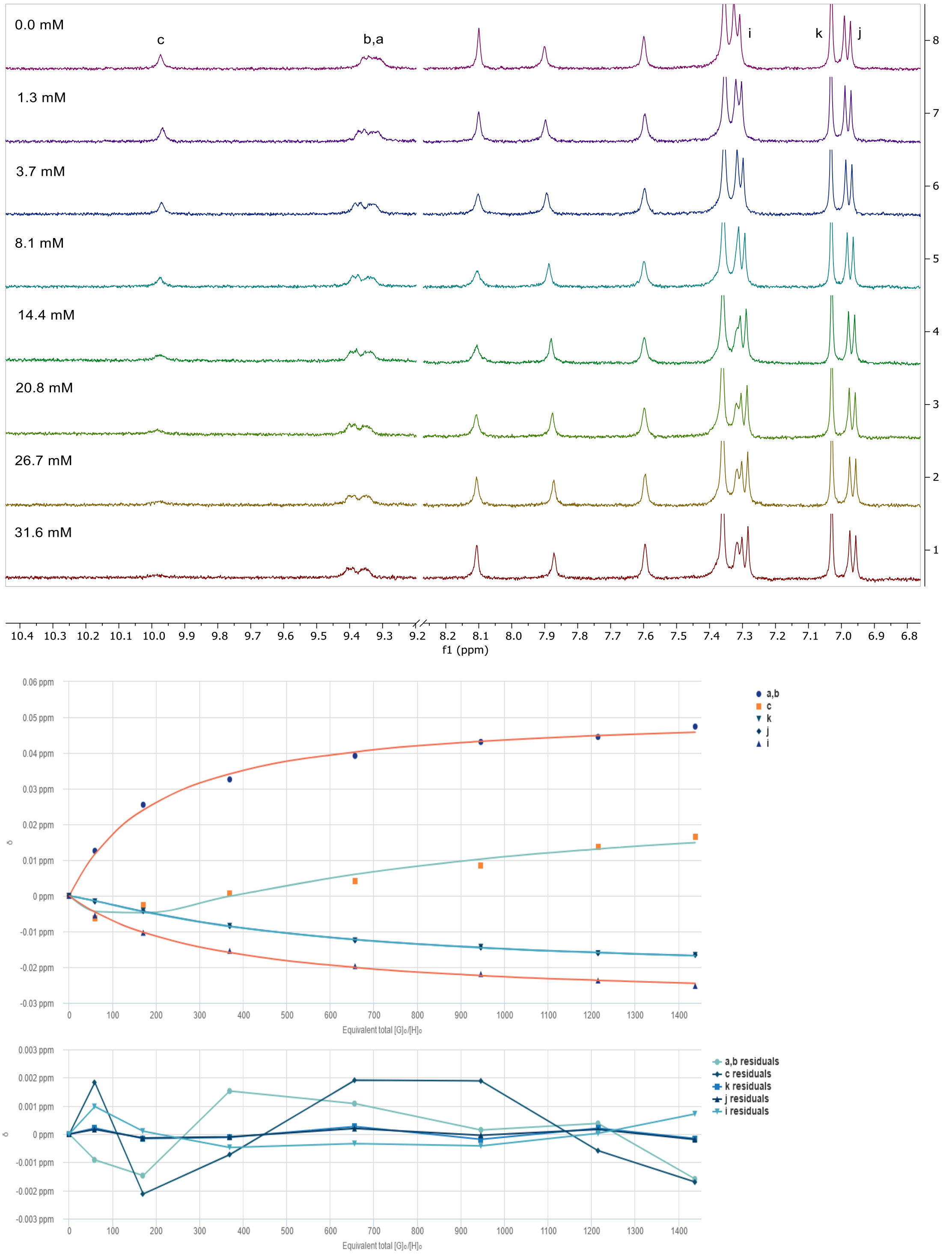

Figure S78. Top) ^{1}H NMR (500 MHz, 9:1 H_2O/D_2O) spectra for receptor **6** (22 μM) titrated with a combined solution of sodium propionate (66 mM) and receptor **6** (22 μM). The concentration of guest is shown on each spectrum. pH = 7.52 at the end of the titration. Bottom) Global fitting of the binding isotherms (protons a, b, c, i , j and k) from Bindfit to a 1:2 non-cooperative model K_a = 311.9 M^{-1} (± 5.0 %). Full fitted data is available online at: http://app.supramolecular.org/bindfit/view/64882fad-c6c8-4de9-94ac-b9d16654a05b

Receptor 6 & benzoate (H_2O/D_2O)

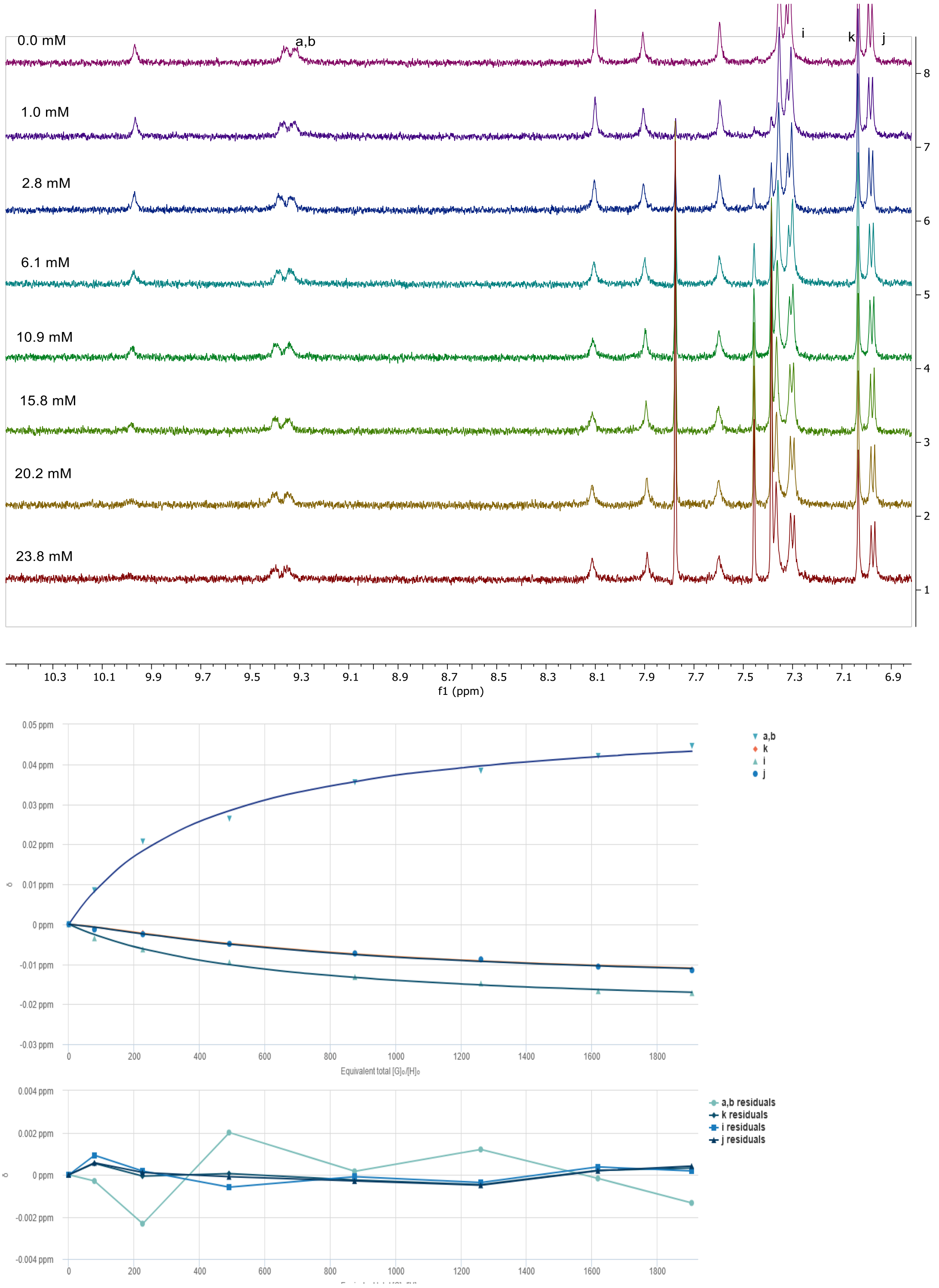

Figure S79. Top) ^{1}H NMR (500 MHz, 9:1 H_2O/D_2O) spectra for receptor **6** (13 μM) titrated with a combined solution of sodium benzoate-d^5 (50 mM) and receptor **6** (13 μM). The concentration of guest is shown on each spectrum. pH = 7.54 at the end of the titration. Bottom) Global fitting of the binding isotherms (protons a, b, i , j and k) from Bindfit to a 1:2 non-cooperative model K_a = 317.7 M^{-1} (± 5.4 %). Full fitted data is available online at: http://app.supramolecular.org/bindfit/view/5a94e312-c445-4639-8b6a-ac6773ec29a1

NMR Structural Studies of Hosts and Complexes

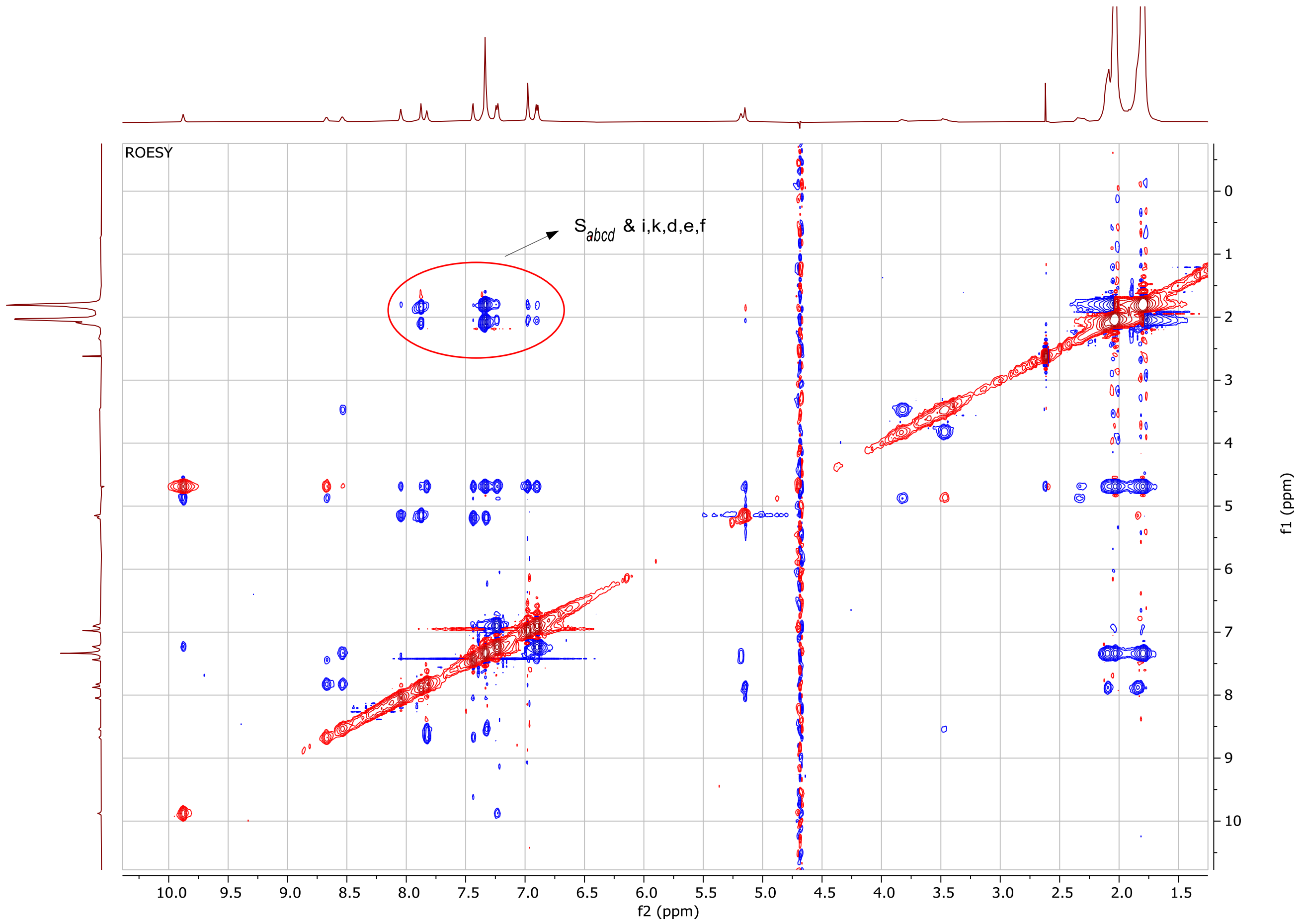

Figure S80. 2D ROESY NMR spectrum (600 MHz) of receptor **5** (1.33 mM) in 9:1 H_2O/D_2O. Cross peaks in the red circle represent connections between solubilising groups and the receptor core aromatic protons, probably due to self-association at this relatively high concentration.

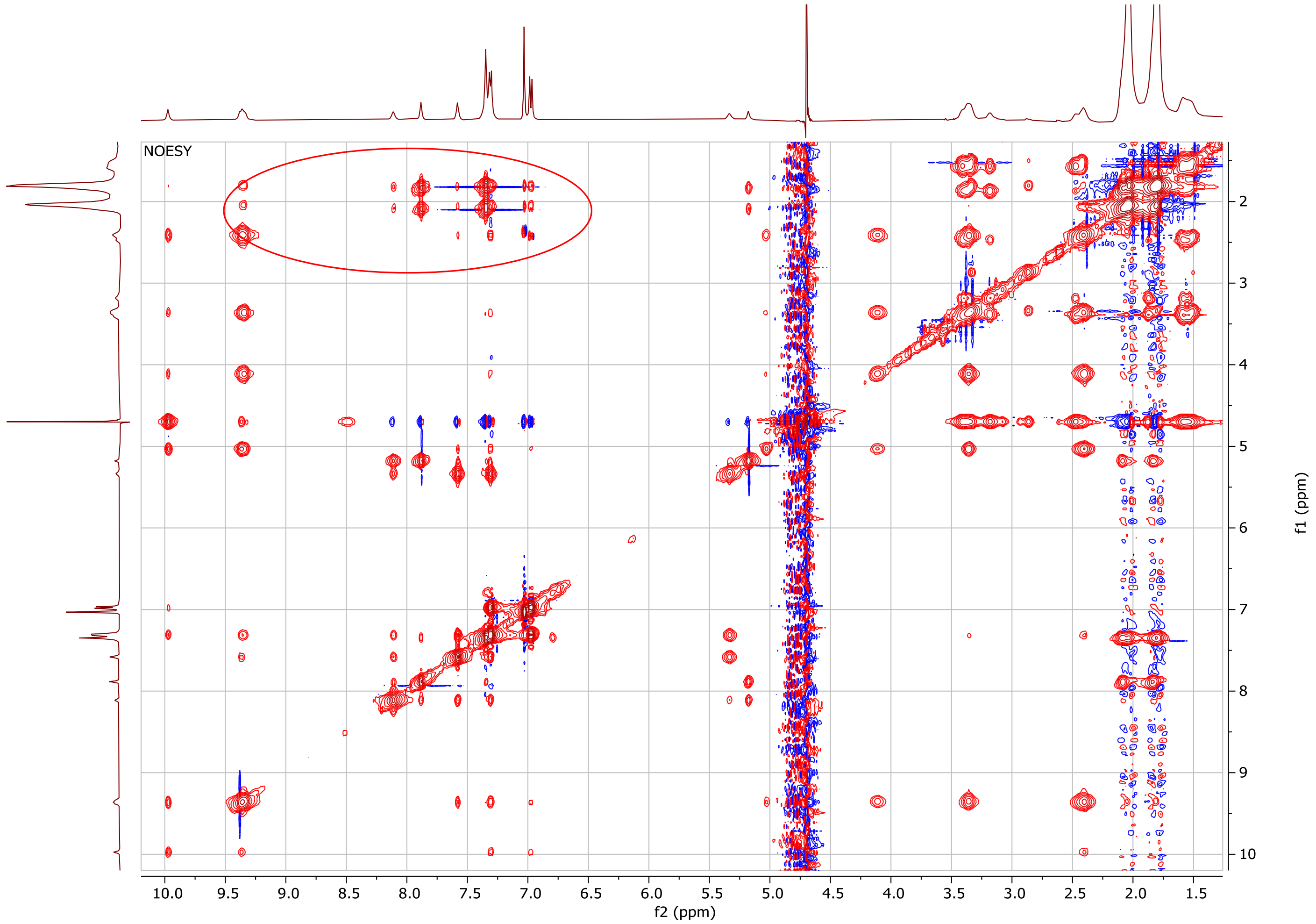

Figure S81. 2D NOESY NMR spectrum (600 MHz, 350 ms mixing time) of receptor **6** (1.81 mM) in 9:1 H_2O/D_2O. Cross peaks in the red circle represent connections between solubilising groups and the receptor core aromatic protons, probably due to self-association at this relatively high concentration.

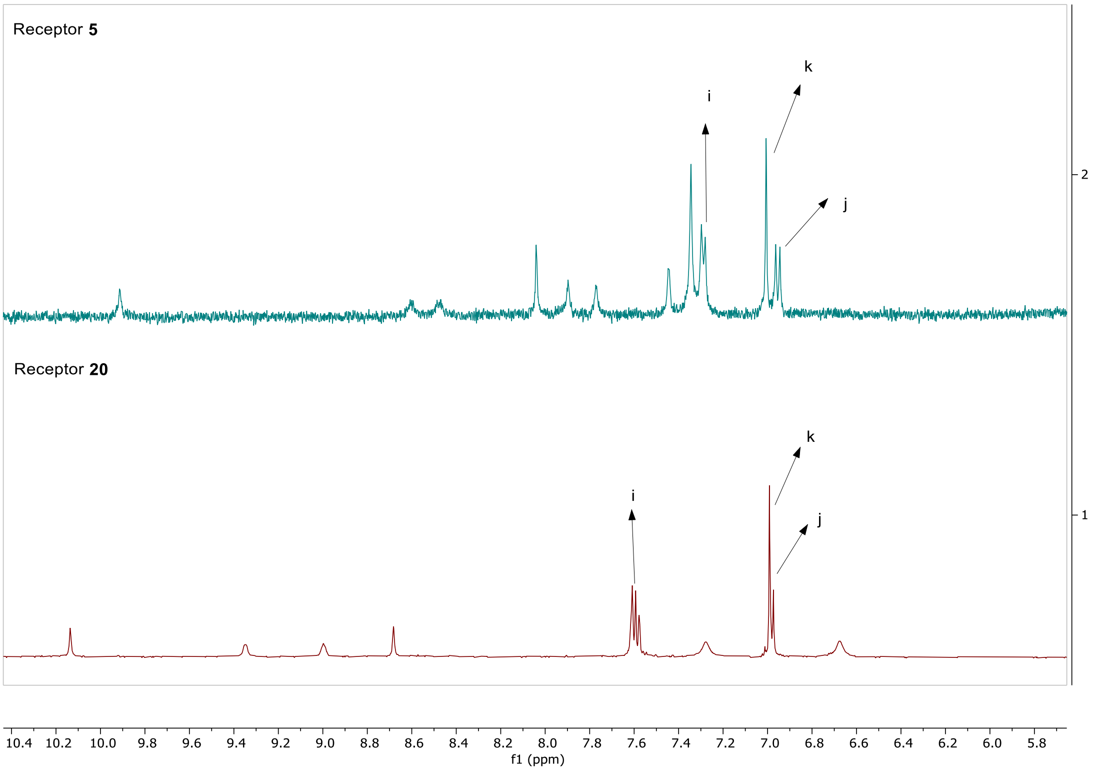

Figure S82. Comparison between ^{1}H NMR spectrum (500 MHz) of receptor **5** (0.022 mM) in 9:1 H_2O/D_2O and ^{1}H NMR spectrum (600 MHz) of receptor **20** (0.17 mM) in DMSO-*d*[6].

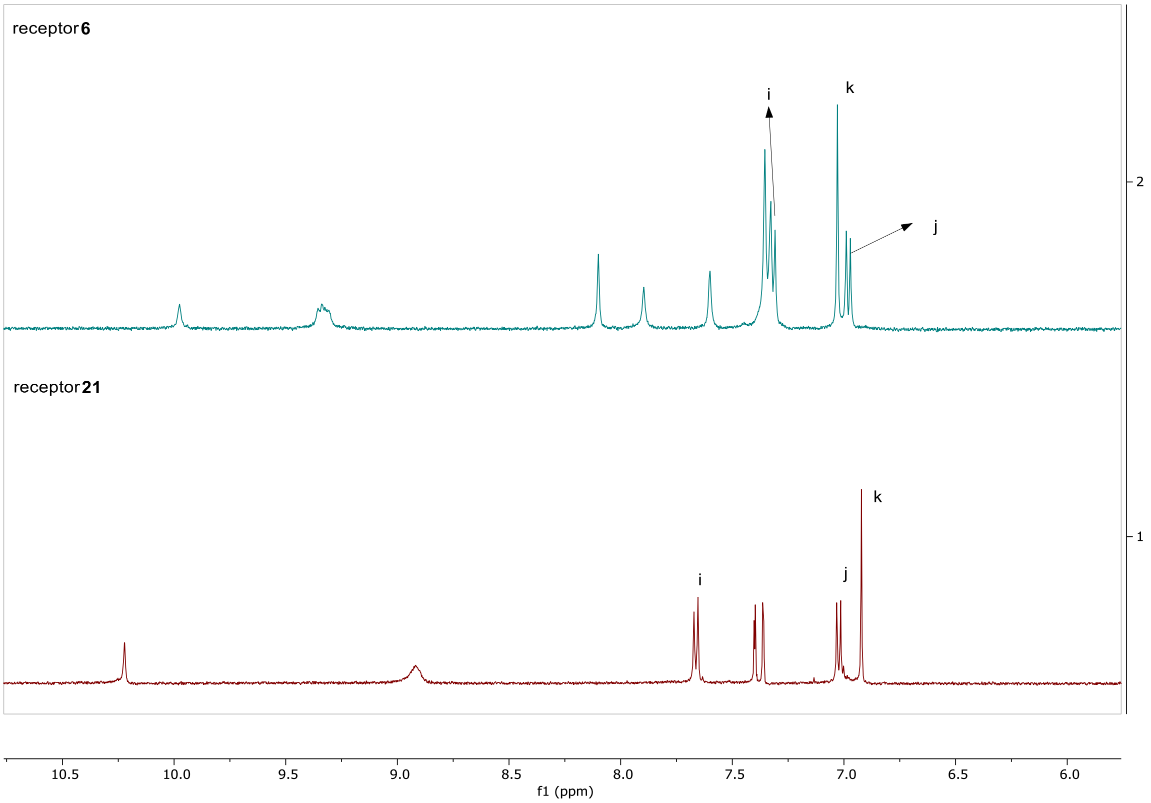

Figure S83. Comparison between ^{1}H NMR spectrum (500 MHz) of receptor **6** (0.056 mM) in 9:1 H_2O/D_2O and ^{1}H NMR spectrum (500 MHz) of receptor **21** (0.1 mM) in DMSO-*d*[6].

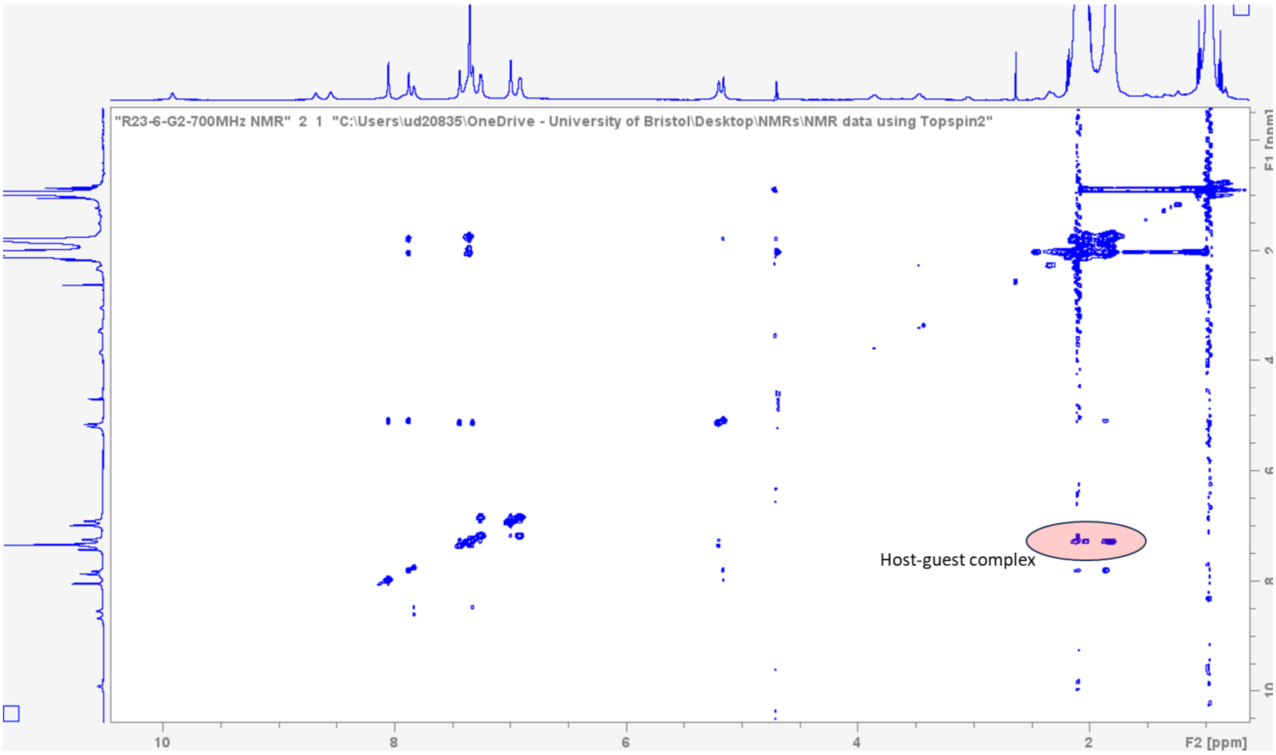
"R23-6-G2-700MHz NMR" 2 1 "C:\Users\ud20835\OneDrive - University of Bristol\Desktop\NMRs\NMR data using Topspin2"
Host-guest complex
F1 [ppm]
F2 [ppm]

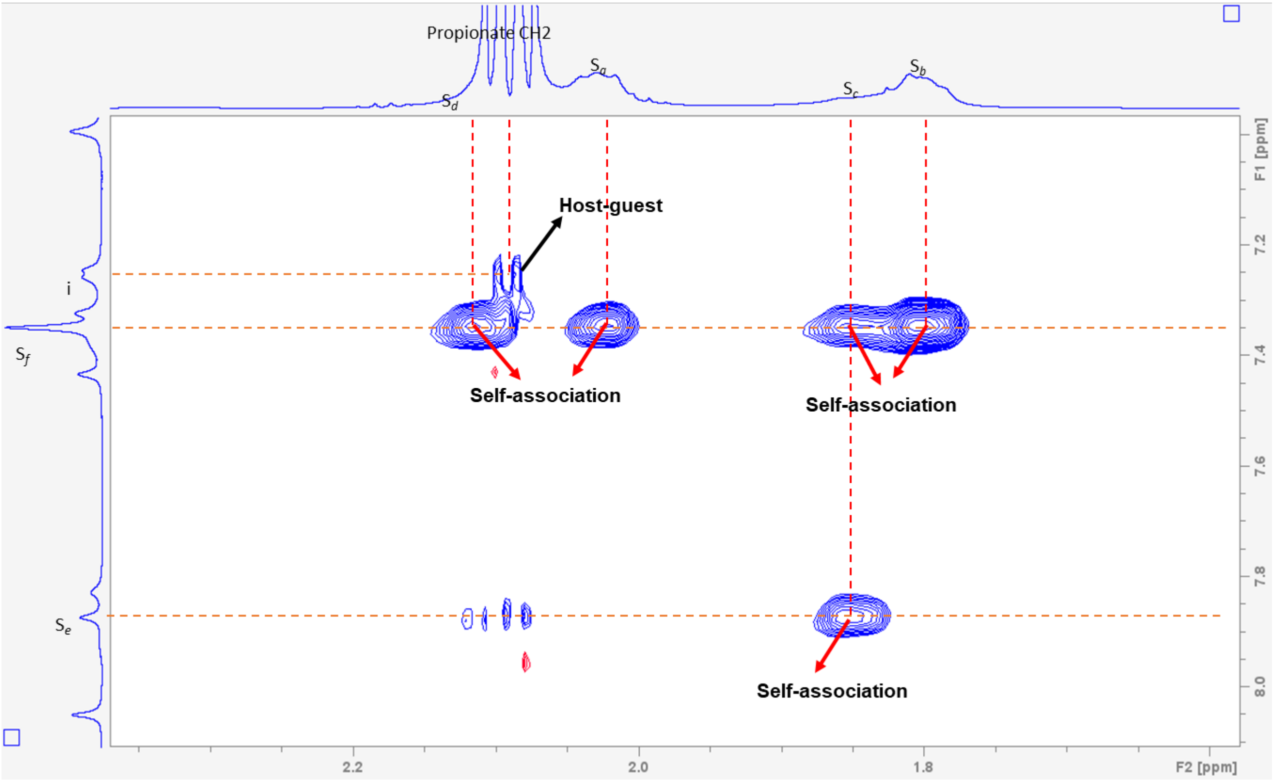
Propionate CH2
Host-guest
Self-association
Self-association
Self-association
F1 [ppm]
F2 [ppm]

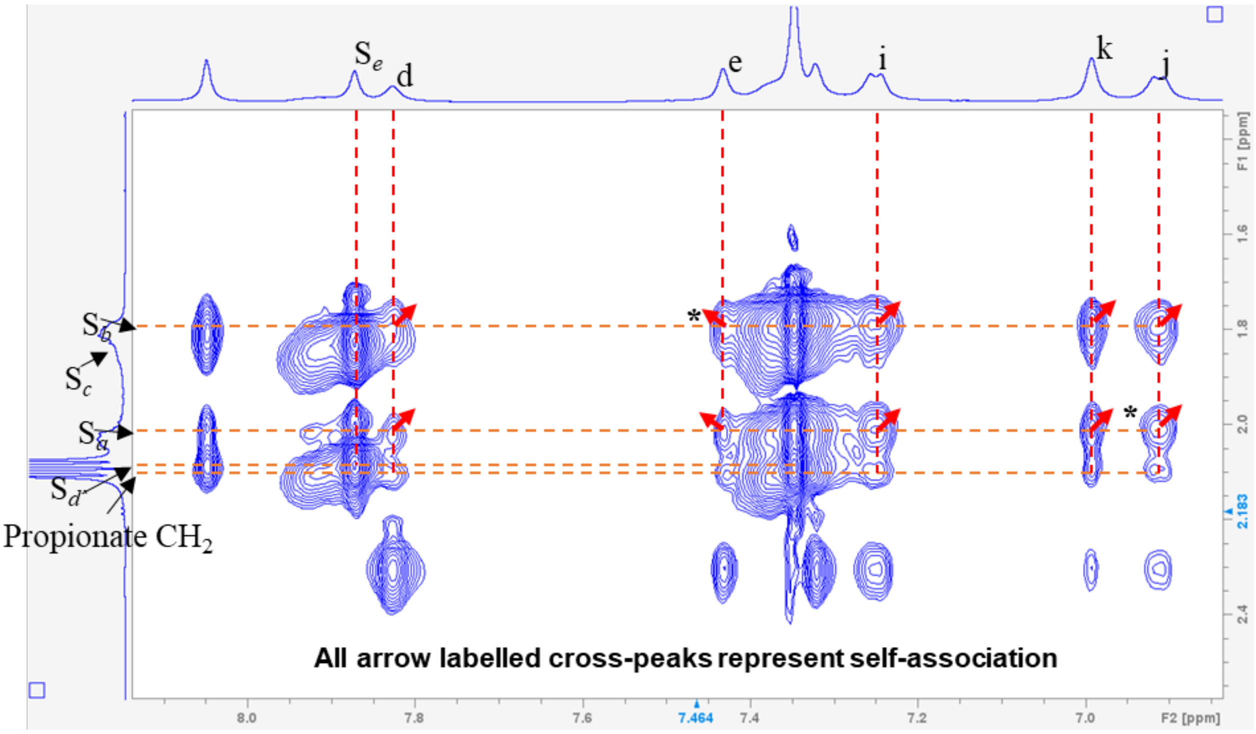

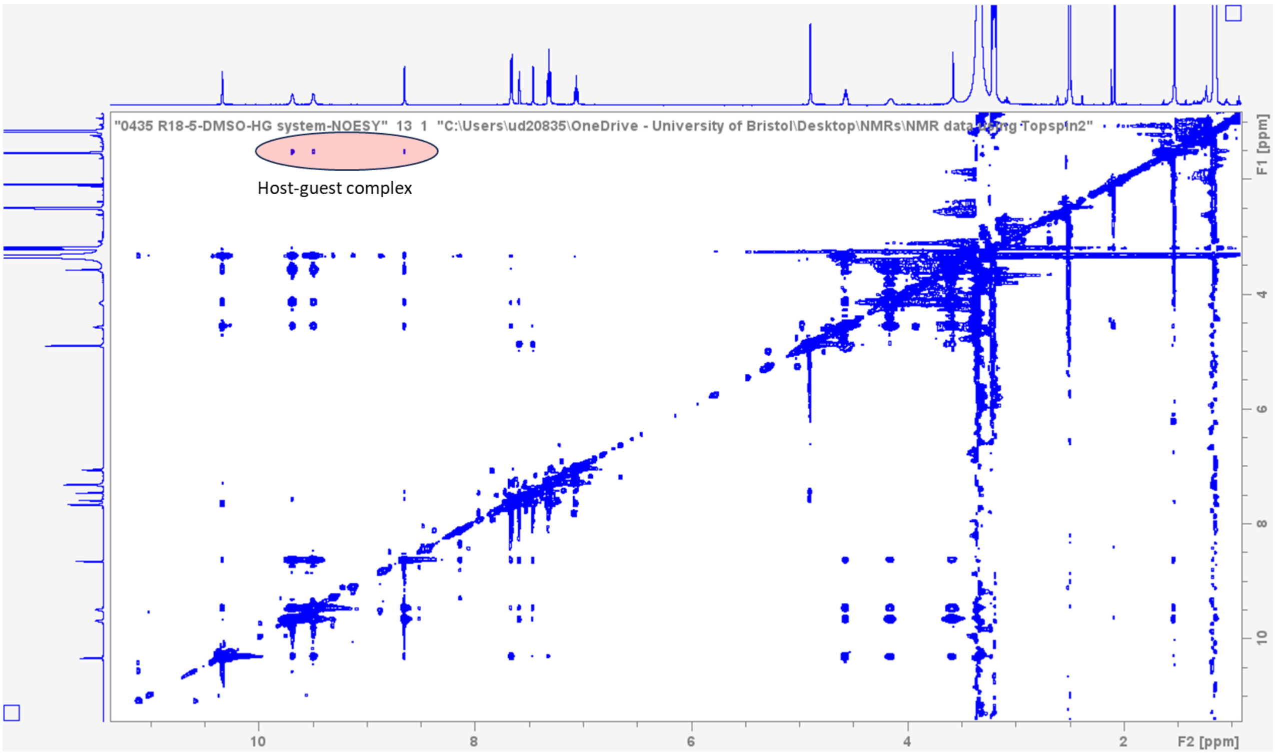

Figure S84. NOESY spectra (700 MHz, 9:1 H_2O /D_2O, 600 ms) of receptor **5** (1.33 mM) + sodium propionate (250 mM).

2.2 Isothermal Titration Microcalorimetry (ITC) titrations

Experimental methods: Isothermal Titration MicroCalorimetry (ITC) experiments were performed on a MicroCal iTC200 microcalorimeter. ITC experiments were carried out at 298 K. Host solutions were prepared by dissolving a weighed amount of solid receptor **5** in HPLC-grade water, the pH was checked to be near-neutral (pH = 7.25). For carboxylate guests (acetate and propionate), the guest stock solution was then prepared by dissolving sodium carboxylate salt in water and adjusting pH to match the host solution as closely as possible (pH = 7.20 – 7.35) by adding the conjugate acid. Other guest (NaCl and Na_2SO_4) stock solutions were prepared by dissolving the compound in water and confirming the neutrality of pH. Guest stock solutions were filtered through WhatmanGD/X disposable filter devices, pore size 0.45 μm. Aliquots (typically 1.0 μL) of guest solution were added o the host solution and the evolution of heat was followed as a function of time. Heats of dilution were measured by injecting the same guest solution into HPLC-grade water using identical conditions.

Data processing: ITC outputs for heat of dilution of substrates and the corresponding binding experiments, as well as analysis curves, are included in Figure S85 – 90. An overview of the binding data, including thermodynamic quantities and errors is given in Table S1 below.

For every addition, the heat of dilution was subtracted from the heat of binding using MicroCal software (MicroCal iTC200 Analysis Add-On Software Package (v7.20) for ORIGIN 7.0). This gave an XY matrix of heat vs. total guest concentration. Obviously bad data was deleted. The data was then analysed using two methods. (1) The matrix was taken to 'One Set of Sites' fitting (N = 2) on MicroCal software to give K_a, ΔG, ΔH and ΔS (Boxes in part *c* of Figures S86 - 90). (2) The matrix was then imported into a specially written Excel programme to fit the data to a 1:2 binding model to give a K_a. ΔG can be derived from K_a and thus ΔS can be derived from ΔH and ΔG using common thermodynamic equations (part *d* of Figures S86 - 90).

The choice of concentrations: ITC experiments were first conducted at [**5**] = 20 μM, which is below the estimated threshold for aggregation. However, due to this low host concentration and low binding constants, the subtracted heat change caused by binding was small and overwhelmed by the error of control experiments (Figure S85). In order to generate analysable data, the host concentration was increased to 250 μM. At this host concentration, the measurable heat change after subtracting the blank was distinct enough to be plotted with good correlation coefficients (*r*). For guest concentrations, propionate at 100 mM and 250 mM were titrated and yielded similar binding results. 250 mM was chosen for guests to maximise the heat change caused by binding.

At a concentration above the monomeric, binding constants (K_a) are expected to be lower due to the self-association. Considering this factor, the results are roughly consistent with those obatined by NMR titrations.

C_{host}	Guest	C_{guest}	K_a (M^{-1})[a]	ΔG (kJ mol^{-1})[a]	ΔH (kJ mol^{-1})[a]	TΔS (kJ mol^{-1})[a]
20 μM	Propionate	250 mM	-[b]	-[b]	-[b]	-[b]
250 μM	Propionate	250 mM	122.0 ± 6.6 %	-11.9	2.2	14.2
250 μM	Propionate	100 mM	123.5 ± 8.9 %	-11.9	3.7	15.6
250 μM	Acetate	250 mM	50.4 ± 1.8 %	-9.7	4.8	14.5
250 μM	Chloride	250 mM	21.3 ± 6.3 %	-7.6	-23.9	-16.3
250 μM	Sulfate	250 mM	230.9 ± 1.2 %	-13.5	1.76	15.3

Table S1. Summary of ITC binding results for receptor **5** with various substrates at 298 K. a. Fitted using an Excel programme (method 2) b. Data cannot be processed.

Receptor 5 (20 µM) & propionate (250 mM)

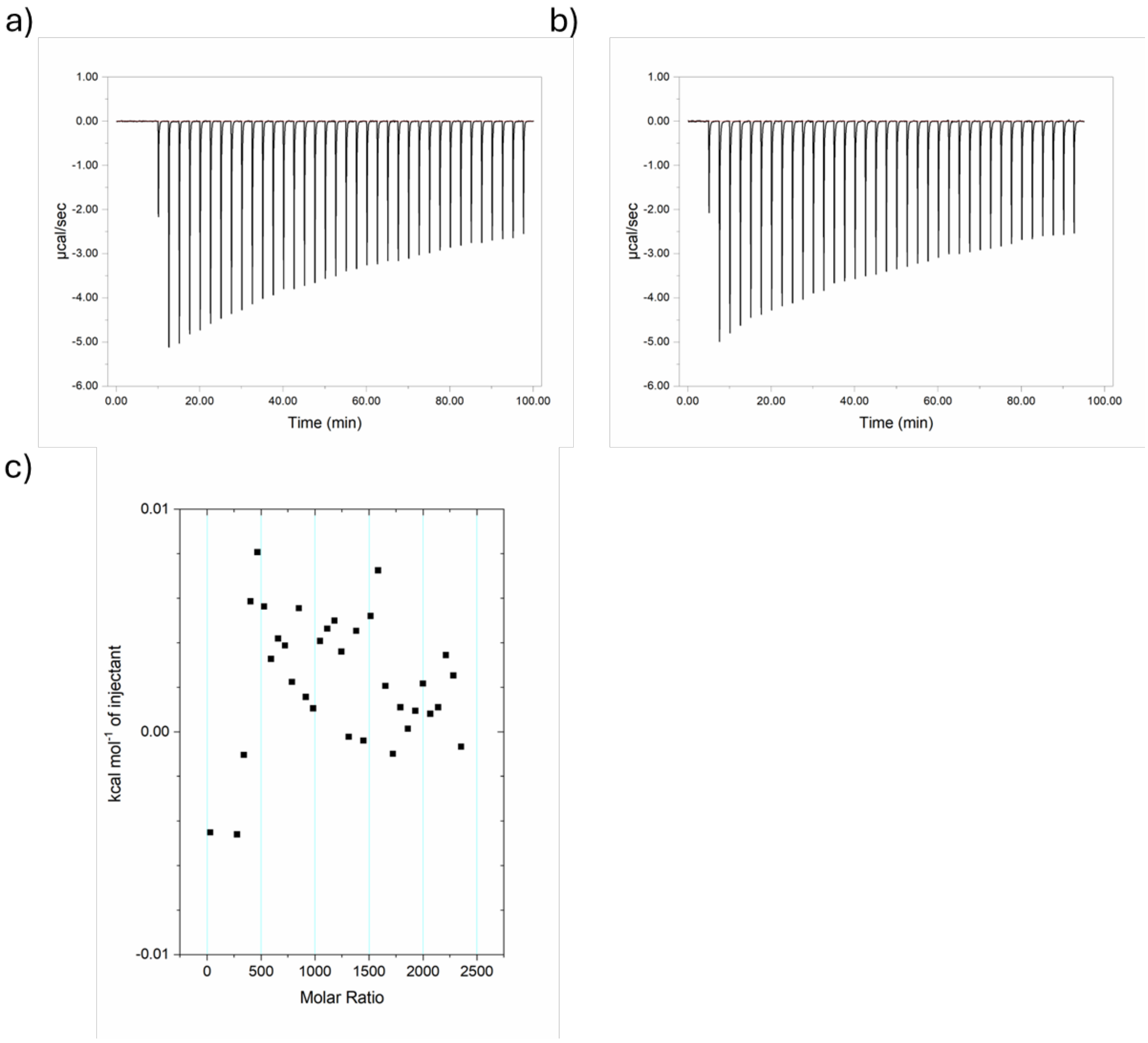

Figure S85. ITC binding results for receptor **5** (25 µM) titrated with sodium propionate (250 mM) in H_2O, in which: a) shows the blank run (addition of substrate into medium); b) shows the titration (substrate into receptor **5**); c) shows the plotted change in enthalpy vs molar ratio. The enthalpy changes are too small to be analysed.

Receptor 5 (250 µM) & propionate (250 mM)

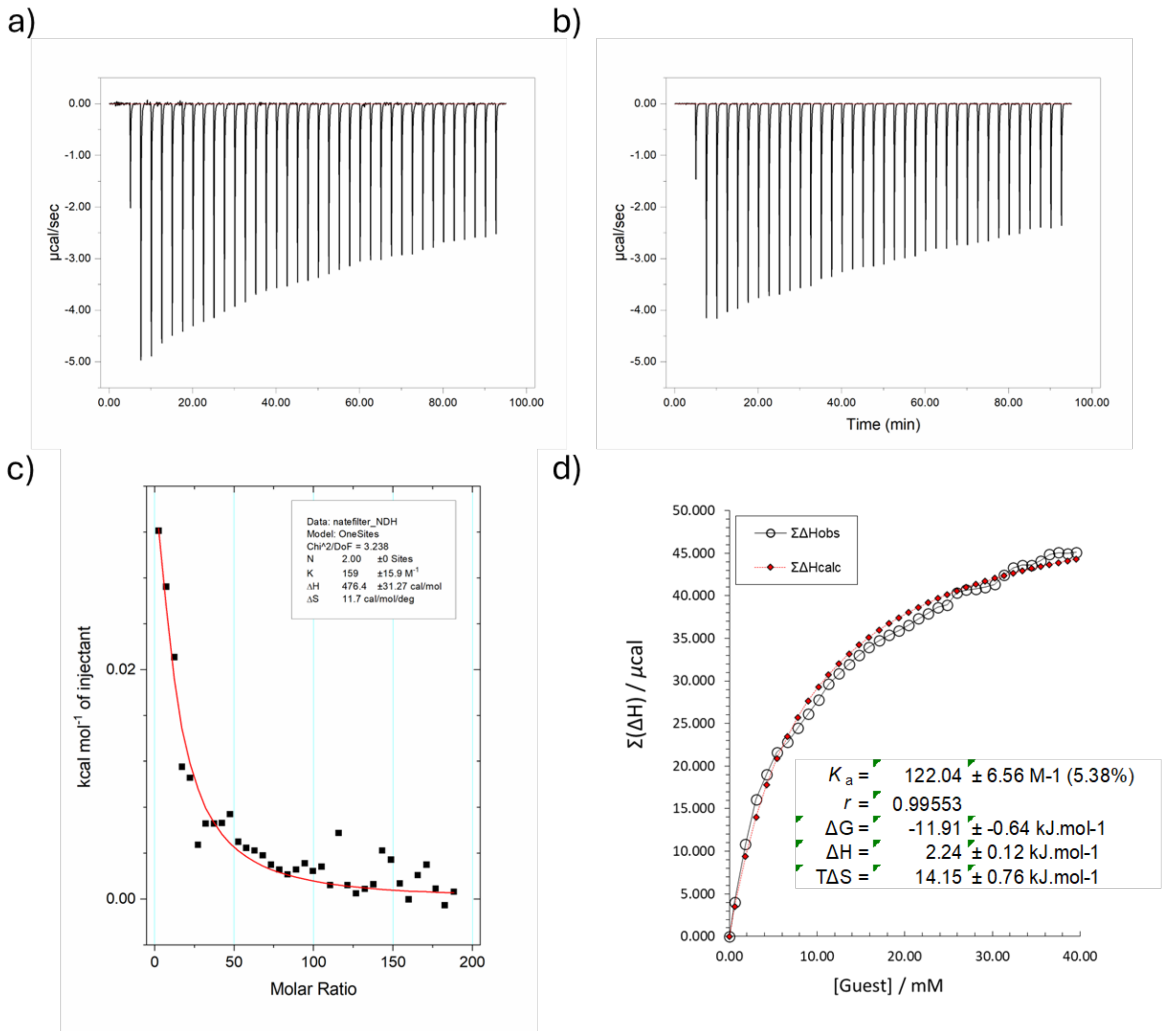

Figure S86. ITC binding results for receptor **5** (25 µM) titrated with sodium propionate (250 mM) in H_2O, in which: a) shows the blank run (addition of substrate into medium); b) shows the titration (substrate into receptor **5**); c) shows the plotted change in enthalpy vs molar ratio. (K_a = 159.0 ± 15.9 M^{-1}) fitted by Microcal Software); and d) shows the fit calculated using an Excel spreadsheet (K_a = 122.0 ± 6.6 M^{-1}).

Receptor 5 (250 µM) & propionate (100 mM)

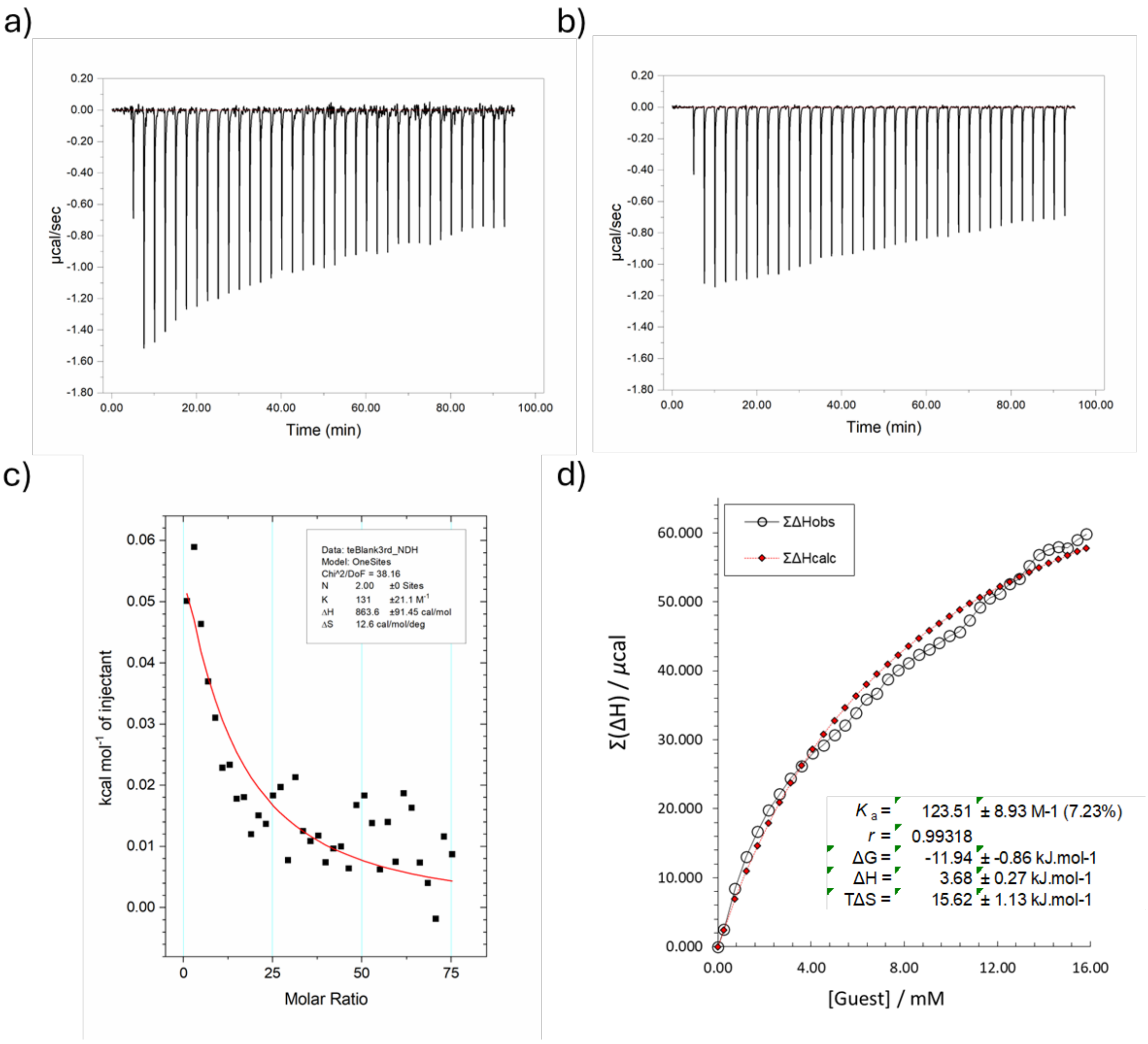

Figure S87. ITC binding results for receptor **5** (250 µM) titrated with sodium propionate (100 mM) in H_2O, in which: a) shows the blank run (addition of substrate into medium); b) shows the titration (substrate into receptor **5**); c) shows the plotted change in enthalpy vs molar ratio. (K_a = 131.0 ± 21.1 M^{-1}) fitted by Microcal Software); and d) shows the fit calculated using an Excel spreadsheet (K_a = 123.5 ± 8.9 M^{-1}).

Receptor 5 (250 μM) & acetate (250 mM)

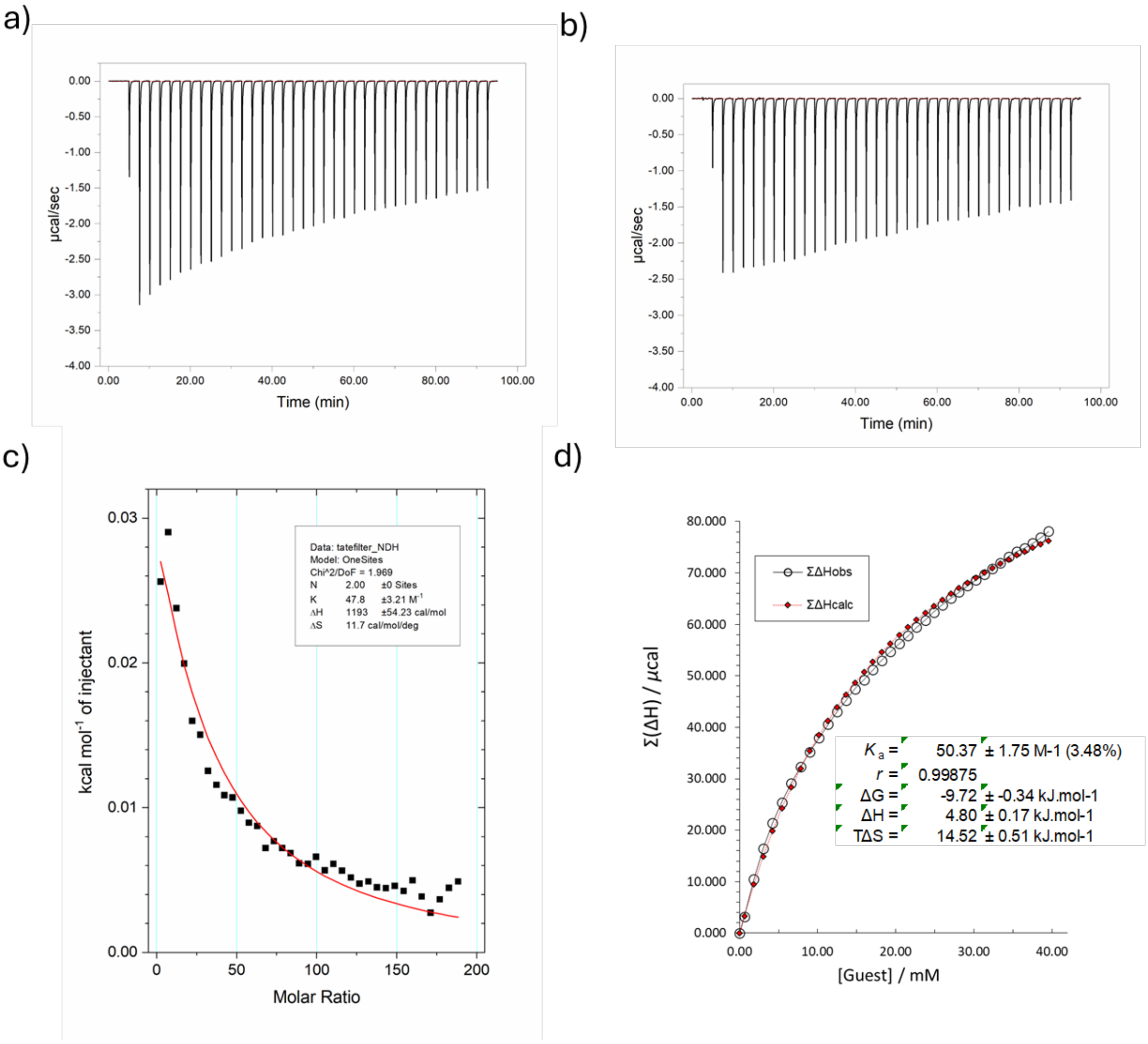

Figure S88. ITC binding results for receptor **5** (250 μM) titrated with sodium acetate (250 mM) in H_2O, in which: a) shows the blank run (addition of substrate into the medium); b) shows the titration (substrate into receptor **5**); c) shows the plotted change in enthalpy vs molar ratio. (K_a = 47.8 ± 3.2 M^{-1}) fitted by Microcal Software); and d) shows the fit calculated using an Excel spreadsheet (K_a = 50.4 ± 1.8 M^{-1}).

Receptor 5 (250 µM) & chloride (250 mM)

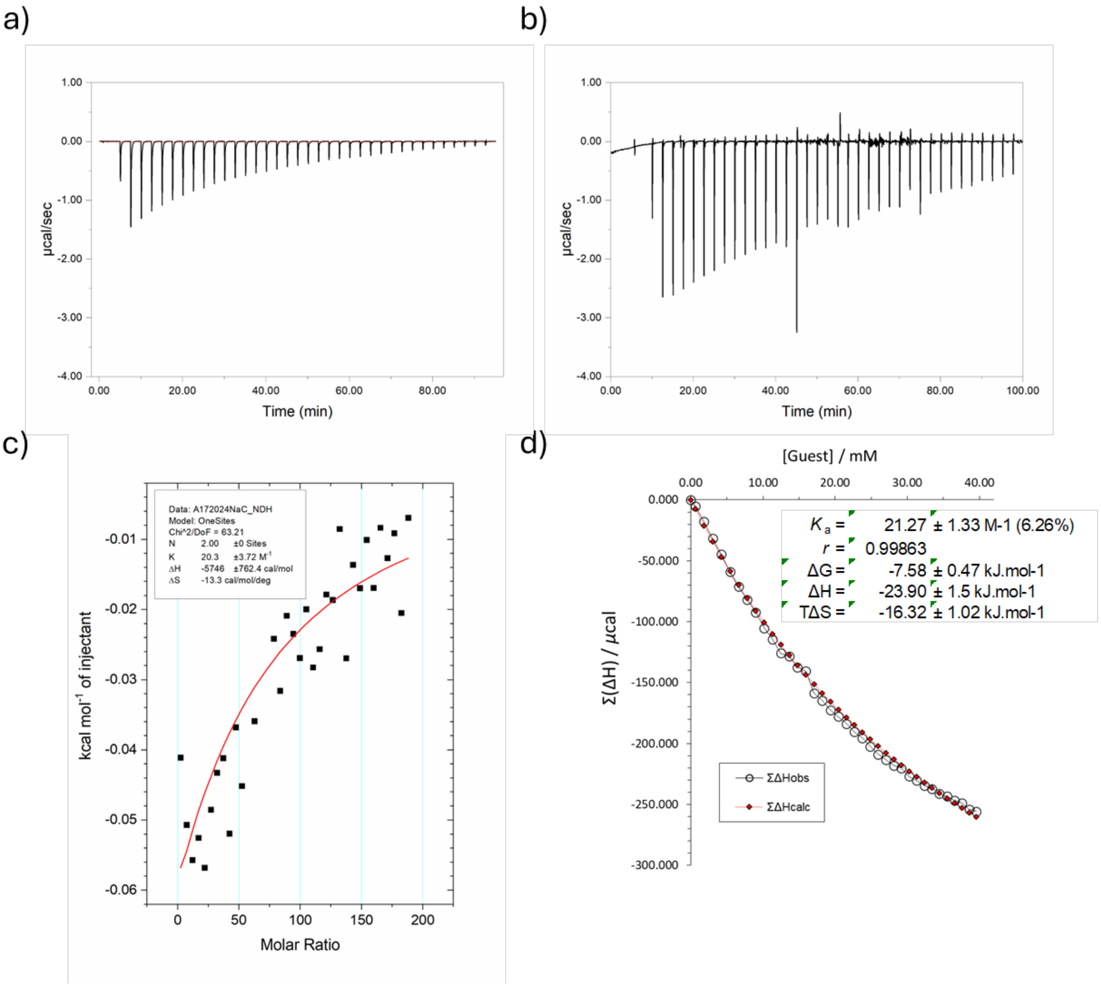

Figure S89. ITC binding results for receptor **5** (250 µM) titrated with sodium chloride (250 mM) in H_2O, in which: a) shows the blank run (addition of substrate into the medium); b) shows the titration (substrate into receptor **5**); c) shows the plotted change in enthalpy vs molar ratio. (K_a = 20.3 ± 3.7 M^{-1}) fitted by Microcal Software); and d) shows the fit calculated using an Excel spreadsheet (K_a = 21.3 ± 1.3 M^{-1}).

Receptor 5 (250 µM) & sulfate (250 mM)

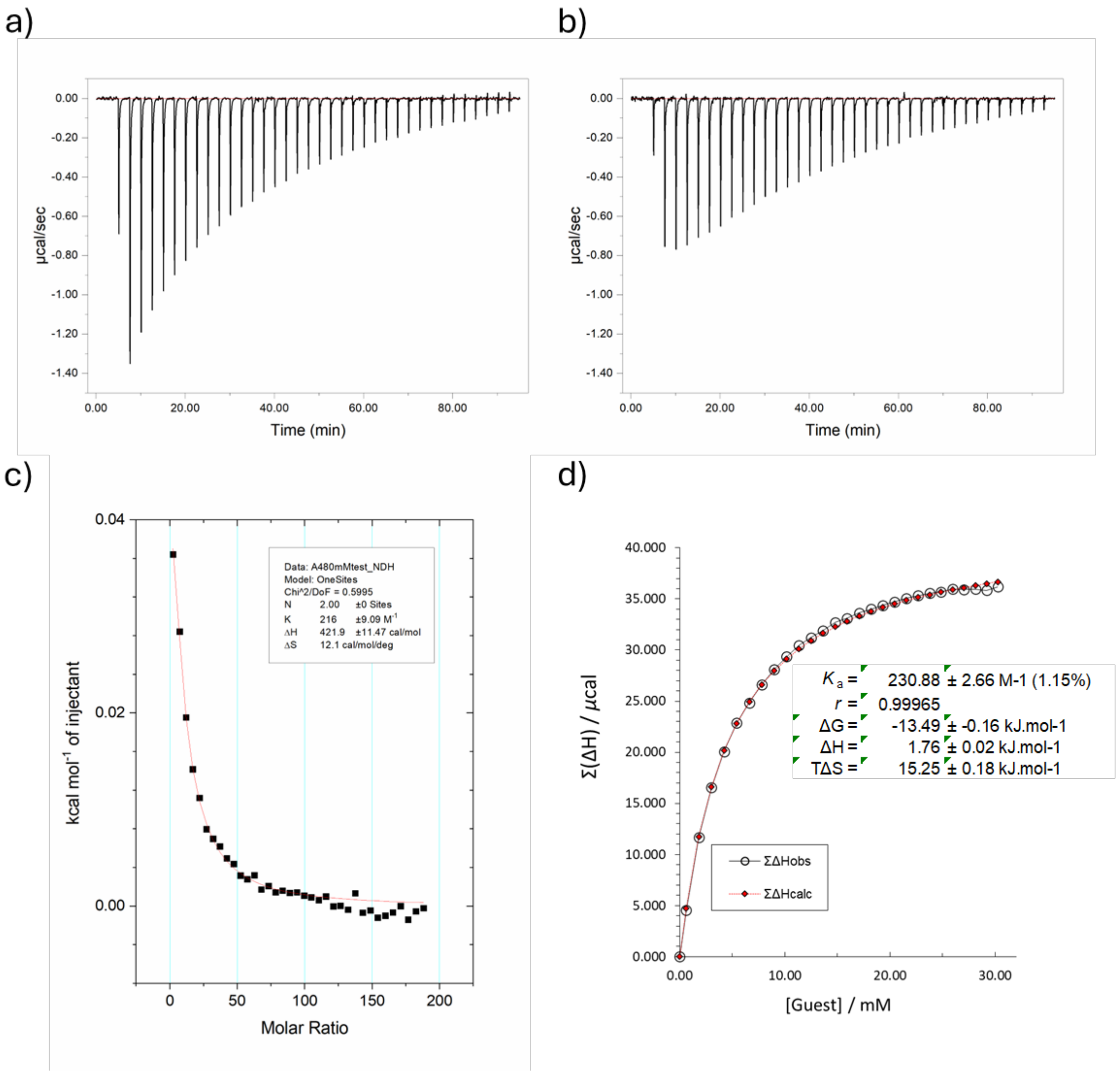

Figure S90. ITC binding results for receptor **5** (250 µM) titrated with sodium sulfate (250 mM) in H_2O, in which: a) shows the blank run (addition of substrate into the medium); b) shows the titration (substrate into receptor **5**); c) shows the plotted change in enthalpy vs molar ratio. (K_a = 216 ± 9.1 M^{-1}) fitted by Microcal Software); and d) shows the fit calculated using an Excel spreadsheet (K_a = 231 ± 2.7 M^{-1}).

3. Modelling studies

Modelling studies employed Maestro Version 13.8, with Batchmin V14.2 for energy minimisation. The calculations employed the OPLS4 force field, aqueous GB/SA solvation, and 4000 MCMM steps.

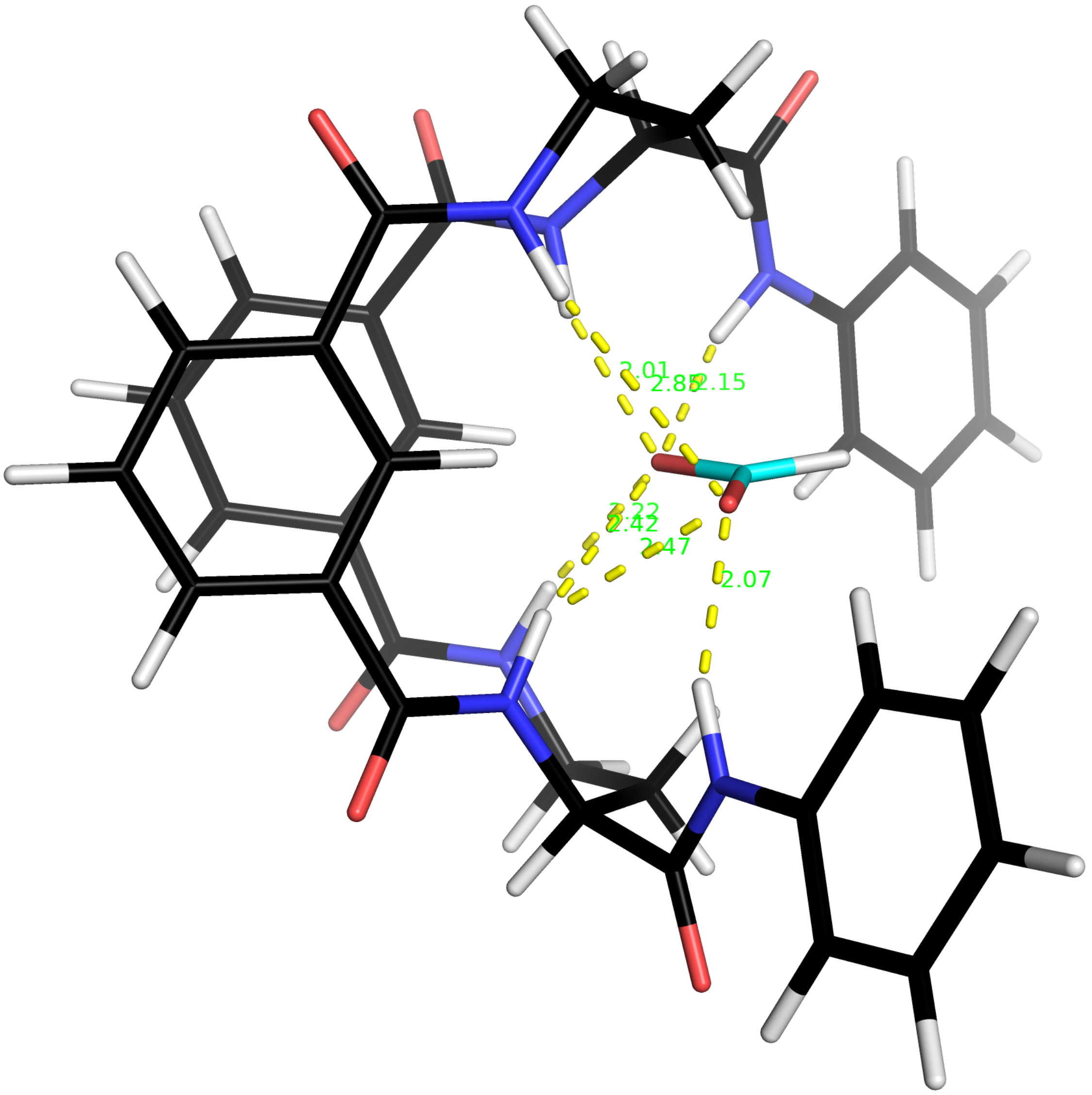

Figure S91. . Lowest energy structure of macrocycle **9** (R = H) bound to formate anion from an MCMM simulation. Conformations were generated by opening of the macrocycle, bond rotations and ring closure. The ground state structure possesses seven short NH···O$^-$ distances ranging from 2.01 – 2.85 Å (yellow broken lines).

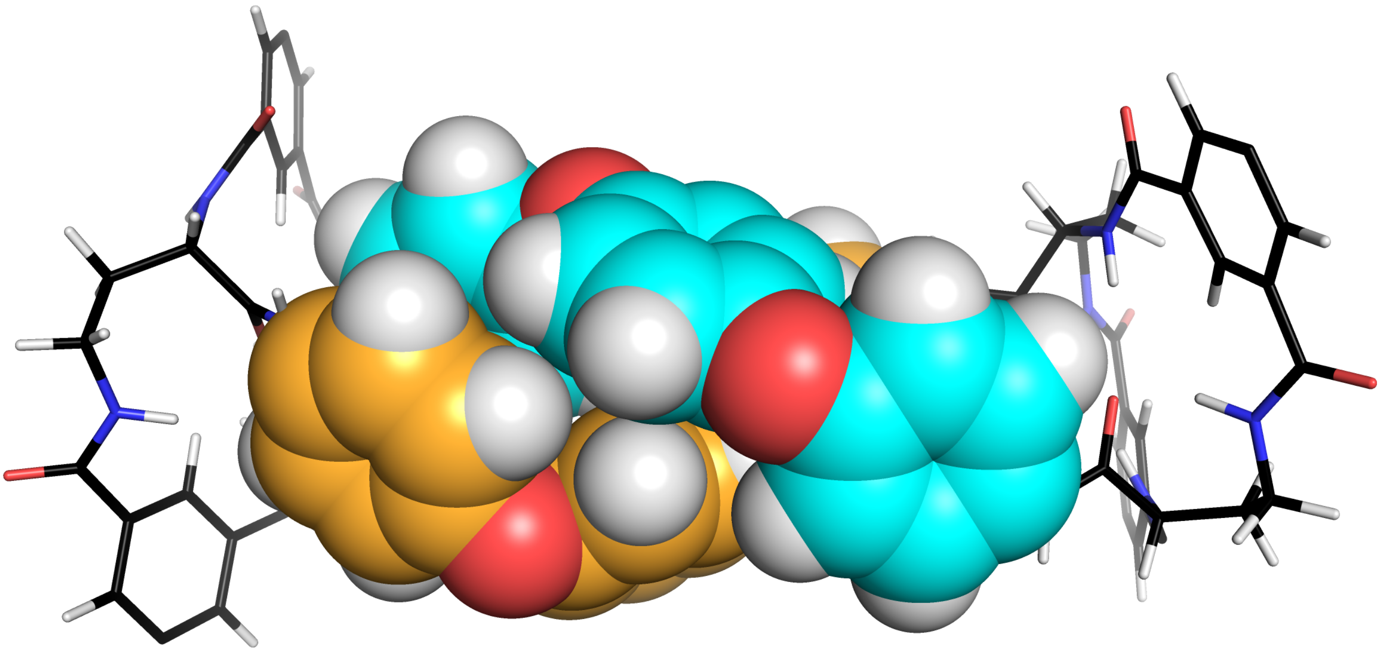

Figure S92. . Energy-minimised structure of receptor **5**, as also shown in Figure 5a, with space-filling representation for the bis-phenoxyphenyl bridges (cyan/gold carbons), and side chains omitted for clarity.

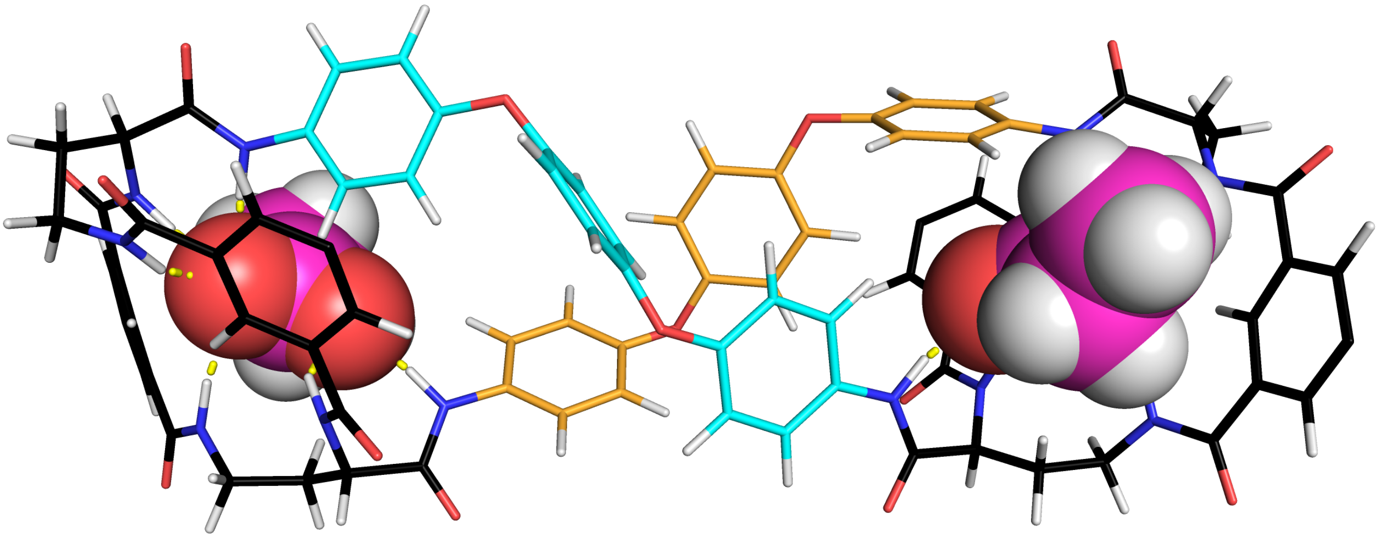

Figure S93. . Energy-minimised structure of receptor **5** binding two propionate anions (magenta carbons) as also shown in Figure 5b. The perspective is chosen to highlight the offset between the bis-phenoxyphenyl bridges in the proposed binding conformation.

1 Destecroix, H. *et al.* Affinity enhancement by dendritic side chains in synthetic carbohydrate receptors. *Angew Chem Int Ed.* **54**, 2057-2061 (2015).

2 Shao, P. *et al.* Synthesis and Evaluation of a Tetra[6,7]quinoxalinoporphyrazine-based Near Infrared Photosensitizer. *RSC Adv.* **7**, 50555-50561 (2017).

3 Sugai, N., Heguri, H., Yamamoto, T. & Tezuka, Y. A Programmed Polymer Folding: Click and Clip Construction of Doubly Fused Tricyclic and Triply Fused Tetracyclic Polymer Topologies. *J. Am. Chem. Soc.* **133**, 19694-19697 (2011).

4 Wang, F. *et al.* Metal coordination mediated reversible conversion between linear and cross-linked supramolecular polymers. *Angew Chem Int Ed.* **49**, 1090-1094 (2010).

5 http://supramolecular.org
